# Spermidine suppresses liver fibrosis by remodeling the communication signal between liver sinusoidal endothelial cells and hepatic stellate cells

**DOI:** 10.1038/s41420-026-03129-4

**Published:** 2026-05-07

**Authors:** Cheng Zeng, Jiao Liu, Zhiqiang Jin, Shan Zhong, Rong Wu, Haoyue Luo, Tao Zeng, Yang Yang, Zhi Zhou

**Affiliations:** 1 Department of Infectious Diseases, Key Laboratory of Molecular Biology for Infectious Diseases (Ministry of Education), Institute for Viral Hepatitis, the Second Affiliated Hospital, Chongqing Medical University, Chongqing, China; 2 Department of Gastroenterology and Hepatology, The Second Affiliated Hospital of Chongqing Medical University, Chongqing, China; 3 Department of Ultrasound, Chengdu Women’s and Children’s Central Hospital, School of Medicine, University of Electronic Science and Technology of China, Chengdu, China

**Keywords:** Ubiquitylation, Imprinting

## Abstract

Hepatic fibrosis is a pivotal stage in which chronic liver disease progresses from reversible injury to decompensation. Liver sinusoidal endothelial cells (LSECs) play a regulatory role in hepatic stellate cells (HSCs) activation through paracrine signaling; therefore, maintaining the physiological phenotype of LSECs is critical for antifibrotic intervention. Spermidine (SPD) has been recognized for its antifibrotic properties; however, its impact on LSECs' function and the underlying mechanism remains largely unknown. In this study, analysis of NHANES data revealed an inverse association between dietary SPD intake and fibrosis risk. Consistently, in vivo and in vitro models demonstrated that SPD significantly ameliorated LSECs dysfunction and attenuated fibrosis progression. Through an integrative analysis incorporating proteomics, public single-cell datasets, and machine-learning prioritization, we identified LSECs-derived biglycan (BGN) as a principal target of SPD; notably, BGN overexpression diminished the capacity of SPD to restore LSECs function and facilitated HSCs activation. Mechanistically, SPD activated NRF2 to increase UBE2G2 expression, thereby enhancing UBE2G2-dependent ubiquitination and degradation of BGN. UBE2G2 knockdown reversed SPD-induced BGN downregulation, subsequently exacerbating LSECs capillarization and enhancing HSCs activation. Furthermore, *Bgn* overexpression in the CCl_4_-induced mouse model markedly attenuated the ability of SPD to improve LSECs dysfunction and its antifibrotic efficacy. In conclusion, our findings uncover a novel mechanism whereby SPD ameliorates LSECs dysfunction and suppresses fibrosis progression by modulating LSECs-derived BGN, suggesting a new therapeutic strategy for liver fibrosis.

**Figure:**
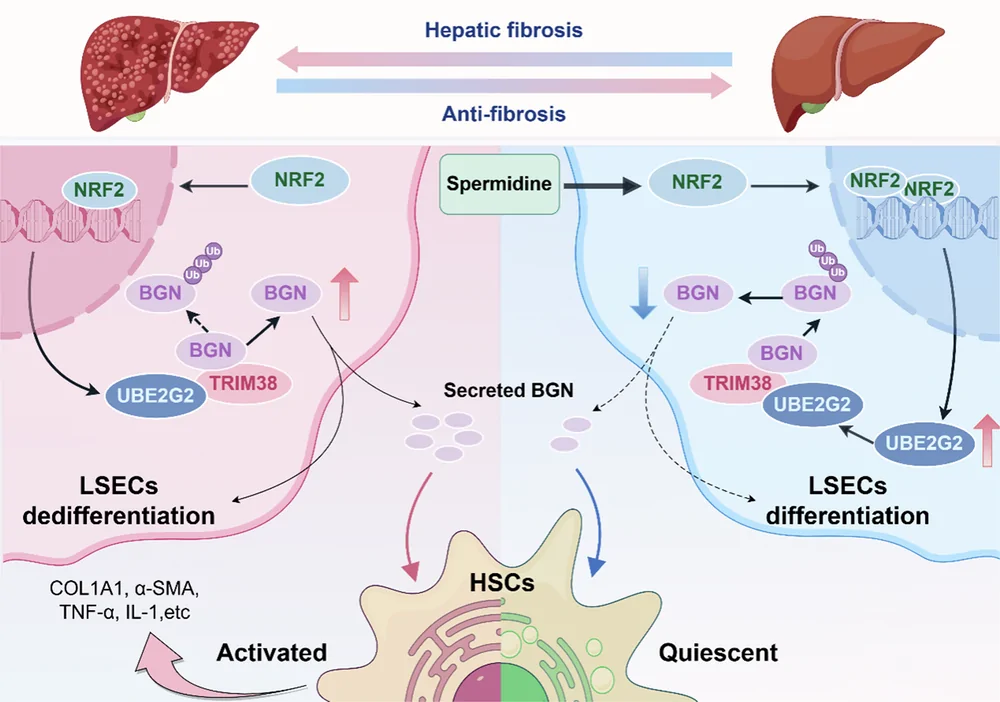
**Schematic working model of the study**. SPD promotes UBE2G2-dependent ubiquitination and proteasomal degradation of BGN, thereby attenuating ERK/p38 phosphorylation, ameliorating LSECs dysfunction, and suppressing HSCs activation.

**Schematic working model of the study**. SPD promotes UBE2G2-dependent ubiquitination and proteasomal degradation of BGN, thereby attenuating ERK/p38 phosphorylation, ameliorating LSECs dysfunction, and suppressing HSCs activation.

## Introduction

Hepatic fibrosis represents a critical pathological stage in the progression of chronic liver disease, with potential advancement to cirrhosis, portal hypertension, and ultimately liver failure [1]. It is characterized by sustained inflammation and the activation of profibrogenic pathways, leading to abnormal accumulation of extracellular matrix (ECM) components within the liver and subsequent remodeling of the hepatic microenvironment [2]. During this process, activated hepatic stellate cells (HSCs)—recognized as the primary producers of collagen—play a central role in driving fibrosis [3]. The activation of HSCs, however, is regulated by a complex network of paracrine signals, including profibrotic and proinflammatory stimuli originating from hepatocytes, Kupffer cells, liver sinusoidal endothelial cells (LSECs), and infiltrating immune cells [4]. Therefore, identification and characterization of the key mediators involved in these intercellular communications may provide novel strategies for modulating HSCs activation and developing antifibrotic therapies.

LSECs are highly specialized cells situated between the sinusoidal lumen and the space of Disse [5]. These cells are characterized by the absence of a continuous basement membrane and the presence of transcellular fenestrae, with approximately 100–150 nm^2^, which facilitate efficient exchange between the blood and hepatic parenchymal cells. Under physiological conditions, LSECs play a crucial role in maintaining sinusoidal architecture and secreting homeostatic signals, thereby fulfilling barrier, scavenging, and regulatory functions [6]. In contrast, chronic exposure to toxic agents, hemodynamic disturbances, or oxidative stress leads to LSECs dysfunction, characterized by basement membrane deposition and the loss of fenestrae—a process known as capillarization [6]. This transdifferentiation not only impairs the exchange of nutrients and metabolites but also activates HSCs through paracrine pathways, including NO signaling and Notch signaling, thereby contributing to hepatic fibrosis [7]. Therefore, restoring or preserving the differentiation of LSECs is regarded as a promising antifibrotic strategy.

Spermidine (SPD), an endogenous polyamine prevalent in mammalian tissues and dietary sources, has recently garnered attention as a metabolite with potential anti-aging properties [8]. Extensive studies show that SPD induces autophagy, enhances mitochondrial homeostasis, reduces oxidative stress, and inhibits the NLRP3 inflammasome, thereby exerting antioxidant, anti-inflammatory, and immune-homeostatic effects [9, 10]. Epidemiological studies further suggest that increased dietary intake of SPD is associated with reduced all-cause mortality and risk of cardiovascular disease [11]. In hepatic research, SPD has been demonstrated to attenuate fibrotic progression by inhibiting HSCs activation through autophagy or TGF-β/Smad signaling pathways [12]; it also provides protection in models of fatty liver disease [13]. Recent evidence indicates that SPD limits hepatic fibrogenesis by preserving mitochondrial function and metabolic homeostasis in hepatic macrophages, accompanied by a shift toward an anti-inflammatory, pro-reparative phenotype [14]. Notably, while SPD significantly enhances endothelial function within the cardiovascular system [10], its role and underlying molecular mechanisms in LSECs—the liver’s specialized vascular bed—remain inadequately characterized. Specifically, it remains to be determined whether SPD could ameliorate LSECs dysfunction and, through the regulation of secreted factors, modulate HSCs activation and fibrogenesis.

Biglycan (BGN) is a small leucine-rich proteoglycan characterized by a 42 kDa core protein with chondroitin sulfate/dermatan sulfate glycosaminoglycan side chains [15]. It is highly expressed in endothelial cells and could be secreted into the extracellular environment. Previous studies have demonstrated that BGN plays important roles in inflammation, tissue remodeling, and endothelial migration and angiogenesis in non-hepatic vascular regions [16–18]. Furthermore, as a secreted factor, BGN contributes to ECM deposition [19]. For example, it facilitates ECM accumulation, HSCs activation and promotes liver fibrosis by upregulating HSP47 [20]. Nevertheless, the regulatory mechanisms of BGN expression in LSECs and its paracrine effects on HSCs activation remain largely unknown and require systematic investigation.

In this study, we demonstrate for the first time that SPD ameliorates LSECs dysfunction and suppresses HSCs activation through downregulation of LSECs-derived BGN. Specifically, SPD promotes UBE2G2-dependent ubiquitination and proteasomal degradation of BGN, thereby attenuating ERK/p38 phosphorylation, restoring the endothelial phenotype, and inhibiting hepatic fibrosis. These findings reveal an SPD–UBE2G2–BGN axis, which remodels LSECs–HSCs paracrine communication and highlights maintenance of the LSECs phenotype as a promising antifibrotic therapeutic strategy.

## Results

### Dietary SPD intake is inversely associated with liver fibrosis risk

**Fig. 1:**
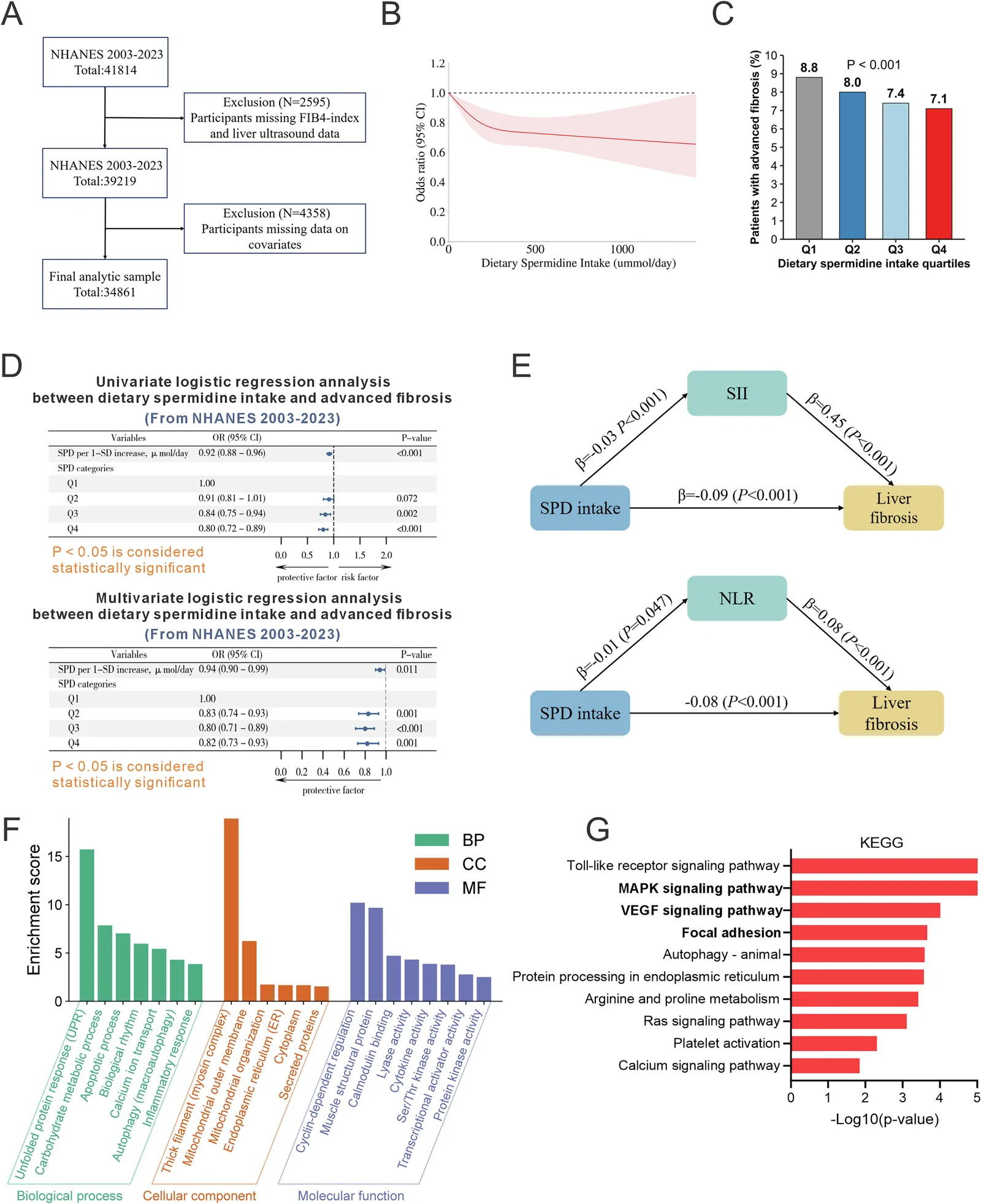
Association between dietary spermidine (SPD) intake and risk of liver fibrosis. **A** Study design flowchart. **B** Restricted cubic spline (RCS) analysis of the multivariable-adjusted association between dietary SPD intake and liver fibrosis; the y-axis shows odds ratios (ORs), and the shaded band indicates the 95% confidence interval (CI). **C** Bar plots showing between-group differences in ALT (alanine aminotransferase), AST (aspartate aminotransferase), and CRP (C-reactive protein) across quartiles of dietary SPD intake: Q1 ≤ 1.7; 1.74 < Q2 ≤ 2.5; 2.5 < Q3 ≤ 3.9; Q4 > 3.9 (units as defined in the Methods). **D** Univariable and multivariable logistic regression analyses of the association between dietary SPD intake and liver fibrosis. Covariates in the adjusted model: age, sex, race, marital status, poverty-income ratio, education level, smoking status, physical activity, and alanine aminotransferase. **E** Mediation pathway illustrating the role of inflammatory markers in the association between dietary SPD intake and liver fibrosis. **F**, **G** GO and KEGG analysis of the overlapped targets using the network pharmacology approach of SPD intake and liver fibrosis.

To investigate whether dietary SPD intake is associated with liver fibrosis risk, we included 34,861 eligible participants from the NHANES database (Fig. 1A). A restricted cubic spline curve revealed an inverse association between dietary SPD intake and liver fibrosis risk (Fig. 1B). When participants were stratified by quartiles of SPD intake, the prevalence of liver fibrosis was significantly lower in the highest intake group (*P* < 0.001, Fig. 1C).

Logistic regression analysis further demonstrated that, in the unadjusted model, each standard deviation increase in SPD intake was associated with a decreased risk of liver fibrosis (OR = 0.92, 95% CI: 0.88–0.96). After adjusting for potential confounders, including age, sex, BMI, race, alcohol consumption, smoking status, educational level, income, diabetes, hypertension, and physical activity, dietary SPD intake persisted as an independent protective factor against liver fibrosis (OR = 0.94, 95% CI: 0.90–0.99; Fig. 1D). Mediation analysis further suggested that the systemic immune-inflammation index (SII) and the neutrophil-to-lymphocyte ratio (NLR) mediated, at least in part, the association between dietary SPD intake and liver fibrosis (Fig. 1E).

To investigate the potential molecular mechanisms underlying this association, we conducted an integration of multiple disease databases alongside a network pharmacology analysis. We extracted liver fibrosis-related targets from OMIM, GeneCards, DisGeNET, and TTD, resulting in 6,605 unique disease-associated targets after removing duplicates (Table S1). Concurrently, 215 potential targets of SPD were identified using the SwissTargetPrediction and CTD databases (Table S2). An intersection analysis identified 138 common targets between spermidine and liver fibrosis (Figs. S1, 2 and Table S3). Functional enrichment analysis of the 138 identified targets demonstrated significant enrichment in several critical biological processes, notably the regulation of endoplasmic reticulum-associated protein degradation and the modulation of secreted protein functions. Subsequent KEGG pathway enrichment analysis revealed that these targets were significantly enriched in the MAPK signaling pathway (including the p38 and JNK pathways), the VEGF signaling pathway, focal adhesion, and Toll-like receptor signaling pathways, all of which are intricately associated with endothelial cell function and the progression of fibrosis (Fig. 1F, G). These findings imply that the antifibrotic effects of SPD may be intricately linked to the regulation of endothelial function, the maintenance of secreted protein homeostasis, and the degradation of endoplasmic reticulum-associated proteins.

### SPD attenuates hepatic fibrosis through the amelioration of LSECs dysfunction

**Fig. 2:**
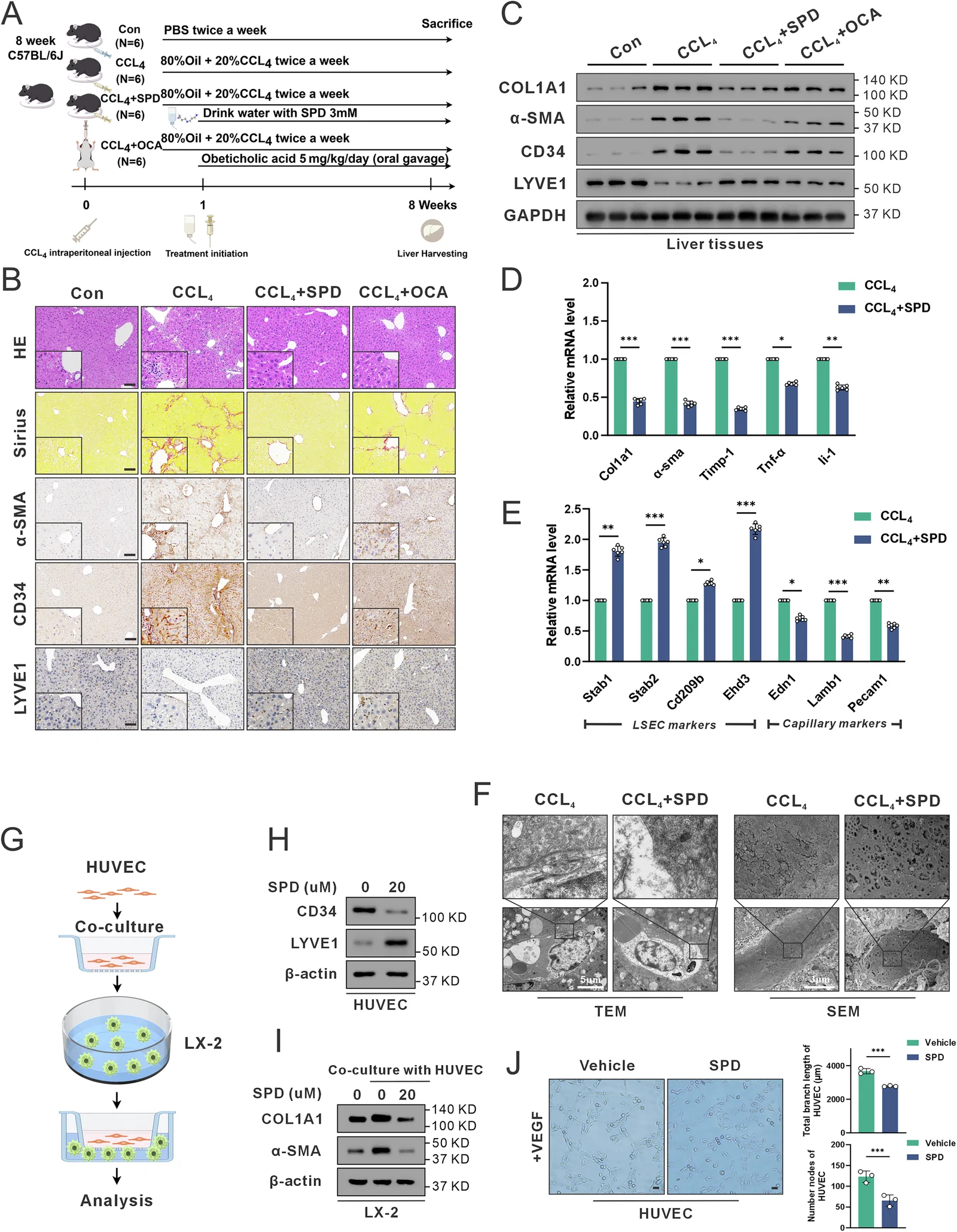
SPD attenuates hepatic fibrosis through the amelioration of LSECs dysfunction. **A** Schematic diagram of the SPD treatment protocol. 8-week-old C57BL/6 J mice (*n* = 6) received intraperitoneal injections of CCl₄ for 8 consecutive weeks to induce liver fibrosis. From the second week onward, SPD (3 mM) was added to the drinking water, and obeticholic acid (OCA, 5 mg/kg) was administered daily via oral gavage for the remaining 7 weeks. **B** Representative images of H&E and Sirius Red staining, as well as immunohistochemical staining for α-SMA, CD34, and LYVE1 in liver sections (bar = 100 µm). **C** Analysis of protein levels of COL1A1, α-SMA, CD34, and LYVE1 in liver tissues by western blot (*n* = 3, performed in biological replicates). **D**, **E** Analysis of mRNA levels of COL1A1, α-SMA, TIMP-1, TNF-α, IL-1, LYVE1, STAB1, STAB2, CD209B, EHD3, CD34, EDN1, LAMB1 and PECAM1 in liver tissues by RT-qPCR (*n* = 6, performed in biological replicates). **F** Representative TEM and SEM images of liver tissues from CCl₄ and CCl₄ +SPD group. **G** Schematic of the co-culture model. LX-2 cells were co-cultured with SPD-treated (or untreated) HUVEC. **H** Analysis of protein levels of CD34 and LYVE1 in HUVEC. **I** Analysis of protein levels of COL1A1 and α-SMA expression in co-cultured LX-2 cells. **J** Matrigel tube-formation assay in HUVEC (*n* = 3, performed in biological replicates). Cells were pretreated with SPD (20 μM) for 24 h, then seeded on matrigel-coated 48-well plates and stimulated with VEGFA (25 ng/mL) for 24 h. Images were captured 24 h after seeding. Data are represented as mean ± SD, *P < 0.05, **P < 0.01, ***P < 0.001.

To elucidate the role of SPD in hepatic fibrosis development and its underlying mechanisms, a CCl₄-induced mouse model of liver fibrosis was established. One week post-induction, mice were administered SPD in the drinking water (3 mM) or OCA (positive control, 5 mg/kg, oral gavage) (Fig. 2A). After 7 weeks of intervention, mice were euthanized for the assessment of liverweight, body weight and serum biochemistry. Compared with the CCl₄ group, SPD treatment significantly ameliorated CCl₄-induced abnormalities, including elevated body weight, liver weight, liver/body weight ratio and levels of ALT and AST (Fig. S2A), thereby confirming its hepatoprotective properties. Histological analyses, including H&E, Sirius red staining, and α-SMA immunostaining, demonstrated that SPD substantially decreased collagen deposition and fibrogenesis compared to CCl₄ group (Fig. 2B). Notably, SPD significantly reduced the CCl₄-induced expansion of CD34 and enhanced LYVE1-positive areas (Fig. 2B), indicating effective inhibition of LSECs capillarization.

To further investigate the regulatory impact of SPD on LSECs function and pathways associated with fibrosis, we examined gene expression in liver tissues of CCl₄-treated mice (primer sequences are listed in Supplementary Table S4). CCl₄ induction markedly upregulated the fibrotic markers, including COL1A1 and α-SMA, as well as inflammatory mediators such as TIMP-1, TNF-α, and IL-1, whereas SPD intervention significantly reduced these elevations (Fig. 2C, D). Moreover, SPD substantially increased the expression of canonical LSECs markers (LYVE1, STAB1, SRAB2, CD209B, and EHD3), while concurrently decreasing the expression of markers associated with capillary endothelial cells (CD34, EDN1, LAMB1, and PECAM) (Fig. 2C, E). Importantly, TEM analysis revealed reduced and discontinuous basement-membrane deposition along the sinusoidal wall in SPD-treated mice relative to the CCl₄ group (Fig. 2F). SEM further showed restoration of sieve-like plates, with a higher density of fenestrae and more uniform diameters (Fig. 2F). Together, these results suggest that SPD suppresses LSECs capillarization and restores their distinctive phenotype, thereby effectively attenuating fibrosis progression.

In liver disease research, human umbilical vein endothelial cells (HUVEC) are commonly used as a surrogate model for LSECs [21, 22]. Therefore, we conducted co-culture experiments using a VEGF-stimulated HUVEC and the human hepatic stellate cell line LX-2 to verify the functional crosstalk between LSECs and HSCs (Fig. 2G). SPD treatment markedly upregulated the expression of LSECs markers such as LYVE1, while downregulating capillary endothelial markers such as CD34 (Figs. 2H and S2B). In the co-culture system, direct co-culture alone did not elicit appreciable changes in fibrosis-related markers in LX-2 cells. In contrast, when LX-2 cells were co-cultured with SPD-treated HUVEC, the expression of fibrosis-associated markers in LX-2 cells was markedly reduced (Figs. 2I and S2C). In tube-formation assays, SPD significantly decreased the total tube length and number of branch points in HUVEC (Fig. 2J), indicating its ability to inhibit pathological angiogenesis. To overcome the limitations of HUVEC in modeling the specialized biological features of LSECs, we isolated primary LSECs and HSCs and established a co-culture system (Fig. S2D). Consistently, SPD attenuated the capillarization phenotype of LSECs, and HSCs co-cultured under these conditions exhibited a concomitant reduction in pro-fibrotic responses (Fig. S2E–G).

Collectively, these results demonstrate that SPD suppresses capillarization of LSECs and restores their characteristic phenotype; consequently, it inhibits HSCs activation through an LSECs-to-HSCs paracrine signaling axis, thereby limiting collagen deposition and fibrotic progression.

### SPD suppresses LSECs capillarization and HSCs activation through downregulation of LSECs-derived BGN

**Fig. 3:**
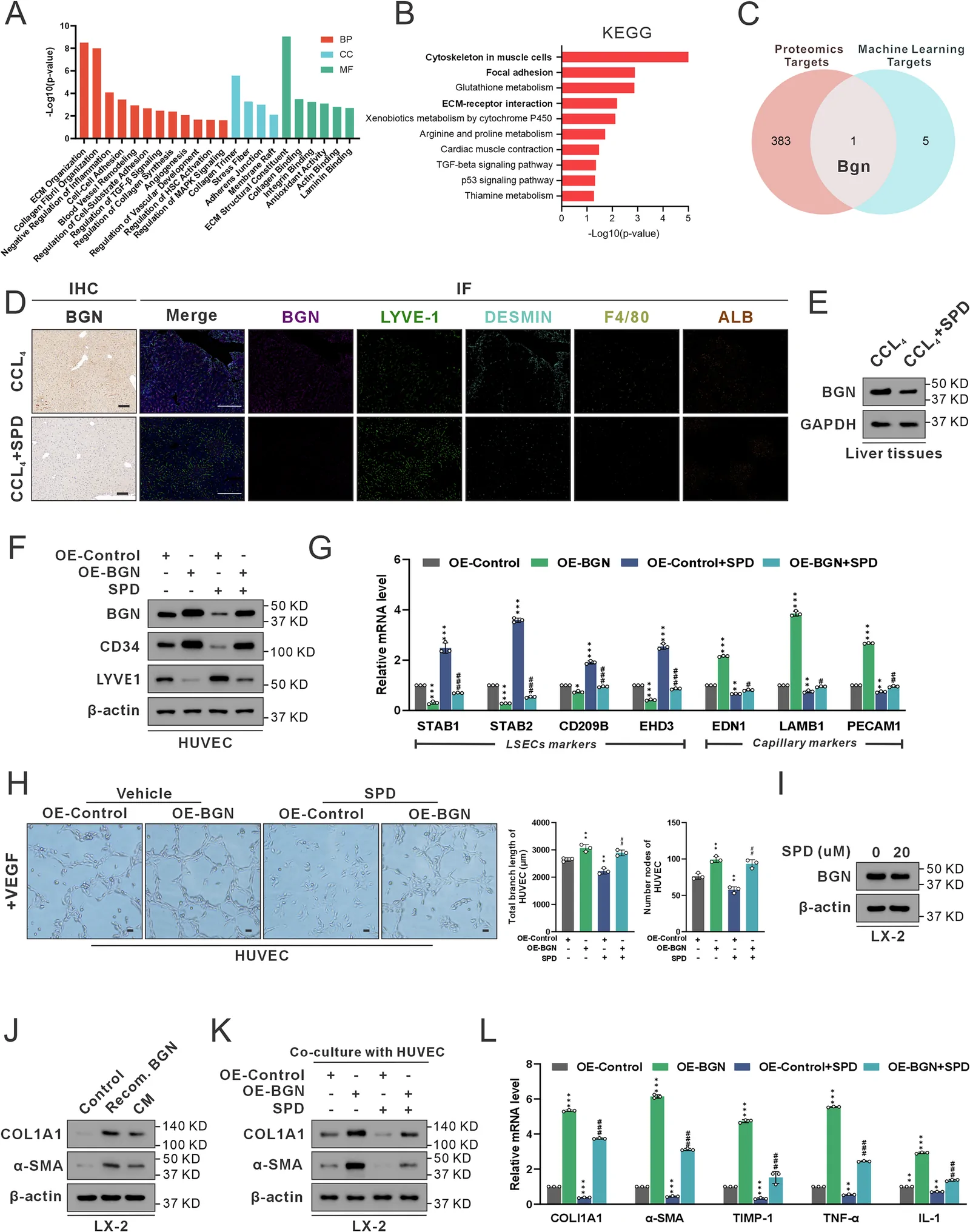
SPD suppresses LSECs capillarization and HSCs activation through downregulation of LSECs-derived BGN. **A**, **B** GO and KEGG enrichment analysis of differentially expressed proteins. **B** Volcano plot of differentially expressed proteins (|FC|≥ 1.5, *P*-value < 0.05). **C** Venn diagram illustrating the overlapping targets between proteomic profiling and machine learning-processed single-cell RNA-seq datasets. **D** Immunohistochemistry (bar = 100 µm) showing BGN staining in liver sections. Immunofluorescence (bar = 100 µm) showing co-immunostaining of BGN with markers for LSECs (LYVE-1), hepatocytes (ALB), Kupffer cells (F4/80), and HSCs (DESMIN). Nuclei were counterstained with DAPI. **E** Analysis of BGN protein levels in liver tissues. HUVEC were infected with adenoviruses AdBGN to overexpress BGN (OE-BGN) or infected with AdControl (OE-Control) for 36 h, followed by treatment with or without SPD (20 μM) for 24 h. **F** Protein levels of BGN, CD34, and LYVE1. **G** Relative mRNA levels of STAB1, STAB2, CD209B, EHD3, CD34, EDN1, LAMB1, and PECAM1 (*n* = 3, performed in biological replicates). **H** HUVEC were seeded on matrigel-coated 48-well plates and treated with VEGFA for an additional 24 h, then images were captured (*n* = 3, performed in biological replicates). **I** Analysis of protein levels of BGN after SPD treatment in LX-2 cells. **J** LX-2 cells were serum-starved overnight and then treated with recombinant BGN protein (100 ng/mL) or conditioned medium (CM) from BGN-overexpressing HUVEC for 48 h, followed by western blot analysis. **K**, **L** Protein levels of COL1A1 and α-SMA, and relative mRNA levels of COL1A1, α-SMA, TIMP-1, TNF-α, and IL-1 in LX-2 cells co-cultured with SPD-treated OE-BGN or OE-Control HUVEC. For (**G**, **H**, **L**), * indicated OE-BGN or OE-Control + SPD vs OE-Control, ^#^ indicated OE-BGN + SPD vs OE-Control + SPD; Data are represented as mean ± SD, *P < 0.05, **P < 0.01, ***P < 0.001; ^#^*P* < 0.05, ^##^*P* < 0.01, ^###^*P* < 0.001.

To elucidate the anti-fibrotic mechanism of SPD through the LSECs–HSCs axis, we conducted proteomic profiling of liver tissues from mice treated with CCl₄ and CCl₄+SPD. Gene Ontology (GO) and Kyoto Encyclopedia of Genes and Genomes (KEGG) enrichment analyses indicated that the differentially expressed proteins were predominantly associated with endothelial cell development, angiogenesis and extracellular matrix formation, with significant enrichment observed in the TGF-β signaling pathway (Fig. 3A, B). Furthermore, we interrogated the publicly available GEO single-cell RNA-sequencing dataset (GSE268846; Fig. S3A) to characterize genes with high expression specificity in LSECs. To ensure robust feature selection, PPI combined four complementary machine-learning algorithms—least absolute shrinkage and selection operator (LASSO) regression, support vector machine (SVM), artificial neural network (ANN), and random forest (RF)—were implemented, with the dataset randomly partitioned into training and validation cohorts at a 7:3 ratio (Fig. S3B–D). Each algorithm independently yielded the top 10 weighted core biomarkers (Fig. S3E). Integrative analysis combining these machine-learning-derived candidates with our proteomic profiling data (Fig. 3C) converged on secreted BGN as a critical target, predominantly localized to LSECs. These results suggest that BGN may play an important role in the pathological phenotypic conversion of LSECs and contribute to fibrotic progression. Subsequently, we examined the expression of BGN following SPD treatment. In the CCl₄-induced mouse model, SPD significantly reduced the protein level of BGN in liver tissues (Fig. 3D, E). IF analysis further demonstrated that BGN predominantly colocalized with LSECs markers, specifically LYVE1, while exhibiting minimal overlap with markers of hepatocytes (ALB), Kupffer cells (F4/80), and HSCs (Desmin). This finding supports an LSECs-enriched distribution of BGN, which was reduced following SPD treatment (Fig. 3D).

To assess the impact of BGN on LSECs function, we developed shRNA constructs to knock down BGN. Functional assays showed that BGN knockdown in HUVEC significantly increased the expression of LSECs markers while decreasing the expression of capillary endothelial markers (Fig. S3F, G). Subsequently, we engineered the adenoviral recombinant AdBGN. The overexpression of BGN notably facilitated LSECs capillarization and largely counteracted amelioration of LSECs dysfunction induced by SPD (Fig. 3F, G). Consistently, tube-formation assays demonstrated that BGN overexpression enhanced the tube-forming ability of HUVEC and increased the number of branch points, whereas BGN overexpression significantly mitigated the anti-angiogenic effects of SPD (Figs. 3H and S3H). We next investigated the underlying mechanism. Prior studies have reported that BGN modulates endothelial phenotype via the ERK/p38 pathway [23]. Consistently, network pharmacology and proteomic enrichment analysis also revealed significant enrichment of the MAPK pathway, prompting us to hypothesize that SPD ameliorates endothelial dysfunction by downregulating BGN and thereby modulating MAPK signaling. Western blot revealed that BGN overexpression markedly increased phosphorylation of ERK and p38—but not JNK—and largely reversed the SPD-induced suppression of MAPK signaling (Fig. S3I). These findings indicate that SPD improves endothelial function by downregulating BGN, which in turn inhibits activation of the ERK/p38 pathway.

We subsequently investigated the impact of SPD on HSCs and elucidated the functional role of BGN within this framework. Firstly, we examined the effect of SPD treatment on BGN levels. Direct exposure of LX-2 cells to SPD did not result in any significant alterations in endogenous BGN levels (Fig. 3I). Considering that BGN is more likely to function as a secreted mediator derived from LSECs in intercellular communication, we evaluated the influence of extracellular BGN on HSCs activation. Under monoculture conditions, LX-2 cells were either treated with recombinant human BGN protein or incubated with conditioned medium from BGN-overexpressing HUVEC. Both recombinant BGN and conditioned medium from BGN-overexpressing HUVEC markedly induced LX-2 cells activation and significantly upregulated the expression of several pro-fibrotic proteins (Fig. 3J). These findings support the hypothesis that secreted BGN facilitates pro-fibrotic responses in HSCs through a paracrine mechanism. Subsequently, an LSECs–HSCs co-culture system was established to assess the cross-cellular effects of BGN on HSCs activation. LX-2 cells co-cultured with HUVEC transduced with a BGN shRNA lentivirus showed a marked reduction in fibrotic marker expression (Fig. S3J, K). In contrast, overexpression of BGN significantly diminished the SPD-mediated antifibrotic phenotype (Fig. 3K, L). Collectively, these findings indicate that BGN, a secreted factor derived from LSECs, promotes HSCs activation and hepatic fibrosis by enhancing ERK/p38-mediated LSECs capillarization, whereas SPD counteracts this process through downregulation of BGN.

### SPD downregulates BGN protein level through NRF2-dependent UBE2G2 upregulation

**Fig. 4:**
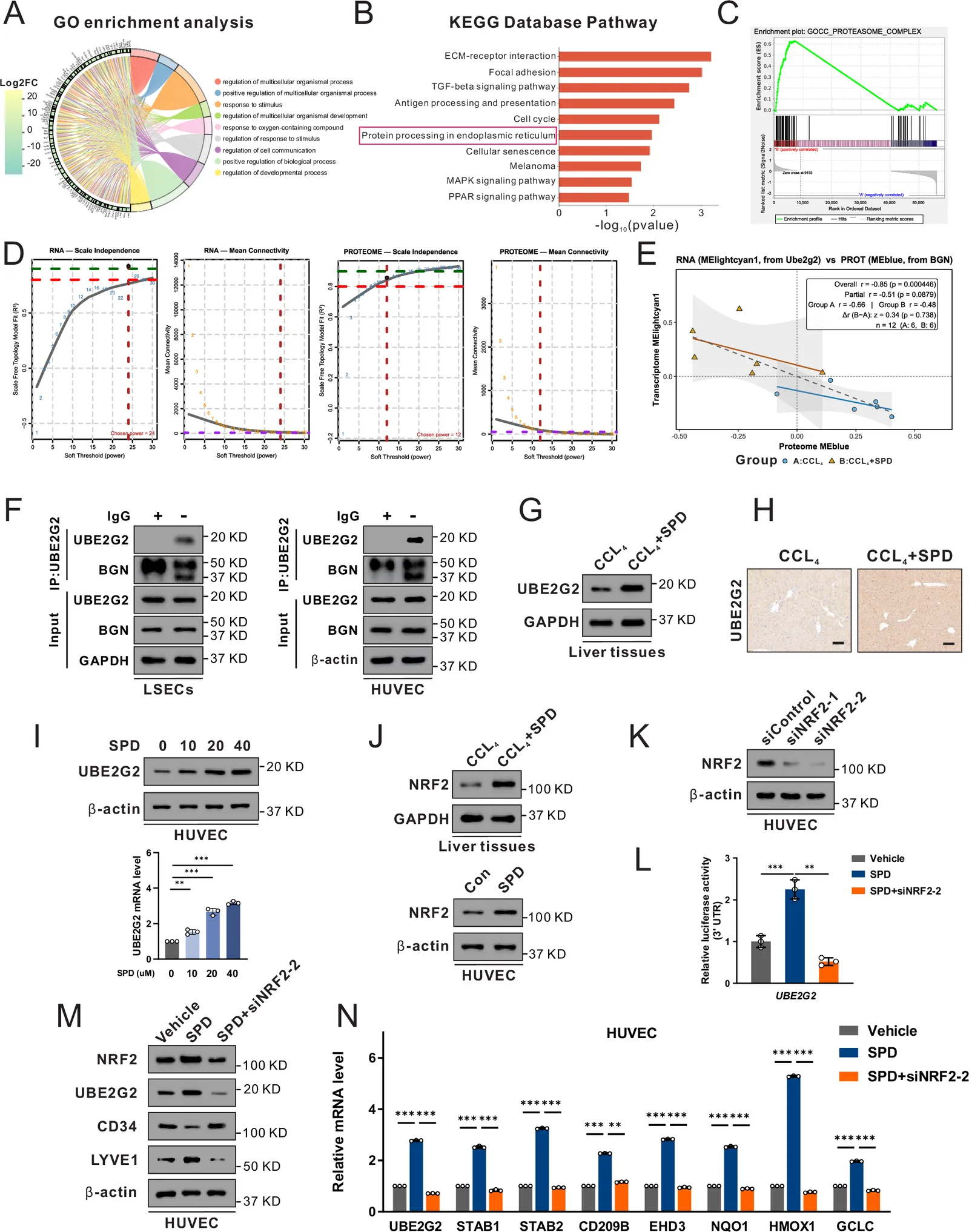
SPD upregulates UBE2G2 via activation of NRF2. **A**–**C** GO, KEGG, and GSEA enrichment analyses of differentially expressed genes. **D** WGCNA soft-thresholding power selection plot. **E** Correlation Between UBE2G2-Associated Transcriptomic Module (MElightcyan1) and BGN-Associated Proteomic Module (MEblue) in WGCNA. **F** Verification of the interaction between UBE2G2 and BGN by Co-IP assay. **G**, **H** UBE2G2 expression in liver tissues by western blot and immunohistochemical staining (bar = 100 µm). **I** Analysis of UBE2G2 expression in HUVEC treated with different concentrations of SPD by western blot and RT-qPCR (*n* = 3, performed in biological replicates). **J** NRF2 expression in liver tissues and HUVEC by western blot. HUVEC were transfected with NFR2 or Control siRNA for 48 h, followed by treatment with SPD for 24 h. **K** Analysis of NRF2 expression in HUVEC by western blot. **L** Relative luciferase activity of constructs containing 3’UTR of UBE2G2 was determined by luciferase reporter assay (*n* = 3, performed in biological replicates). **M**, **N** Western blot and RT-qPCR analyses of indicated genes. Data are represented as mean ± SD, *P < 0.05, **P < 0.01, ***P < 0.001.

Proteomic profiling, coupled with experimental validation, confirmed that BGN is downregulated following SPD treatment, while its mRNA levels remain unchanged (Fig. S4A). Additionally, treatment with the proteasome inhibitor MG132 reversed the SPD-induced reduction in protein BGN levels (Fig. S4B). These suggest a post-transcriptional regulatory mechanism for protein BGN. To further investigate the mechanism by which SPD downregulates the secreted protein BGN, we conducted RNA-seq on liver tissues from mice treated with CCl₄ and CCl₄+SPD. GO and KEGG enrichment analyses revealed that the differentially expressed genes (DEGs) involved in endoplasmic reticulum protein folding and degradation were largely enriched (Fig. 4A–C), suggesting that SPD likely facilitates the degradation of protein BGN via the ubiquitin–proteasome pathway.

To elucidate the key regulatory gene, we conducted individual overexpression of significantly enriched ubiquitin ligases and evaluated their impact on BGN expression (Fig. S4C). Through integration with WGCNA, these results identified the E2 ubiquitin-conjugating enzyme UBE2G2 as the crucial regulator (Figs. 4D, E and S4D, E). Notably, this is consistent with our network pharmacology enrichment implicating ER protein homeostasis and secreted-protein regulation (Fig. 1F, G). Subsequent Co-IP assays confirmed the interaction between UBE2G2 and BGN (Fig. 4F), leading to the designation of UBE2G2 as an upstream regulator of BGN. Further investigation of UBE2G2 expression in a CCl₄-induced mouse model revealed that SPD treatment significantly elevated UBE2G2 levels (Figs. 4G, H and S4F). In cell models, UBE2G2 expression exhibited a dose-dependent increase in response to SPD treatment (Figs. 4I and S4G). In vitro assays revealed that while UBE2G2 was expressed in both LSECs and HSCs, SPD treatment induced a more pronounced upregulation of UBE2G2 in LSECs (Fig. S4H). These findings suggest that UBE2G2 primarily modulates BGN expression within LSECs.

Nuclear factor erythroid 2-related factor 2 (NRF2) is a transcription factor involved in the regulation of fibrosis, and previous research has demonstrated that NRF2 could enhance *UBE2G2* promoter activity [24, 25]. In alignment with findings that SPD could activate NRF2, western blot revealed a significant increase in NRF2 protein levels in liver tissues and LSECs following SPD treatment (Figs. 4J and S4I). Moreover, the regulation of NRF2 by SPD is dependent on KEAP1(Fig. S4J). Subsequently, chromatin immunoprecipitation (ChIP) assay confirmed the occupancy of NRF2 at the *UBE2G2* promoter region, and NRF2 binding was significantly increased following SPD treatment (Fig. S4K). Luciferase reporter assays showed that SPD significantly elevated *UBE2G2* promoter activity, an effect that was notably diminished by NRF2 knockdown (Fig. 4K, L). Therefore, we demonstrated that SPD activated NRF2 to enhance *UBE2G2* promoter activity, thereby upregulating UBE2G2 expression. Consistently, NRF2 knockdown attenuated the SPD-mediated inhibition of the LSECs capillarization and the upregulation of antioxidant target genes (Fig. 4M, N). Overall, these results indicate that SPD enhances UBE2G2 expression through NRF2 activation; in turn, UBE2G2 likely promotes BGN degradation via the ubiquitin–proteasome pathway, thereby suppressing the capillarization phenotype of LSECs.

### SPD downregulates BGN by promoting UBE2G2-mediated ubiquitination degradation

**Fig. 5:**
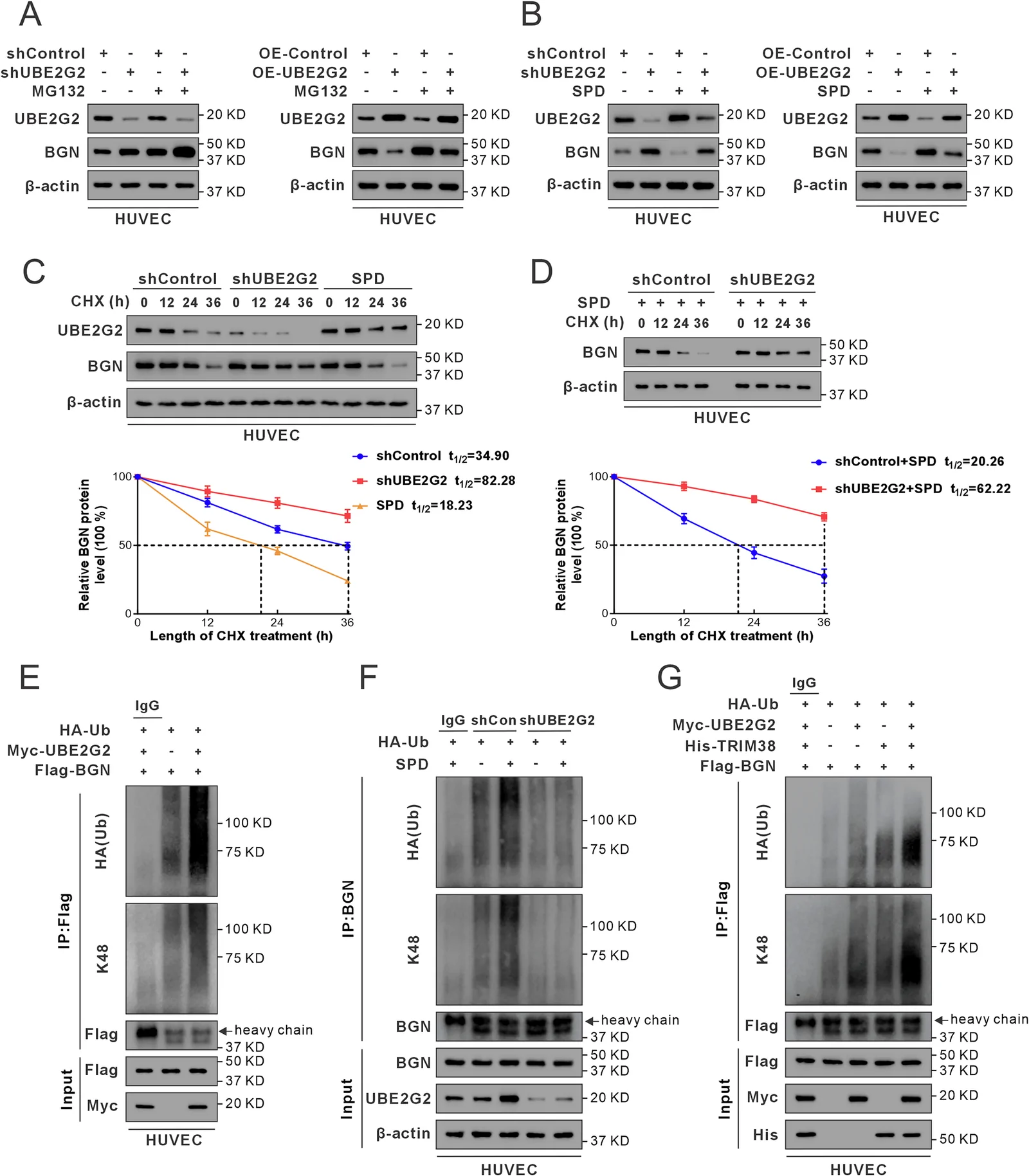
SPD upregulates UBE2G2 to promote the ubiquitination and degradation of BGN. HUVEC were transfected with UBE2G2 (or Control) shRNA lentivirus (left) or adenoviruses AdUBE2G2 (or Control) (right), then cells were treated with MG132 (**A**, 10 μM) or SPD (**B**, 20 μM) and subjected to western blot. **C**, **D** Half-life and quantitative analysis of BGN (*n* = 3, performed in biological replicates). The basal levels of BGN expression at 0 h were adjusted to a similar level for comparison. **C** HUVEC were transfected with shUBE2G2 or treated with SPD, then subjected to 100 μM cycloheximide (CHX) for the indicated time. **D** HUVEC were transfected with shUBE2G2, combined with SPD treatment, then followed by CHX for the indicated time. HUVEC were transfected with HA-tagged ubiquitin (HA-Ub), and BGN ubiquitination was immunoprecipitated using anti-Flag or anti-BGN antibody. Arrow indicates the IgG heavy chain band. All the presented input was adjusted to a similar level for the following assay. **E** HUVEC were transfected with (or without) Myc-UBE2G2 and Flag-BGN. **F** HUVEC were transfected with shUBE2G2 or shControl, then treated with or without SPD (20 μM) for 24 h. **G** HUVEC were co-transfected with Flag-BGN, Myc-UBE2G2 or His-TRIM38.

To elucidate the specific mechanism by which SPD facilitates UBE2G2-mediated degradation of BGN protein, we initially assessed the regulatory influence of UBE2G2 on the expression of protein BGN through the generation of knockdown and overexpression constructs. In HUVEC, UBE2G2 knockdown resulted in a significant increase in the abundance of protein BGN, whereas MG132 effectively prevented the degradation of BGN protein (Fig. 5A, left). Importantly, MG132 was able to counteract the reduction in BGN protein levels induced by UBE2G2 overexpression (Fig. 5A, right), suggesting that UBE2G2 modulates BGN via a proteasome-dependent pathway. Following SPD treatment, there was a notable upregulation of UBE2G2 expression, accompanied by a concomitant decrease in BGN levels (Fig. 5B, right); crucially, UBE2G2 knockdown significantly attenuated the SPD-induced downregulation of BGN (Fig. 5B, left). Collectively, these findings indicate that SPD downregulates the expression of BGN by upregulating UBE2G2 and activating the ubiquitin–proteasome pathway

Subsequently, we assessed the impact of SPD/NRF2/UBE2G2 axis on BGN stability. Protein stability assays indicated that UBE2G2 knockdown significantly extended the half-life of BGN protein, whereas treatment with SPD or UBE2G2 overexpression considerably shortened it (Figs. 5C and S5A). Consistently, UBE2G2 knockdown significantly counteracted the destabilization of BGN protein induced by SPD (Figs. 5D and S5B), suggesting that SPD reduces the stability of BGN protein through the upregulation of UBE2G2. In addition, NRF2 knockdown significantly attenuated SPD-induced UBE2G2 upregulation and subsequent BGN degradation (Fig. S5C), indicating that NRF2 is essential for the SPD-mediated regulation of UBE2G2 expression and BGN degradation. To determine whether this destabilization is attributable to ubiquitin-mediated proteasomal degradation, we examined the ubiquitination of BGN. In HUVEC, UBE2G2 overexpression significantly enhanced both the total ubiquitination and K48-linked polyubiquitination of BGN (Fig. 5E). Furthermore, SPD promoted ubiquitin-dependent degradation of BGN, whereas this effect was markedly attenuated upon UBE2G2 knockdown (Fig. 5F), demonstrating that UBE2G2 is required for the SPD-driven ubiquitination and degradation of BGN.

E2 ubiquitin-conjugating enzymes are known to collaborate with substrate-specific E3 ligases to facilitate K48-linked polyubiquitination. Previous research has demonstrated that the E3 ligase TRIM38 mediates UBE2G2-dependent ubiquitination and subsequent degradation of substrates [26]. In this study, we investigated the cooperative regulation of BGN by UBE2G2 and TRIM38. Co-expression of UBE2G2 and TRIM38 resulted in a greater reduction in BGN protein levels compared to the expression of either UBE2G2 or TRIM38 alone (Fig. S5D). Furthermore, ubiquitination assays revealed that, compared to the sole overexpression, the co-expression of UBE2G2 and TRIM38 significantly enhanced ubiquitination of protein BGN (Fig. 5G). In addition, TRIM38 knockdown/rescue experiments showed that TRIM38 knockdown significantly inhibited SPD/UBE2G2-mediated BGN degradation, while TRIM38 overexpression restored this process (Fig. S5E, F). These results collectively suggest that SPD inhibited BGN expression by upregulating UBE2G2, which, in conjunction with TRIM38, facilitates the ubiquitination-dependent degradation of BGN.

### SPD ameliorates LSECs dysfunction and restrains HSCs activation via UBE2G2-mediated ubiquitination and degradation of BGN

**Fig. 6:**
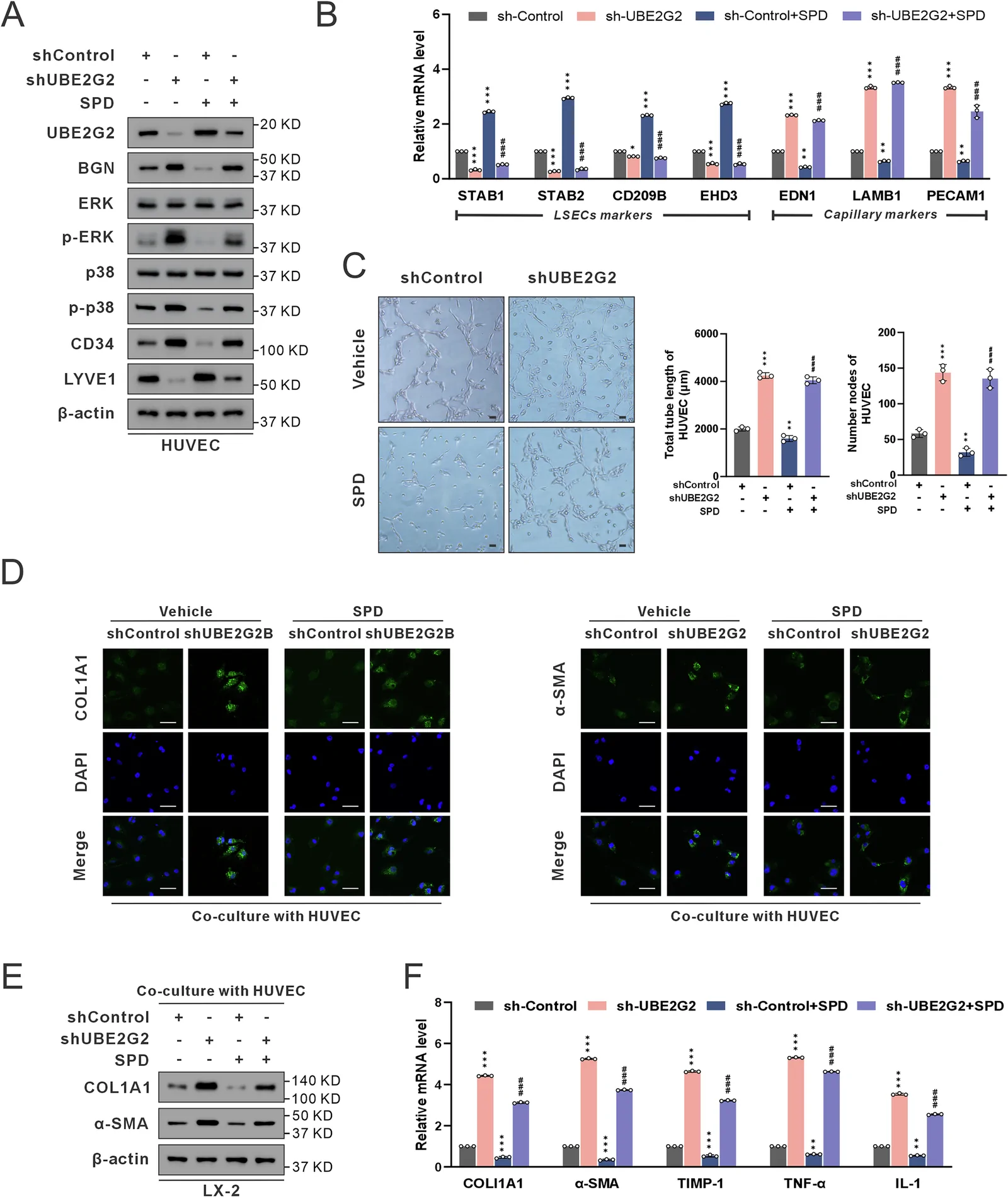
SPD ameliorates LSECs dysfunction and restrains HSC activation via UBE2G2-mediated BGN ubiquitination. HUVEC were transfected with UBE2G2 (or Control) shRNA lentivirus, then treated with SPD (20 μM), and analyzed by western blot (**A**), RT-qPCR (**B**, *n* = 3, performed in biological replicates), and Matrigel tube formation assays (**C**, bar = 50 μM, *n* = 3, performed in biological replicates). LX-2 cells were co-cultured with HUVEC treated as above-mentioned. **D** Immunofluorescence staining of COL1A1 and α-SMA (bar = 50 μM). **E** Analysis of protein levels of COL1A1 and α-SMA. **F** Analysis of mRNA levels of fibrotic markers (*n* = 3, performed in biological replicates). For (**B**, **C**, **F**), * indicated shUBE2G2 or shControl + SPD vs shControl, ^#^ indicated shUBE2G2+ SPD vs shControl + SPD; Data are represented as mean ± SD, *P < 0.05, **P < 0.01, ***P < 0.001; ^#^*P* < 0.05, ^##^*P* < 0.01, ^###^*P* < 0.001.

Building on the evidence that SPD upregulates UBE2G2 to facilitate the ubiquitination and subsequent degradation of protein BGN, we conducted a functional assessment of this pathway to evaluate its impact on the LSECs phenotype and HSCs activation. In HUVEC, UBE2G2 knockdown led to a significant increase in BGN protein levels, accompanied by increased phosphorylation of ERK and p38, and a notable enhancement in LSECs capillarization (Fig. 6A, B). Furthermore, under SPD co-treatment, cells with UBE2G2 knockdown demonstrated an enhanced tube-forming capacity and a greater number of branch points compared to the control group (Figs. 6C and S6A). These results suggest that SPD effectively mitigates LSECs dysfunction by promoting the UBE2G2-dependent ubiquitination and degradation of BGN.

To further evaluate whether this mechanism affects stellate cell activation, we co-cultured LX-2 cells with HUVEC transduced with shControl or shUBE2G2. UBE2G2 knockdown significantly increased the expression of fibrotic and inflammatory markers in co-cultured LX-2 cells and markedly attenuated the ability of SPD to suppress HSCs activation (Fig. 6D–F). Co-culture experiments using primary LSECs and primary HSCs further corroborated these findings (Fig. S6B–E). Collectively, these results indicate that SPD alleviates endothelial dysfunction and thereby inhibits HSCs activation by promoting UBE2G2-dependent ubiquitination and degradation of BGN.

### SPD ameliorates LSECs dysfunction and suppresses hepatic fibrosis through the UBE2G2/BGN axis

**Fig. 7:**
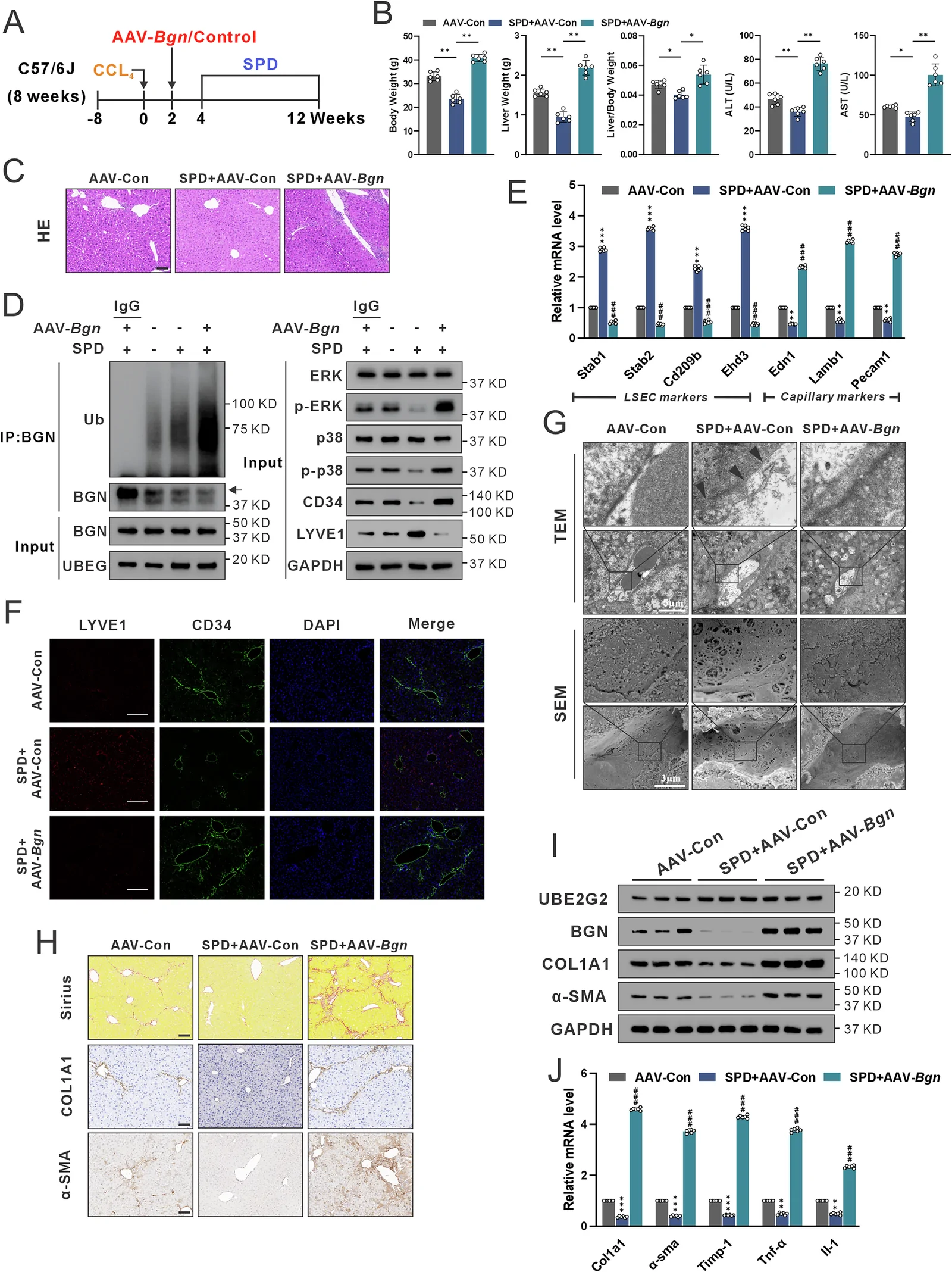
*Bgn* overexpression counteracts the therapeutic effects of SPD in fibrosis. **A** Schematic Diagram of mice treatment. Briefly, mice (*n* = 6) received intraperitoneal injections of CCl₄ and were administered with AAV vectors (AAV-control or AAV-*Bgn*) via the tail vein at 10 weeks of age, followed by an 8-week SPD treatment. **B** Body weight, liver weight, liver/body weight ratio, serum ALT and AST levels in each group. **C** Representative images of H&E staining of liver sections (bar = 100 μM). **D**–**G** Effects of AAV-*Bgn* on LSECs dysfunction. **D** Analysis of BGN ubiquitination, BGN, UBE2G2, ERK/p-ERK, p38/p-p38, CD34, LYVE1 expression in the liver tissues by western blot. The arrow indicates the IgG heavy chain band. **E** Analysis of mRNA levels of LSECs markers and capillary endothelial markers in liver tissues (*n* = 6, performed in biological replicates). **F** Immunofluorescence staining of LYVE1 and CD34 (bar = 100 μM). **G** Representative TEM and SEM images of liver tissues. Effects of AAV-*Bgn* on HSCs activation. **H** Representative images of Sirius Red staining and immunohistochemical staining for COL1A1 and α-SMA in liver sections (bar = 100 μM). **I** Analysis of related protein levels in the livers tissues (*n* = 3, performed in biological replicates). **J** Analysis of mRNA levels of fibrotic markers in liver tissues (*n* = 6, performed in biological replicates). Data are represented as mean ± SD, *P < 0.05, **P < 0.01, ***P < 0.001.

To evaluate whether SPD functions via the UBE2G2/BGN axis in vivo, we employed AAV-mediated overexpression of *Bgn* in conjunction with SPD treatment in a CCl₄-induced liver fibrosis model (Fig. 7A). SPD markedly alleviated liver injury, as evidenced by decreases in liver weight, liver/body weight ratio, and serum ALT/AST levels. Notably, AAV-*Bgn* mostly abrogated these protective effects (Fig. 7B), supported by H&E staining (Fig. 7C). Consistently, western blot analysis demonstrated that AAV-*Bgn* reversed the SPD-induced UBE2G2-mediated downregulation of BGN, reinstating BGN expression and reactivating the ERK/p38 pathway (Fig. 7D). Correspondingly, western blot, qPCR, and immunofluorescence analyses revealed that AAV-*Bgn* counteracted the SPD-mediated inhibition of LSECs capillarization (Fig. 7D–F). TEM and SEM analysis further revealed that the SPD-induced increase in sinusoidal fenestrae was significantly diminished by AAV-*Bgn* (Fig. 7G). Finally, both histological and molecular analysis indicated that *Bgn* overexpression in LSECs markedly abrogated the beneficial effects of SPD, resulting in increased collagen deposition and exacerbated sinusoidal endothelial capillarization, and further aggravating hepatic fibrotic and inflammatory phenotypes (Figs. 7H–J and S7A).

Collectively, these data demonstrate that SPD improves LSECs function and suppresses hepatic fibrosis through the UBE2G2/BGN axis, whereas *Bgn* overexpression significantly diminishes the antifibrotic efficacy of SPD in vivo.

## Discussion

LSECs are liver-specific microvascular endothelial cells, with high trans-sinusoidal permeability due to their dense fenestrae and absence of a continuous basement membrane, making them a critical barrier that maintains hepatic homeostasis [27]. Under chronic liver injury, LSECs undergo a process known as capillarization, characterized by the loss of fenestrations, alterations in their secretome and the formation of a new basement membrane [28]. This phenotypic transformation precedes the onset of fibrosis and remodels the interaction between LSECs and HSCs [28]. Under homeostatic conditions, LSECs sustain HSCs in a quiescent state by continuously producing nitric oxide (NO), which activates the soluble guanylate cyclase (sGC)–cyclic guanosine monophosphate (cGMP) pathway [29]. However, capillarization of LSECs diminished the inhibitory influence on HSCs via the downregulation of endothelial nitric oxide synthase and a consequent reduction in NO bioavailability [6, 30]. Regarding the secretome, homeostatic LSECs secrete hepatocyte growth factor (HGF) and heparin-binding epidermal growth factor-like growth factor (HB-EGF) to inhibit HSCs activation [31–33]. In contrast, during fibrosis, LSECs shift towards producing pro-inflammatory and pro-fibrogenic signals, such as VEGF and Angiopoietin-2 [34, 35]. Concurrently, matrix remodeling leads to the deposition of collagen IV and laminin, which both impair molecular exchange [36]. Thus, LSECs–HSCs communication plays a central role in fibrogenesis, and it is of considerable clinical significance to delineate their intercellular signaling mechanisms. In this study, we used a CCl₄-induced mouse model and an in vitro co-culture system, combined with proteomics, transcriptomics, single-cell omics, a machine learning algorithm and molecular biology assays, to show that SPD ameliorates endothelial dysfunction by downregulating LSECs-derived BGN, thereby suppressing HSCs activation and the progression of hepatic fibrosis.

Utilizing population-based data, we first found that dietary SPD intake is inversely associated with the risk of hepatic fibrosis, with systemic inflammation potentially mediating part of this association. This observation aligns with previous epidemiological studies linking higher SPD intake to decreased all-cause mortality and cardiovascular risk [10], suggesting a systemic protective effect of SPD potentially mediated through anti-inflammatory pathways [37]. Subsequently, we employed network-pharmacology analyses to explore the molecular interactions between SPD intervention and LSECs dysfunction. The overlap of predicted SPD targets with genes related to liver fibrosis was significantly enriched in angiogenesis and focal adhesion pathways, indicating that SPD may exert its antifibrotic effects primarily by modulating the intrahepatic vascular microenvironment. Notably, this is consistent with the current understanding that LSECs dysfunction often precedes fibrosis and influences fibrotic susceptibility [38]. Consequently, we hypothesize that the antifibrotic activity of SPD is initiated by its effects on the LSECs phenotype; in other words, SPD may reshape the functional state and secretome of LSECs to blunt the profibrogenic cues required for HSCs activation, thereby restraining fibrogenesis.

To further explore the targets of SPD, we employed an integrative approach combining proteomics, publicly available single-cell datasets of LSECs, and machine-learning methodologies. This comprehensive analysis led to the identification of BGN as a significant regulator of endothelial function and fibrogenesis. BGN, a small leucine-rich proteoglycan, plays dual roles as both a structural component of the ECM and a signaling modulator in vascular homeostasis and inflammation [39–41]. In non-hepatic vascular environments, BGN facilitates endothelial cell migration and angiogenesis through autocrine mechanisms and enhances pro-angiogenic signaling by binding or upregulating VEGFA [41]. Under inflammatory conditions, BGN acts as a damage-associated molecular pattern (DAMP), activating the TLR2/4–MAPK–NF-κB pathways, inducing TNF-α and other cytokines, and promoting ECM deposition [42]. Clinically, numerous studies have reported that serum or tissue levels of BGN increase with the progression of fibrosis, serving as a potential biomarker for distinguishing significant fibrosis and monitoring disease progression or therapeutic response [43, 44]. Consistent with these observations, our experiments showed that SPD markedly downregulated BGN and reduced ECM accumulation in the CCl₄-induced mice fibrosis model; Meanwhile, SPD similarly lowered BGN levels and improved LSECs function by attenuating ERK/p38 phosphorylation in vitro. Together, these data suggest that LSECs-derived BGN exerts a dual function—promoting LSECs capillarization while simultaneously activating HSCs, thereby driving hepatic fibrogenesis. This positions BGN as a viable target for antifibrotic therapy. Nevertheless, additional single-cell datasets will be valuable to validate and refine the regulatory mechanisms governing BGN expression in endothelial cells.

Interestingly, while SPD significantly diminished the abundance of BGN protein, the levels of its mRNA remained unchanged, indicating regulation at a post-transcriptional level. Transcriptomic analysis further demonstrated a notable enrichment of DEGs within pathways related to endoplasmic-reticulum protein folding and degradation. Additionally, WGCNA also indicated that the BGN-containing module was closely associated with ubiquitination. In conjunction with MG132 experiments, these findings suggest that SPD facilitates BGN degradation through the ubiquitin–proteasome pathway. Subsequent prioritization guided by WGCNA, along with overexpression assays, pinpointed the E2 ubiquitin-conjugating enzyme UBE2G2 as a critical factor influencing BGN stability. Previous research showed that UBE2G2 participates in ER-associated degradation [45], in collaboration with the E3 ligase TRIM38, mediated K48-linked ubiquitination and degradation of LGALS3BP to negatively regulate angiogenesis [26, 46]. However, its function in liver fibrosis remains largely unknown. In this study, we demonstrate that SPD activates NRF2 to upregulate UBE2G2, thereby enhancing UBE2G2-dependent ubiquitination and degradation of BGN, which in turn ameliorates LSECs dysfunction and attenuates HSCs activation. Distinct from prior work focused on transcriptional control of BGN, our findings, for the first time, uncover a ubiquitin-dependent regulatory mechanism for BGN and provide a new perspective for targeting this fibrotic mediator.

Overall, this study reveals for the first time that LSECs-derived BGN is a key secreted mediator linking endothelial dysfunction to HSCs activation and demonstrates that SPD exerts antifibrotic effects through downregulation of BGN. Mechanistically, SPD activates the NRF2–UBE2G2 axis to enhance the ubiquitination and subsequent proteasomal degradation of LSEC-derived BGN. The ensuing decrease in BGN levels mitigates LSECs capillarization and inhibits HSCs activation. Thus, this study highlights the significance of SPD in maintaining the physiological phenotype of LSECs and proposes that targeting the UBE2G2–BGN axis as a potential therapeutic strategy to impede the progression of hepatic fibrosis.

Although the effects of SPD on LSECs–HSCs communication have been elucidated specifically, this study still has several limitations. Firstly, we did not use LSEC-specific deletion models for UBE2G2 or BGN, which limits our ability to define the contribution of the SPD-NRF2-UBE2G2-BGN axis to the antifibrotic effects in LSECs. Secondly, the NHANES analysis of dietary spermidine and fibrosis risk is observational and cannot prove causality. Despite adjusting for confounders, residual confounding and reverse causation may still be present. Future prospective cohort studies with circulating spermidine measurements and longitudinal fibrosis follow-up are needed for stronger causal evidence and to validate the proposed mechanism.

## Conclusions

Our findings uncover a novel mechanism whereby SPD ameliorates LSECs dysfunction and suppresses fibrosis progression by modulating LSECs-derived BGN, suggesting a new therapeutic strategy for liver fibrosis.

## Materials and methods

### Data source and clinical analysis

Dietary and clinical data were obtained from the publicly available National Health and Nutrition Examination Survey (NHANES, 2003–2023; https://wwwn.cdc.gov/nchs/nhanes/) and the Food Patterns Equivalents Database (FPED; https://www.ars.usda.gov/…/fped-databases/). Spermidine intake was calculated by integrating previously published spermidine content in food groups with the FPED food consumption data [47].

All participants provided written informed consent prior to participation. The NHANES protocols were approved by the National Center for Health Statistics Research Ethics Review Board (CDC, Atlanta, GA, USA).

Liver fibrosis was assessed using transient elastography (liver stiffness measurement, LSM) and the Fibrosis-4 index (FIB-4), a widely accepted non-invasive surrogate marker for liver fibrosis. Fibrosis was defined as either LSM > 7.0 kPa or FIB-4 index > 2.67 [48, 49].

### Animal model and experimental design

A total of 24 male C57BL/6 mice (6 weeks old) were purchased from Huachuang Xinnuo Pharmaceutical Technology Co., Ltd. (Jiangsu, China) (License No. SCXK [Su] 2022-00009) and housed under specific pathogen-free conditions at the Animal Centre of the Second Affiliated Hospital of Chongqing Medical University. The mice were maintained on a 12 h light/dark cycle at a constant temperature (20–24 °C) and humidity (40–70%), with ad libitum access to standard chow and water. All feeding, management, cleaning, sampling, and handling procedures were performed in accordance with the relevant regulations of the Ethics Committee of Chongqing Medical University.

After a 2-week acclimatization period, the mice were randomly assigned to four groups (*n* = 6 per group): Control (Con), CCl₄, CCl₄ + Spermidine (SPD, Sigma-Aldrich, S0266), and CCl₄ + Obeticholic Acid (OCA; MedChemExpress, HY-12222). No blinding was performed during the experiment. Except for the Con group, all mice received intraperitoneal injections of 20% CCl₄ in corn oil (2 mL/kg) twice a week for 8 weeks. The Con group received an equivalent volume of corn oil. After being injected with 20% CCl₄ for one week, the SPD group was provided with drinking water containing 3 mM SPD (refreshed every 3 days), while the OCA group was administered obeticholic acid by oral gavage at 5 mg/kg/day. At the end of the 8-week experimental period, mice were euthanized, and serum and liver tissue samples were collected and stored at −80 °C for subsequent analyses.

### Mouse AAV8 construction and injection

Recombinant adeno-associated virus serotype 8 (AAV8) vectors encoding murine biglycan (AAV8-Tie2-*Bgn*) were generated by OBiO Technology (Shanghai) Corp., Ltd. The AAV8-Tie2-Control vector served as the control. Transgene expression was regulated by the endothelial-specific Tie2 promoter. Vector genome (vg) titers were quantified by the manufacturer using quantitative PCR with vector-specific primers. Ten-week-old male C57BL/6J mice received intravenous tail-vein injections of AAV at a dosage of 2 × 10^12^ vg per mouse, with each mouse receiving 100 μL.

### Cell culture and processing

LX-2 cells were purchased from Ubigene (Guangzhou, China). Human umbilical vein endothelial cells (HUVEC) were kindly provided by Dr. Hengqiu He (Department of Oncology, the Second Affiliated Hospital of Chongqing Medical University). LX-2 cells were cultured in Dulbecco’s Modified Eagle Medium (DMEM; SH30243.01, HyClone, Logan, UT, USA) supplemented with 10% fetal bovine serum (Natocor, Argentina), 100 U/mL penicillin, and 100 μg/mL streptomycin (SV30010, HyClone). HUVEC were maintained in endothelial cell medium (Immocell, IMC-309). All cells were incubated at 37 °C in a humidified atmosphere containing 5% CO₂.

For LX-2 cell activation and HUVEC dysfunction, cells were serum-starved in 0.1% FBS for 12 h, followed by stimulation with 10 ng/mL recombinant human TGF-β1 for 24 h and 48 h, respectively.

For co-culture experiments, LX-2 cells or primary HSCs were plated in 12-well plates. HUVEC or primary LSECs were seeded onto polyester transwell inserts (0.4-μm pores; BIOFIL, Guangzhou, China) and allowed to adhere overnight. Inserts were then placed into the LX-2 or HSCs-containing wells and co-cultured for 48 h in DMEM with 2% FBS. After co-culture, the Transwell inserts were removed, the medium in the lower chamber was aspirated, and the cells were gently washed twice with PBS before immediate collection for analyses.

### Immunoprecipitation (IP) assay

Cells were transfected with HA-Ub, or co-transfected with HA-Ub, Flag-BGN, and Myc-UBE2G2, or together with His-TRIM38. To prevent degradation, 10 μM MG132 (T2154, TargetMol, USA) was added before harvesting. Cells were lysed with 1% SDS buffer and boiled for denaturation after 48 h post-transfection. Immunoprecipitation was performed using anti-Flag or anti-BGN antibodies, followed by incubation with protein A/G agarose beads for 4 h. Beads-bound complexes were washed four times and subjected to a Western blot. Ubiquitination levels of BGN were assessed with anti-HA and anti-K48 antibodies. All the presented input was adjusted to a similar level for the following assay.

### Tube formation assay

HUVEC and primary LSECs were first cultured in 60-mm dishes and transfected with the indicated shRNA or overexpression plasmids. 24 h later, the cells were seeded onto 48-well plates precoated with Matrigel (50 μL/well; Corning, USA) and incubated in medium containing vascular endothelial growth factor (VEGF)-A (25 ng/mL; Miltenyi Biotech, Germany; #130-092-007) at 37 °C in a humidified atmosphere with 5% CO₂. After 24 h, tube-like structures were examined and photographed using an inverted microscope (Nikon ECLIPSE Ts2 equipped with a Nikon DS-Fi2 camera; Nikon, USA). The number and total length of tubular structures were quantified using ImageJ software (NIH, USA).

### Transmission electron microscopy (TEM) and scanning electron microscopy (SEM)

Liver samples were fixed in glutaraldehyde, followed by postfixation in osmium tetroxide. The tissues were dehydrated through a graded ethanol series. For TEM, tissues were embedded in Epon resin, ultrathin-sectioned (60–80 nm), stained with uranyl acetate and lead citrate, and imaged at 80–120 kV. For SEM, dehydrated specimens were dried by CO₂ critical-point drying (with hexamethyldisilazane as a validated low-cost alternative), mounted with the sinusoidal surface facing up, sputter-coated with 5–10 nm Au/Pd, and imaged at low accelerating voltage (1–5 kV). Samples were then examined with an electron microscope (Zeiss, Germany). The porosity of LSECs, defined as the percentage of the surface area occupied by fenestrae, was determined from SEM images.

### Histological analysis

Liver tissues were fixed in 4% paraformaldehyde (E672002, Sangon Biotech) at 4 °C for 24 h, embedded in paraffin, and sectioned at a thickness of 5 μm. The sections were deparaffinized in xylene and rehydrated through a graded ethanol series, followed by hematoxylin–eosin (H&E), Sirius Red staining and other indicated antibodies. Images were acquired using a digital pathology slide scanner.

### Proteomics analysis using DIA mass spectrometry

Proteomics analysis was performed at Majorbio Bio-pharm Biotechnology Co., Ltd. (Shanghai, China). Proteins were extracted from liver tissue using lysis buffer containing urea, SDS, and protease inhibitors. After quantification, proteins were digested with trypsin, and peptides were desalted, dried, and re-dissolved for analysis.

Peptide samples were analyzed on an Orbitrap Astral mass spectrometer (Thermo, USA) in DIA mode at Majorbio Bio-pharm Technology Co., Ltd. (Shanghai, China). Raw DIA data were processed using Spectronaut software (v19) with a false discovery rate (FDR) < 1% at both peptide and protein levels. Protein quantification was performed using the MaxLFQ algorithm.

Differentially expressed proteins (DEPs) were defined by fold change >1.2 or <0.83 and *P* < 0.05. Functional enrichment analyses of DEPs, including Gene Ontology (GO) and Kyoto Encyclopedia of Genes and Genomes (KEGG) pathways, were conducted, and protein–protein interaction analysis was performed using STRING (v11.5).

### Transcriptomics analysis using RNA-seq

RNA-seq was performed at Majorbio Bio-pharm Biotechnology Co., Ltd. (Shanghai, China). Briefly, total RNA was extracted from liver tissue using TRIzol reagent (Invitrogen, CA, USA) following the manufacturer’s instructions. RNA quality was assessed using the 5300 Bioanalyser (Agilent, USA) and NanoDrop ND-2000 (Thermo Fisher Scientific, USA). High-quality RNA samples (OD260/280 = 1.8–2.2, OD260/230 ≥ 2.0, RQN ≥ 6.5, 28S:18S ≥ 1.0, >1 μg) were used for library preparation.

RNA-seq libraries were constructed using the Illumina® Stranded mRNA Prep, Ligation Kit (San Diego, CA, USA) following the manufacturer’s protocol and sequenced on the Illumina NovaSeq X Plus platform (PE150) or DNBSEQ-T7 platform (PE150, MGI Tech, China) at Shanghai Majorbio Bio-pharm Biotechnology Co., Ltd. (Shanghai, China).

Raw reads were processed with fastp, aligned to the reference genome using HISAT2, and assembled by StringTie. Gene abundances were quantified with RSEM and normalized as transcripts per million reads (TPM). Differential expression analysis was performed using DESeq2 and DEGseq, with DEGs defined as |FC | ≥ 1.5 and FDR < 0.05 (DESeq2) or FDR < 0.001 (DEGseq).

Functional enrichment analysis of DEGs was conducted using GOATOOLS (GO) and scipy (KEGG), with Bonferroni correction (*P* < 0.05). Data visualization was performed in R (v4.4.1).

### Screening of core targets using machine learning algorithms

To identify the core targets involved in SPD-mediated improvement of LSECs dysfunction, this study integrated machine learning algorithms with single-cell transcriptomics and proteomics data. Four machine learning methods were employed: LASSO, NNET, RF, and SVM. LASSO analysis was performed using the “glmnet” package in R, while NNET analysis was carried out using the “nnet” package. Random Forest (RF) analysis was conducted with the “randomForest” package, and SVM analysis was implemented using the “e1071” package. The top ten genes identified by each of these algorithms were subjected to Venn diagram analysis to identify overlapping genes. These overlapping genes were defined as the core targets associated with LSECs dysfunction. Furthermore, these core targets were cross-referenced with proteomics data to refine the final set of SPD-mediated core targets involved in hepatic sinusoidal endothelial cell dysfunction.

### Isolation of primary LSECs and HSCs

Primary HSCs and LSECs were isolated from mice using collagenase perfusion combined with density gradient centrifugation, as previously described [7]. Briefly, anesthetized mice were subjected to in situ portal vein perfusion with 30 mL Hank’s balanced salt solution containing 0.5 mmol/L ethylenediaminetetraacetic acid, followed by perfusion with 30 mL of 0.05% collagenase IV (Sigma-Aldrich, C4-BIOC) solution. The liver was excised, minced, and further digested with 0.025% collagenase IV at 37 °C for 30 min to obtain a single-cell suspension.

The digested suspension was centrifuged at 50 × *g* for 4 min to remove undigested tissue fragments. The supernatant was then centrifuged at 500 × *g* for 8 min at 4 °C, and the resulting pellet was resuspended and passed through a 40 μm cell strainer to obtain a suspension containing non-parenchymal cells (NPCs).

The NPC suspension was centrifuged at 25 × *g* to remove residual hepatocytes. The supernatant was subsequently centrifuged at 300 × *g* to collect NPCs, which were resuspended in culture medium and layered onto the top of a two-step Percoll gradient (50% lower layer, 25% upper layer). The gradient was centrifuged at 900 × *g* for 25 min. Cells located at the interface between the 25% and 50% Percoll layers were primarily LSECs, whereas cells in the upper 25% Percoll layer were enriched in HSCs. Both fractions were collected, washed with PBS, and used for subsequent experiments.

### Ethics statement

The animal experiments were approved by the Ethics Committee on Use and Care of Animals of the Second Affiliated Hospital, Chongqing Medical University, Chongqing, China (Approval No. IACUC-SAHCQMU-2025-0142).

### Statistical analysis

All research data were analysed and plotted using R software (version 4.4.1) and GraphPad Prism 10.1.2. Data consist of means ± SD of the mean from three independent biological replicates. Student’s *t* test or paired t-test was used for the comparison of two groups. One-way ANOVA followed by Tukey’s test was used for all other variables. Pearson’s correlation coefficient (R) was used for the comparison of linear correlations. *P*-value < 0.05 was regarded as significant. *P < 0.05, **P < 0.01, ***P < 0.001.

## Supplementary information

Supplementary information

Full and uncropped western blots

## Data Availability

Data will be made available on request.

## References

[1] Hammerich L, Tacke F. Hepatic inflammatory responses in liver fibrosis. Nat Rev Gastroenterol Hepatol. 2023;20:633–46. 10.1038/s41575-023-00807-x 37400694

[2] Parola M, Pinzani M. Liver fibrosis in NAFLD/NASH: from pathophysiology towards diagnostic and therapeutic strategies. Mol Asp Med. 2024;95:101231. 10.1016/j.mam.2023.101231 38056058

[3] Bourebaba N, Marycz K. Hepatic stellate cells role in the course of metabolic disorders development—A molecular overview. Pharm Res. 2021;170:105739. 10.1016/j.phrs.2021.105739 34171492

[4] Tsuchida T, Friedman SL. Mechanisms of hepatic stellate cell activation. Nat Rev Gastroenterol Hepatol. 2017;14:397–411. 10.1038/nrgastro.2017.38 28487545

[5] Hammoutene A, Rautou P-E. Role of liver sinusoidal endothelial cells in non-alcoholic fatty liver disease. J Hepatol. 2019;70:1278–91. 10.1016/j.jhep.2019.02.012 30797053

[6] McConnell MJ, Kostallari E, Ibrahim SH, Iwakiri Y. The evolving role of liver sinusoidal endothelial cells in liver health and disease. Hepatology. 2023;78:649–69. 10.1097/HEP.0000000000000207 PMC10315420 36626620

[7] Duan J-L, Ruan B, Yan X-C, Liang L, Song P, Yang Z-Y, et al. Endothelial Notch activation reshapes the angiocrine of sinusoidal endothelia to aggravate liver fibrosis and blunt regeneration in mice. Hepatology. 2018;68:677–90. 10.1002/hep.29834 PMC6099357 29420858

[8] Hofer SJ, Liang Y, Zimmermann A, Schroeder S, Dengjel J, Kroemer G, et al. Spermidine-induced hypusination preserves mitochondrial and cognitive function during aging. Autophagy. 2021;17:2037–9. 10.1080/15548627.2021.1933299 PMC8386697 34105442

[9] Chamoto K, Zhang B, Tajima M, Honjo T, Fagarasan S. Spermidine—an old molecule with a new age-defying immune function. Trends Cell Biol. 2024;34:363–70. 10.1016/j.tcb.2023.08.002 37723019

[10] Madeo F, Eisenberg T, Pietrocola F, Kroemer G. Spermidine in health and disease. Science. 2018;359:eaan2788. 10.1126/science.aan2788 29371440

[11] Han S, Qian M, Zhang N, Zhang R, Liu M, Wang J, et al. The Association of dietary polyamines with mortality and the risk of cardiovascular disease: a prospective study in the UK Biobank. Nutrients. 2024;16:4335. 10.3390/nu16244335 PMC11678356 39770955

[12] Liu P, de la Vega MR, Dodson M, Yue F, Shi B, Fang D, et al. Spermidine confers liver protection by enhancing Nrf2 signaling through a MAP1S-mediated noncanonical mechanism. Hepatology. 2019;70:372–88. 10.1002/hep.30616 PMC6597327 30873635

[13] Zhou J, Pang J, Tripathi M, Ho JP, Widjaja AA, Shekeran SG, et al. Spermidine-mediated hypusination of translation factor EIF5A improves mitochondrial fatty acid oxidation and prevents non-alcoholic steatohepatitis progression. Nat Commun. 2022;13:5202. 10.1038/s41467-022-32788-x PMC9440896 36057633

[14] Liu R, Cao L, Zhou Y, Li Y, Wang T, Meng Z, et al. Spermidine enhances macrophages anti-inflammatory and regenerative functions by improving mitochondrial fitness. Phytomedicine. 2025;148:157349. 10.1016/j.phymed.2025.157349 41067199

[15] Cong L, Maishi N, Annan DA, Young MF, Morimoto H, Morimoto M, et al. Inhibition of stromal biglycan promotes normalization of the tumor microenvironment and enhances chemotherapeutic efficacy. Breast Cancer Res. 2021;23:51. 10.1186/s13058-021-01423-w PMC8108358 33966638

[16] Grandoch M, Kohlmorgen C, Melchior-Becker A, Feldmann K, Homann S, Müller J, et al. Loss of Biglycan enhances thrombin generation in apolipoprotein E-deficient mice: implications for inflammation and atherosclerosis. Arterioscler Thromb Vasc Biol. 2016;36:e41–50. 10.1161/ATVBAHA.115.306973 27034473

[17] Yamamoto K, Ohga N, Hida Y, Maishi N, Kawamoto T, Kitayama K, et al. Biglycan is a specific marker and an autocrine angiogenic factor of tumour endothelial cells. Br J Cancer. 2012;106:1214–23. 10.1038/bjc.2012.59 PMC3304426 22374465

[18] Liu M, Zhao P, Feng H, Yang Y, Zhang X, Chen E, et al. Biglycan stimulates retinal pathological angiogenesis via up-regulation of CXCL12 expression in pericytes. FASEB J. 2025;39:e70262. 10.1096/fj.202401903R PMC11701870 39760177

[19] Yu M, Si C, Xinjue H, Pan Y, Dai Y, Jin C, et al. Biglycan deficiency alleviates intestinal fibrosis through BMP-7-mediated Smad1/5/8 signaling. J Crohns Colitis. 2025;19:jjaf065. 10.1093/ecco-jcc/jjaf065 40249230

[20] Yu M, He X, Song X, Gao J, Pan J, Zhou T, et al. Biglycan promotes hepatic fibrosis through activating heat shock protein 47. Liver Int. 2023;43:500–12. 10.1111/liv.15477 36371672

[21] de Haan W, Dheedene W, Apelt K, Décombas-Deschamps S, Vinckier S, Verhulst S, et al. Endothelial Zeb2 preserves the hepatic angioarchitecture and protects against liver fibrosis. Cardiovasc Res. 2022;118:1262–75. 10.1093/cvr/cvab148 PMC8953454 33909875

[22] Nulan Y, Felli E, Selicean S-E, Prampolini M, Berzigotti A, Gracia-Sancho J, et al. Carvedilol decreases hepatic vascular resistance by reducing fibrogenesis and reversing endothelial dysfunction in cirrhotic rats. JHEP Rep. 2026;8:101681. 10.1016/j.jhepr.2025.101681 PMC12878606 41659769

[23] Gubbiotti MA, Buraschi S, Kapoor A, Iozzo RV. Proteoglycan signaling in tumor angiogenesis and endothelial cell autophagy. Semin Cancer Biol. 2020;62:1–8. 10.1016/j.semcancer.2019.05.003 PMC7864242 31078640

[24] Wadgaonkar P, Bi Z, Wan J, Fu Y, Zhang Q, Almutairy B, et al. Arsenic activates the ER stress-associated unfolded protein response via the activating transcription factor 6 in human bronchial epithelial cells. Biomedicines. 2022;10:967. 10.3390/biomedicines10050967 PMC9139116 35625704

[25] Yang Z, Tai Y, Lan T, Zhao C, Gao J-H, Tang C-W, et al. Inhibition of cyclooxygenase-2 upregulates the nuclear factor erythroid 2-related factor 2 signaling pathway to mitigate hepatocyte ferroptosis in chronic liver injury. J Clin Transl Hepatol. 2025;13:409–17. 10.14218/JCTH.2024.00440 PMC12078174 40385941

[26] Zhao A, Zhou C, Li J, Wang Z, Zhu H, Shen S, et al. UBE2G2 inhibits vasculogenic mimicry and metastasis of uveal melanoma by promoting ubiquitination of LGALS3BP. Acta Pharm Sin B. 2024;14:5201–18. 10.1016/j.apsb.2024.09.005 PMC11725101 39807310

[27] Gracia-Sancho J, Caparrós E, Fernández-Iglesias A, Francés R. Role of liver sinusoidal endothelial cells in liver diseases. Nat Rev Gastroenterol Hepatol. 2021;18:411–31. 10.1038/s41575-020-00411-3 33589830

[28] Qu J, Wang L, Li Y, Li X. Liver sinusoidal endothelial cell: an important yet often overlooked player in liver fibrosis. Clin Mol Hepatol. 2024;30:303–25. 10.3350/cmh.2024.0022 PMC11261236 38414375

[29] Chen T, Shi Z, Zhao Y, Meng X, Zhao S, Zheng L, et al. LncRNA Airn maintains LSEC differentiation to alleviate liver fibrosis via the KLF2-eNOS-sGC pathway. BMC Med. 2022;20:335. 10.1186/s12916-022-02523-w PMC9520944 36171606

[30] Marrone G, Shah VH, Gracia-Sancho J. Sinusoidal communication in liver fibrosis and regeneration. J Hepatol. 2016;65:608–17. 10.1016/j.jhep.2016.04.018 PMC4992446 27151183

[31] Lao Y, Li Y, Zhang P, Shao Q, Lin W, Qiu B, et al. Targeting endothelial Erk1/2-Akt axis as a regeneration strategy to bypass fibrosis during chronic liver injury in mice. Mol Ther. 2018;26:2779–97. 10.1016/j.ymthe.2018.08.016 PMC6277559 30266653

[32] Zhang X-J, Olsavszky V, Yin Y, Wang B, Engleitner T, Öllinger R, et al. Angiocrine hepatocyte growth factor signaling controls physiological organ and body size and dynamic hepatocyte proliferation to prevent liver damage during regeneration. Am J Pathol. 2020;190:358–71. 10.1016/j.ajpath.2019.10.009 31783007

[33] Maretti-Mira AC, Wang X, Wang L, DeLeve LD. Incomplete differentiation of engrafted bone marrow endothelial progenitor cells initiates hepatic fibrosis in the rat. Hepatology. 2019;69:1259–72. 10.1002/hep.30227 PMC6387651 30141211

[34] Ding B-S, Nolan DJ, Butler JM, James D, Babazadeh AO, Rosenwaks Z, et al. Inductive angiocrine signals from sinusoidal endothelium are required for liver regeneration. Nature. 2010;468:310–5. 10.1038/nature09493 PMC3058628 21068842

[35] Wang Y, Wang C, Yang F, Chen Y, Shi Y, Xu R, et al. USP9X-enriched MSC-sEV inhibits LSEC angiogenesis in MASH mice by downregulating the IκBα/NF-κB/Ang-2 pathway. Pharm Res. 2024;209:107471. 10.1016/j.phrs.2024.107471 39427871

[36] Li G, Lin J, Peng Y, Qin K, Wen L, Zhao T, et al. Curcumol may reverse early and advanced liver fibrogenesis through downregulating the uPA/uPAR pathway. Phytother Res. 2020;34:1421–35. 10.1002/ptr.6616 31989700

[37] Pham CN, Castelli F, Finet F, Leroy C, Chollet C, Chirayath TW, et al. Spermidine reproduces the anti-inflammatory effects of intermittent fasting and prevents urate and calcium pyrophosphate crystal-induced inflammation. Arthritis Rheumatol. 2026;78:449–62. 10.1002/art.43367 PMC12936900 40884015

[38] DeLeve LD. Liver sinusoidal endothelial cells in hepatic fibrosis. Hepatology. 2015;61:1740–6. 10.1002/hep.27376 PMC4333127 25131509

[39] Han CY, Kang I, Harten IA, Gebe JA, Chan CK, Omer M, et al. Adipocyte-derived versican and macrophage-derived biglycan control adipose tissue inflammation in obesity. Cell Rep. 2020;31:107818. 10.1016/j.celrep.2020.107818 PMC7384517 32610121

[40] Wight TN. A role for proteoglycans in vascular disease. Matrix Biol. 2018;71–72:396–420. 10.1016/j.matbio.2018.02.019 PMC6110991 29499356

[41] Hu L, Zang M, Wang H-X, Li J-F, Su L-P, Yan M, et al. Biglycan stimulates VEGF expression in endothelial cells by activating the TLR signaling pathway. Mol Oncol. 2016;10:1473–84. 10.1016/j.molonc.2016.08.002 PMC5423211 27590684

[42] Roedig H, Damiescu R, Zeng-Brouwers J, Kutija I, Trebicka J, Wygrecka M, et al. Danger matrix molecules orchestrate CD14/CD44 signaling in cancer development. Semin Cancer Biol. 2020;62:31–47. 10.1016/j.semcancer.2019.07.026 31412297

[43] Ciftciler R, Ozenirler S, Yucel AA, Cengiz M, Erkan G, Buyukdemirci E, et al. The importance of serum biglycan levels as a fibrosis marker in patients with chronic hepatitis B. J Clin Lab Anal. 2017;31:e22109. 10.1002/jcla.22109 PMC6817276 27925300

[44] Cengiz M, Yilmaz G, Ozenirler S. Serum Biglycan as a diagnostic marker for non-alcoholic steatohepatitis and liver fibrosis. Clin Lab. 2021;67. 10.7754/Clin.Lab.2020.200709. 33739030

[45] Chakrabarti KS, Li J, Das R, Byrd RA. Conformational dynamics and allostery in E2:E3 interactions drive ubiquitination: gp78 and Ube2g2. Structure. 2017;25:794–805.e5. 10.1016/j.str.2017.03.016 PMC5512444 28434917

[46] Liu W, Shang Y, Zeng Y, Liu C, Li Y, Zhai L, et al. Dimeric Ube2g2 simultaneously engages donor and acceptor ubiquitins to form Lys48-linked ubiquitin chains. EMBO J. 2014;33:46–61. 10.1002/embj.201385315 PMC3990682 24366945

[47] Ma Y, Qi L, Zhu F, Xiao M, Li M, Zhang X, et al. Association between dietary spermidine intake and cognitive performance in older adults: The U.S. National Health and Nutrition Examination Survey, 2011-2014. J Affect Disord. 2025;381:174–82. 10.1016/j.jad.2025.03.181 40180050

[48] Kim HY, Yu JH, Chon YE, Kim SU, Kim MN, Han JW, et al. Prevalence of clinically significant liver fibrosis in the general population: a systematic review and meta-analysis. Clin Mol Hepatol. 2024;30:S199–213. 10.3350/cmh.2024.0351 PMC11493351 39074982

[49] Duarte-Rojo A, Taouli B, Leung DH, Levine D, Nayfeh T, Hasan B, et al. Imaging-based noninvasive liver disease assessment for staging liver fibrosis in chronic liver disease: a systematic review supporting the AASLD Practice Guideline. Hepatology. 2025;81:725–48. 10.1097/HEP.0000000000000852 38489521

