# Supplementary material for: Spermidine suppresses liver fibrosis by remodeling the communication signal between liver sinusoidal endothelial cells and hepatic stellate cells
Source: Cell Death Discov. 2026 May 7;12:287. doi: 10.1038/s41420-026-03129-4 (PMC13320189; doi:10.1038/s41420-026-03129-4)
Supplement: Supplementary file 1 — Supplementary information [file 41420_2026_3129_MOESM1_ESM.docx]

**Supplementary Information**

**Spermidine Suppresses Liver Fibrosis by Remodeling the Communication Between Liver Sinusoidal Endothelial Cells and Hepatic Stellate Cells**

Cheng Zeng ^1†^, Jiao Liu ^1†^, Zhiqiang Jin ^1†^, Shan Zhong ^1^, Rong Wu ^3^, Haoyue Luo ^1^, Tao Zeng ^4^, Yang Yang ^2,1*^, and Zhi Zhou ^1*^

^1^Department of Infectious Diseases, Key Laboratory of Molecular Biology for Infectious Diseases (Ministry of Education), Institute for Viral Hepatitis, the Second Affiliated Hospital, Chongqing Medical University, Chongqing 401336, China.

^2^Department of Liver Diseases, Chongqing Traditional Chinese Medicine Hospital, Chongqing 400021, China.

^3^Department of Gastroenterology and Hepatology, The Second Affiliated Hospital of Chongqing Medical University, Chongqing, 400010, China.

^4^Department of Ultrasound, Chengdu Women’s and Children’s Central Hospital, School of Medicine, University of Electronic Science and Technology of China, Chengdu, China.

†These authors contributed equally to this work.

Correspondence to: Zhi Zhou ([zhouzhi2300@hospital.cqmu.edu.cn](mailto:zhouzhi2300@hospital.cqmu.edu.cn)) and

Yang Yang (yangyangxd95@163.com).

**This PDF file includes:**

Supplementary Materials and Methods

Supplementary Figure and Tables

**Supplementary Materials and Methods**

**Gene Knockdown and Overexpression**

For gene knockdown, shRNA targeting BGN or UBE2G2 was designed and cloned into pLL3.7 lentivirus vector by Beijing Tsingke Biotechnology Co., Ltd. (Beijing, China). A negative control construct (shControl) was also generated. the lentiviral vectors were co-transfected with psPAX2 and pMD2.G into 293T cells by Lipofectamine 3000. At 48 h after transfection, supernatants containing viruses were collected and filtered. Then cells were infected with lentiviruses with 5 μg/mL polybrene. In addition, the siRNA targeting NRF2 was also designed and constructed by Beijing Tsingke Biotechnology Co., Ltd.

For gene overexpression, the adenoviral recombinant AdBGN or AdUBE2G2 were generated using the AdEasy system by Beijing Tsingke Biotechnology Co., Ltd. A negative control construct (AdControl) was also generated. In addition, the recombinant plasmid pcDNA3.1-Flag-BGN, pcDNA3.1-Myc-UBE2G2, pcDNA3.1-His-TRIM38 were also constructed by Beijing Tsingke Biotechnology Co., Ltd.

**Western Blot**

Proteins were extracted from C57BL/6 mouse liver tissue and cultured cells using lysis buffer (P0013, Beyotime Biotechnology, Shanghai, China) supplemented with 1% phenylmethylsulfonyl fluoride (PMSF; YESEN, China) and 1% phosphatase inhibitor (EpiZyme, Shanghai, China). Protein concentrations were determined using the bicinchoninic acid (BCA) assay (Boster, China). Samples were denatured at 100 °C for 5 min and separated by 10% SDS-PAGE, followed by transfer to polyvinylidene difluoride membranes (Millipore, Boston, MA, USA).

Membranes were blocked with 5% non-fat milk and incubated overnight at 4 °C with the following primary antibodies: Antibodies against K48 (ab271911), p-JNK1/2/3 (ab124956), UBE2G2 (ab174296) and NRF2 (ab313825) were obtained from Abcam (Cambridge, UK). Antibodies against ERK1/2 (4695) and p-ERK1/2 (4370) were from Cell Signaling Technology (Danvers, MA, USA). Antibodies against BGN (T58375) and α-SMA (T55295) were from Abmart (Shanghai, China). Antibodies against COL1A1 (A24112), His (AE086) and Myc (AE070) were from Abclonal Technology (Wuhan, China). Antibodies against CD34 (14486-1-AP), LYVE1 (32649-1-AP), and β-actin (20536-1-AP) were from Proteintech (Wuhan, China). Antibody against GAPDH (GB15002-100) was from Servicebio (Wuhan, China). Antibody against HA (26183) was from Invitrogen (Carlsbad, CA, USA). Antibody against FLAG (F3165) was from Sigma-Aldrich (Germany). Antibodies against p38 (ET1702-65), phospho-p38 (T180+Y182) (ER2001-52), and JNK (ET1601-28) were from HUABIO (Hangzhou, China). HRP-conjugated secondary antibodies (goat anti-mouse IgG and goat anti-rabbit IgG) were purchased from ZSGB-BIO (Beijing, China).

Protein bands were visualized using a Bio-Rad ChemiDoc XRS+ imaging system after incubation with HRP-conjugated secondary antibodies and chemiluminescent detection.

**BGN protein stability assay**

To examine the stability of endogenous BGN protein, HUVEC and primary LSECs were treated with the protein synthesis inhibitor cycloheximide (CHX, 100 μM; MCE, Monmouth Junction, NJ, USA). Cells were harvested at 0, 12, 24, and 36 h after CHX treatment, and whole-cell lysates were prepared for protein analysis. For comparison of degradation kinetics, BGN expression levels at 0 h were set as baseline and used for normalization.

**Co-immunoprecipitation (IP) assay**

Primary LSECs and HUVEC were lysed in buffer containing 50 mM Tris-HCl (pH 7.4), 150 mM NaCl, 1 mM EDTA (Adamas-life), and 1% Triton X-100 (Adamas-life), supplemented with protease inhibitor cocktail (#4693159001, Roche, Switzerland) and phosphatase inhibitor (C0002, TargetMol, USA). After centrifugation to remove cell debris, the supernatant was incubated with an anti-UBE2G2 antibody at 4 °C overnight with gentle rotation. The following day, Protein A/G magnetic beads (HY-K0202, MCE, USA) were added and incubated for approximately 4 h. Immune complexes were washed thoroughly, and the precipitates were subjected to immunoblotting analysis.

**Chromatin immunoprecipitation assay**

In total, 6 × 10⁶ cells were cross-linked with 1% paraformaldehyde for 10 minutes at 37°C. Cell lysates were sonicated using a Bioruptor at high output power for 15 cycles (30 seconds ON and 30 seconds OFF). Supernatants were collected and incubated with anti-NRF2 antibody (ab137550, Cambridge, UK) or control IgG overnight at 4°C. Chromatin-antibody complexes were captured using protein A/G agarose beads (HY-K0202, MCE, USA), washed, and then eluted. DNA-protein complexes were reverse cross-linked in a water bath at 65°C for 4 hours and treated with proteinase K. DNA was purified using phenol-chloroform-isopentanol extraction, followed by ethanol precipitation. Finally, the DNA was quantified by real-time PCR.

**Immunofluorescence (IF) assay**

Cell coverslips or liver sections were fixed with 4% paraformaldehyde for 25 min and washed with PBS, followed by permeabilization with 0.1% Triton X-100 for 10 min. The samples were then incubated with the indicated primary antibodies at 4 °C overnight. On the following day, fluorophore-conjugated secondary antibodies were applied for 1 h at room temperature in the dark. After PBS washes, coverslips were mounted with an antifade mounting medium containing DAPI (G1407, Servicebio, Wuhan, China) for nuclear staining. Images were acquired using a Nikon confocal microscope (Nikon, Japan).

**RNA extraction and RT-qPCR**

Total RNA was extracted from liver tissue, LSECs, HSCs, HUVEC, and LX-2 cells using a total RNA extraction kit, following the manufacturer’s instructions. RNA concentration and purity were determined spectrophotometrically, and the RNA was reverse-transcribed into cDNA using a reverse transcription kit. The mRNA expression levels of target genes were quantified by real-time quantitative PCR (RT-qPCR). Relative mRNA expression was normalized to Actb and calculated using the 2^−ΔΔCT method. Primer sequences are provided in the Supplementary Materials.

**Luciferase reporter assay**

The 3’UTR regions of UBE2G2 were cloned into pGL3-Basic by Beijing Tsingke Biotech Co., Ltd. Cells co-transfected with reporter plasmids (UBE2G2-3’UTR-pGL) and pRL-TK control were harvested at 48 h. Luciferase activity was measured using the Dual-Luciferase Kit (Promega E1910). Data are presented as the mean ± SD from three independent experiments.

**Serum Biochemistry**

Serum alanine aminotransferase (ALT) and aspartate aminotransferase (AST) levels were determined using commercial assay kits (Nanjing Jiancheng Bioengineering Institute, Nanjing, China) in accordance with the manufacturer’s instructions.

**Network pharmacology analysis**

Hepatic fibrosis-related genes were collected from OMIM (https://omim.org), GeneCards (https://www.genecards.org/), DisGeNET (https://www.disgenet.org/)^1^, and TTD (http://db.idrblab.net/ttd/)^2^ databases and combined. The SMILES representations of SPD were retrieved from PubChem (http://pubchem.ncbi.nlm.nih.gov)^3^. For screening, SwissADME (http://www.swissadme.ch/)^4^ was employed, with gastrointestinal absorption (GI) set to "high" and drug similarity meeting at least two of the following "YES" criteria: Lipinski, Veber, and Egan. The identified SPD compounds were then input into SwissTargetPrediction (http://www.swisstargetprediction.ch/)^5^ , selecting those with a prediction probability > 0.1. All protein targets were standardized using the UniProt database (http://www.uniprot.org/). A Venn diagram was used to identify overlapping targets between hepatic fibrosis-related and SPD-related genes, which were considered as potential therapeutic targets of SPD for hepatic fibrosis.

Protein-protein interaction (PPI) networks were identified using STRING (https://string-db.org), and the results were visualised and analysed using Cytoscape 3.8.0^6^. Network topology analysis was performed using the Centiscape plugin, and key genes were identified based on degree centrality (DC) ≥ 2. Gene function and pathway enrichment analysis were performed using the GO database, the KEGG database, and the Metascape platform^7^. Finally, a ‘Component-Targets-Pathway’ network was constructed based on the above results.

**References**

1. Piñero J, Saüch J, Sanz F, Furlong LI. The DisGeNET cytoscape app: Exploring and visualizing disease genomics data. *Comput Struct Biotechnol J*. 2021;19:2960-2967. doi:10.1016/j.csbj.2021.05.015

2. Zhou Y, Zhang Y, Zhao D, et al. TTD: Therapeutic Target Database describing target druggability information. *Nucleic Acids Res*. 2024;52(D1):D1465-D1477. doi:10.1093/nar/gkad751

3. Kim S, Chen J, Cheng T, et al. PubChem 2023 update. *Nucleic Acids Res*. 2023;51(D1):D1373-D1380. doi:10.1093/nar/gkac956

4. Daina A, Michielin O, Zoete V. SwissADME: a free web tool to evaluate pharmacokinetics, drug-likeness and medicinal chemistry friendliness of small molecules. *Sci Rep*. 2017;7:42717. doi:10.1038/srep42717

5. Daina A, Michielin O, Zoete V. SwissTargetPrediction: updated data and new features for efficient prediction of protein targets of small molecules. *Nucleic Acids Res*. 2019;47(W1):W357-W364. doi:10.1093/nar/gkz382

6. Shannon P, Markiel A, Ozier O, et al. Cytoscape: a software environment for integrated models of biomolecular interaction networks. *Genome Res*. 2003;13(11):2498-2504. doi:10.1101/gr.1239303

7. Zhou Y, Zhou B, Pache L, et al. Metascape provides a biologist-oriented resource for the analysis of systems-level datasets. *Nat Commun*. 2019;10(1):1523. doi:10.1038/s41467-019-09234-6

**Supplementary Figures**

**
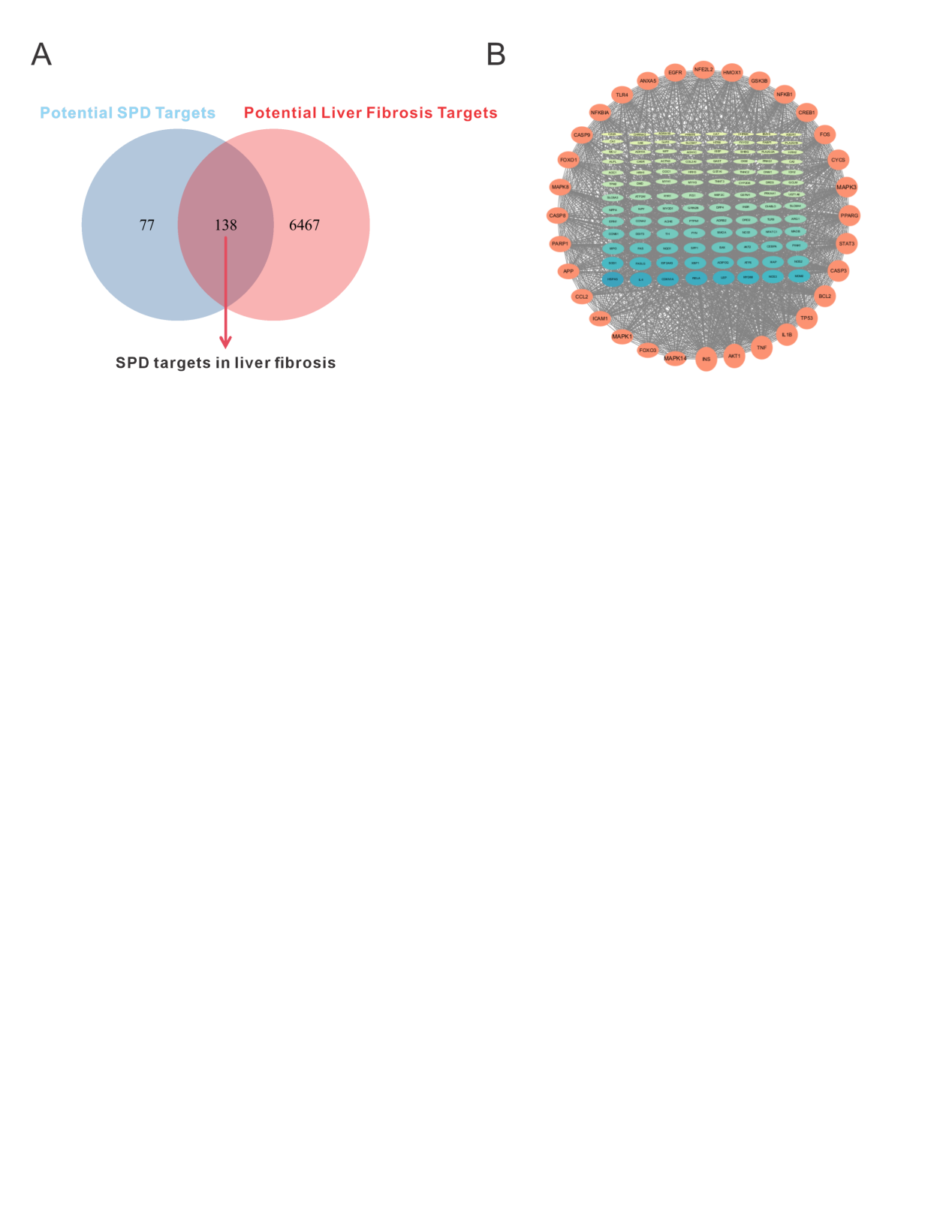
 Figure S1.** **Network Pharmacology and PPI Analysis of SPD in Liver Fibrosis Treatment.** (A) Venn diagram showing the overlapped targets by Network pharmacology analysis of SPD intake and liver fibrosis. (B) PPI network analysis: 138 key targets selected by plug-in Centiscape (degree centrality≥2).

**
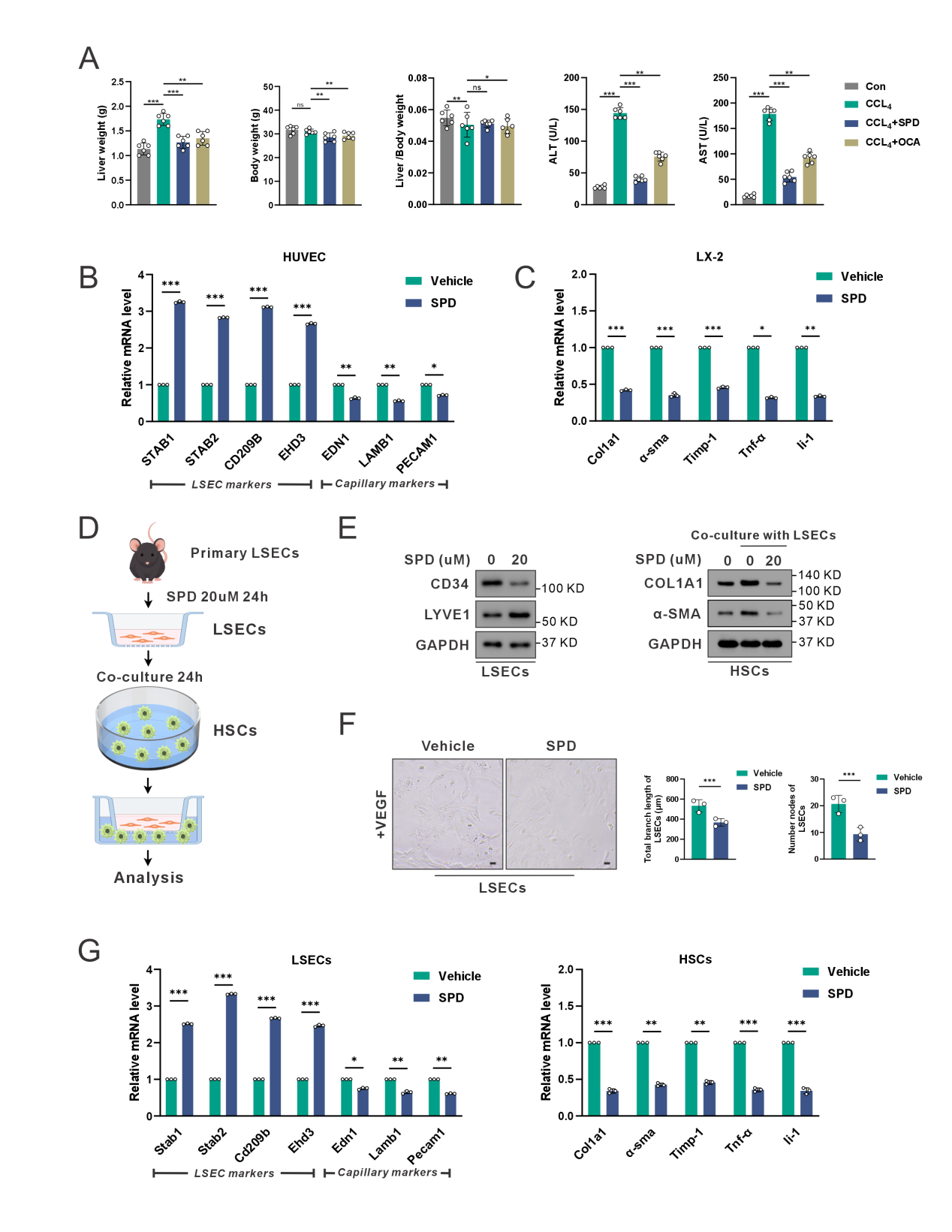
 Figure S2.** **SPD attenuates hepatic fibrosis through amelioration of LSECs dysfunction *in vitro*.** (A) Body weight, liver weight, liver/body weight ratio and serum ALT and AST levels in each group. (B-C) LX-2 cells were co-cultured with SPD-treated (or untreated) HUVEC. Analysis of mRNA levels of indicated markers in primary HUVEC (B) and LX-2 cells (C) were measured by RT-qPCR (n = 3, performed in biological replicates). (D) Schematic of the co-culture model. Primary HSCs were co-cultured with SPD-treated (or untreated) primary LSECs. (E) Analysis of CD34 and LYVE1 expression in SPD-treated LSECs, and COL1A1 and α-SMA expression in HSCs by western blot. (F) Matrigel tube-formation assay in Primary LSECs (n = 3, performed in biological replicates). Cells were pretreated with SPD (20 μM) for 24 h, then seeded on matrigel-coated 48-well plates and stimulated with VEGFA (25 ng/mL) for 24 h. Images were captured 24 h after seeding. (G) Analysis of mRNA levels of indicated markers in primary LSECs (left) or primary HSCs (right) by RT-qPCR (n = 3, performed in biological replicates). Data are represented as mean ± SD, **P* < 0.05, ***P* < 0.01, ****P* < 0.001.


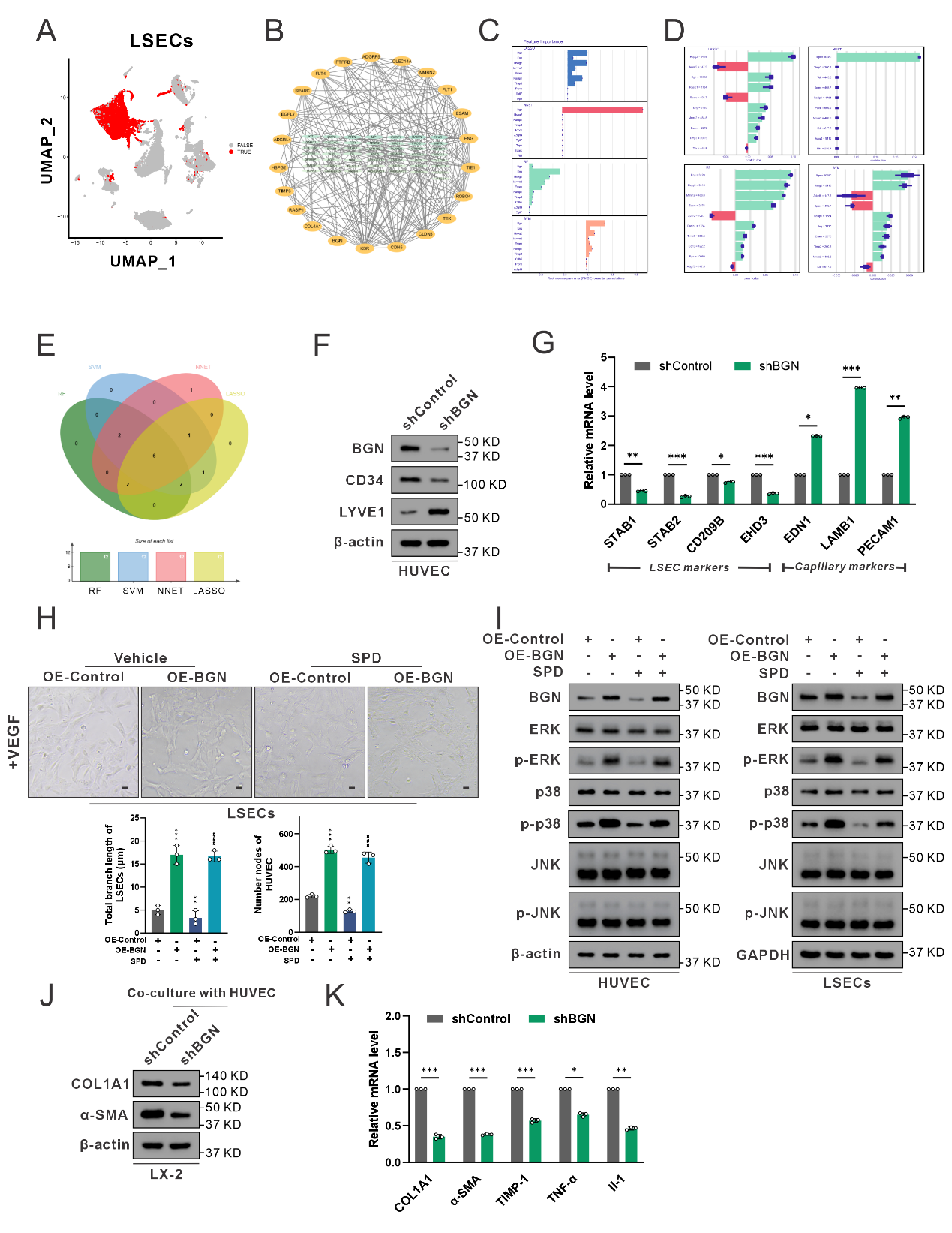
 **Figure S3. SPD suppresses LSECs capillarization and HSCs activation through downregulation of LSECs-derived BGN.** (A) Single-cell data for LSECs were obtained from single-cell RNA-seq analysis of mouse liver tissues in the GSE268846 dataset. (B) PPI network analysis: 22 key targets selected by plug-in Centiscape (degree centrality ≥9). (C) Feature importance evaluation of key targets using four machine learning models based on transcriptomic data train dataset. (D) Validation of target importance on validation dataset by break down method and SHAP value. (E) Intersection of top 10 importance genes among four models, yielding 6 core genes. (F-G) HUVECs were transfected with BGN or Control shRNA lentivirus. (F) Analysis of protein levels of BGN, CD34 and LYVE1 expression in HUVEC by western blot. (G) Analysis of mRNA levels of STAB1, STAB2, CD209B, EHD3, CD34, EDN1, LAMB1 and PECAM1 in HUVEC by RT-qPCR. (H-I) HUVEC or primary LSECs were infected with AdBGN to overexpress BGN (OE-BGN) or AdControl (OE-Control) for 36 h, followed by treated with or without SPD (20 μM) for 24 h. (H) LSECs were seeded on matrigel-coated 48-well plates and treated with VEGFA for an additional 24 h, then images were captured (n = 3, performed in biological replicates). (I) Protein levels of MAPK signaling pathway-related targets. (J-K) LX-2 cells were co-cultured with HUVEC transfected with BGN shRNA lentivirus. (J) Analysis of protein levels of COL1A1 and α-SMA in LX-2 cells. (K) Analysis of mRNA levels of fibrotic markers in LX-2 cells. For H, * indicated OE-BGN or OE-Control + SPD vs OE-Control, ^#^ indicated OE-BGN+ SPD vs OE-Control + SPD. Data are represented as mean ± SD, **P* < 0.05, ***P* < 0.01, ****P* < 0.001; ^#^*P* < 0.05, ^##^*P* < 0.01, ^###^*P* < 0.001.


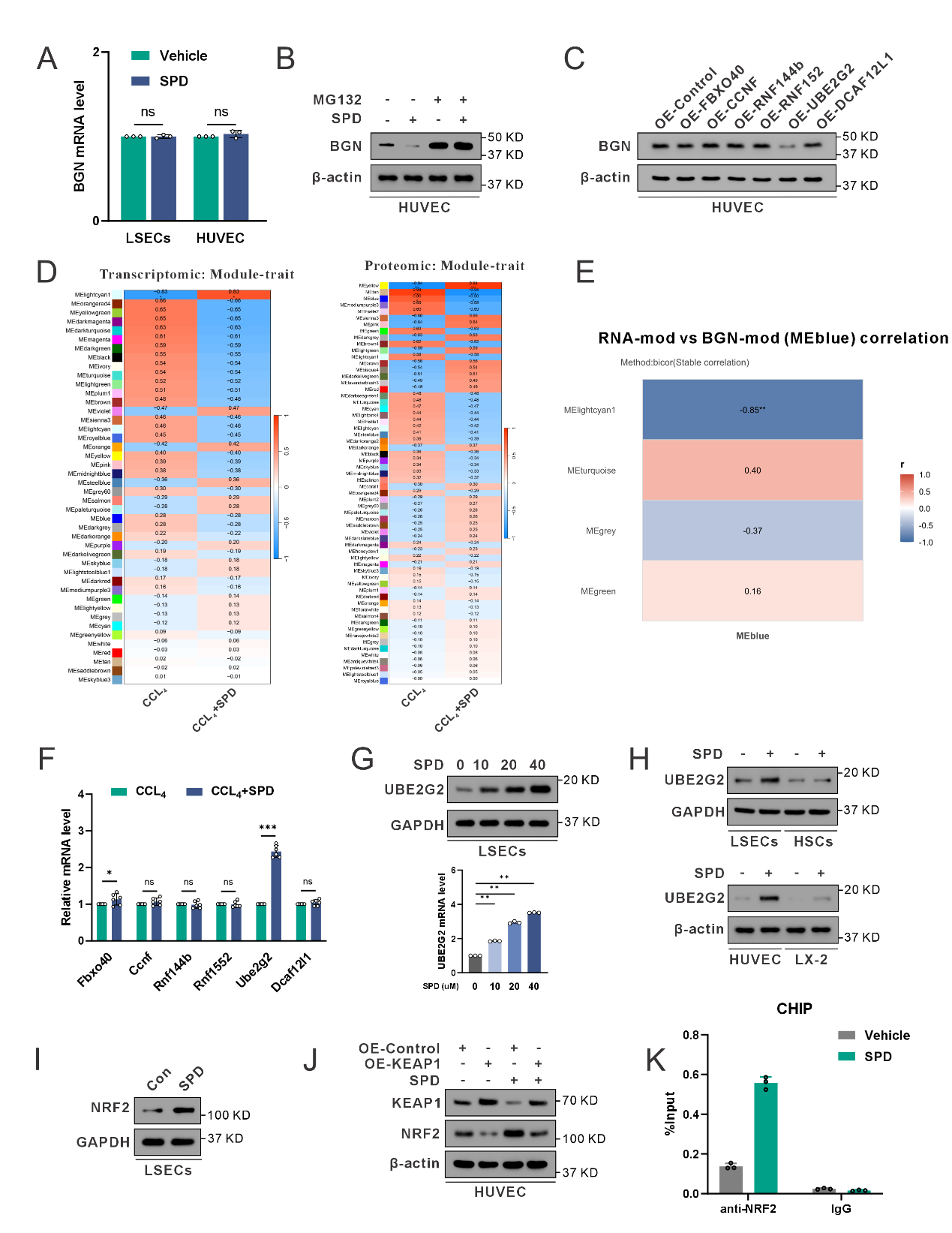
 **Figure S4. SPD upregulates UBE2G2 via activation of NRF2.** (A) Analysis of mRNA levels of BGN in CCl_4_-induced mice model (n = 3, performed in biological replicates). (B) HUVECs were treated with or without MG132 (10 μM) and/or SPD (20 μM). BGN protein levels were analyzed by western blot. (C) Analysis of BGN expression in HUVEC overexpressing core genes by western blot. (D) Module-trait relationships in WGCNA for transcriptomic (left panel) and proteomic (right panel) datasets (CCl_4_ and CCl_4_+SPD groups). (E) Correlation between RNA modules (y-axis) and the BGN-containing module (MEblue) (x-axis) in WGCNA. (F) Analysis of DEGs expression in SPD-treated CCl_4_-induced mice model by RT-qPCR (n = 6, performed in biological replicates). (G) Analysis of UBE2G2 expression in LSECs treated with different concentrations of SPD by western blot and RT-qPCR (n = 3, performed in biological replicates). (H) Comparison of UBE2G2 expression induced by SPD in different cell types. (I) Primary LSECs were treated with SPD (20 μM) for 24 h, and NRF2 expression was analyzed by western blot. (J) Protein levels of KEAP1 and NRF2 in HUVEC treated with SPD and overexpressing either OE-KEAP1 or OE-Control. (K) NRF2 binding to the *UBE2G2* gene promoter region was determined by chromatin immunoprecipitation (ChIP) assays. HUVECs were treated with SPD or vehicle as indicated. IgG was used as a negative control. Data are represented as mean ± SD, **P* < 0.05, ***P* < 0.01, ****P* < 0.001.

**
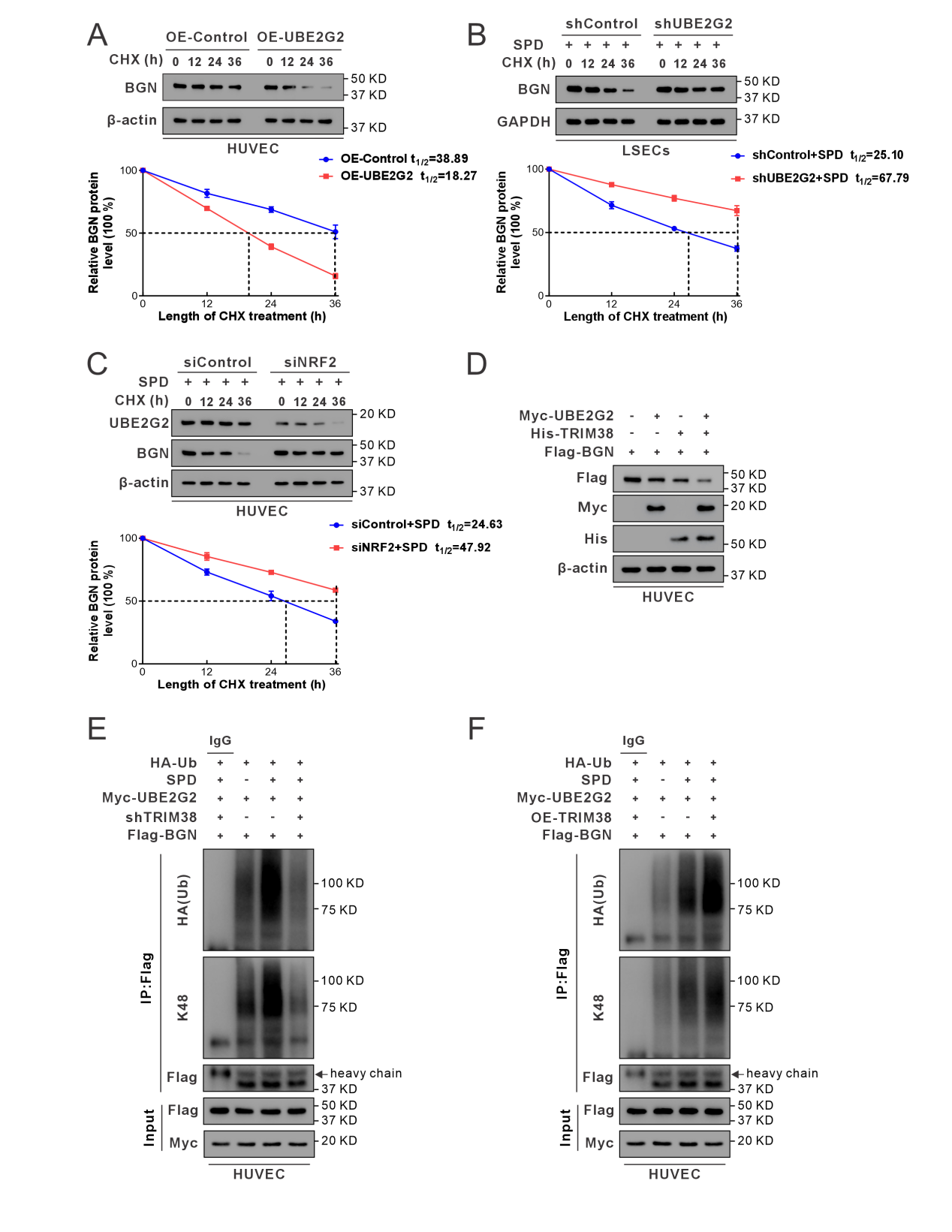
 Figure S5.** **SPD upregulates UBE2G2 to reduce the protein stability of BGN.** (A) HUVECs were transfected with AdUBE2G2 or AdControl, followed by CHX (100 μM) treatment for the indicated times (n = 3, performed in biological replicates). (B) Primary LSECs were transfected with shUBE2G2 or control shRNA lentivirus, combined with SPD treatment (20 μM), followed by CHX (100 μM) treatment for the indicated times (n = 3, performed in biological replicates). (C) HUVECs were transfected with siNRF2 or control siRNA lentivirus, combined with SPD treatment (20 μM), followed by CHX (100 μM) treatment for the indicated times (n = 3, performed in biological replicates). (D) The effect of TRIM38 cooperating with UBE2G2 on regulating BGN expression was detected by western blot. (E-F) HUVECs were co-transfected with Flag-BGN and Myc-UBE2G2, followed by transfection with either shTRIM38 (E) or AdTRIM38 (F). The cells were subsequently treated with or without SPD (20 μM) for 24 hours. BGN ubiquitination was assessed by immunoprecipitation using an anti-Flag antibody. Arrow indicates the IgG heavy chain band. All the presented input was adjusted to a similar level for the following assay.


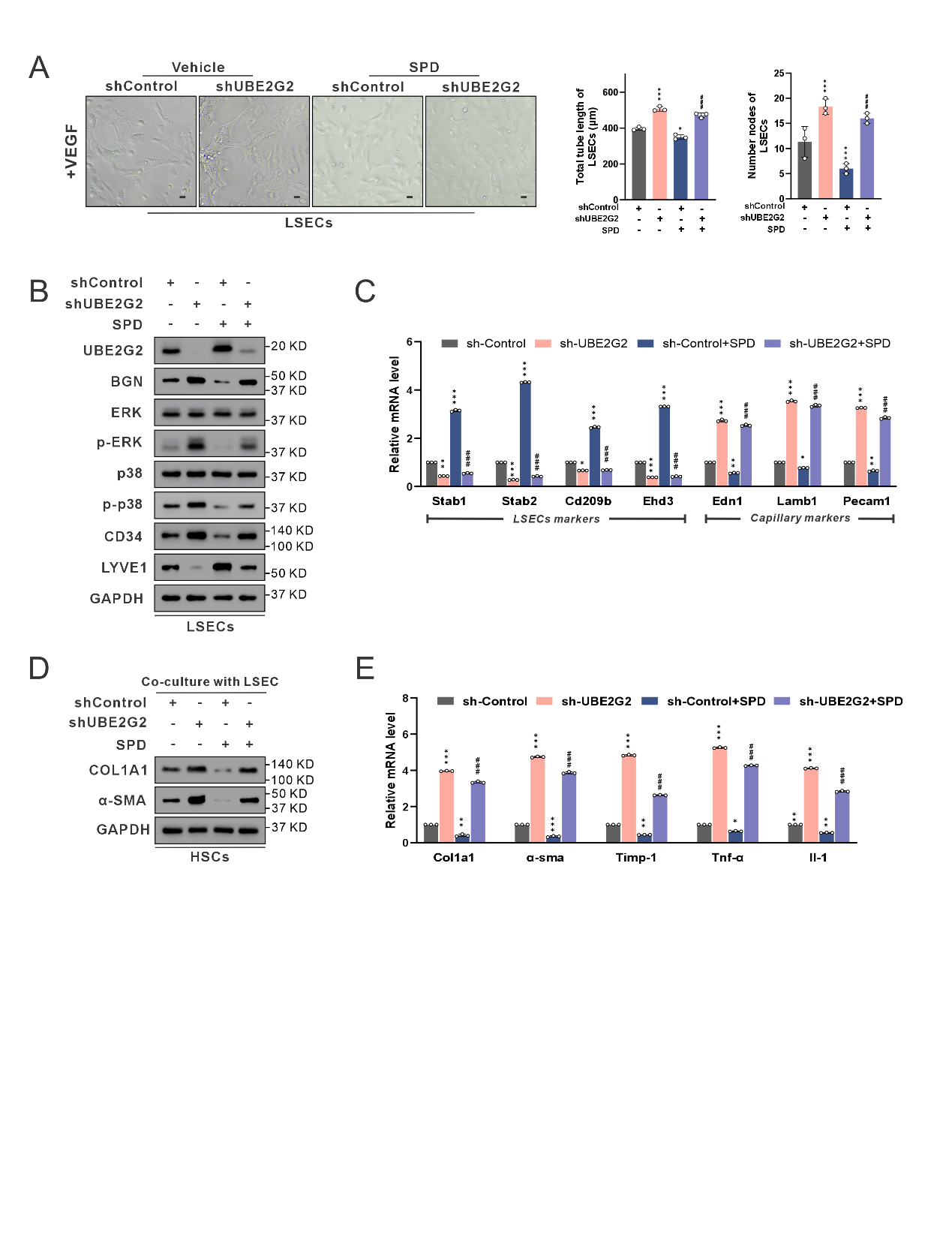
 **Figure S6.** **SPD ameliorates LSECs dysfunction and restrains HSCs activation via UBE2G2-mediated BGN ubiquitination.** (A-C) Primary LSECs were transfected with UBE2G2 or Control shRNA lentivirus, combined with (or without) SPD (20 μM), and analyzed by tube-formation assay (A), western blot (B) and RT-qPCR (C, n = 3, performed in biological replicates). (D-E) Primary HSCs were co-cultured with LSECs-treated as above mentioned. (D) Protein levels of COL1A1 and α-SMA in primary HSCs were determined by western blot. (E) mRNA levels of fibrotic markers in primary HSCs were determined by RT-qPCR (n = 3, performed in biological replicates). For A, C and E, * indicated shUBE2G2 or shControl + SPD vs shControl, ^#^ indicated shUBE2G2+ SPD vs shControl + SPD; Data are represented as mean ± SD, **P* < 0.05, ***P* < 0.01, ****P* < 0.001; ^#^*P* < 0.05, ^##^*P* < 0.01, ^###^*P* < 0.001.


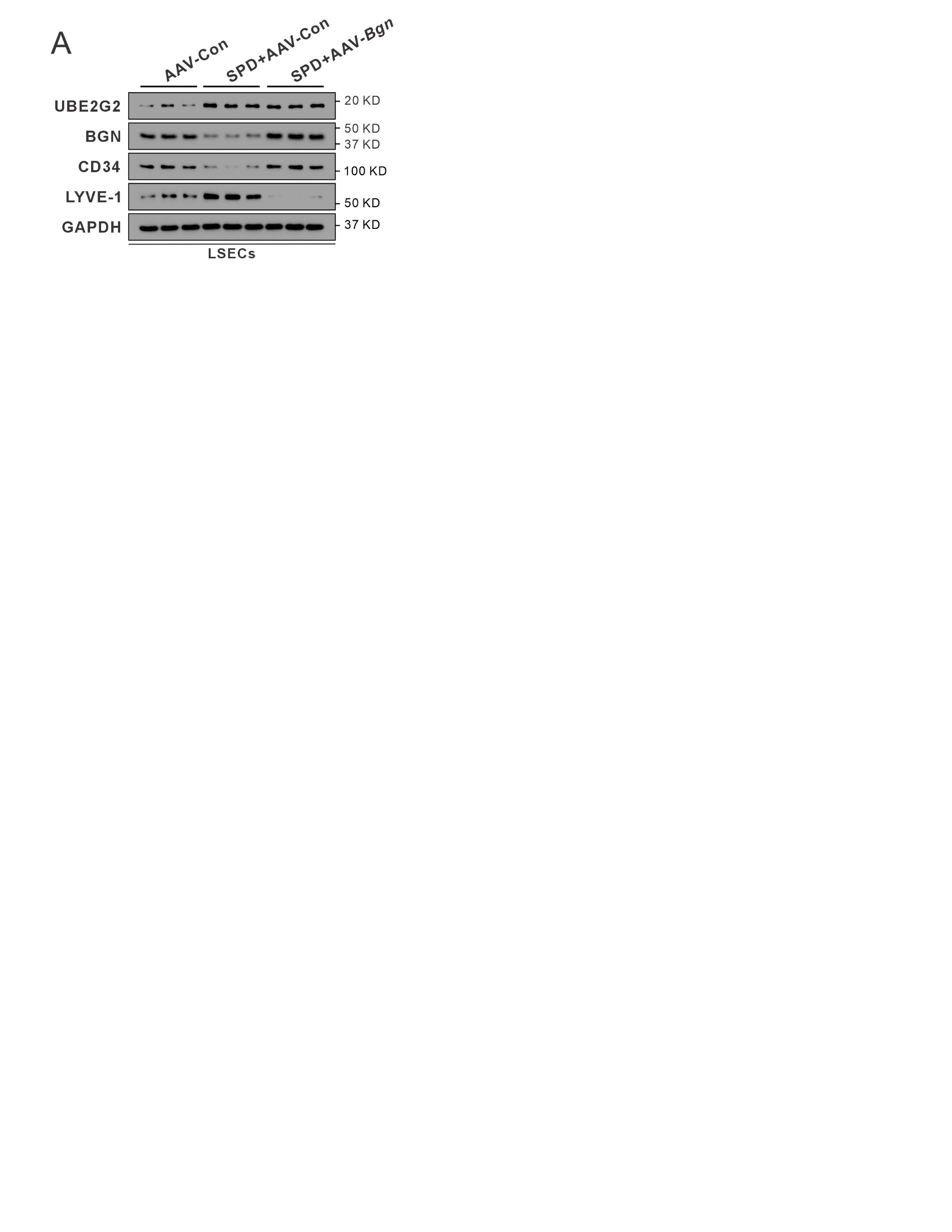


**Figure S7. *Bgn* overexpression counteracts the therapeutic effects of SPD in fibrosis.** (A) Analysis of related protein levels in the isolated LSECs (n = 3, performed in biological replicates).

**Table S1.** Liver fibrosis-related targets (n = 6605) identified from OMIM, GeneCards, DisGeNET, and TTD databases.

| **No.** | **Target** | **No.** | **Target** | **No.** | **Target** | **No.** | **Target** |
| --- | --- | --- | --- | --- | --- | --- | --- |
| 1 | PKHD1 | 2 | PKD1 | 3 | PNPLA3 | 4 | PRKCSH |
| 5 | SEC63 | 6 | PKD2 | 7 | UTP4 | 8 | SERPINA1 |
| 9 | HFE | 10 | MARS1 | 11 | TP53 | 12 | MIR122 |
| 13 | ALB | 14 | LRP5 | 15 | KRT18 | 16 | GYS2 |
| 17 | TRMU | 18 | CTNNB1 | 19 | GPT | 20 | GANAB |
| 21 | SLC17A5 | 22 | MIR7-3HG | 23 | ABCB4 | 24 | NR1H4 |
| 25 | INS | 26 | TNF | 27 | TGFB1 | 28 | KRT8 |
| 29 | HNF4A | 30 | HULC | 31 | APC | 32 | ABCB11 |
| 33 | IL6 | 34 | ATP7B | 35 | IL21R | 36 | F2 |
| 37 | AFP | 38 | PPARG | 39 | PHKA2 | 40 | MET |
| 41 | SLC30A10 | 42 | MIR17 | 43 | NBAS | 44 | FARSB |
| 45 | PHKG2 | 46 | CYP2E1 | 47 | MEG3 | 48 | HAMP |
| 49 | MIR21 | 50 | ALG8 | 51 | GGT1 | 52 | PYGL |
| 53 | MALAT1 | 54 | LOC126859690 | 55 | TERT | 56 | GAS5 |
| 57 | CP | 58 | TSC2 | 59 | CYP3A4 | 60 | FAH |
| 61 | TJP2 | 62 | STAT3 | 63 | POLG | 64 | PIK3CA |
| 65 | IL12RB1 | 66 | HOTAIR | 67 | ABCC2 | 68 | LIPA |
| 69 | MIR150 | 70 | ATP8B1 | 71 | HNF1A | 72 | G6PC1 |
| 73 | SLC40A1 | 74 | CFTR | 75 | HJV | 76 | TFR2 |
| 77 | MIR212 | 78 | SLCO1B1 | 79 | MIR451A | 80 | H19 |
| 81 | SLC25A13 | 82 | KRAS | 83 | MIR30D | 84 | TSC1 |
| 85 | KRT7 | 86 | INSR | 87 | IL10 | 88 | UGT1A1 |
| 89 | HADHA | 90 | CASP8 | 91 | HNF1B | 92 | MIR223 |
| 93 | CYP7A1 | 94 | MIR34A | 95 | SMAD4 | 96 | IFNG |
| 97 | VDR | 98 | ALDOB | 99 | AKT1 | 100 | DLAT |
| 101 | GBE1 | 102 | CPT1A | 103 | MIRLET7B | 104 | EGFR |
| 105 | ADIPOQ | 106 | MIR20A | 107 | CDKN2A | 108 | AXIN1 |
| 109 | HGF | 110 | ARG1 | 111 | ALDH2 | 112 | CBS |
| 113 | AGL | 114 | SLC11A2 | 115 | GPC3 | 116 | APOB |
| 117 | IL12A | 118 | MIR27A | 119 | HLA-DRB1 | 120 | PHKB |
| 121 | SLC10A1 | 122 | PPARA | 123 | APOE | 124 | BMP6 |
| 125 | MPV17 | 126 | MIR324 | 127 | MIR200B | 128 | ICAM1 |
| 129 | LARS1 | 130 | MIR483 | 131 | GFER | 132 | MIRLET7C |
| 133 | CCND1 | 134 | DCDC2 | 135 | MIR126 | 136 | GLUL |
| 137 | IRF5 | 138 | SLC2A2 | 139 | DGUOK | 140 | TNPO3 |
| 141 | CYP1A2 | 142 | TNFSF15 | 143 | MIR22 | 144 | MIR30A |
| 145 | MIR494 | 146 | LIVAR | 147 | PTEN | 148 | LMNA |
| 149 | IL1B | 150 | NR1H3 | 151 | LEP | 152 | NOTCH1 |
| 153 | IGF2R | 154 | TF | 155 | SPIB | 156 | MIR142 |
| 157 | OTC | 158 | IL21R-AS1 | 159 | HSD3B7 | 160 | FABP1 |
| 161 | MIR200A | 162 | MIR130A | 163 | JAG1 | 164 | APOA1 |
| 165 | CYP7B1 | 166 | CRP | 167 | SLCO1B3 | 168 | SEPSECS |
| 169 | NR1H2 | 170 | TM6SF2 | 171 | KIT | 172 | ASS1 |
| 173 | GCK | 174 | NOTCH2 | 175 | HSD17B13 | 176 | PKLR |
| 177 | FOCAD | 178 | BCS1L | 179 | AGT | 180 | MIR155 |
| 181 | FBP1 | 182 | BRAF | 183 | POU2AF1 | 184 | CYP2A6 |
| 185 | NR1I2 | 186 | MIR132 | 187 | MTTP | 188 | MMEL1 |
| 189 | SMPD1 | 190 | AKR1D1 | 191 | ALPP | 192 | CTLA4 |
| 193 | PFKL | 194 | FGFR2 | 195 | CAV1 | 196 | TTR |
| 197 | MIR214 | 198 | NPHP3 | 199 | HMOX1 | 200 | GAA |
| 201 | TMEM67 | 202 | SLC37A4 | 203 | YAP1 | 204 | FAS |
| 205 | IFNA1 | 206 | MIR29A | 207 | MIR361 | 208 | MIR26A1 |
| 209 | MIR203A | 210 | TYMP | 211 | TLR4 | 212 | REN |
| 213 | GSTM1 | 214 | FECH | 215 | MIR197 | 216 | LBR |
| 217 | MIR31 | 218 | MIR296 | 219 | FOXM1 | 220 | GNAS |
| 221 | ADH1B | 222 | NPC1 | 223 | KRT19 | 224 | ATM |
| 225 | CYP27A1 | 226 | PC | 227 | MIRLET7D | 228 | MIR27B |
| 229 | ABCB1 | 230 | SERPINC1 | 231 | MIR140 | 232 | LDLR |
| 233 | FTH1 | 234 | NF1 | 235 | SRC | 236 | PDGFRA |
| 237 | PTPN11 | 238 | ENG | 239 | HRAS | 240 | CPT2 |
| 241 | MIR328 | 242 | GBA1 | 243 | MIR151A | 244 | PBC2 |
| 245 | PBC3 | 246 | PBC4 | 247 | PBC5 | 248 | PVT1 |
| 249 | MIR183 | 250 | CXCL8 | 251 | MTHFR | 252 | APTR |
| 253 | EGF | 254 | MIR191 | 255 | ABCG5 | 256 | MIR133B |
| 257 | MIR378A | 258 | HP | 259 | MIR299 | 260 | BAAT |
| 261 | CCL2 | 262 | AMACR | 263 | MIR125A | 264 | RAF1 |
| 265 | IGF1 | 266 | CD40LG | 267 | ERBB2 | 268 | MIR145 |
| 269 | SLC2A1 | 270 | TALDO1 | 271 | ARID1A | 272 | JAK2 |
| 273 | MIR107 | 274 | CDKN3 | 275 | TIMP1 | 276 | INVS |
| 277 | MIR15A | 278 | ABCC3 | 279 | SREBF1 | 280 | TNFRSF1A |
| 281 | MIR148A | 282 | NRAS | 283 | CYP2D6 | 284 | MIRLET7E |
| 285 | MIR33A | 286 | NAFLD1 | 287 | ACE | 288 | ACOX1 |
| 289 | ALG9 | 290 | FGFR1 | 291 | AKR1A1 | 292 | MIR200C |
| 293 | CDC25A | 294 | PFKFB1 | 295 | TUG1 | 296 | ELN |
| 297 | MIR139 | 298 | NPHP1 | 299 | MIR519D | 300 | MYO5B |
| 301 | ABCG8 | 302 | VEGFA | 303 | GNMT | 304 | MYC |
| 305 | FASLG | 306 | YARS1 | 307 | MIR29C | 308 | MMUT |
| 309 | CC2D2A | 310 | RBP4 | 311 | LEPR | 312 | SST |
| 313 | MIR181B1 | 314 | PCSK9 | 315 | LOC126806659 | 316 | ASL |
| 317 | DNAJB11 | 318 | MUC1 | 319 | STK11 | 320 | NPHP4 |
| 321 | FH | 322 | MIR326 | 323 | POLG2 | 324 | AGXT |
| 325 | SOD2 | 326 | MKS1 | 327 | HBB | 328 | MIR10B |
| 329 | IDH2 | 330 | OFD1 | 331 | IFNL3 | 332 | F5 |
| 333 | NPC2 | 334 | MIR146A | 335 | MAP2K1 | 336 | CALR |
| 337 | IDH1 | 338 | WRAP53 | 339 | ALPL | 340 | IGF2 |
| 341 | ADH1C | 342 | TAT | 343 | GSTP1 | 344 | MIR16-1 |
| 345 | MBL2 | 346 | SLC51A | 347 | FOXP3 | 348 | MIR222 |
| 349 | ACADVL | 350 | CALCA | 351 | ESR1 | 352 | GPT2 |
| 353 | PMS2 | 354 | RPGRIP1L | 355 | LOC106096416 | 356 | IL2 |
| 357 | ALAS2 | 358 | MAT1A | 359 | UROD | 360 | RET |
| 361 | PMM2 | 362 | CLDN1 | 363 | LCAT | 364 | ABCB7 |
| 365 | PCK1 | 366 | PDGFRL | 367 | CDH1 | 368 | NFKB1 |
| 369 | SERPINE1 | 370 | LPL | 371 | ACADM | 372 | KRT20 |
| 373 | F3 | 374 | NEAT1 | 375 | MIR106B | 376 | UGT1A9 |
| 377 | KCNQ1OT1 | 378 | AXL | 379 | HSD17B4 | 380 | ALK |
| 381 | MIR23A | 382 | DHCR7 | 383 | EPCAM | 384 | XBP1 |
| 385 | RAD50 | 386 | NAGLU | 387 | SLC10A2 | 388 | MIR18A |
| 389 | IL1A | 390 | CYP2C19 | 391 | GUSB | 392 | HLA-DQB1 |
| 393 | FBN1 | 394 | TCF4 | 395 | MIR215 | 396 | PNPLA2 |
| 397 | MIR346 | 398 | GPBAR1 | 399 | BCL2 | 400 | MIR221 |
| 401 | SMO | 402 | MIR181A1 | 403 | HFE-AS1 | 404 | NBN |
| 405 | CYP1A1 | 406 | FUCA1 | 407 | MIR25 | 408 | CEBPA |
| 409 | SEC61A1 | 410 | EDN1 | 411 | PEX1 | 412 | HLA-B |
| 413 | UGT1A7 | 414 | NR1I3 | 415 | ONECUT1 | 416 | IL2RA |
| 417 | IL4 | 418 | MPI | 419 | RETN | 420 | AGPAT2 |
| 421 | PGM1 | 422 | IGFBP3 | 423 | EPHX1 | 424 | HMGCL |
| 425 | BMP2 | 426 | MIR141 | 427 | CD14 | 428 | ENSG00000266919 |
| 429 | MIR301A | 430 | ABCA1 | 431 | VHL | 432 | BAP1 |
| 433 | CEP290 | 434 | LOC110806263 | 435 | SLC51B | 436 | HIF1A |
| 437 | FOXA2 | 438 | MTOR | 439 | ALDOA | 440 | MIR154 |
| 441 | FGF19 | 442 | PBRM1 | 443 | MIR187 | 444 | STAT1 |
| 445 | PRKAG2 | 446 | MIR199A1 | 447 | PDHX | 448 | NTRK1 |
| 449 | NOS2 | 450 | TMEM216 | 451 | MIR182 | 452 | STARD13 |
| 453 | SNHG1 | 454 | PYGM | 455 | CAMTA1 | 456 | CCN2 |
| 457 | MIR26B | 458 | FGFR3 | 459 | MIR30E | 460 | EPO |
| 461 | MIR500A | 462 | MIR143 | 463 | MIR199B | 464 | ATG7 |
| 465 | NOS3 | 466 | TLR2 | 467 | USP53 | 468 | SLC4A2 |
| 469 | G6PD | 470 | SULT2A1 | 471 | TWNK | 472 | KIF12 |
| 473 | NR0B2 | 474 | GLUD1 | 475 | ABHD5 | 476 | BAX |
| 477 | CES1 | 478 | MIR152 | 479 | MMP9 | 480 | HNF1A-AS1 |
| 481 | ALDH9A1 | 482 | HMGCR | 483 | PEX5 | 484 | UCA1 |
| 485 | LIPC | 486 | IL17A | 487 | XDH | 488 | NPPA |
| 489 | RBCK1 | 490 | GSTT1 | 491 | MIR195 | 492 | MECOM |
| 493 | NRG1 | 494 | APOC3 | 495 | MIF | 496 | MSR1 |
| 497 | DBT | 498 | CD36 | 499 | IDUA | 500 | CERNA3 |
| 501 | CEBPB | 502 | PPARGC1A | 503 | NR5A2 | 504 | RXRA |
| 505 | ALAD | 506 | NEK8 | 507 | ROS1 | 508 | MIR216A |
| 509 | BHMT | 510 | MIR196A2 | 511 | TFAM | 512 | IGF2-AS |
| 513 | TFDP1 | 514 | CAVIN1 | 515 | DLD | 516 | JUN |
| 517 | AKT2 | 518 | MIR224 | 519 | DDR2 | 520 | RRM2B |
| 521 | SLC22A5 | 522 | XIST | 523 | HPD | 524 | MAPK8 |
| 525 | GAPDH | 526 | GALT | 527 | ADAR | 528 | PEX2 |
| 529 | MMP2 | 530 | LDHA | 531 | MIR125B1 | 532 | CCAT1 |
| 533 | MIR23B | 534 | GNE | 535 | CCR5 | 536 | MYOM1 |
| 537 | GLS2 | 538 | UGT1A6 | 539 | NTRK2 | 540 | ASGR2 |
| 541 | PSAP | 542 | FASN | 543 | PEMT | 544 | ACVRL1 |
| 545 | SI | 546 | NR3C1 | 547 | BSCL2 | 548 | CDKN2B-AS1 |
| 549 | HMBS | 550 | CYP2B6 | 551 | VWF | 552 | SCT |
| 553 | MIR30C1 | 554 | MIR19A | 555 | LTBP4 | 556 | MIR338 |
| 557 | NOD2 | 558 | SUCLG1 | 559 | FLCN | 560 | CD79A |
| 561 | MIR93 | 562 | PCAT1 | 563 | GYS1 | 564 | HK1 |
| 565 | CYP2C9 | 566 | MIRLET7A1 | 567 | PPIG | 568 | ADAMTS13 |
| 569 | DPYD | 570 | IRS1 | 571 | PHKA1 | 572 | LOC126806658 |
| 573 | SOD1 | 574 | RUVBL1 | 575 | FANCI | 576 | PEX6 |
| 577 | GPI | 578 | MTUS1 | 579 | MIR219A1 | 580 | IGFBP1 |
| 581 | TREX1 | 582 | PCK2 | 583 | ETFDH | 584 | ERBB4 |
| 585 | ABCC4 | 586 | UCP2 | 587 | UGT1A4 | 588 | LOC129933155 |
| 589 | CPS1 | 590 | GLS | 591 | TGFBR2 | 592 | NTRK3 |
| 593 | HADHB | 594 | VPS33B | 595 | CAT | 596 | PEX16 |
| 597 | MIR429 | 598 | SNHG15 | 599 | IFT172 | 600 | KDR |
| 601 | MIR185 | 602 | FGFR4 | 603 | THPO | 604 | GLA |
| 605 | SLC5A5 | 606 | DKK3 | 607 | CXCL10 | 608 | IDS |
| 609 | HADH | 610 | MIR205 | 611 | SLC27A5 | 612 | SNHG12 |
| 613 | MIR127 | 614 | HLA-DQA1 | 615 | IL6R | 616 | CETP |
| 617 | PDCD1 | 618 | SLPI | 619 | MIR99A | 620 | SRA1 |
| 621 | SOCS3 | 622 | AHSG | 623 | BRCA2 | 624 | SOD2-OT1 |
| 625 | SKIC2 | 626 | SLC25A20 | 627 | AGTR1 | 628 | IFNA2 |
| 629 | CDH17 | 630 | STEAP4 | 631 | SNHG6 | 632 | INPP5E |
| 633 | UMOD | 634 | LAMP2 | 635 | NAT2 | 636 | MIR24-2 |
| 637 | PEX11B | 638 | SMAD3 | 639 | ITGB1 | 640 | HSPA6 |
| 641 | CYTOR | 642 | SKIC3 | 643 | WDR19 | 644 | PCNA |
| 645 | GOLM1 | 646 | NFE2L2 | 647 | CRNDE | 648 | LINC01419 |
| 649 | LIPE | 650 | MIR96 | 651 | PTH | 652 | PEX13 |
| 653 | MIR128-2 | 654 | MMP1 | 655 | CPOX | 656 | DGCR5 |
| 657 | MIR186 | 658 | CES2 | 659 | ADA | 660 | SLC25A15 |
| 661 | SLC7A7 | 662 | PRPSAP1 | 663 | SCARB1 | 664 | DANCR |
| 665 | LINC00665 | 666 | MIR26A2 | 667 | HAGLR | 668 | CD34 |
| 669 | RB1 | 670 | TIMP2 | 671 | DPP4 | 672 | TCF7 |
| 673 | CEACAM3 | 674 | CCAT2 | 675 | MIRLET7G | 676 | SCP2 |
| 677 | IL18 | 678 | NEU1 | 679 | HOTTIP | 680 | LNCARSR |
| 681 | CASC15 | 682 | PEX14 | 683 | MIR22HG | 684 | PLIN2 |
| 685 | FOS | 686 | FOXO1 | 687 | TTC21B | 688 | WDR35 |
| 689 | MST1 | 690 | IFNAR2 | 691 | WT1 | 692 | MAP3K20 |
| 693 | ZFAS1 | 694 | MIR125B2 | 695 | SPP1 | 696 | MIR103A2 |
| 697 | UROS | 698 | BGLAP | 699 | ASAH1 | 700 | GLB1 |
| 701 | SIRT1 | 702 | CEP164 | 703 | MIR95 | 704 | ACADS |
| 705 | MIR148B | 706 | MIR181A2 | 707 | GDF2 | 708 | CXCL12 |
| 709 | PCBP2-OT1 | 710 | JAK1 | 711 | LNCRNA-ATB | 712 | B2M |
| 713 | FLNA | 714 | BTD | 715 | NPTN-IT1 | 716 | TYMS |
| 717 | HOXA11-AS | 718 | PFKM | 719 | CDH4 | 720 | RIOX2 |
| 721 | MT-TP | 722 | MT1DP | 723 | FALEC | 724 | UGT1A |
| 725 | PLG | 726 | PAH | 727 | DLC1 | 728 | GALK1 |
| 729 | URB2 | 730 | CXCR4 | 731 | BBS2 | 732 | LINC00261 |
| 733 | TP53COR1 | 734 | DBH-AS1 | 735 | SNHG20 | 736 | ALMS1 |
| 737 | TTF1 | 738 | POLR1HASP | 739 | BBS4 | 740 | SPRY4-IT1 |
| 741 | LBP | 742 | ARSA | 743 | CD46 | 744 | GYG1 |
| 745 | NAGS | 746 | SLC11A1 | 747 | MIR100HG | 748 | ACAD9 |
| 749 | MIR136 | 750 | CD4 | 751 | MPO | 752 | SLC7A6 |
| 753 | AFAP1-AS1 | 754 | MCU | 755 | PTPRC | 756 | PLIN1 |
| 757 | LINC-ROR | 758 | BMP7 | 759 | PDGFRB | 760 | MIR137 |
| 761 | BBS1 | 762 | DZIP1L | 763 | SMARCA4 | 764 | MIR374A |
| 765 | LINC00941 | 766 | NORAD | 767 | B9D1 | 768 | LINC00926 |
| 769 | HOXA13 | 770 | LINC01018 | 771 | CBR3-AS1 | 772 | MVK |
| 773 | COL4A1 | 774 | THADA | 775 | PANDAR | 776 | SLC25A4 |
| 777 | ABCC8 | 778 | FMO3 | 779 | CXCR3 | 780 | MOGS |
| 781 | SOCS1 | 782 | PEX26 | 783 | SNHG3 | 784 | MFN2 |
| 785 | MIR9-2 | 786 | IL1RN | 787 | DYNC2H1 | 788 | ETFB |
| 789 | PPOX | 790 | LOC129997612 | 791 | BANCR | 792 | UGT1A8 |
| 793 | LNCBRM | 794 | IFT56 | 795 | PTGS2 | 796 | FBLN5 |
| 797 | HLA-A | 798 | ZFYVE19 | 799 | EPAS1 | 800 | EFEMP2 |
| 801 | AOC4P | 802 | CCEPR | 803 | ACTA2-AS1 | 804 | PEX19 |
| 805 | EHHADH | 806 | PDP1 | 807 | CD8A | 808 | HSPD1 |
| 809 | NDUFS1 | 810 | FTX | 811 | FABP5P3 | 812 | SQSTM1 |
| 813 | WT1-AS | 814 | UFC1 | 815 | SOX2-OT | 816 | MAPK1 |
| 817 | MIR124-2 | 818 | GJB1 | 819 | CDKN1B | 820 | HOXA-AS2 |
| 821 | CPS1-IT1 | 822 | SLCO1A2 | 823 | MBOAT7 | 824 | LCN2 |
| 825 | MDM2 | 826 | ZEB1-AS1 | 827 | TK2 | 828 | VIPAS39 |
| 829 | TRIM37 | 830 | PCAT29 | 831 | CDKN1A | 832 | AIRE |
| 833 | CASR | 834 | FTL | 835 | TGFA | 836 | UGT1A10 |
| 837 | KCTD13 | 838 | MIRLET7A3 | 839 | TLR3 | 840 | CASC2 |
| 841 | FGF21 | 842 | SGSH | 843 | MIR181C | 844 | PEX10 |
| 845 | TUSC7 | 846 | TCTN2 | 847 | GLT1D1 | 848 | CASC11 |
| 849 | GAS5-AS1 | 850 | ARL13B | 851 | PIK3R1 | 852 | ARSB |
| 853 | DRAIC | 854 | HGD | 855 | FAM13A | 856 | LINC01138 |
| 857 | PEX12 | 858 | SMAD2 | 859 | ETFA | 860 | CIDEC |
| 861 | EGILA | 862 | PDX1 | 863 | MIRLET7A2 | 864 | FN1 |
| 865 | GFOD3P | 866 | FGF2 | 867 | SHOC2 | 868 | ACTA2 |
| 869 | KRTAP5-AS1 | 870 | SEMA6A-AS1 | 871 | KLF1 | 872 | COG6 |
| 873 | SLC25A27 | 874 | HEIH | 875 | BAIAP2-DT | 876 | CTH |
| 877 | CTSD | 878 | MAN2B1 | 879 | EGFR-AS1 | 880 | FAM83A-AS1 |
| 881 | ENPP1 | 882 | MLXIPL | 883 | NUP210 | 884 | TMEM231 |
| 885 | HBA2 | 886 | PRECSIT | 887 | SERPINA3 | 888 | BBS5 |
| 889 | MIR181B2 | 890 | BMPR2 | 891 | C20orf204 | 892 | VIM |
| 893 | GALNS | 894 | TFRC | 895 | FER1L4 | 896 | CCL5 |
| 897 | SUCLA2 | 898 | TKT | 899 | PROM1 | 900 | ACTC1 |
| 901 | GCLC | 902 | ZNF674-AS1 | 903 | NKILA | 904 | BBS7 |
| 905 | TNFSF11 | 906 | ACOX2 | 907 | LINC02027 | 908 | TMEM237 |
| 909 | LIG4 | 910 | NNT-AS1 | 911 | ZEB2-AS1 | 912 | AHCY |
| 913 | CARD8-AS1 | 914 | LPIN1 | 915 | CAV3 | 916 | CYP3A5 |
| 917 | CASP3 | 918 | SHBG | 919 | GATA6 | 920 | ICAM4-AS1 |
| 921 | MSH2 | 922 | CFH | 923 | TMEM51-AS1 | 924 | RAB4B-EGLN2 |
| 925 | PEPD | 926 | GHET1 | 927 | IFIH1 | 928 | LINC00601 |
| 929 | BBS12 | 930 | PTENP1 | 931 | FLT1 | 932 | LINC00589 |
| 933 | LINC00173 | 934 | CHI3L1 | 935 | ACACA | 936 | TERC |
| 937 | NR2F1-AS1 | 938 | BBS9 | 939 | JPX | 940 | PDHA1 |
| 941 | NGLY1 | 942 | PDIA3P1 | 943 | ARC | 944 | C14orf132 |
| 945 | PNP | 946 | MAPKAPK5-AS1 | 947 | VLDLR-AS1 | 948 | ASGR1 |
| 949 | GJA1 | 950 | F9 | 951 | CXCL1P1 | 952 | FBXL4 |
| 953 | XIAP | 954 | CECR7 | 955 | PRAL | 956 | SDCCAG8 |
| 957 | HTR2A-AS1 | 958 | NPM1 | 959 | ZNF350-AS1 | 960 | BLVRA |
| 961 | GIHCG | 962 | RARA | 963 | SLC39A4 | 964 | GPC3-AS1 |
| 965 | CCND3P1 | 966 | MLH1 | 967 | DIP2C-AS1 | 968 | IQCB1 |
| 969 | SEC23B | 970 | MIR146B | 971 | BBS10 | 972 | VMA22 |
| 973 | RANBP2 | 974 | LDC1P | 975 | LINC00974 | 976 | LINC02055 |
| 977 | OVCH1-AS1 | 978 | ULK4P2 | 979 | IATPR | 980 | CYCS |
| 981 | CD44 | 982 | ABCC6 | 983 | MT-CYB | 984 | CA3-AS1 |
| 985 | SUMF1 | 986 | PCNA-AS1 | 987 | TGFBR1 | 988 | CEL |
| 989 | SHH | 990 | PRKCD | 991 | TPMT | 992 | LINC00554 |
| 993 | SCD | 994 | PRC1-AS1 | 995 | HBA1 | 996 | SLC7A9 |
| 997 | SERHL | 998 | TGFB2 | 999 | SLC2A4 | 1000 | PGK1 |
| 1001 | G6PC3 | 1002 | ADK | 1003 | PIK3CG | 1004 | PEX3 |
| 1005 | MIRLET7F2 | 1006 | MIR1-2 | 1007 | HMGCS2 | 1008 | TCTN1 |
| 1009 | THBD | 1010 | COQ2 | 1011 | GLIS3 | 1012 | UGT1A3 |
| 1013 | STAT2 | 1014 | IFNGR1 | 1015 | MAT2A | 1016 | CDK4 |
| 1017 | DDIT3 | 1018 | PTCH1 | 1019 | NARF-AS1 | 1020 | SIRT1-AS |
| 1021 | CDR1 | 1022 | GNPTAB | 1023 | FGA | 1024 | PCCA |
| 1025 | ANGPTL3 | 1026 | IKBKB | 1027 | THY1-AS1 | 1028 | CERNA2 |
| 1029 | AHI1 | 1030 | ADM | 1031 | MCL1 | 1032 | VCAM1 |
| 1033 | IKBKG | 1034 | GALE | 1035 | LGALS3 | 1036 | LTF |
| 1037 | LINC00383 | 1038 | TCTN3 | 1039 | ITCH | 1040 | B9D2 |
| 1041 | MAPK14 | 1042 | SREBF2 | 1043 | ENPP2 | 1044 | MAN1B1 |
| 1045 | DPM1 | 1046 | CCL20 | 1047 | NFKBIA | 1048 | EDNRA |
| 1049 | IL2RB | 1050 | BIRC5 | 1051 | LINC00210 | 1052 | GCG |
| 1053 | PSMB9 | 1054 | GCDH | 1055 | TNFSF10 | 1056 | NHLRC1 |
| 1057 | TMEM138 | 1058 | GPD1 | 1059 | HSPA5 | 1060 | SAMHD1 |
| 1061 | GATA1 | 1062 | AQP2 | 1063 | GHRL | 1064 | ZMPSTE24 |
| 1065 | NOTCH3 | 1066 | CD81 | 1067 | MYCN | 1068 | CD58 |
| 1069 | SERPINA6 | 1070 | HGSNAT | 1071 | PSMB8 | 1072 | STEAP3 |
| 1073 | DPYS | 1074 | ZIC3 | 1075 | DNAH8 | 1076 | VCP |
| 1077 | ENO1 | 1078 | UPB1 | 1079 | ABCD3 | 1080 | PEX7 |
| 1081 | MKI67 | 1082 | LINC02605 | 1083 | PON1 | 1084 | MIR192 |
| 1085 | KCNJ11 | 1086 | NAGA | 1087 | MTR | 1088 | SERPINF2 |
| 1089 | SCO1 | 1090 | BCHE | 1091 | SMAD7 | 1092 | PKM |
| 1093 | GDF15 | 1094 | CBL | 1095 | IRF3 | 1096 | HPX |
| 1097 | FADD | 1098 | ABCC1 | 1099 | MMP7 | 1100 | CYP3A7 |
| 1101 | C3 | 1102 | LOC113687175 | 1103 | SEMA4D | 1104 | SUOX |
| 1105 | STXBP2 | 1106 | EPHX2 | 1107 | UGT2B7 | 1108 | GNS |
| 1109 | TMEM70 | 1110 | SCO2 | 1111 | EZH2 | 1112 | KIAA0586 |
| 1113 | OGDH | 1114 | LOC126805688 | 1115 | MMP3 | 1116 | CEACAM5 |
| 1117 | ATP6AP2 | 1118 | ATP6AP1 | 1119 | LGALS3BP | 1120 | STAT4 |
| 1121 | GZMB | 1122 | GFM1 | 1123 | HMGB1 | 1124 | BRCA1 |
| 1125 | AHR | 1126 | TLR9 | 1127 | IFNAR1 | 1128 | RASSF1 |
| 1129 | SPG7 | 1130 | AR | 1131 | ALG11 | 1132 | CREBBP |
| 1133 | IGF1R | 1134 | ACTB | 1135 | ALG2 | 1136 | BCKDHB |
| 1137 | CHUK | 1138 | PLAU | 1139 | AVP | 1140 | NAF1 |
| 1141 | POLGARF | 1142 | SLC35C1 | 1143 | COG4 | 1144 | CIITA |
| 1145 | GPR35 | 1146 | PDHB | 1147 | ERCC1 | 1148 | SERAC1 |
| 1149 | IL7R | 1150 | PROX1 | 1151 | APOA5 | 1152 | PCCB |
| 1153 | RNASEH2C | 1154 | KCNN4 | 1155 | CD274 | 1156 | STAT5B |
| 1157 | SCARB2 | 1158 | HSPA4 | 1159 | BID | 1160 | SLCO2B1 |
| 1161 | LECT2 | 1162 | PML | 1163 | HHEX | 1164 | PRF1 |
| 1165 | RLBP1 | 1166 | MIR149 | 1167 | MIR424 | 1168 | CA2 |
| 1169 | ENO3 | 1170 | PCBD1 | 1171 | GYG2 | 1172 | MEN1 |
| 1173 | ACADL | 1174 | EPM2A | 1175 | SP1 | 1176 | B4GALT1 |
| 1177 | COL1A1 | 1178 | ALG1 | 1179 | RGN | 1180 | PTPN3 |
| 1181 | GJB2 | 1182 | SURF1 | 1183 | RELA | 1184 | MMAA |
| 1185 | MCCC2 | 1186 | TINF2 | 1187 | CDKN2B | 1188 | PDGFB |
| 1189 | EP300 | 1190 | SLC46A1 | 1191 | TTC7A | 1192 | ABCG2 |
| 1193 | POU5F1 | 1194 | AOX1 | 1195 | MKKS | 1196 | CYP1B1 |
| 1197 | GLRX5 | 1198 | GSK3B | 1199 | CPLANE1 | 1200 | ZNF423 |
| 1201 | SP100 | 1202 | MIR373 | 1203 | GC | 1204 | ELANE |
| 1205 | PRKACA | 1206 | RTEL1 | 1207 | GAMT | 1208 | BCL2L1 |
| 1209 | RBPJ | 1210 | AQP1 | 1211 | LCA5 | 1212 | CYP19A1 |
| 1213 | LMBRD1 | 1214 | CYP8B1 | 1215 | ALG6 | 1216 | LTA |
| 1217 | TPI1 | 1218 | NPY | 1219 | SMAD6 | 1220 | CDK2 |
| 1221 | VTN | 1222 | NF2 | 1223 | NQO1 | 1224 | IL12RB2 |
| 1225 | RARB | 1226 | MYH9 | 1227 | TMPRSS6 | 1228 | LPIN2 |
| 1229 | SCTR | 1230 | COQ9 | 1231 | ECHS1 | 1232 | MMAB |
| 1233 | C4A | 1234 | VMA12 | 1235 | KIF7 | 1236 | ATP8B1-AS1 |
| 1237 | MAPK3 | 1238 | DNMT1 | 1239 | AUH | 1240 | RNASEH2B |
| 1241 | TNFRSF1B | 1242 | DDOST | 1243 | PLAT | 1244 | CTC1 |
| 1245 | HLA-C | 1246 | DES | 1247 | ABCD1 | 1248 | PRTN3 |
| 1249 | CXCL9 | 1250 | ACVR2B | 1251 | IVD | 1252 | GH1 |
| 1253 | EIF2AK3 | 1254 | DGAT1 | 1255 | IRF1 | 1256 | GATM |
| 1257 | MGMT | 1258 | SLC22A1 | 1259 | GSTA1 | 1260 | LOC114803475 |
| 1261 | NLRP3 | 1262 | GNPAT | 1263 | MME | 1264 | IGFBP2 |
| 1265 | GIMAP5 | 1266 | IL6ST | 1267 | EIF2S1 | 1268 | FGL1 |
| 1269 | CTSA | 1270 | CYP17A1 | 1271 | PCSK1 | 1272 | OAT |
| 1273 | ALG12 | 1274 | SDHB | 1275 | EIF2AK2 | 1276 | ACAT1 |
| 1277 | CDKN1C | 1278 | ITGAL | 1279 | GALC | 1280 | SLC25A3 |
| 1281 | TMEM165 | 1282 | GATA2 | 1283 | DKC1 | 1284 | IRS2 |
| 1285 | MIR210 | 1286 | GATA4 | 1287 | ANGPT1 | 1288 | KIF23 |
| 1289 | ATF4 | 1290 | PGM3 | 1291 | BMPR1A | 1292 | OPA3 |
| 1293 | OPA1 | 1294 | HSP90AA1 | 1295 | LSR | 1296 | APOH |
| 1297 | PLAUR | 1298 | APOL1 | 1299 | BBIP1 | 1300 | TAFAZZIN |
| 1301 | LEFTY2 | 1302 | CREB3L3 | 1303 | CALM2 | 1304 | DOLK |
| 1305 | DNMT3B | 1306 | BMP4 | 1307 | PHYH | 1308 | TNFAIP3 |
| 1309 | ADIPOR2 | 1310 | GHR | 1311 | FOXA3 | 1312 | DNMT3A |
| 1313 | ACSF3 | 1314 | PGAM2 | 1315 | ATP7A | 1316 | MYB |
| 1317 | LYST | 1318 | HAVCR2 | 1319 | SDHC | 1320 | SDHD |
| 1321 | PRKAA2 | 1322 | CPT1B | 1323 | SPINT2 | 1324 | DNAL1 |
| 1325 | FOXA1 | 1326 | MIR34C | 1327 | ANGPT2 | 1328 | AGA |
| 1329 | APOC2 | 1330 | RSF1 | 1331 | RNASEH2A | 1332 | CEP41 |
| 1333 | FABP6 | 1334 | ELMOD2 | 1335 | BCKDHA | 1336 | RNF220 |
| 1337 | PGR-AS1 | 1338 | GCKR | 1339 | ST6GAL1 | 1340 | AREG |
| 1341 | EPHB4 | 1342 | HES1 | 1343 | CLEC4M | 1344 | MMACHC |
| 1345 | PIK3CD | 1346 | CREB1 | 1347 | WNT4 | 1348 | RUNX1 |
| 1349 | DPM3 | 1350 | SRD5A3 | 1351 | UNC45A | 1352 | LIPT1 |
| 1353 | CD40 | 1354 | PTF1A | 1355 | PIGA | 1356 | COG7 |
| 1357 | ALG3 | 1358 | NNMT | 1359 | FOXRED1 | 1360 | IARS1 |
| 1361 | KLF6 | 1362 | SNAI2 | 1363 | F7 | 1364 | BTK |
| 1365 | IFNB1 | 1366 | TCF7L2 | 1367 | CCR2 | 1368 | PIGR |
| 1369 | KLKB1 | 1370 | MICA | 1371 | XRCC1 | 1372 | MSH6 |
| 1373 | COX5A | 1374 | SLC5A1 | 1375 | COL4A3 | 1376 | GLDC |
| 1377 | MT-CO1 | 1378 | TULP3 | 1379 | RYR1 | 1380 | SLC16A1 |
| 1381 | MIR1225 | 1382 | CST3 | 1383 | HEXB | 1384 | MPL |
| 1385 | SALL4 | 1386 | CDAN1 | 1387 | EDNRB | 1388 | SPINK1 |
| 1389 | RPGRIP1 | 1390 | PPARD | 1391 | THBS1 | 1392 | BCR |
| 1393 | HLCS | 1394 | PODXL | 1395 | ADAM10 | 1396 | SERPINF1 |
| 1397 | GSTM3 | 1398 | PKD1L1 | 1399 | AURKA | 1400 | CNR1 |
| 1401 | SELP | 1402 | MT-CO3 | 1403 | SOS1 | 1404 | FOXO3 |
| 1405 | NME1 | 1406 | NKX2-1 | 1407 | TNFRSF10A | 1408 | UGT1A5 |
| 1409 | DPAGT1 | 1410 | NRP1 | 1411 | GCH1 | 1412 | VCL |
| 1413 | TSFM | 1414 | FABP2 | 1415 | COG8 | 1416 | SUN2 |
| 1417 | CYP2C8 | 1418 | HSD11B1 | 1419 | CX3CR1 | 1420 | SNAI1 |
| 1421 | ONECUT2 | 1422 | ABL1 | 1423 | ACACB | 1424 | ARL6 |
| 1425 | UTS2 | 1426 | DHDDS | 1427 | AXIN2 | 1428 | ERBB3 |
| 1429 | EIF2AK4 | 1430 | MYD88 | 1431 | SELL | 1432 | EYA1 |
| 1433 | MED12 | 1434 | AKR1C4 | 1435 | PTK2 | 1436 | AP1S1 |
| 1437 | STX5 | 1438 | PRDX5 | 1439 | TSPO | 1440 | APTX |
| 1441 | SP140 | 1442 | FCGR2A | 1443 | SOAT2 | 1444 | EDAR |
| 1445 | CLU | 1446 | CTSB | 1447 | PDSS2 | 1448 | MT-TL1 |
| 1449 | TTC8 | 1450 | COG1 | 1451 | ATP6V0A2 | 1452 | TIMP3 |
| 1453 | ERCC4 | 1454 | TFF3 | 1455 | LCT | 1456 | GLI1 |
| 1457 | MMP13 | 1458 | RAG2 | 1459 | RPGR | 1460 | SERPINB3 |
| 1461 | EGLN1 | 1462 | NOP10 | 1463 | GPAM | 1464 | SUGCT |
| 1465 | TRIM32 | 1466 | COG5 | 1467 | PAX8 | 1468 | HYAL1 |
| 1469 | KITLG | 1470 | DMD | 1471 | CCNA2 | 1472 | CDH3 |
| 1473 | EFNB2 | 1474 | CSF3 | 1475 | MT-ND1 | 1476 | COQ6 |
| 1477 | YBX1 | 1478 | SETD2 | 1479 | SELE | 1480 | IL15 |
| 1481 | PARN | 1482 | CYBB | 1483 | DNASE2 | 1484 | CD80 |
| 1485 | MIR9-1 | 1486 | MIR193B | 1487 | JUP | 1488 | PGR |
| 1489 | MIR98 | 1490 | DGAT2 | 1491 | TNFRSF10B | 1492 | PPT1 |
| 1493 | TNFRSF11B | 1494 | UMPS | 1495 | MCCC1 | 1496 | BAK1 |
| 1497 | COL3A1 | 1498 | ID2 | 1499 | NPHS2 | 1500 | TGIF1 |
| 1501 | BSG | 1502 | RHOA | 1503 | FXYD2 | 1504 | TOP2A |
| 1505 | UNC13D | 1506 | PAX2 | 1507 | AGK | 1508 | SERPINA7 |
| 1509 | MYL2 | 1510 | TXN | 1511 | IRF7 | 1512 | IFNL4 |
| 1513 | HEXA | 1514 | SLC3A1 | 1515 | MIR193A | 1516 | TEK |
| 1517 | CLPB | 1518 | DVL1 | 1519 | LMF1 | 1520 | UGT2B4 |
| 1521 | BIRC3 | 1522 | COL4A4 | 1523 | FGB | 1524 | ACTN4 |
| 1525 | PARP1 | 1526 | MEFV | 1527 | MIR33B | 1528 | IL10RB |
| 1529 | CHKA | 1530 | SLC12A1 | 1531 | SNORD15A | 1532 | SLC39A14 |
| 1533 | FLAD1 | 1534 | KLB | 1535 | EGR1 | 1536 | IL5 |
| 1537 | CCR6 | 1538 | ANPEP | 1539 | PECAM1 | 1540 | SLC35A2 |
| 1541 | SARDH | 1542 | WNT1 | 1543 | EPOR | 1544 | CHD7 |
| 1545 | EBP | 1546 | ODC1 | 1547 | SAR1B | 1548 | AKT3 |
| 1549 | FANCC | 1550 | PHKA2-AS1 | 1551 | HSD17B10 | 1552 | HSPA1A |
| 1553 | QDPR | 1554 | IKZF1 | 1555 | TAP1 | 1556 | WNT5A |
| 1557 | TNFRSF6B | 1558 | NDUFB9 | 1559 | SDHA | 1560 | SYP |
| 1561 | AOC3 | 1562 | SPTB | 1563 | PINX1 | 1564 | SLC39A8 |
| 1565 | GREM1 | 1566 | SALL1 | 1567 | BTNL2 | 1568 | ANXA2 |
| 1569 | ITGAV | 1570 | PRKAG3 | 1571 | MIR6766 | 1572 | NDUFS4 |
| 1573 | MTRR | 1574 | ARID2 | 1575 | SMARCB1 | 1576 | MAP3K5 |
| 1577 | NPPB | 1578 | FARSA | 1579 | NT5C3A | 1580 | CLN3 |
| 1581 | MANBA | 1582 | NOS1 | 1583 | CLN6 | 1584 | SAA1 |
| 1585 | CASP9 | 1586 | WFS1 | 1587 | MTAP | 1588 | CTSK |
| 1589 | IL1R1 | 1590 | FHIT | 1591 | CALM1 | 1592 | CEACAM6 |
| 1593 | MIR15B | 1594 | SGPL1 | 1595 | CNP | 1596 | ATP4A |
| 1597 | MIR130B | 1598 | DMBT1 | 1599 | CHEK2 | 1600 | CCL3 |
| 1601 | CDK1 | 1602 | ATF6 | 1603 | HSPB1 | 1604 | MMP14 |
| 1605 | TET2 | 1606 | IL18BP | 1607 | NDUFAF2 | 1608 | COL18A1 |
| 1609 | EDN3 | 1610 | RUNX3 | 1611 | OSM | 1612 | TP73 |
| 1613 | CDH2 | 1614 | PHGDH | 1615 | CHD4 | 1616 | ABCG1 |
| 1617 | ANO5 | 1618 | AQP9 | 1619 | TTPA | 1620 | ELAVL1 |
| 1621 | SLC25A1 | 1622 | ACVR1 | 1623 | SDC1 | 1624 | NHP2 |
| 1625 | VIPR1 | 1626 | CCNE1 | 1627 | GRB2 | 1628 | TWIST1 |
| 1629 | CD27 | 1630 | LOC129934096 | 1631 | DYSF | 1632 | AAAS |
| 1633 | LDLRAP1 | 1634 | FLNB | 1635 | NPHS1 | 1636 | ACADSB |
| 1637 | CSF1R | 1638 | PTS | 1639 | MTRFR | 1640 | NDUFV1 |
| 1641 | LOX | 1642 | PRKCA | 1643 | APOA2 | 1644 | CEACAM1 |
| 1645 | HSP90B1 | 1646 | TIMM8A | 1647 | SPTA1 | 1648 | PNKD |
| 1649 | NFS1 | 1650 | HGFAC | 1651 | FGG | 1652 | MCOLN1 |
| 1653 | ANK1 | 1654 | NPC1L1 | 1655 | STX11 | 1656 | CYLD |
| 1657 | CCNB1 | 1658 | PIK3CB | 1659 | DNM1L | 1660 | ACY1 |
| 1661 | RAG1 | 1662 | VEGFC | 1663 | BSND | 1664 | AGPS |
| 1665 | SOX9 | 1666 | CTNNA1 | 1667 | PPP1CB | 1668 | SMARCAL1 |
| 1669 | ETHE1 | 1670 | AMFR | 1671 | PRKN | 1672 | PTPN1 |
| 1673 | DCTN4 | 1674 | BDNF-AS | 1675 | CD86 | 1676 | FST |
| 1677 | NOTCH4 | 1678 | CLCNKB | 1679 | CDK6 | 1680 | BECN1 |
| 1681 | HDAC1 | 1682 | HPRT1 | 1683 | IL13 | 1684 | MUTYH |
| 1685 | IREB2 | 1686 | AFG3L2 | 1687 | IFT80 | 1688 | OXA1L |
| 1689 | DNASE1L3 | 1690 | DGUOK-AS1 | 1691 | FLT4 | 1692 | PRKG1 |
| 1693 | BCL6 | 1694 | ADSL | 1695 | ETS1 | 1696 | SLC35A1 |
| 1697 | RPL11 | 1698 | SULT1A1 | 1699 | TRAPPC11 | 1700 | KNG1 |
| 1701 | CCL4 | 1702 | SLC2A10 | 1703 | SOAT1 | 1704 | ALDH5A1 |
| 1705 | POLD1 | 1706 | ADRB2 | 1707 | WDPCP | 1708 | IL2RG |
| 1709 | HBG2 | 1710 | FLT3 | 1711 | TIMMDC1 | 1712 | OCLN |
| 1713 | AMT | 1714 | IFT43 | 1715 | CASP1 | 1716 | SLC22A7 |
| 1717 | ALG13 | 1718 | IL22 | 1719 | TPP1 | 1720 | FKRP |
| 1721 | SSTR2 | 1722 | COMT | 1723 | SLC12A3 | 1724 | FREM2 |
| 1725 | DNAJB1 | 1726 | IFT74 | 1727 | ASXL1 | 1728 | NODAL |
| 1729 | WDR83OS | 1730 | COL4A2 | 1731 | FGF1 | 1732 | CDC42 |
| 1733 | CTSC | 1734 | KCNH1 | 1735 | DPM2 | 1736 | KCNQ1 |
| 1737 | GSTZ1 | 1738 | E2F1 | 1739 | SFTPC | 1740 | PTPN22 |
| 1741 | MAGT1 | 1742 | ITGB3 | 1743 | F2R | 1744 | RPL5 |
| 1745 | CD1D | 1746 | FBXW7 | 1747 | REG3A | 1748 | CLCN5 |
| 1749 | MOCS2 | 1750 | LOC126806400 | 1751 | OGG1 | 1752 | MT-ATP6 |
| 1753 | PEBP1 | 1754 | WRN | 1755 | RAB27A | 1756 | MRPS7 |
| 1757 | CD163 | 1758 | SERPING1 | 1759 | SHC1 | 1760 | YARS2 |
| 1761 | LPA | 1762 | KCNJ1 | 1763 | PON2 | 1764 | ATIC |
| 1765 | ISCU | 1766 | NKX2-5 | 1767 | HCFC1 | 1768 | CYBA |
| 1769 | PALB2 | 1770 | CHGA | 1771 | IRF8 | 1772 | ACAD8 |
| 1773 | MT-CO2 | 1774 | CD28 | 1775 | ITPA | 1776 | LAMA2 |
| 1777 | MDK | 1778 | TAB2 | 1779 | LEPQTL1 | 1780 | FTCD |
| 1781 | RPS6KB1 | 1782 | COQ4 | 1783 | EZR | 1784 | SLC9A3 |
| 1785 | ADAMTSL2 | 1786 | TRPC6 | 1787 | MX1 | 1788 | COL4A5 |
| 1789 | MGAT3 | 1790 | SARS2 | 1791 | COPA | 1792 | AKR1B10 |
| 1793 | PARK7 | 1794 | ITGA6 | 1795 | PRPS1 | 1796 | APRT |
| 1797 | CBLIF | 1798 | DBI | 1799 | ABCB6 | 1800 | PANK2 |
| 1801 | ACTA1 | 1802 | ITGA3 | 1803 | NCOA3 | 1804 | NAMPT |
| 1805 | TYK2 | 1806 | L2HGDH | 1807 | HIBCH | 1808 | MIR376A1 |
| 1809 | TMPO | 1810 | BIRC2 | 1811 | STT3A | 1812 | BPI |
| 1813 | COQ5 | 1814 | MYH11 | 1815 | KLRK1 | 1816 | PRKD1 |
| 1817 | CCR1 | 1818 | KLHL12 | 1819 | ANO10 | 1820 | AVPR2 |
| 1821 | COL2A1 | 1822 | EARS2 | 1823 | MLYCD | 1824 | TKFC |
| 1825 | CTNS | 1826 | ARNT | 1827 | SOX4 | 1828 | IGF2BP2 |
| 1829 | MIR144 | 1830 | EEF1A1 | 1831 | GRN | 1832 | GSR |
| 1833 | ERCC2 | 1834 | CKS1B | 1835 | TCN2 | 1836 | SKP2 |
| 1837 | AKR1C2 | 1838 | TBX20 | 1839 | CD2AP | 1840 | AP1B1 |
| 1841 | PON3 | 1842 | ILK | 1843 | PI4KA | 1844 | FURIN |
| 1845 | ANXA5 | 1846 | PRKDC | 1847 | CDIN1 | 1848 | DKK1 |
| 1849 | ATXN2 | 1850 | GABRG2 | 1851 | RPLP0 | 1852 | STX1A |
| 1853 | MPDU1 | 1854 | BLM | 1855 | CTCF | 1856 | COG2 |
| 1857 | ALDH7A1 | 1858 | SOS2 | 1859 | SOX17 | 1860 | PLA2G6 |
| 1861 | FGF20 | 1862 | HDAC9 | 1863 | YY1 | 1864 | DYM |
| 1865 | PRKAR1A | 1866 | ITGAM | 1867 | A2M | 1868 | DHODH |
| 1869 | MIR204 | 1870 | ASPA | 1871 | ANGPTL8 | 1872 | IFT88 |
| 1873 | KCNJ10 | 1874 | ZEB2 | 1875 | VPS33A | 1876 | SPEN |
| 1877 | CCR7 | 1878 | POMC | 1879 | DDX3X | 1880 | GDF1 |
| 1881 | THRB | 1882 | ALDH3A2 | 1883 | DLST | 1884 | IRAK4 |
| 1885 | PTMA | 1886 | PGD | 1887 | MIR423 | 1888 | PSMD10 |
| 1889 | AMN | 1890 | DYNC2LI1 | 1891 | HBEGF | 1892 | MMP11 |
| 1893 | KMT2D | 1894 | DLK1 | 1895 | IQGAP1 | 1896 | SC5D |
| 1897 | MIR370 | 1898 | MTA1 | 1899 | MOCOS | 1900 | SLC38A3 |
| 1901 | USP18 | 1902 | TLN1 | 1903 | FLVCR1 | 1904 | DUSP1 |
| 1905 | HK2 | 1906 | NRIR | 1907 | NOX4 | 1908 | CTSG |
| 1909 | AKR1C1 | 1910 | SSR4 | 1911 | KRIT1 | 1912 | SEMA7A |
| 1913 | CSF1 | 1914 | CUBN | 1915 | D2HGDH | 1916 | RPS24 |
| 1917 | SRF | 1918 | FABP4 | 1919 | MIR335 | 1920 | XRCC6 |
| 1921 | DNM2 | 1922 | FAS-AS1 | 1923 | SLC4A1 | 1924 | PCYT1A |
| 1925 | FABP12 | 1926 | DNAH5 | 1927 | PRDX1 | 1928 | IFT27 |
| 1929 | GAST | 1930 | MMADHC | 1931 | TBX1 | 1932 | HLA-DPB1 |
| 1933 | PDSS1 | 1934 | SPTBN1 | 1935 | LMNB2 | 1936 | IL32 |
| 1937 | SIX2 | 1938 | SGCB | 1939 | CHIT1 | 1940 | SULT1E1 |
| 1941 | TAP2 | 1942 | NR2F2 | 1943 | GMPPA | 1944 | TBC1D4 |
| 1945 | ACLY | 1946 | LTBR | 1947 | SLC26A1 | 1948 | BCL2L11 |
| 1949 | SH2B3 | 1950 | SERPINB2 | 1951 | RUNX2 | 1952 | MUC16 |
| 1953 | FANCD2 | 1954 | HIC1 | 1955 | STT3B | 1956 | HPSE |
| 1957 | MT-ND5 | 1958 | MIR92A1 | 1959 | IDO1 | 1960 | PIM2 |
| 1961 | RPS19 | 1962 | ERCC6 | 1963 | AARS1 | 1964 | RIGI |
| 1965 | SBDS | 1966 | CLEC4G | 1967 | MIR574 | 1968 | GPHN |
| 1969 | POFUT1 | 1970 | PTP4A1 | 1971 | HLA-G | 1972 | PIN1 |
| 1973 | SEC61B | 1974 | ABCD4 | 1975 | PIK3C2A | 1976 | COX15 |
| 1977 | SLC67A1 | 1978 | PTTG1 | 1979 | RFT1 | 1980 | BMI1 |
| 1981 | GOT1 | 1982 | PRL | 1983 | TMEM126A | 1984 | GCSH |
| 1985 | ANG | 1986 | IFI27 | 1987 | UCN | 1988 | BTRC |
| 1989 | HMGA1 | 1990 | MIRLET7I | 1991 | LMNB1 | 1992 | MOCS1 |
| 1993 | HDGF | 1994 | RAC1 | 1995 | APOBEC1 | 1996 | COQ7 |
| 1997 | CHEK1 | 1998 | PRKCI | 1999 | MCEE | 2000 | NSD1 |
| 2001 | AMPD1 | 2002 | SERPINA10 | 2003 | ID1 | 2004 | KIF3B |
| 2005 | UAP1 | 2006 | MAGEA4 | 2007 | MIR486-1 | 2008 | LIG3 |
| 2009 | CLPX | 2010 | MAOA | 2011 | MIR511 | 2012 | NARS2 |
| 2013 | CLN8 | 2014 | XPC | 2015 | DDC | 2016 | SERPINA12 |
| 2017 | ERCC5 | 2018 | TH | 2019 | TXNIP | 2020 | GJA5 |
| 2021 | MICB | 2022 | CENPB | 2023 | MB | 2024 | GPX4 |
| 2025 | ITGA5 | 2026 | THY1 | 2027 | SP3 | 2028 | EPHA2 |
| 2029 | LDHB | 2030 | CAD | 2031 | CFLAR | 2032 | CCN1 |
| 2033 | ORM1 | 2034 | LZTFL1 | 2035 | STMN1 | 2036 | MT-ND3 |
| 2037 | ACTG1 | 2038 | CD47 | 2039 | RPS10 | 2040 | MVP |
| 2041 | CD82 | 2042 | LOC112449713 | 2043 | ADCY10 | 2044 | JUND |
| 2045 | SOCS2 | 2046 | CSF2 | 2047 | DICER1 | 2048 | UGP2 |
| 2049 | SRSF2 | 2050 | RRAS | 2051 | AK2 | 2052 | MMP12 |
| 2053 | PRKCB | 2054 | TGFB3 | 2055 | ICOS | 2056 | SCNN1A |
| 2057 | RBL2 | 2058 | LAMP1 | 2059 | BAD | 2060 | UQCRB |
| 2061 | NCF2 | 2062 | KHSRP | 2063 | PTK2B | 2064 | GLI3 |
| 2065 | SP110 | 2066 | GM2A | 2067 | SH2D1A | 2068 | CCDC40 |
| 2069 | MGAT5 | 2070 | ORM2 | 2071 | PYGB | 2072 | GFAP |
| 2073 | RORC | 2074 | MIR342 | 2075 | BLVRB | 2076 | DARS2 |
| 2077 | MIR24-1 | 2078 | TRAF6 | 2079 | CCK | 2080 | CDKN2C |
| 2081 | NDUFAF5 | 2082 | TRIM21 | 2083 | MIR455 | 2084 | MT-ND2 |
| 2085 | C5 | 2086 | COQ8A | 2087 | USF1 | 2088 | PPP1R3C |
| 2089 | PRKAB1 | 2090 | RECK | 2091 | ITGA8 | 2092 | EEF1A2 |
| 2093 | AADAC | 2094 | ACSL4 | 2095 | FCAR | 2096 | MTDH |
| 2097 | CDX2 | 2098 | RPS26 | 2099 | PPP2R1B | 2100 | NADK2 |
| 2101 | TFAP2A | 2102 | SFRP1 | 2103 | FOLR1 | 2104 | ZFPM2 |
| 2105 | CCN4 | 2106 | AURKB | 2107 | CCL16 | 2108 | ITIH4 |
| 2109 | ADIPOR1 | 2110 | EGLN3 | 2111 | MYOT | 2112 | PSMB4 |
| 2113 | UQCRC2 | 2114 | PLK1 | 2115 | OXCT1 | 2116 | TJP1 |
| 2117 | FDX2 | 2118 | HAX1 | 2119 | EVC2 | 2120 | UPK2 |
| 2121 | CDH13 | 2122 | PUS1 | 2123 | GFPT1 | 2124 | ATP13A2 |
| 2125 | BOLA3 | 2126 | MIR532 | 2127 | ERCC3 | 2128 | ALAS1 |
| 2129 | SHARPIN | 2130 | MIR499A | 2131 | SIRPA | 2132 | LIPG |
| 2133 | CACNA1S | 2134 | RHD | 2135 | CA5A | 2136 | ACO1 |
| 2137 | RBM10 | 2138 | KRT5 | 2139 | RXRB | 2140 | CAPN2 |
| 2141 | DOCK6 | 2142 | RAC2 | 2143 | DIABLO | 2144 | HSPG2 |
| 2145 | TMC4 | 2146 | GNPTG | 2147 | GMPPB | 2148 | MAT2B |
| 2149 | CSK | 2150 | ARHGAP31 | 2151 | IMMT | 2152 | CD151 |
| 2153 | C1S | 2154 | S100A8 | 2155 | MAPK9 | 2156 | IL11 |
| 2157 | PRDX6 | 2158 | APOC1 | 2159 | PSMA7 | 2160 | PROC |
| 2161 | FANCA | 2162 | NEUROG3 | 2163 | LOXL2 | 2164 | RARRES2 |
| 2165 | CLCA4 | 2166 | SLC6A8 | 2167 | PNPLA6 | 2168 | SLC3A2 |
| 2169 | FOSL1 | 2170 | MAPK11 | 2171 | CXCL2 | 2172 | MIR106A |
| 2173 | MIR124-1 | 2174 | TUSC3 | 2175 | IKZF3 | 2176 | KIF1B |
| 2177 | MIR30B | 2178 | RARS1 | 2179 | NAT1 | 2180 | LGALS1 |
| 2181 | NPPC | 2182 | S100A9 | 2183 | PARS2 | 2184 | ITGA1 |
| 2185 | ATG5 | 2186 | REG1A | 2187 | SCN4A | 2188 | MIR29B1 |
| 2189 | MMP8 | 2190 | SLC19A3 | 2191 | PRKAA1 | 2192 | SYK |
| 2193 | TANGO2 | 2194 | LGR5 | 2195 | SELENOP | 2196 | RIPK1 |
| 2197 | RARS2 | 2198 | SOX10 | 2199 | MIR345 | 2200 | CANX |
| 2201 | MIR32 | 2202 | PTPA | 2203 | PNPO | 2204 | MFSD8 |
| 2205 | POGLUT1 | 2206 | HDAC2 | 2207 | ATOX1 | 2208 | CR2 |
| 2209 | TNNI3 | 2210 | GALM | 2211 | FCN2 | 2212 | CD209 |
| 2213 | ANXA1 | 2214 | CSTB | 2215 | MIR101-1 | 2216 | TPM1 |
| 2217 | FANCG | 2218 | C1QBP | 2219 | TLL1 | 2220 | RDX |
| 2221 | EIF6 | 2222 | SRD5A2 | 2223 | HTATIP2 | 2224 | C4B |
| 2225 | CXCR2 | 2226 | TFEB | 2227 | MIR320A | 2228 | RBBP4 |
| 2229 | BCAR1 | 2230 | CADM1 | 2231 | HHIP | 2232 | NHLRC2 |
| 2233 | MAGEA1 | 2234 | FARS2 | 2235 | MMP15 | 2236 | CFHR3 |
| 2237 | RPS6 | 2238 | ALDH3A1 | 2239 | PIGM | 2240 | IGHE |
| 2241 | PIM1 | 2242 | PSC | 2243 | MT-ND4 | 2244 | BCL10 |
| 2245 | ATRIP | 2246 | MIR100 | 2247 | DDB1 | 2248 | DSP |
| 2249 | MIR615 | 2250 | SSB | 2251 | TBX5 | 2252 | CYP51A1 |
| 2253 | CD63 | 2254 | ABCC11 | 2255 | EIF4G2 | 2256 | ASPH |
| 2257 | H6PD | 2258 | ADH1A | 2259 | SPG11 | 2260 | ABCC5 |
| 2261 | LOC106627981 | 2262 | CSE1L | 2263 | CLCN1 | 2264 | CCL21 |
| 2265 | IL3 | 2266 | PRDX3 | 2267 | TFCP2 | 2268 | FGF7 |
| 2269 | DLL4 | 2270 | PXN | 2271 | TNXB | 2272 | MRC1 |
| 2273 | CD68 | 2274 | SLC26A9 | 2275 | BRIP1 | 2276 | MIR196A1 |
| 2277 | NEK2 | 2278 | PITX2 | 2279 | LARS2 | 2280 | LAMA4 |
| 2281 | TNFRSF9 | 2282 | APEX1 | 2283 | SLC26A3 | 2284 | KISS1 |
| 2285 | EPB42 | 2286 | GSS | 2287 | P4HB | 2288 | CES3 |
| 2289 | NCF4 | 2290 | EDEM1 | 2291 | FADS1 | 2292 | CXCL1 |
| 2293 | MIR128-1 | 2294 | LPCAT3 | 2295 | MIR377 | 2296 | MERTK |
| 2297 | MIR10A | 2298 | CDK13 | 2299 | CCNH | 2300 | BCL11A |
| 2301 | FXN | 2302 | CDC6 | 2303 | MIR28 | 2304 | HLA-DRB5 |
| 2305 | BMPR1B | 2306 | KHK | 2307 | F8 | 2308 | FOXH1 |
| 2309 | LIAS | 2310 | MT1G | 2311 | CLN5 | 2312 | NSMCE2 |
| 2313 | HBG1 | 2314 | TLR7 | 2315 | EMD | 2316 | PSMC4 |
| 2317 | CD55 | 2318 | WHRN | 2319 | DENND1B | 2320 | LRP1 |
| 2321 | RBP1 | 2322 | MIR542 | 2323 | TRPV1 | 2324 | XPR1 |
| 2325 | FLNC | 2326 | FKTN | 2327 | IL33 | 2328 | RAD51B |
| 2329 | GLO1 | 2330 | CDK12 | 2331 | EFTUD2 | 2332 | ERN1 |
| 2333 | TRPV4 | 2334 | EPRS1 | 2335 | STAT5A | 2336 | SLC22A4 |
| 2337 | XPO1 | 2338 | NCAM1 | 2339 | DNAH11 | 2340 | MED1 |
| 2341 | LOC106560211 | 2342 | HSPA8 | 2343 | MITF | 2344 | HBD |
| 2345 | ATP5F1A | 2346 | SMAD9 | 2347 | TM4SF5 | 2348 | PRDM2 |
| 2349 | COL5A1 | 2350 | MIR133A1 | 2351 | CD19 | 2352 | CYP11A1 |
| 2353 | CTTN | 2354 | PEG10 | 2355 | PRIM1 | 2356 | TULP1 |
| 2357 | ERCC8 | 2358 | MAP3K1 | 2359 | TPX2 | 2360 | SGK1 |
| 2361 | COPS5 | 2362 | MIR513A1 | 2363 | IL9 | 2364 | DOCK8 |
| 2365 | MIR211 | 2366 | STC1 | 2367 | CR1 | 2368 | APOD |
| 2369 | SIAH1 | 2370 | BAG3 | 2371 | VIP | 2372 | NFKB2 |
| 2373 | F13A1 | 2374 | FUBP1 | 2375 | LAMA5 | 2376 | FAM111A |
| 2377 | ESR2 | 2378 | CARS2 | 2379 | LYRM4 | 2380 | SERPINI1 |
| 2381 | PPAT | 2382 | KEAP1 | 2383 | FANCF | 2384 | MECP2 |
| 2385 | MIR505 | 2386 | TNFRSF25 | 2387 | MTARC1 | 2388 | ERFE |
| 2389 | SLC39A5 | 2390 | HACD1 | 2391 | CCNG1 | 2392 | AIP |
| 2393 | CFAP418 | 2394 | MIR302A | 2395 | SERPINA2 | 2396 | MGAT2 |
| 2397 | SEPTIN9 | 2398 | TRIB1 | 2399 | ITGB2 | 2400 | ADAM17 |
| 2401 | BUB1 | 2402 | MIR331 | 2403 | MIR885 | 2404 | VKORC1 |
| 2405 | MST1R | 2406 | IFT81 | 2407 | XPA | 2408 | SLC6A14 |
| 2409 | SLC47A1 | 2410 | BNIP3 | 2411 | STX3 | 2412 | EIF2B4 |
| 2413 | CCKBR | 2414 | NFE2L1 | 2415 | NDUFA13 | 2416 | S100A4 |
| 2417 | ARHGAP1 | 2418 | PSKH1 | 2419 | BLZF1 | 2420 | HNRNPH1 |
| 2421 | OSTM1 | 2422 | CEP19 | 2423 | KMT2A | 2424 | AZGP1 |
| 2425 | POMT1 | 2426 | PRKCZ | 2427 | ADRB3 | 2428 | RPS17 |
| 2429 | GPIHBP1 | 2430 | MT-ND6 | 2431 | RELN | 2432 | MAPK12 |
| 2433 | AGER | 2434 | MIR339 | 2435 | ENTPD2 | 2436 | APOA4 |
| 2437 | ACKR2 | 2438 | VDAC1 | 2439 | CDK9 | 2440 | KAAG1 |
| 2441 | CRELD1 | 2442 | NR3C2 | 2443 | MMP10 | 2444 | PLCG1 |
| 2445 | MIR425 | 2446 | COX4I2 | 2447 | ETS2 | 2448 | SLC2A3 |
| 2449 | AMBP | 2450 | HABP2 | 2451 | SERPINB1 | 2452 | RHBDF2 |
| 2453 | TNFRSF11A | 2454 | COLEC10 | 2455 | PTPN13 | 2456 | MIR135A1 |
| 2457 | MDH2 | 2458 | PCSK5 | 2459 | ACD | 2460 | NEK9 |
| 2461 | TSG101 | 2462 | POMGNT2 | 2463 | ING1 | 2464 | MIR501 |
| 2465 | SDHAF1 | 2466 | PLAAT3 | 2467 | SLC25A10 | 2468 | TMEM43 |
| 2469 | ADCY6 | 2470 | DCK | 2471 | GRB10 | 2472 | CASP10 |
| 2473 | ANXA10 | 2474 | RNF31 | 2475 | ADH4 | 2476 | MIR99B |
| 2477 | FA2H | 2478 | TRMT5 | 2479 | TRADD | 2480 | PDE4A |
| 2481 | RTN4 | 2482 | FANCL | 2483 | TG | 2484 | TTC19 |
| 2485 | CISH | 2486 | ACP5 | 2487 | BCOR | 2488 | COMMD1 |
| 2489 | FCGRT | 2490 | CTSL | 2491 | TXNDC15 | 2492 | CYP4F2 |
| 2493 | OGT | 2494 | RPS7 | 2495 | TEP1 | 2496 | RING1 |
| 2497 | RMRP | 2498 | RASA1 | 2499 | LTBP3 | 2500 | PRKACG |
| 2501 | SLC27A4 | 2502 | KRT14 | 2503 | CPEB4 | 2504 | NFU1 |
| 2505 | FDFT1 | 2506 | CYBRD1 | 2507 | PROS1 | 2508 | FAP |
| 2509 | LDB3 | 2510 | POMT2 | 2511 | PMM1 | 2512 | SLC26A4 |
| 2513 | RPL3 | 2514 | PRODH | 2515 | CTNND1 | 2516 | SOST |
| 2517 | MIR506 | 2518 | DAPK1 | 2519 | PPM1F | 2520 | TRPM6 |
| 2521 | PDE5A | 2522 | DNAAF2 | 2523 | ABCC10 | 2524 | CKB |
| 2525 | SKP1 | 2526 | CCDC39 | 2527 | TCN1 | 2528 | STING1 |
| 2529 | AZIN1 | 2530 | SFRP5 | 2531 | PDZK1 | 2532 | RPS6KA3 |
| 2533 | WWOX | 2534 | PYCARD | 2535 | ROCK1 | 2536 | OPTN |
| 2537 | CD320 | 2538 | SNHG7 | 2539 | RAI1 | 2540 | MIR590 |
| 2541 | B3GLCT | 2542 | CFB | 2543 | UGGT1 | 2544 | TYR |
| 2545 | PSEN1 | 2546 | VIL1 | 2547 | SPINT1 | 2548 | ATP6V1E1 |
| 2549 | NHERF1 | 2550 | HNRNPA1 | 2551 | PRDM1 | 2552 | CAV2 |
| 2553 | SMG1 | 2554 | PSMG2 | 2555 | FUT8 | 2556 | GIMAP1-GIMAP5 |
| 2557 | CCT2 | 2558 | XRCC5 | 2559 | STAT6 | 2560 | RPL35A |
| 2561 | RNF125 | 2562 | PSMA3 | 2563 | DVL3 | 2564 | MIP |
| 2565 | SMYD3 | 2566 | GATA5 | 2567 | GSDMB | 2568 | ACP1 |
| 2569 | RNPC3 | 2570 | SLC19A2 | 2571 | PXDN | 2572 | AQP11 |
| 2573 | SLC1A5 | 2574 | CALM3 | 2575 | MIR379 | 2576 | PCBP1 |
| 2577 | RHOC | 2578 | ATP5F1E | 2579 | S100B | 2580 | ATP11A |
| 2581 | DDB2 | 2582 | TFPI2 | 2583 | LACTB | 2584 | ANTXR2 |
| 2585 | DRD2 | 2586 | MIR503 | 2587 | ABCA12 | 2588 | CFC1 |
| 2589 | HAVCR1 | 2590 | LYVE1 | 2591 | UPF1 | 2592 | PGF |
| 2593 | ZMYND10 | 2594 | RUVBL2 | 2595 | CDC25C | 2596 | RAD51 |
| 2597 | NGFR | 2598 | SERPINH1 | 2599 | IHH | 2600 | SEC14L2 |
| 2601 | CTNNBIP1 | 2602 | FBL | 2603 | MIR675 | 2604 | PAK1 |
| 2605 | MAN2C1 | 2606 | ESD | 2607 | DNASE1 | 2608 | ENO2 |
| 2609 | NPLOC4 | 2610 | AOC1 | 2611 | CHRM3 | 2612 | ERLIN1 |
| 2613 | F10 | 2614 | KCNJ2 | 2615 | MAN2A1 | 2616 | EOGT |
| 2617 | ENTPD1 | 2618 | DMXL2 | 2619 | USH2A | 2620 | TLR5 |
| 2621 | SPP2 | 2622 | CDIPT | 2623 | MIR625 | 2624 | MAGEA3 |
| 2625 | ADAM9 | 2626 | FNDC5 | 2627 | SLC25A26 | 2628 | ATR |
| 2629 | CTSS | 2630 | TPO | 2631 | NAT8 | 2632 | STBD1 |
| 2633 | LOC129933372 | 2634 | PRDX4 | 2635 | LOC107133510 | 2636 | KL |
| 2637 | ABCA3 | 2638 | BNIPL | 2639 | RFX6 | 2640 | IL17RA |
| 2641 | PRLR | 2642 | NRF1 | 2643 | SERPINE2 | 2644 | ITGA2 |
| 2645 | MIR18B | 2646 | DNAAF1 | 2647 | FANCE | 2648 | MTO1 |
| 2649 | MAD2L1 | 2650 | PWAR1 | 2651 | CUL7 | 2652 | ADRA2A |
| 2653 | C1R | 2654 | NCOA1 | 2655 | TREH | 2656 | SLC6A9 |
| 2657 | COASY | 2658 | DNAI1 | 2659 | TAGLN | 2660 | TNFRSF8 |
| 2661 | CHMP5 | 2662 | ISG15 | 2663 | SULF1 | 2664 | RHCE |
| 2665 | ALCAM | 2666 | APOBEC3G | 2667 | KAT2B | 2668 | GAL3ST1 |
| 2669 | PDLIM3 | 2670 | TNFRSF13B | 2671 | TNFSF13B | 2672 | TBK1 |
| 2673 | GSN | 2674 | PTPRO | 2675 | MRPL3 | 2676 | ITPR3 |
| 2677 | GLI2 | 2678 | FAF2 | 2679 | FANCM | 2680 | SSBP1 |
| 2681 | FBLN2 | 2682 | POSTN | 2683 | GLP1R | 2684 | LOC112272621 |
| 2685 | CD59 | 2686 | RECQL4 | 2687 | MIR495 | 2688 | TRIM63 |
| 2689 | MYH6 | 2690 | NDUFB11 | 2691 | SPAG1 | 2692 | CFHR1 |
| 2693 | ARID1B | 2694 | HM13 | 2695 | BCL2L2 | 2696 | MIR660 |
| 2697 | MYH3 | 2698 | SETX | 2699 | IFNGR2 | 2700 | TBX3 |
| 2701 | STAB1 | 2702 | REL | 2703 | MUC2 | 2704 | LAPTM4B |
| 2705 | HRG | 2706 | CCL19 | 2707 | EXOSC3 | 2708 | HEPH |
| 2709 | MIR491 | 2710 | STAB2 | 2711 | G6PC2 | 2712 | SERPINB5 |
| 2713 | MIR103A1 | 2714 | MIR340 | 2715 | IFNAR2-IL10RB | 2716 | EXO1 |
| 2717 | PANK1 | 2718 | DLGAP5 | 2719 | SLC6A19 | 2720 | DEK |
| 2721 | RNASE3 | 2722 | MIR188 | 2723 | AGPAT1 | 2724 | OAS1 |
| 2725 | PYCR1 | 2726 | VPS11 | 2727 | MT-ATP8 | 2728 | HNRNPDL |
| 2729 | NPHP3-ACAD11 | 2730 | SPHK1 | 2731 | IL7 | 2732 | PIEZO1 |
| 2733 | FGF18 | 2734 | GOT2 | 2735 | SFTPD | 2736 | MSRA |
| 2737 | IL10RA | 2738 | CNR2 | 2739 | VPS13B | 2740 | NISCH |
| 2741 | RAB8A | 2742 | ETV6 | 2743 | ADAMTS17 | 2744 | SCNN1B |
| 2745 | CYC1 | 2746 | GAS8 | 2747 | NXF1 | 2748 | IL12B |
| 2749 | MIR19B1 | 2750 | CFAP298 | 2751 | FGF3 | 2752 | GALNT2 |
| 2753 | HSP90AB1 | 2754 | STUB1 | 2755 | KRT1 | 2756 | HSPA9 |
| 2757 | B3GAT3 | 2758 | COL6A2 | 2759 | MYBL2 | 2760 | GBA3 |
| 2761 | KIR3DL1 | 2762 | CNTNAP2 | 2763 | CA7 | 2764 | POT1 |
| 2765 | HLA-E | 2766 | MMP16 | 2767 | SEC16A | 2768 | LYRM7 |
| 2769 | EPG5 | 2770 | LIN28B | 2771 | MYO1B | 2772 | FGF4 |
| 2773 | WWTR1 | 2774 | CLDN7 | 2775 | SKI | 2776 | HLA-DRA |
| 2777 | AKR1B1 | 2778 | SDR9C7 | 2779 | GATAD1 | 2780 | SPTAN1 |
| 2781 | PALLD | 2782 | AICDA | 2783 | MYL3 | 2784 | PITRM1 |
| 2785 | RAD51C | 2786 | LASP1 | 2787 | NECTIN2 | 2788 | MYO7A |
| 2789 | ACOX3 | 2790 | RPS27A | 2791 | DNAAF11 | 2792 | SMN1 |
| 2793 | STN1 | 2794 | NUMB | 2795 | MIR129-1 | 2796 | E2F4 |
| 2797 | FGGY | 2798 | CDC25B | 2799 | E2F3 | 2800 | LRBA |
| 2801 | CALB2 | 2802 | LOC130009838 | 2803 | HDGFL2 | 2804 | AP2M1 |
| 2805 | LAMB3 | 2806 | WIF1 | 2807 | HSD3B2 | 2808 | RACK1 |
| 2809 | VEGFB | 2810 | HIRA | 2811 | CNNM2 | 2812 | CACNA1C |
| 2813 | MIR545 | 2814 | MIR502 | 2815 | STAP1 | 2816 | SLC25A37 |
| 2817 | KLF2 | 2818 | MIR493 | 2819 | ITK | 2820 | WNT3 |
| 2821 | DNAAF5 | 2822 | CTF1 | 2823 | HOXA1 | 2824 | KCNA5 |
| 2825 | NKRF | 2826 | NUP62 | 2827 | LAP3 | 2828 | DUOX2 |
| 2829 | SPARC | 2830 | SLC30A2 | 2831 | MIR541 | 2832 | TP53BP1 |
| 2833 | APOM | 2834 | SLC35D1 | 2835 | ZCCHC8 | 2836 | RTEL1-TNFRSF6B |
| 2837 | YBX3 | 2838 | INSL6 | 2839 | CYP2R1 | 2840 | SLC6A3 |
| 2841 | FNDC3B | 2842 | MAVS | 2843 | ORMDL3 | 2844 | PSMA4 |
| 2845 | FGL2 | 2846 | FOXF1 | 2847 | SIAH2 | 2848 | MFAP5 |
| 2849 | NT5E | 2850 | TTC28 | 2851 | PLPP6 | 2852 | YWHAE |
| 2853 | MIR1228 | 2854 | PDCD1LG2 | 2855 | NEDD9 | 2856 | RNF7 |
| 2857 | CACNA2D1 | 2858 | U2AF1 | 2859 | CLDN16 | 2860 | LOC126859653 |
| 2861 | CABIN1 | 2862 | LOC126862761 | 2863 | CPB2 | 2864 | CTAG1B |
| 2865 | TAL1 | 2866 | RPS20 | 2867 | BARD1 | 2868 | NOL11 |
| 2869 | KCNK3 | 2870 | TMBIM6 | 2871 | SNCA | 2872 | CDH5 |
| 2873 | ATRIP-TREX1 | 2874 | PMS1 | 2875 | CAPN10 | 2876 | MARCKS |
| 2877 | NLRP1 | 2878 | DPP9 | 2879 | SHROOM3 | 2880 | SOX2 |
| 2881 | PHB2 | 2882 | SERPINA4 | 2883 | USP9X | 2884 | ATP11C |
| 2885 | XRCC4 | 2886 | HLF | 2887 | LOC126861110 | 2888 | SLC25A17 |
| 2889 | MYH7 | 2890 | MAOB | 2891 | HBS1L | 2892 | MASP1 |
| 2893 | MGST1 | 2894 | FAT4 | 2895 | C2 | 2896 | FHL2 |
| 2897 | EBAG9 | 2898 | HNRNPUL1 | 2899 | TRA-TGC7-1 | 2900 | NIPA2 |
| 2901 | CBX5 | 2902 | LOC111674472 | 2903 | CXADR | 2904 | SSTR1 |
| 2905 | PTX3 | 2906 | MIR20B | 2907 | CENPF | 2908 | MADCAM1 |
| 2909 | TFAP2B | 2910 | TMEM30A | 2911 | HSD11B2 | 2912 | ABCB8 |
| 2913 | MTF1 | 2914 | EXOC3L4 | 2915 | MIR363 | 2916 | MAF |
| 2917 | SLC5A9 | 2918 | PTH1R | 2919 | GNLY | 2920 | KTN1 |
| 2921 | TSHR | 2922 | EPHA1 | 2923 | SIRT3 | 2924 | QARS1 |
| 2925 | FANCB | 2926 | ATP1A1 | 2927 | PRKACB | 2928 | TGFBR3 |
| 2929 | AGXT2 | 2930 | EXT2 | 2931 | HNRNPA2B1 | 2932 | MIR92B |
| 2933 | WNT7B | 2934 | MIR134 | 2935 | GRK2 | 2936 | MSI1 |
| 2937 | SLC39A13 | 2938 | MIR372 | 2939 | SUCLG2 | 2940 | MIR485 |
| 2941 | HRH1 | 2942 | TBX4 | 2943 | ARG2 | 2944 | PSMD4 |
| 2945 | HAND1 | 2946 | MIR190A | 2947 | CYP21A2 | 2948 | MEIS2 |
| 2949 | LGALS9 | 2950 | BCL3 | 2951 | MIR138-1 | 2952 | UCP1 |
| 2953 | UGDH | 2954 | LHX1 | 2955 | ACVR2A | 2956 | MIR181D |
| 2957 | SAR1A | 2958 | FMR1 | 2959 | UCK2 | 2960 | CCNC |
| 2961 | SLCO4A1 | 2962 | KDELR1 | 2963 | DNAAF3 | 2964 | MIA2 |
| 2965 | COX10 | 2966 | USB1 | 2967 | RO60 | 2968 | PHKG1 |
| 2969 | NR1D1 | 2970 | CCBE1 | 2971 | FZD7 | 2972 | TRE-TTC3-1 |
| 2973 | MRPS16 | 2974 | AFM | 2975 | SIRT6 | 2976 | PPP1R15B |
| 2977 | CUTA | 2978 | GP2 | 2979 | TARDBP | 2980 | EDA |
| 2981 | SEC24C | 2982 | BMP10 | 2983 | ITIH2 | 2984 | PDLIM5 |
| 2985 | CFTR-AS1 | 2986 | DLG5 | 2987 | ITGA4 | 2988 | EHBP1L1 |
| 2989 | HYOU1 | 2990 | NKX2-6 | 2991 | MIR766 | 2992 | ITGA2B |
| 2993 | RELB | 2994 | APLN | 2995 | KDM6A | 2996 | HMOX2 |
| 2997 | CNNM4 | 2998 | ABCB10 | 2999 | SFRP2 | 3000 | SRP54 |
| 3001 | IQGAP2 | 3002 | KIR2DL2 | 3003 | MIR202 | 3004 | SPRED1 |
| 3005 | CRYAB | 3006 | ZEB1 | 3007 | TGM2 | 3008 | WNT5B |
| 3009 | DAND5 | 3010 | GCLM | 3011 | PHB1 | 3012 | ALDH16A1 |
| 3013 | CCDC65 | 3014 | RPS15 | 3015 | SLC4A4 | 3016 | TSBP1 |
| 3017 | CXCL11 | 3018 | NANOG | 3019 | MIR524 | 3020 | MT2A |
| 3021 | CXCR5 | 3022 | PSMB10 | 3023 | KCNA1 | 3024 | PSMD9 |
| 3025 | MRTFA | 3026 | GBA2 | 3027 | ANGPTL4 | 3028 | SERPIND1 |
| 3029 | MIR628 | 3030 | SFRP4 | 3031 | CSNK2B | 3032 | RDH12 |
| 3033 | MAN1A1 | 3034 | PF4 | 3035 | ADPRH | 3036 | ODAD1 |
| 3037 | PDGFA | 3038 | DNAJC21 | 3039 | MIR433 | 3040 | MYO5A |
| 3041 | SNHG22 | 3042 | NLRC4 | 3043 | PAX3 | 3044 | BRD2 |
| 3045 | ALPI | 3046 | F11 | 3047 | ALS2 | 3048 | TNFSF12 |
| 3049 | MSLN | 3050 | EPB41 | 3051 | DNAH1 | 3052 | FCGR2B |
| 3053 | SLX4 | 3054 | MCFD2 | 3055 | IL16 | 3056 | BMAL1 |
| 3057 | PLEC | 3058 | TMSB4X | 3059 | CLPP | 3060 | ATG16L1 |
| 3061 | SULF2 | 3062 | NRTN | 3063 | INSIG1 | 3064 | COL11A2 |
| 3065 | SLC25A28 | 3066 | VPS4A | 3067 | TRIT1 | 3068 | CS |
| 3069 | MIR382 | 3070 | ITPR2 | 3071 | MELTF | 3072 | PDIA3 |
| 3073 | SERPINA11 | 3074 | BMP1 | 3075 | HKDC1 | 3076 | SFTPB |
| 3077 | TRB | 3078 | ESCO2 | 3079 | ATP8B3 | 3080 | VAPB |
| 3081 | PTPN2 | 3082 | MIR302C | 3083 | SESN2 | 3084 | MR1 |
| 3085 | UGCG | 3086 | LAG3 | 3087 | PDPN | 3088 | UBR1 |
| 3089 | CLDN19 | 3090 | SUFU | 3091 | MIRLET7F1 | 3092 | NLK |
| 3093 | MDC1 | 3094 | CDR1-AS | 3095 | CLTC | 3096 | PRKAG1 |
| 3097 | PSMA1 | 3098 | RIPK3 | 3099 | DBH | 3100 | DOCK7 |
| 3101 | FTO | 3102 | MUC6 | 3103 | COL14A1 | 3104 | CGAS |
| 3105 | IL4R | 3106 | MGME1 | 3107 | MAP1LC3A | 3108 | CCL11 |
| 3109 | FGF23 | 3110 | MUC5AC | 3111 | PZP | 3112 | ST3GAL3 |
| 3113 | NAA15 | 3114 | RSPH3 | 3115 | HLA-DPA1 | 3116 | KIF3A |
| 3117 | MT1E | 3118 | KCNJ5 | 3119 | ZBTB24 | 3120 | CD276 |
| 3121 | WNT3A | 3122 | NEFH | 3123 | TSEN34 | 3124 | MIR375 |
| 3125 | FBXL5 | 3126 | LAMC2 | 3127 | UBE2D1 | 3128 | SCARNA5 |
| 3129 | CHAT | 3130 | EFL1 | 3131 | PLCL2 | 3132 | DCTN1 |
| 3133 | MIR1247 | 3134 | AKAP13 | 3135 | IMPDH2 | 3136 | MSTN |
| 3137 | GPC1 | 3138 | CLIC1 | 3139 | ATP12A | 3140 | RNF5 |
| 3141 | ATF5 | 3142 | CDX1 | 3143 | HYDIN | 3144 | FGF10 |
| 3145 | CCL15-CCL14 | 3146 | ACE2 | 3147 | MCM2 | 3148 | ADAMTS2 |
| 3149 | FCGR3A | 3150 | NGF | 3151 | MIR873 | 3152 | ID3 |
| 3153 | PGPEP1 | 3154 | LOC106099062 | 3155 | RSPH1 | 3156 | GGT2P |
| 3157 | MUC3A | 3158 | BRD4 | 3159 | SCAP | 3160 | MYH14 |
| 3161 | DCN | 3162 | CHMP2B | 3163 | CFP | 3164 | CSF3R |
| 3165 | IL23A | 3166 | IL17F | 3167 | DDX5 | 3168 | DDX39B |
| 3169 | PDGFC | 3170 | ATP8B2 | 3171 | MASP2 | 3172 | BGN |
| 3173 | STEAP1 | 3174 | TRC-GCA24-1 | 3175 | PCSK6 | 3176 | SMARCA2 |
| 3177 | EXOC4 | 3178 | RPA1 | 3179 | MLPH | 3180 | GPX1 |
| 3181 | EIF4E | 3182 | NOP2 | 3183 | DNAAF4 | 3184 | RHOD |
| 3185 | MIR371A | 3186 | PSTPIP1 | 3187 | THBS2 | 3188 | CSTA |
| 3189 | SRSF1 | 3190 | COL6A3 | 3191 | MIR330 | 3192 | MIR362 |
| 3193 | GHRH | 3194 | SOX18 | 3195 | TNFRSF13C | 3196 | MIR876 |
| 3197 | ACO2 | 3198 | CXCR6 | 3199 | WEE1 | 3200 | HK3 |
| 3201 | GYPA | 3202 | MIR365A | 3203 | PRKAB2 | 3204 | LOC111674475 |
| 3205 | MIR135B | 3206 | PRDX2 | 3207 | ODAD2 | 3208 | ECE1 |
| 3209 | CYP39A1 | 3210 | MIR1307 | 3211 | HEMGN | 3212 | DAG1 |
| 3213 | RSPH4A | 3214 | LOC113664106 | 3215 | TNNC1 | 3216 | PIGL |
| 3217 | LTBP1 | 3218 | MIR490 | 3219 | YWHAZ | 3220 | CAST |
| 3221 | PDXK | 3222 | ACAA1 | 3223 | LOC108663984 | 3224 | NIPAL4 |
| 3225 | MIR942 | 3226 | JAG2 | 3227 | LOC110006319 | 3228 | OTULIN |
| 3229 | MIR1207 | 3230 | VASH1 | 3231 | XYLT1 | 3232 | NCL |
| 3233 | MBD4 | 3234 | CSAD | 3235 | CCNO | 3236 | ZFPM1 |
| 3237 | IGKC | 3238 | ADORA2A | 3239 | CCS | 3240 | COL1A2 |
| 3241 | HNRNPC | 3242 | EIF4EBP1 | 3243 | EEF2 | 3244 | CDH23 |
| 3245 | SLC22A3 | 3246 | RPLP1 | 3247 | LSM11 | 3248 | SLC30A5 |
| 3249 | SYVN1 | 3250 | CXCR1 | 3251 | PFAS | 3252 | TOR1A |
| 3253 | BCL9L | 3254 | ADD3 | 3255 | MUC5B | 3256 | CITED2 |
| 3257 | DHCR24 | 3258 | HELLS | 3259 | NES | 3260 | PLA2G2A |
| 3261 | PIAS1 | 3262 | SLC7A2 | 3263 | NEK3 | 3264 | DCLK1 |
| 3265 | CDC37 | 3266 | PCSK7 | 3267 | RSPH9 | 3268 | DNAJC5 |
| 3269 | DUSP19 | 3270 | PRSS1 | 3271 | ZHX2 | 3272 | CYP4F22 |
| 3273 | UNG | 3274 | PLIN5 | 3275 | PROP1 | 3276 | ELMO1 |
| 3277 | UQCC2 | 3278 | SFTPA2 | 3279 | DNAI2 | 3280 | CBR1 |
| 3281 | MSH5 | 3282 | CCL27 | 3283 | APEH | 3284 | MIR452 |
| 3285 | EFEMP1 | 3286 | ACTN1 | 3287 | UBE2L3 | 3288 | TGM1 |
| 3289 | MGLL | 3290 | RPS14 | 3291 | MIR769 | 3292 | TGDS |
| 3293 | CAPZA2 | 3294 | LHX2 | 3295 | MIR7-1 | 3296 | APP |
| 3297 | PFKFB3 | 3298 | TXN2 | 3299 | MIR652 | 3300 | CD38 |
| 3301 | CDO1 | 3302 | MAP2K4 | 3303 | COX17 | 3304 | TRMT10A |
| 3305 | ABCF1 | 3306 | ENHO | 3307 | HNRNPK | 3308 | GET3 |
| 3309 | CKMT2 | 3310 | MAP1LC3B | 3311 | SLC29A4 | 3312 | CSNK2A1 |
| 3313 | CLDN10 | 3314 | ABHD10 | 3315 | HOXA10 | 3316 | FUT2 |
| 3317 | FIP1L1 | 3318 | PRNP | 3319 | TMEM259 | 3320 | LAMTOR5 |
| 3321 | C2orf69 | 3322 | ADH5 | 3323 | ACHE | 3324 | FSCN1 |
| 3325 | TXNRD1 | 3326 | GDNF | 3327 | PPBP | 3328 | S1PR2 |
| 3329 | ATP2B4 | 3330 | DDAH2 | 3331 | G0S2 | 3332 | ACBD6 |
| 3333 | SRRT | 3334 | MIR582 | 3335 | PAX6 | 3336 | AQP3 |
| 3337 | ADAM12 | 3338 | PTGS1 | 3339 | MIR105-1 | 3340 | PPP1R12A |
| 3341 | ARRB1 | 3342 | NFATC1 | 3343 | GADD45A | 3344 | DEPDC5 |
| 3345 | RPS11 | 3346 | NOP53 | 3347 | ZNF469 | 3348 | TRU-TCA1-1 |
| 3349 | CCAR2 | 3350 | HSPB2 | 3351 | FYN | 3352 | LOC110806306 |
| 3353 | CAMP | 3354 | ADCY3 | 3355 | LOC102723566 | 3356 | OPRM1 |
| 3357 | MIR877 | 3358 | ALOX12B | 3359 | SOX11 | 3360 | AMDHD1 |
| 3361 | MIR216B | 3362 | MIR584 | 3363 | KIR2DL3 | 3364 | DLL1 |
| 3365 | CBLB | 3366 | TMEM176A | 3367 | SLC6A4 | 3368 | C2CD3 |
| 3369 | LOC129930433 | 3370 | PIGT | 3371 | IL1RL1 | 3372 | IGES |
| 3373 | PLOD2 | 3374 | DRC1 | 3375 | MAGEC2 | 3376 | SMARCE1 |
| 3377 | BDNF | 3378 | HTRA1 | 3379 | KLF4 | 3380 | MAPK10 |
| 3381 | IGF2BP1 | 3382 | UQCRQ | 3383 | CIDEB | 3384 | LEMD3 |
| 3385 | ADAMTS10 | 3386 | NME8 | 3387 | NELFA | 3388 | HPS4 |
| 3389 | RPS25 | 3390 | MAP3K7 | 3391 | MIR671 | 3392 | TFPI |
| 3393 | AKAP1 | 3394 | POLH | 3395 | PPA1 | 3396 | LOC110366354 |
| 3397 | GTPBP1 | 3398 | ATP6V1A | 3399 | DNAAF19 | 3400 | CARMIL2 |
| 3401 | TERF2 | 3402 | TREM1 | 3403 | UNC13A | 3404 | TMBIM4 |
| 3405 | IL1RAPL2 | 3406 | CA4 | 3407 | CKM | 3408 | ZNF142 |
| 3409 | PIPOX | 3410 | ESM1 | 3411 | RNASEH1 | 3412 | XK |
| 3413 | ITGB6 | 3414 | LOC111674477 | 3415 | SLC33A1 | 3416 | LMAN1 |
| 3417 | CCM2 | 3418 | SNRPA | 3419 | TCAP | 3420 | PDPK1 |
| 3421 | PARVB | 3422 | ATF3 | 3423 | FUT6 | 3424 | TECRL |
| 3425 | MYO1A | 3426 | UTP6 | 3427 | SAMM50 | 3428 | MS4A1 |
| 3429 | APBB1 | 3430 | SUGP1 | 3431 | VEGFD | 3432 | MIR432 |
| 3433 | IDE | 3434 | CKMT1B | 3435 | GPAT3 | 3436 | AIPL1 |
| 3437 | HCP5 | 3438 | PAX5 | 3439 | PCNT | 3440 | PRIMPOL |
| 3441 | DDR1 | 3442 | FAM161A | 3443 | TGFBI | 3444 | DACT1 |
| 3445 | RPLP2 | 3446 | FKBP8 | 3447 | FEN1 | 3448 | TUBB3 |
| 3449 | KCTD7 | 3450 | TAB1 | 3451 | CXCL5 | 3452 | APCS |
| 3453 | SPATA7 | 3454 | DEFB1 | 3455 | CFL1 | 3456 | CELF1 |
| 3457 | CLEC16A | 3458 | CXCL16 | 3459 | CYGB | 3460 | MESP2 |
| 3461 | ST3GAL5 | 3462 | MIR589 | 3463 | HEY1 | 3464 | CRH |
| 3465 | CD5L | 3466 | SLC30A6 | 3467 | PSAT1 | 3468 | PSMD12 |
| 3469 | ARCN1 | 3470 | AOAH | 3471 | NEDD4L | 3472 | MIR1236 |
| 3473 | ABCB9 | 3474 | NTS | 3475 | NME4 | 3476 | DNAAF6 |
| 3477 | MYOZ2 | 3478 | ACVR1B | 3479 | KRT17 | 3480 | RBM7 |
| 3481 | ATP2A2 | 3482 | PSMD8 | 3483 | SFN | 3484 | ITGB4 |
| 3485 | ADH7 | 3486 | IYD | 3487 | XPNPEP1 | 3488 | FHOD3 |
| 3489 | GOPC | 3490 | EXOSC2 | 3491 | RHAG | 3492 | XRCC2 |
| 3493 | PPP1R3A | 3494 | DTNA | 3495 | GLMP | 3496 | ARPC2 |
| 3497 | DNA2 | 3498 | STS | 3499 | ALOX5 | 3500 | LAMA3 |
| 3501 | SPTLC1 | 3502 | MAPT | 3503 | PNLIP | 3504 | LONP1 |
| 3505 | PABPC1 | 3506 | SLC30A9 | 3507 | PLCB3 | 3508 | DNAJA1 |
| 3509 | CCR9 | 3510 | SSTR3 | 3511 | PIK3C3 | 3512 | BPGM |
| 3513 | NFIC | 3514 | EDEM3 | 3515 | RPS18 | 3516 | TSPAN2 |
| 3517 | SMC3 | 3518 | MSN | 3519 | GUCY2D | 3520 | IL1RAP |
| 3521 | HSF1 | 3522 | PLA2G12B | 3523 | KLK3 | 3524 | DHDH |
| 3525 | FIG4 | 3526 | IGF2BP3 | 3527 | PSMB3 | 3528 | NCOA4 |
| 3529 | SNORD44 | 3530 | NDRG2 | 3531 | MIR218-1 | 3532 | SLC1A2 |
| 3533 | GTF2I | 3534 | TTF2 | 3535 | MAML1 | 3536 | P2RX7 |
| 3537 | TRIM5 | 3538 | TTC36 | 3539 | GAL | 3540 | IFT20 |
| 3541 | CUL1 | 3542 | MIR767 | 3543 | DISP1 | 3544 | NEIL2 |
| 3545 | CPQ | 3546 | CXCL13 | 3547 | PNOC | 3548 | ALDH1B1 |
| 3549 | KIR2DL4 | 3550 | CCT8 | 3551 | SLC39A1 | 3552 | UQCRFS1 |
| 3553 | HLA-DQA2 | 3554 | DCAF17 | 3555 | AMH | 3556 | MC1R |
| 3557 | PSMA5 | 3558 | PINK1 | 3559 | RPL38 | 3560 | TLR8 |
| 3561 | CD9 | 3562 | UQCR11 | 3563 | TMEM65 | 3564 | PLD3 |
| 3565 | RNASET2 | 3566 | SYTL4 | 3567 | RPN1 | 3568 | THBS3 |
| 3569 | SHMT2 | 3570 | PAGE2B | 3571 | CCHCR1 | 3572 | MIR526B |
| 3573 | SUPT5H | 3574 | ZNF384 | 3575 | NRARP | 3576 | TOE1 |
| 3577 | GPX3 | 3578 | GPNMB | 3579 | ANXA6 | 3580 | ARL15 |
| 3581 | SLC23A1 | 3582 | FOXC2 | 3583 | FLI1 | 3584 | PRCD |
| 3585 | PSMD3 | 3586 | KCNN3 | 3587 | MLN | 3588 | MIR550A1 |
| 3589 | DERL2 | 3590 | HSD3B1 | 3591 | SOX13 | 3592 | CPN1 |
| 3593 | LGALS4 | 3594 | SON | 3595 | CX3CL1 | 3596 | CROCC |
| 3597 | HSPE1 | 3598 | RSPO1 | 3599 | ATP8A1 | 3600 | CLEC7A |
| 3601 | RFX1 | 3602 | CCL17 | 3603 | RRM2 | 3604 | FBXL20 |
| 3605 | FCSK | 3606 | RPS6KA1 | 3607 | MBP | 3608 | POLRMT |
| 3609 | EIF2B3 | 3610 | ABO | 3611 | ARRB2 | 3612 | CD24 |
| 3613 | CALCOCO2 | 3614 | ATP8A2 | 3615 | UQCR10 | 3616 | TREM2 |
| 3617 | CLDN14 | 3618 | RAD21 | 3619 | TRRAP | 3620 | CSN1S1 |
| 3621 | MIR383 | 3622 | PCLAF | 3623 | CLCN2 | 3624 | MIR654 |
| 3625 | TNC | 3626 | PPIA | 3627 | EPHB2 | 3628 | RPL22 |
| 3629 | CTNND2 | 3630 | C19orf12 | 3631 | CFHR5 | 3632 | RAB11A |
| 3633 | HLA-DQB2 | 3634 | EHMT2 | 3635 | TNFRSF14 | 3636 | PLA2G4A |
| 3637 | MTREX | 3638 | PPARGC1B | 3639 | BCAM | 3640 | SPRTN |
| 3641 | MAP3K15 | 3642 | RPL34 | 3643 | RPS27 | 3644 | RPS6KA4 |
| 3645 | AIM2 | 3646 | MIR206 | 3647 | TRA2B | 3648 | DERL1 |
| 3649 | KHDRBS1 | 3650 | TICAM1 | 3651 | TCOF1 | 3652 | PLP1 |
| 3653 | PPP2CA | 3654 | LIPF | 3655 | RPL27 | 3656 | AAK1 |
| 3657 | NAGK | 3658 | SOX7 | 3659 | NEO1 | 3660 | RAB5A |
| 3661 | MIR520A | 3662 | PLIN3 | 3663 | DLG4 | 3664 | NAB1 |
| 3665 | CDH18 | 3666 | SLC23A2 | 3667 | CFAP221 | 3668 | HSPA1B |
| 3669 | EXT1 | 3670 | SLC12A2 | 3671 | SRSF3 | 3672 | SERBP1 |
| 3673 | ACRBP | 3674 | GSDMA | 3675 | DDX6 | 3676 | TRIB3 |
| 3677 | ZFP36L1 | 3678 | ZFP36 | 3679 | PREP | 3680 | TNNT3 |
| 3681 | CCN6 | 3682 | RPL31 | 3683 | MED24 | 3684 | RARG |
| 3685 | MIR6870 | 3686 | BAMBI | 3687 | RPL6 | 3688 | UBD |
| 3689 | RECQL5 | 3690 | TPM2 | 3691 | SLC7A5 | 3692 | DDHD2 |
| 3693 | AQP7 | 3694 | KIF11 | 3695 | HLA-DRB6 | 3696 | IFITM3 |
| 3697 | CHRD | 3698 | MIR199A2 | 3699 | MIR939 | 3700 | EMG1 |
| 3701 | CERS3 | 3702 | PROSER2 | 3703 | CPD | 3704 | ADRA1D |
| 3705 | TEAD1 | 3706 | RPS3 | 3707 | ADD1 | 3708 | MIR367 |
| 3709 | CD69 | 3710 | ACTR3 | 3711 | PBX1 | 3712 | NDRG1 |
| 3713 | PPP1R10 | 3714 | SPTLC2 | 3715 | TAGLN2 | 3716 | LGMN |
| 3717 | MGAM | 3718 | MED23 | 3719 | DAW1 | 3720 | AVPR1A |
| 3721 | MYL9 | 3722 | CCND2 | 3723 | HYCC1 | 3724 | TAX1BP1 |
| 3725 | S1PR3 | 3726 | MIR744 | 3727 | SMC1A | 3728 | NTF3 |
| 3729 | MSH3 | 3730 | CLDN6 | 3731 | SLC29A1 | 3732 | DBN1 |
| 3733 | UQCRC1 | 3734 | HNRNPD | 3735 | PTHLH | 3736 | GDA |
| 3737 | ATP5F1B | 3738 | ALOXE3 | 3739 | SART1 | 3740 | LMAN2 |
| 3741 | PSMD11 | 3742 | PHEX | 3743 | DOCK2 | 3744 | FOXC1 |
| 3745 | MFGE8 | 3746 | TK1 | 3747 | LDAF1 | 3748 | DIS3L |
| 3749 | PIGV | 3750 | SEMA6A | 3751 | MSX2 | 3752 | RPL26 |
| 3753 | NRXN3 | 3754 | AASS | 3755 | NCOA6 | 3756 | ATP5MG |
| 3757 | CTHRC1 | 3758 | SRSF4 | 3759 | NOLC1 | 3760 | TRAF1 |
| 3761 | ASAH2 | 3762 | COL6A1 | 3763 | IRAK1 | 3764 | YWHAB |
| 3765 | PDIA6 | 3766 | PDIA4 | 3767 | TNFAIP2 | 3768 | SNX27 |
| 3769 | VAPA | 3770 | PTGER2 | 3771 | SRSF9 | 3772 | CCL22 |
| 3773 | FITM2 | 3774 | IL27 | 3775 | DEDD | 3776 | AMPD3 |
| 3777 | H2AX | 3778 | ADCYAP1 | 3779 | SCRIB | 3780 | ELOVL6 |
| 3781 | FOXP1 | 3782 | MUC20 | 3783 | VTCN1 | 3784 | SLC31A1 |
| 3785 | UBC | 3786 | COPG1 | 3787 | MIR509-1 | 3788 | F2RL2 |
| 3789 | ENGASE | 3790 | METTL3 | 3791 | CXCL14 | 3792 | CHD2 |
| 3793 | CARD9 | 3794 | CGA | 3795 | LSS | 3796 | IL21 |
| 3797 | PAM | 3798 | CBFB | 3799 | S100A6 | 3800 | SMURF1 |
| 3801 | MUC4 | 3802 | JUNB | 3803 | MIR411 | 3804 | MIR518A1 |
| 3805 | SNRNP70 | 3806 | KCTD1 | 3807 | MIAT | 3808 | PSMB7 |
| 3809 | PDE3B | 3810 | ADRM1 | 3811 | HBB-LCR | 3812 | SFTPA1 |
| 3813 | PLSCR1 | 3814 | SLC9A1 | 3815 | PSMA6 | 3816 | DTX1 |
| 3817 | RXYLT1 | 3818 | MIR515-1 | 3819 | FLG2 | 3820 | MRI1 |
| 3821 | MIR302B | 3822 | CD109 | 3823 | ARL14 | 3824 | CEMIP2 |
| 3825 | SRP72 | 3826 | HCK | 3827 | DOCK11 | 3828 | TP63 |
| 3829 | CYP20A1 | 3830 | UBA1 | 3831 | PSEN2 | 3832 | LCN1 |
| 3833 | MIR508 | 3834 | LONP2 | 3835 | PLIN4 | 3836 | UCHL1 |
| 3837 | FARP1 | 3838 | KHDRBS3 | 3839 | LEMD2 | 3840 | WDR93 |
| 3841 | RBM8A | 3842 | MC4R | 3843 | SERPINB4 | 3844 | MIR520D |
| 3845 | NSFL1C | 3846 | NPR2 | 3847 | TRIM14 | 3848 | PRSS8 |
| 3849 | ABHD6 | 3850 | RASSF5 | 3851 | PI3 | 3852 | DGCR8 |
| 3853 | RPL29 | 3854 | PCBP2 | 3855 | FABP5 | 3856 | TET1 |
| 3857 | VSIR | 3858 | LINC00511 | 3859 | FOXJ1 | 3860 | GSTO1 |
| 3861 | MIR512-1 | 3862 | DNAJB6 | 3863 | RACGAP1 | 3864 | NAPB |
| 3865 | FOXE1 | 3866 | AGRN | 3867 | LHPP | 3868 | PSMG4 |
| 3869 | MAP2K7 | 3870 | HUWE1 | 3871 | M6PR | 3872 | ACKR1 |
| 3873 | AP4M1 | 3874 | IL22RA2 | 3875 | RPL32 | 3876 | MIR504 |
| 3877 | AMER1 | 3878 | ISG20 | 3879 | MTPAP | 3880 | PIAS3 |
| 3881 | PNPLA1 | 3882 | SLAMF1 | 3883 | SEC24D | 3884 | MFAP4 |
| 3885 | VARS2 | 3886 | LDB1 | 3887 | ALG14 | 3888 | UGT3A1 |
| 3889 | THAP1 | 3890 | IGFBP4 | 3891 | HPS1 | 3892 | TNFRSF12A |
| 3893 | ENDOG | 3894 | ATP2B1 | 3895 | IER3IP1 | 3896 | PSENEN |
| 3897 | NAA80 | 3898 | TLR1 | 3899 | IGFBP5 | 3900 | SEC23A |
| 3901 | CCAR1 | 3902 | SIK1 | 3903 | SYNPO | 3904 | SOX6 |
| 3905 | GAB1 | 3906 | NFKBIL1 | 3907 | GJC2 | 3908 | GNB3 |
| 3909 | LFNG | 3910 | SPATA2 | 3911 | IL12A-AS1 | 3912 | NCSTN |
| 3913 | SMURF2 | 3914 | MYO6 | 3915 | HTRA2 | 3916 | EGLN2 |
| 3917 | CTRB2 | 3918 | MIR605 | 3919 | GPER1 | 3920 | CTRB1 |
| 3921 | SERPINA9 | 3922 | MIR497 | 3923 | UHRF1 | 3924 | AHNAK |
| 3925 | ARF1 | 3926 | S100A1 | 3927 | HSPH1 | 3928 | NOX1 |
| 3929 | IL23R | 3930 | WARS1 | 3931 | TBX21 | 3932 | LRRK2 |
| 3933 | YIPF5 | 3934 | CAVIN2 | 3935 | CCT3 | 3936 | MATN2 |
| 3937 | PSMA2 | 3938 | HDAC4 | 3939 | JCAD | 3940 | CALU |
| 3941 | LPO | 3942 | PTPN6 | 3943 | GAS6 | 3944 | HLA-DMA |
| 3945 | EXOC6 | 3946 | STARD3 | 3947 | CRIPTO | 3948 | PNPLA7 |
| 3949 | PSMB5 | 3950 | IFIT1 | 3951 | CIRBP | 3952 | NCAPG2 |
| 3953 | DMTN | 3954 | IGFBP7 | 3955 | CEP89 | 3956 | SLC2A5 |
| 3957 | AHSP | 3958 | GSDMD | 3959 | PFN1 | 3960 | TRIB2 |
| 3961 | PSMD13 | 3962 | LAMB1 | 3963 | KDM8 | 3964 | AEBP1 |
| 3965 | RBM39 | 3966 | PRSS2 | 3967 | SURF4 | 3968 | UBTF |
| 3969 | MOGAT1 | 3970 | COPB1 | 3971 | PSMC5 | 3972 | SCNN1G |
| 3973 | YWHAG | 3974 | SEC24A | 3975 | PROCR | 3976 | LSM2 |
| 3977 | DDX50 | 3978 | DROSHA | 3979 | PUS10 | 3980 | COPG2 |
| 3981 | CALML3 | 3982 | OS9 | 3983 | PPIAL4A | 3984 | PSMC3 |
| 3985 | ESRRG | 3986 | FKBP5 | 3987 | P2RX4 | 3988 | SVIL |
| 3989 | GRPEL1 | 3990 | XRN1 | 3991 | PREPL | 3992 | HMGB2 |
| 3993 | ING4 | 3994 | TPR | 3995 | CREBZF | 3996 | ITGAX |
| 3997 | PIH1D1 | 3998 | PHF20 | 3999 | NHERF2 | 4000 | DHX16 |
| 4001 | DLEU2 | 4002 | S100A10 | 4003 | LY6G5B | 4004 | RNH1 |
| 4005 | CLDN2 | 4006 | GATAD2A | 4007 | HIPK2 | 4008 | LTB |
| 4009 | CHRNA5 | 4010 | B4GAT1 | 4011 | FZD5 | 4012 | ARIH1 |
| 4013 | NID1 | 4014 | MIR518D | 4015 | FDX1 | 4016 | LINC01093 |
| 4017 | RPS4X | 4018 | LOC113633877 | 4019 | LINC00336 | 4020 | SNAP29 |
| 4021 | BEST1 | 4022 | PIGS | 4023 | PRX | 4024 | POPDC1 |
| 4025 | CYP46A1 | 4026 | CEP192 | 4027 | PAPPA | 4028 | GFUS |
| 4029 | TBP | 4030 | RGMA | 4031 | ACBD5 | 4032 | SKIL |
| 4033 | MIR708 | 4034 | NUMBL | 4035 | IL37 | 4036 | CNTROB |
| 4037 | HNRNPH3 | 4038 | WDR4 | 4039 | SLC9A9 | 4040 | MAP1B |
| 4041 | YTHDC2 | 4042 | ANXA3 | 4043 | PYDC1 | 4044 | PRKCE |
| 4045 | ROCK2 | 4046 | LPCAT1 | 4047 | ATPAF1 | 4048 | JMJD6 |
| 4049 | IGHM | 4050 | ICAM3 | 4051 | GOLPH3 | 4052 | NTN1 |
| 4053 | PSMD1 | 4054 | STX18 | 4055 | CCL8 | 4056 | HTR2B |
| 4057 | MX2 | 4058 | WNT9A | 4059 | SLC7A11 | 4060 | CDK8 |
| 4061 | GDF11 | 4062 | PUS7 | 4063 | CSN2 | 4064 | NSUN2 |
| 4065 | FGF9 | 4066 | SCG5 | 4067 | NALT1 | 4068 | IPO7 |
| 4069 | ALKBH5 | 4070 | DUS3L | 4071 | MIR514A1 | 4072 | PHF1 |
| 4073 | FCGR3B | 4074 | TNFSF14 | 4075 | C4BPA | 4076 | MIB2 |
| 4077 | ATF6B | 4078 | MAN2B2 | 4079 | DNAJC8 | 4080 | DUOX1 |
| 4081 | CCR4 | 4082 | B3GALNT2 | 4083 | FLII | 4084 | RFNG |
| 4085 | CHGB | 4086 | PLPP2 | 4087 | PRPF31 | 4088 | LRG1 |
| 4089 | ADAMTSL1 | 4090 | SEMA3B | 4091 | KDM3A | 4092 | PTGIS |
| 4093 | PYY | 4094 | ADORA1 | 4095 | CSPG4 | 4096 | SCAMP2 |
| 4097 | LINC01133 | 4098 | NFE2 | 4099 | MYH1 | 4100 | LOC111832670 |
| 4101 | RNF213 | 4102 | TNFAIP8L2 | 4103 | CTSH | 4104 | ATP13A1 |
| 4105 | TFB2M | 4106 | CAVIN3 | 4107 | HCG25 | 4108 | PCDH19 |
| 4109 | MTX2 | 4110 | CASC3 | 4111 | IGKV1-33 | 4112 | RAP1A |
| 4113 | CLPTM1L | 4114 | FAM111B | 4115 | PLBD2 | 4116 | ROBO1 |
| 4117 | GRB7 | 4118 | ANKRD11 | 4119 | TIRAP | 4120 | DFFA |
| 4121 | COG3 | 4122 | FGF5 | 4123 | ADARB1 | 4124 | FGF16 |
| 4125 | TRIP11 | 4126 | MEST | 4127 | TERF1 | 4128 | LCLAT1 |
| 4129 | LOC111674463 | 4130 | ZUP1 | 4131 | PDCD10 | 4132 | NIPA1 |
| 4133 | ATP11B | 4134 | LOC129934769 | 4135 | PTGES | 4136 | PGAP3 |
| 4137 | SLC4A7 | 4138 | RPL23A | 4139 | DPP6 | 4140 | TNFRSF18 |
| 4141 | TSPAN32 | 4142 | ZNF77 | 4143 | MIR29B2 | 4144 | CREB3 |
| 4145 | KLLN | 4146 | HRNR | 4147 | COQ3 | 4148 | RPL15 |
| 4149 | UBE3C | 4150 | CD1A | 4151 | C1GALT1C1 | 4152 | F2RL1 |
| 4153 | APH1A | 4154 | IFNL1 | 4155 | CHRNA3 | 4156 | DYNLL1 |
| 4157 | MINCR | 4158 | AP1S3 | 4159 | SEC13 | 4160 | IBA57 |
| 4161 | ENOSF1 | 4162 | SELENOI | 4163 | GDF9 | 4164 | H2BC21 |
| 4165 | B4GALT7 | 4166 | LOC129998967 | 4167 | PSMD14 | 4168 | HLA-S |
| 4169 | SIAE | 4170 | OXT | 4171 | CIB1 | 4172 | CLDN9 |
| 4173 | IL18R1 | 4174 | CD22 | 4175 | VPS52 | 4176 | PREB |
| 4177 | CCL18 | 4178 | MLLT6 | 4179 | ABHD12 | 4180 | MIR517A |
| 4181 | TMEM14C | 4182 | SNW1 | 4183 | ZBTB7A | 4184 | PGM2 |
| 4185 | ATP8B4 | 4186 | VTI1B | 4187 | ZFAND6 | 4188 | ADRA1B |
| 4189 | HLA-DMB | 4190 | TRAF3IP2 | 4191 | ZBP1 | 4192 | CSF2RA |
| 4193 | GUCY2C | 4194 | B3GALT4 | 4195 | AHSA1 | 4196 | SPSB2 |
| 4197 | APLNR | 4198 | GRHL2 | 4199 | RBMS1 | 4200 | DNAJA2 |
| 4201 | FBLN1 | 4202 | RPS8 | 4203 | CERS5 | 4204 | NPDC1 |
| 4205 | PNPLA5 | 4206 | VPS35 | 4207 | NCR1 | 4208 | ZCCHC14 |
| 4209 | ARID3A | 4210 | RAB10 | 4211 | MFAP2 | 4212 | RBMS3 |
| 4213 | RIC1 | 4214 | POLR2L | 4215 | TRIM25 | 4216 | SEMA3C |
| 4217 | KDM6B | 4218 | IL13RA2 | 4219 | YWHAQ | 4220 | WIPF2 |
| 4221 | MAL | 4222 | CRK | 4223 | ADAMTS3 | 4224 | CD180 |
| 4225 | ASF1B | 4226 | MYOD1 | 4227 | GCFC2 | 4228 | IFITM1 |
| 4229 | SCARA5 | 4230 | SLC48A1 | 4231 | CD248 | 4232 | SQLE |
| 4233 | ITM2B | 4234 | INPP4A | 4235 | MED13L | 4236 | ISCA2 |
| 4237 | LPIN3 | 4238 | LOC129998833 | 4239 | NOP58 | 4240 | CCL25 |
| 4241 | DHX15 | 4242 | EIF2AK1 | 4243 | VAMP3 | 4244 | LOC129998796 |
| 4245 | COX11 | 4246 | QRSL1 | 4247 | KIR2DL5A | 4248 | FBXO7 |
| 4249 | GDF7 | 4250 | SLX1A | 4251 | VDAC2 | 4252 | BMPER |
| 4253 | LEF1-AS1 | 4254 | STMN2 | 4255 | PIGY | 4256 | MT-TK |
| 4257 | LPAR2 | 4258 | WSPAR | 4259 | TMEM30B | 4260 | PSMB1 |
| 4261 | AUP1 | 4262 | PBX2 | 4263 | AIF1 | 4264 | HAL |
| 4265 | SERPINB12 | 4266 | CIAPIN1 | 4267 | FER | 4268 | RPS29 |
| 4269 | EHD1 | 4270 | TNFSF4 | 4271 | WNT9B | 4272 | LOC130058947 |
| 4273 | INHBA | 4274 | CCL7 | 4275 | DDX60L | 4276 | POLR3C |
| 4277 | NCBP1 | 4278 | SKIC8 | 4279 | DUS2 | 4280 | RPL7 |
| 4281 | NDFIP1 | 4282 | CCN3 | 4283 | SMAD1 | 4284 | EDC4 |
| 4285 | NPHP3-AS1 | 4286 | HAND2 | 4287 | IL22RA1 | 4288 | COPE |
| 4289 | SIGLEC1 | 4290 | VSIG4 | 4291 | TEX14 | 4292 | GZMM |
| 4293 | IL34 | 4294 | SLCO3A1 | 4295 | WDR43 | 4296 | CHSY1 |
| 4297 | FBXO6 | 4298 | RECQL | 4299 | CELA1 | 4300 | SMPD3 |
| 4301 | YWHAH | 4302 | FBXO27 | 4303 | ZP4 | 4304 | ISL1 |
| 4305 | LRP1B | 4306 | PLA2G1B | 4307 | TIMP4 | 4308 | HBE1 |
| 4309 | METTL14 | 4310 | ABCF2 | 4311 | NANS | 4312 | FGF22 |
| 4313 | GXYLT1 | 4314 | CREB3L2 | 4315 | MIR4435-2HG | 4316 | HPGD |
| 4317 | GRP | 4318 | AIMP1 | 4319 | FBXO2 | 4320 | MT1H |
| 4321 | UQCC1 | 4322 | MANF | 4323 | COMP | 4324 | SORT1 |
| 4325 | DCTN2 | 4326 | MIR454 | 4327 | DDX11-AS1 | 4328 | SUPV3L1 |
| 4329 | SLC25A11 | 4330 | S100A12 | 4331 | PSMC2 | 4332 | ZNF513 |
| 4333 | HEY2 | 4334 | ABHD16A | 4335 | WASHC5 | 4336 | DDI2 |
| 4337 | LOC130005193 | 4338 | BICD2 | 4339 | MARVELD2 | 4340 | MAEA |
| 4341 | ZC3H12A | 4342 | TCF19 | 4343 | SETD1B | 4344 | TSLP |
| 4345 | GH2 | 4346 | LOC654780 | 4347 | PRPF6 | 4348 | FGF17 |
| 4349 | DHX37 | 4350 | TSPAN8 | 4351 | RMI2 | 4352 | AP1M1 |
| 4353 | SMAD5 | 4354 | SEPTIN7 | 4355 | GZF1 | 4356 | PSMB6 |
| 4357 | ECM1 | 4358 | GOLGB1 | 4359 | ABCA4 | 4360 | PLVAP |
| 4361 | GPAT4 | 4362 | C5AR1 | 4363 | DNAJB12 | 4364 | PRG2 |
| 4365 | DIO2 | 4366 | COL13A1 | 4367 | HDAC8 | 4368 | HILPDA |
| 4369 | ACRV1 | 4370 | INPPL1 | 4371 | MAPKAPK5 | 4372 | NCAN |
| 4373 | TUBB2B | 4374 | RAN | 4375 | CNTLN | 4376 | RPS23 |
| 4377 | EGFL7 | 4378 | DHFR2 | 4379 | GPR161 | 4380 | UCP3 |
| 4381 | MAML3 | 4382 | RGMB | 4383 | DNM3 | 4384 | SLC25A5 |
| 4385 | CTRC | 4386 | FUT4 | 4387 | KLHL41 | 4388 | ESYT1 |
| 4389 | KLHDC7A | 4390 | UQCRH | 4391 | BACH1 | 4392 | DIRAS3 |
| 4393 | PLEKHG6 | 4394 | RPS28 | 4395 | MAML2 | 4396 | COIL |
| 4397 | DDX21 | 4398 | LTBP2 | 4399 | LNX1 | 4400 | EXOC7 |
| 4401 | HSD17B7 | 4402 | CACNB2 | 4403 | EPHA8 | 4404 | SLC1A1 |
| 4405 | POLR1A | 4406 | ZNF780B | 4407 | SLC9A6 | 4408 | TSBP1-AS1 |
| 4409 | EBLN2 | 4410 | PRMT1 | 4411 | MRPS22 | 4412 | EIF3A |
| 4413 | PSMB2 | 4414 | NCR3 | 4415 | KBTBD13 | 4416 | MUS81 |
| 4417 | ERAP1 | 4418 | MACROD2 | 4419 | DCDC2B | 4420 | HNRNPUL2-BSCL2 |
| 4421 | MED16 | 4422 | PEX11G | 4423 | AKAP12 | 4424 | FSTL1 |
| 4425 | SNTB2 | 4426 | RNF152 | 4427 | TUBB6 | 4428 | SERTAD1 |
| 4429 | ST3GAL2 | 4430 | GAK | 4431 | YME1L1 | 4432 | FFAR4 |
| 4433 | PSMC6 | 4434 | SLC10A7 | 4435 | CMA1 | 4436 | PDCD4 |
| 4437 | FCN1 | 4438 | LOC126862987 | 4439 | PPP1R3E | 4440 | IL9R |
| 4441 | HLA-DOA | 4442 | PPIAL4C | 4443 | UBE3A | 4444 | PARVA |
| 4445 | TANK | 4446 | TRIM33 | 4447 | ABCC12 | 4448 | XXYLT1 |
| 4449 | DNAJC6 | 4450 | RAD23B | 4451 | TPH1 | 4452 | DLEU1 |
| 4453 | MEF2A | 4454 | FBXO42 | 4455 | PATJ | 4456 | PACSIN2 |
| 4457 | ZFAND4 | 4458 | KCNQ2 | 4459 | SPATA17 | 4460 | SSTR5 |
| 4461 | SEM1 | 4462 | NOP56 | 4463 | P2RY2 | 4464 | HLA-F |
| 4465 | KIFC3 | 4466 | FOXP2 | 4467 | MIR155HG | 4468 | FGF8 |
| 4469 | RPIA | 4470 | SEC31A | 4471 | ARHGAP45 | 4472 | RAVER1 |
| 4473 | TACSTD2 | 4474 | VANGL2 | 4475 | TRPC1 | 4476 | LOC106804612 |
| 4477 | GAS1 | 4478 | UTS2R | 4479 | ATAD2 | 4480 | EDN2 |
| 4481 | CDV3 | 4482 | LRP4 | 4483 | FBXO44 | 4484 | CLTCL1 |
| 4485 | PPP2R2A | 4486 | NR4A3 | 4487 | AMY2A | 4488 | MBL1P |
| 4489 | ZKSCAN1 | 4490 | MIR498 | 4491 | CYP21A1P | 4492 | PLA1A |
| 4493 | FMOD | 4494 | SCGB1A1 | 4495 | LOC399900 | 4496 | TFF2 |
| 4497 | VAMP1 | 4498 | TBX2 | 4499 | COPB2 | 4500 | RAD51D |
| 4501 | LAMC1 | 4502 | FIBP | 4503 | CDCA3 | 4504 | GALNT11 |
| 4505 | NOG | 4506 | ASIC1 | 4507 | HIVEP1 | 4508 | NDUFB2 |
| 4509 | NEURL1B | 4510 | ZNF185 | 4511 | CLCN3 | 4512 | ZNF546 |
| 4513 | DDX41 | 4514 | SLC38A5 | 4515 | GMNN | 4516 | ZNF417 |
| 4517 | ZNF133 | 4518 | C1RL-AS1 | 4519 | ESRRA | 4520 | SLC17A2 |
| 4521 | CHCHD2 | 4522 | BAG2 | 4523 | KLHL8 | 4524 | ACY3 |
| 4525 | STAMBP | 4526 | CAPN7 | 4527 | RNU4ATAC | 4528 | TTLL11 |
| 4529 | KCP | 4530 | MAP3K6 | 4531 | ENSG00000256967 | 4532 | PRKG2 |
| 4533 | PLPP3 | 4534 | ADAMTS9-AS2 | 4535 | IL1R2 | 4536 | PDCD6 |
| 4537 | PDE7B | 4538 | PPP1R14A | 4539 | CCL15 | 4540 | NUBP1 |
| 4541 | LCS1 | 4542 | ERG | 4543 | NCAPD2 | 4544 | TSC22D3 |
| 4545 | C19orf48P | 4546 | RALA | 4547 | ATAD1 | 4548 | GJC1 |
| 4549 | ATP10D | 4550 | CSH2 | 4551 | NADSYN1 | 4552 | HSCB |
| 4553 | HMGCS1 | 4554 | SNU13 | 4555 | ELAPOR2 | 4556 | GGTLC3 |
| 4557 | RPL30 | 4558 | C4BPB | 4559 | SLC1A4 | 4560 | RNASEL |
| 4561 | CMC2 | 4562 | PIGN | 4563 | MSBP1 | 4564 | ERCC6L2 |
| 4565 | MGP | 4566 | SCLY | 4567 | TFPT | 4568 | EFS |
| 4569 | SPEF2 | 4570 | MIR34B | 4571 | MIR571 | 4572 | TMBIM1 |
| 4573 | TRP-AGG2-5 | 4574 | BRF1 | 4575 | SPHK2 | 4576 | PNPLA4 |
| 4577 | KLRG1 | 4578 | IGHG1 | 4579 | LOC130062560 | 4580 | CMC4 |
| 4581 | DCLRE1B | 4582 | MAFG | 4583 | ABHD2 | 4584 | NINJ1 |
| 4585 | SLC34A2 | 4586 | PSME4 | 4587 | MIR449A | 4588 | PTRHD1 |
| 4589 | SYNGAP1 | 4590 | MARCO | 4591 | TAPBP | 4592 | RPL18A |
| 4593 | SDC2 | 4594 | TMPRSS2 | 4595 | ANAPC1 | 4596 | MIR522 |
| 4597 | MEF2C | 4598 | PLA2G7 | 4599 | SLC25A38 | 4600 | SEC24B |
| 4601 | ENTPD3 | 4602 | PLXNA2 | 4603 | GPSM3 | 4604 | IFNA8 |
| 4605 | PCOLCE | 4606 | SUMO1 | 4607 | ITLN1 | 4608 | NSUN5 |
| 4609 | CGB3 | 4610 | CLDN4 | 4611 | STX10 | 4612 | LAIR1 |
| 4613 | IRF9 | 4614 | KRT13 | 4615 | CFD | 4616 | OPRK1 |
| 4617 | NMD3 | 4618 | LSM14A | 4619 | PTAFR | 4620 | TEN1 |
| 4621 | TOLLIP | 4622 | KCNK4 | 4623 | CISD2 | 4624 | SPATS2L |
| 4625 | HBQ1 | 4626 | POMK | 4627 | LRRC32 | 4628 | GOLIM4 |
| 4629 | LIPM | 4630 | ANXA4 | 4631 | POU2F3 | 4632 | TBL1X |
| 4633 | ACTN3 | 4634 | MCTP2 | 4635 | PSMD7 | 4636 | SLX4IP |
| 4637 | S1PR1 | 4638 | MBD6 | 4639 | CBX7 | 4640 | PHLDB1 |
| 4641 | HSD17B1 | 4642 | ANP32A | 4643 | TIGIT | 4644 | FIS1 |
| 4645 | NAP1L1 | 4646 | ST8SIA1 | 4647 | COPZ1 | 4648 | MIR124-3 |
| 4649 | ZNF699 | 4650 | GRSF1 | 4651 | GNG3 | 4652 | TAOK1 |
| 4653 | AGR2 | 4654 | LINC00667 | 4655 | PIGQ | 4656 | RMND5B |
| 4657 | THOP1 | 4658 | PTPRA | 4659 | TNFSF9 | 4660 | XPO4 |
| 4661 | PNPLA8 | 4662 | ARHGEF10L | 4663 | CCNG2 | 4664 | CCDC171 |
| 4665 | HDLBP | 4666 | NRM | 4667 | NIPBL | 4668 | RPL36 |
| 4669 | TERF2IP | 4670 | EBI3 | 4671 | EBPL | 4672 | KIR2DS2 |
| 4673 | STX6 | 4674 | ABHD3 | 4675 | SNAPIN | 4676 | ENSG00000219410 |
| 4677 | EPS15 | 4678 | SEL1L3 | 4679 | MT-RNR1 | 4680 | CWH43 |
| 4681 | KLF14 | 4682 | ABCB5 | 4683 | MSL1 | 4684 | PLA2G15 |
| 4685 | TRMT112 | 4686 | TNFRSF4 | 4687 | RAB14 | 4688 | CAPSL |
| 4689 | DEFB4A | 4690 | EIF3E | 4691 | RILPL1 | 4692 | FAM3A |
| 4693 | FZD4 | 4694 | MIR410 | 4695 | TSC22D4 | 4696 | SLC35A5 |
| 4697 | CTSZ | 4698 | SNORA64 | 4699 | PWAR4 | 4700 | RPS12 |
| 4701 | DLL3 | 4702 | PDE4B | 4703 | SUV39H1 | 4704 | ADORA2B |
| 4705 | ITPK1 | 4706 | SYNM | 4707 | HIF1AN | 4708 | CCDC112 |
| 4709 | CLDND1 | 4710 | ZBTB17 | 4711 | RNF8 | 4712 | MAST3 |
| 4713 | ACAN | 4714 | CWC25 | 4715 | PEX11A | 4716 | MTX1 |
| 4717 | CXCL3 | 4718 | SF3B4 | 4719 | OGA | 4720 | PLB1 |
| 4721 | IFIT3 | 4722 | SLC4A5 | 4723 | CCDC88A | 4724 | LITAF |
| 4725 | MAP1S | 4726 | BLOC1S4 | 4727 | MATN3 | 4728 | SPRY4-AS1 |
| 4729 | GTF2H5 | 4730 | EFNA4 | 4731 | NELFCD | 4732 | GZMA |
| 4733 | SP140L | 4734 | SNAPC4 | 4735 | PRRC2A | 4736 | B4GALT6 |
| 4737 | MARCHF6 | 4738 | CLCN4 | 4739 | NFE2L3 | 4740 | SH2D4A |
| 4741 | RBFOX3 | 4742 | MAN2A2 | 4743 | F2RL3 | 4744 | TNFRSF17 |
| 4745 | ZPBP2 | 4746 | PI4K2B | 4747 | GMIP | 4748 | GLMN |
| 4749 | GRIN3B | 4750 | GP9 | 4751 | SLC2A8 | 4752 | LOC130057954 |
| 4753 | INHBB | 4754 | TET3 | 4755 | DCP2 | 4756 | DNAJC17 |
| 4757 | SNHG8 | 4758 | CYB5B | 4759 | VPS37D | 4760 | CEP295 |
| 4761 | MRPL34 | 4762 | SLC31A2 | 4763 | TPH2 | 4764 | DCP1A |
| 4765 | VPS13C | 4766 | NACC1 | 4767 | KLK6 | 4768 | DEAF1 |
| 4769 | EXOSC10 | 4770 | PIK3R3 | 4771 | MMP17 | 4772 | ZC3HAV1 |
| 4773 | BMAL2 | 4774 | NTMT1 | 4775 | PTGIR | 4776 | RPL3P2 |
| 4777 | RBMY1A1 | 4778 | MRPS2 | 4779 | MAPKAP1 | 4780 | HTR3A |
| 4781 | PDCD5 | 4782 | PDE4D | 4783 | ONECUT3 | 4784 | FAM215A |
| 4785 | H4C16 | 4786 | FCGR1A | 4787 | VCAN | 4788 | AQP5 |
| 4789 | GJA8 | 4790 | FAXC | 4791 | ENSG00000247853 | 4792 | RBM48 |
| 4793 | HES5 | 4794 | GLT8D2 | 4795 | SPAG17 | 4796 | KANK2 |
| 4797 | MBD2 | 4798 | LOXL1 | 4799 | DIS3 | 4800 | SNAP23 |
| 4801 | CNOT7 | 4802 | KIR2DL1 | 4803 | FAT1 | 4804 | USP22 |
| 4805 | ATP1A2 | 4806 | RFX3 | 4807 | CCDC180 | 4808 | TYRO3 |
| 4809 | LUM | 4810 | ATP9A | 4811 | TXNDC9 | 4812 | MCM6 |
| 4813 | TMCO1 | 4814 | MIR381 | 4815 | RPAP3 | 4816 | LAMP3 |
| 4817 | LUC7L2 | 4818 | CCDC22 | 4819 | CTBP2 | 4820 | CILP2 |
| 4821 | MIA3 | 4822 | FGF6 | 4823 | TIE1 | 4824 | ASIC5 |
| 4825 | ANP32B | 4826 | CRACR2A | 4827 | CRABP1 | 4828 | LORICRIN |
| 4829 | TYSND1 | 4830 | IFNA10 | 4831 | ZFYVE9 | 4832 | VCPIP1 |
| 4833 | BOP1 | 4834 | TAS2R20 | 4835 | NETO1 | 4836 | REEP1 |
| 4837 | ZNF267 | 4838 | KCNMA1 | 4839 | TRPM5 | 4840 | SATB1 |
| 4841 | NEURL1 | 4842 | CCER2 | 4843 | MICA-AS1 | 4844 | NASP |
| 4845 | PGAM1 | 4846 | SMARCA5 | 4847 | MRPL37 | 4848 | LOC129937586 |
| 4849 | TWSG1 | 4850 | EXOSC9 | 4851 | WDR82 | 4852 | TRIB1AL |
| 4853 | GGPS1 | 4854 | CCL26 | 4855 | GSTO2 | 4856 | MACC1 |
| 4857 | TLCD4-RWDD3 | 4858 | PLK4 | 4859 | AP1G1 | 4860 | DNM1 |
| 4861 | LOC130001683 | 4862 | TNFAIP8L1 | 4863 | GOSR1 | 4864 | NUP88 |
| 4865 | P2RY1 | 4866 | DPP8 | 4867 | TNFSF13 | 4868 | ARSH |
| 4869 | HCG27 | 4870 | PRKCG | 4871 | MSMO1 | 4872 | C6orf47 |
| 4873 | PAK2 | 4874 | RNF20 | 4875 | ZNHIT3 | 4876 | IFNA4 |
| 4877 | EXOSC6 | 4878 | BTBD10 | 4879 | SLC7A8 | 4880 | ATP10A |
| 4881 | PSORS1C1 | 4882 | ASF1A | 4883 | MIR190B | 4884 | PHF6 |
| 4885 | SNORA48 | 4886 | CLPS | 4887 | SIGLEC7 | 4888 | ASIC3 |
| 4889 | FCGBP | 4890 | TOMM40 | 4891 | USP15 | 4892 | NUDT15 |
| 4893 | DHX38 | 4894 | TRN-GTT2-1 | 4895 | OLR1 | 4896 | MIR9-3 |
| 4897 | ALPK2 | 4898 | RBFOX1 | 4899 | ZNF408 | 4900 | GPR183 |
| 4901 | PIGW | 4902 | UTP14A | 4903 | INPP5K | 4904 | DIP2C |
| 4905 | GP1BA | 4906 | RPA3 | 4907 | WHR1B | 4908 | LOC105374114 |
| 4909 | RSPO2 | 4910 | BDH2 | 4911 | FSTL3 | 4912 | ISCA1 |
| 4913 | GIP | 4914 | HAS2 | 4915 | SLC28A2 | 4916 | PGAP1 |
| 4917 | WNT2 | 4918 | GPR31 | 4919 | TFDP2 | 4920 | CSF2RB |
| 4921 | AGTR2 | 4922 | CDH11 | 4923 | KIR3DL2 | 4924 | EPX |
| 4925 | POLI | 4926 | TRAF4 | 4927 | KIR2DS4 | 4928 | ALKAL1 |
| 4929 | SYTL2 | 4930 | CH25H | 4931 | PSME3 | 4932 | MFF |
| 4933 | FOXL1 | 4934 | MIR1246 | 4935 | KRT10-AS1 | 4936 | MIR663A |
| 4937 | USF2 | 4938 | SLC35A4 | 4939 | PMP22 | 4940 | TAMM41 |
| 4941 | SYNJ1 | 4942 | PPA2 | 4943 | FAM223A | 4944 | NLRP6 |
| 4945 | ELMO2 | 4946 | BASP1 | 4947 | EGOT | 4948 | MIR576 |
| 4949 | ATP6V1C1 | 4950 | LGALS7 | 4951 | RPL22L1 | 4952 | MPV17L2 |
| 4953 | ZNF558 | 4954 | LOC129389388 | 4955 | DHX58 | 4956 | PORCN |
| 4957 | NFATC3 | 4958 | UFM1 | 4959 | ENPP3 | 4960 | IFT25 |
| 4961 | EXOSC8 | 4962 | TGS1 | 4963 | MMRN1 | 4964 | RPL6P8 |
| 4965 | ATP1A4 | 4966 | LCOR | 4967 | CKS2 | 4968 | LOC126860438 |
| 4969 | HLA-DRB4 | 4970 | ITFG1 | 4971 | TAF1 | 4972 | POLR1D |
| 4973 | IFNA5 | 4974 | KLF5 | 4975 | LOC112694756 | 4976 | GPX7 |
| 4977 | GIT2 | 4978 | NOSIP | 4979 | DRD4 | 4980 | HTR2A |
| 4981 | SMG6 | 4982 | SCARNA12 | 4983 | IRAK3 | 4984 | CEP43 |
| 4985 | IFNG-AS1 | 4986 | LOC643339 | 4987 | IFNA14 | 4988 | HAPLN1 |
| 4989 | FUNDC2 | 4990 | CRX | 4991 | ABHD15 | 4992 | PSORS1C2 |
| 4993 | PTPRH | 4994 | SLC9B1 | 4995 | NBR1 | 4996 | FERMT1 |
| 4997 | SETD7 | 4998 | RHEX | 4999 | LPAR1 | 5000 | CSH1 |
| 5001 | PROK1 | 5002 | MIR194-1 | 5003 | UCHL5 | 5004 | DYNC1LI2 |
| 5005 | CXCL6 | 5006 | IFNA21 | 5007 | CWF19L1 | 5008 | WHR1 |
| 5009 | ZNF148 | 5010 | TRD-GTC9-1 | 5011 | ATG9B | 5012 | YES1 |
| 5013 | IFNA16 | 5014 | PIGO | 5015 | LINC01100 | 5016 | PSG2 |
| 5017 | FAF1 | 5018 | RTF1 | 5019 | NSDHL | 5020 | ELOVL4 |
| 5021 | SOX1 | 5022 | BRS3 | 5023 | ZFPL1 | 5024 | FAM43A |
| 5025 | ESPL1 | 5026 | SRY | 5027 | GDPD3 | 5028 | IPO4 |
| 5029 | HRH2 | 5030 | HES6 | 5031 | PRKD3 | 5032 | SSX2 |
| 5033 | ACYP2 | 5034 | RDH5 | 5035 | MCAM | 5036 | CA1 |
| 5037 | CCDC86 | 5038 | SYT7 | 5039 | ZIC2 | 5040 | MAN1A2 |
| 5041 | IFRD1 | 5042 | THBS4 | 5043 | ERLEC1 | 5044 | UBE2Q1 |
| 5045 | IBSP | 5046 | LOC100288123 | 5047 | PPP1R3D | 5048 | CENATAC |
| 5049 | UTP15 | 5050 | SATB2 | 5051 | CD226 | 5052 | SRPK2 |
| 5053 | MIR612 | 5054 | KIR3DL3 | 5055 | AKAP11 | 5056 | MT-ND4L |
| 5057 | HLA-DOB | 5058 | TBX6 | 5059 | RNU1-1 | 5060 | LOC130056931 |
| 5061 | SYNGR1 | 5062 | SP7 | 5063 | IFNA7 | 5064 | IFNA17 |
| 5065 | POGLUT2 | 5066 | CARMN | 5067 | BMP5 | 5068 | RFX2 |
| 5069 | PNLIPRP2 | 5070 | CCL28 | 5071 | HAR1B | 5072 | PPP1R13L |
| 5073 | TCF15 | 5074 | EXOSC5 | 5075 | SEMA5A | 5076 | ENSG00000272540 |
| 5077 | CCR3 | 5078 | HIF1A-AS2 | 5079 | MIR4651 | 5080 | SNRPN |
| 5081 | CPLX2 | 5082 | ZNF621 | 5083 | LINC-PINT | 5084 | MIB1 |
| 5085 | WASHC5-AS1 | 5086 | CARD10 | 5087 | TNP2 | 5088 | KCTD11 |
| 5089 | GUCY1A1 | 5090 | PBX4 | 5091 | SMTN | 5092 | AGPAT5 |
| 5093 | DMRTA1 | 5094 | DUT | 5095 | HTR6 | 5096 | SLC22A15 |
| 5097 | MRGPRX4 | 5098 | UBQLN1 | 5099 | GUCA2A | 5100 | POLA1 |
| 5101 | ENSG00000253106 | 5102 | ITGA11 | 5103 | LINC01139 | 5104 | KCNK18 |
| 5105 | FBXL19 | 5106 | NBPF20 | 5107 | CEP135 | 5108 | NEMP2 |
| 5109 | KCTD3 | 5110 | ENSG00000272501 | 5111 | EGR3 | 5112 | KRT16 |
| 5113 | LOC126863275 | 5114 | SMIM1 | 5115 | MFNG | 5116 | MIR409 |
| 5117 | MIR208B | 5118 | CERS6 | 5119 | OR1E2 | 5120 | FAM66B |
| 5121 | SMIM2-IT1 | 5122 | BMS1 | 5123 | RNF139-DT | 5124 | TTC17 |
| 5125 | DNAJB2 | 5126 | OGN | 5127 | GPAA1 | 5128 | KRT33A |
| 5129 | LARP7 | 5130 | GSTA4 | 5131 | TTC7B | 5132 | ELK3 |
| 5133 | DCDC2C | 5134 | MAG | 5135 | PAX1 | 5136 | LMF2 |
| 5137 | TSPY1 | 5138 | FCRL6 | 5139 | ITSN2 | 5140 | COL21A1 |
| 5141 | HSPA12A | 5142 | LTN1 | 5143 | DGKQ | 5144 | ALG1L1P |
| 5145 | ASRGL1 | 5146 | LBH | 5147 | GIPC1 | 5148 | DDIT4 |
| 5149 | HSD17B3 | 5150 | MIR629 | 5151 | CAMKMT | 5152 | DNER |
| 5153 | HES4 | 5154 | RWDD3-DT | 5155 | SMC5 | 5156 | CALCOCO1 |
| 5157 | MMP24 | 5158 | LY96 | 5159 | CDC42BPG | 5160 | CEP250 |
| 5161 | ITSN1 | 5162 | GYPB | 5163 | HEYL | 5164 | MPP1 |
| 5165 | TSPAN33 | 5166 | GCNT3 | 5167 | ANGPTL2 | 5168 | USP4 |
| 5169 | MIR3677 | 5170 | ATP6AP1L | 5171 | CCL1 | 5172 | PXDNL |
| 5173 | NPB | 5174 | ANKRD13C-DT | 5175 | LOC102723407 | 5176 | ATP2A1 |
| 5177 | TBC1D20 | 5178 | DMRTA2 | 5179 | DNAH12 | 5180 | ACP3 |
| 5181 | DHRS4-AS1 | 5182 | GULP1 | 5183 | ENSG00000271581 | 5184 | VWCE |
| 5185 | TNKS | 5186 | CSRP2 | 5187 | DBNL | 5188 | LOC124905027 |
| 5189 | PSORS1C3 | 5190 | NBPF9 | 5191 | DLEU7 | 5192 | LAMA1 |
| 5193 | HCG23 | 5194 | MCRS1 | 5195 | HES7 | 5196 | PLA2G5 |
| 5197 | FMNL2 | 5198 | GABBR1 | 5199 | MIR1915 | 5200 | CCDC142 |
| 5201 | PRRX1 | 5202 | CPEB1 | 5203 | AGPAT3 | 5204 | ARAP1 |
| 5205 | LINC00426 | 5206 | LPAR5 | 5207 | XCR1 | 5208 | LDB2 |
| 5209 | HAGHL | 5210 | IFNA6 | 5211 | LINC01721 | 5212 | TNXA |
| 5213 | CFAP91 | 5214 | SNORD24 | 5215 | SPATA31H1 | 5216 | TRUB1 |
| 5217 | KRT75 | 5218 | TMC1 | 5219 | RNF14 | 5220 | TNN |
| 5221 | TNRC6A | 5222 | MIR101-2 | 5223 | MIR3150B | 5224 | ENOPH1 |
| 5225 | LINC00322 | 5226 | MIR920 | 5227 | NIP7 | 5228 | PRPF4 |
| 5229 | KCNK6 | 5230 | UBE2M | 5231 | IL31 | 5232 | DPH6 |
| 5233 | GMFB | 5234 | MSX1 | 5235 | CIMAP3 | 5236 | DDI1 |
| 5237 | NIN | 5238 | SNCG | 5239 | SCAI | 5240 | MOB1A |
| 5241 | ETS1-AS1 | 5242 | KRT6A | 5243 | CDK5RAP1 | 5244 | YRDC |
| 5245 | GGCT | 5246 | MIR637 | 5247 | LINC00323 | 5248 | ZNF674 |
| 5249 | LINC01508 | 5250 | DUSP26 | 5251 | RCC2 | 5252 | CNTF |
| 5253 | TMEM86A | 5254 | TAPBPL | 5255 | C1QTNF3 | 5256 | STAU1 |
| 5257 | SLC5A7 | 5258 | DNAJB14 | 5259 | RBP3 | 5260 | C1orf53 |
| 5261 | ADAM1A | 5262 | HOXD13 | 5263 | KLK10 | 5264 | FBLL1 |
| 5265 | MIEF1 | 5266 | KIR2DS1 | 5267 | NUTM1 | 5268 | CENPC |
| 5269 | STX2 | 5270 | SEPTIN6 | 5271 | MMP25 | 5272 | NR2E3 |
| 5273 | SAMD8 | 5274 | BYSL | 5275 | DENND2D | 5276 | LINC02412 |
| 5277 | KCTD5 | 5278 | RAB18 | 5279 | ENSG00000286231 | 5280 | BNIP2 |
| 5281 | LINC01538 | 5282 | LINC01762 | 5283 | SETD3 | 5284 | CLCA1 |
| 5285 | lnc-JCAD-2 | 5286 | HOTAIRM1 | 5287 | PHPT1 | 5288 | CIR1 |
| 5289 | CHMP4B | 5290 | NLRX1 | 5291 | ZNF207 | 5292 | FBRS |
| 5293 | ADGRE2 | 5294 | CACNG5 | 5295 | CD27-AS1 | 5296 | C1GALT1 |
| 5297 | TMEM17 | 5298 | PCDH12 | 5299 | CCDC88B | 5300 | ENSG00000266469 |
| 5301 | BRINP1 | 5302 | AFG2B | 5303 | TUBG1 | 5304 | SNX22 |
| 5305 | LINC01684 | 5306 | AQP4 | 5307 | TALDO1P1 | 5308 | ANKLE1 |
| 5309 | DOLPP1 | 5310 | REPIN1 | 5311 | APOBEC3C | 5312 | GALNTL5 |
| 5313 | ADGRA3 | 5314 | LINC01134 | 5315 | MIR577 | 5316 | GTF3C1 |
| 5317 | TRPV2 | 5318 | LINC02384 | 5319 | SAMD9L | 5320 | CCR8 |
| 5321 | LPAR6 | 5322 | COPS3 | 5323 | KCTD12 | 5324 | ZWINT |
| 5325 | HLA-DQB1-AS1 | 5326 | ITGB8 | 5327 | LINC00596 | 5328 | LINC01204 |
| 5329 | OLFM1 | 5330 | SPRY2 | 5331 | FAM3B | 5332 | HCLS1 |
| 5333 | IFI44 | 5334 | TOMM34 | 5335 | RNF111 | 5336 | PDIA2 |
| 5337 | FBXW5 | 5338 | NPRL2 | 5339 | MSMP | 5340 | PRORP |
| 5341 | HCCS | 5342 | MRPL38 | 5343 | TNNC2 | 5344 | DDX60 |
| 5345 | P2RX5-TAX1BP3 | 5346 | BTG4 | 5347 | SLIT2 | 5348 | AIRN |
| 5349 | SNHG5 | 5350 | RCOR3 | 5351 | FCER2 | 5352 | MIR1301 |
| 5353 | P2RY4 | 5354 | UCKL1 | 5355 | FPR2 | 5356 | LRRC66 |
| 5357 | MRPL4 | 5358 | IL19 | 5359 | LOC126806211 | 5360 | LINC01679 |
| 5361 | ICAM4 | 5362 | IL25 | 5363 | BDKRB2 | 5364 | NLE1 |
| 5365 | FCHSD1 | 5366 | DCUN1D1 | 5367 | PAPOLA | 5368 | HYCC2 |
| 5369 | MICALL1 | 5370 | ROR2 | 5371 | WDR45 | 5372 | NUCB2 |
| 5373 | ARHGAP4 | 5374 | SNORA21 | 5375 | SNHG14 | 5376 | MIR376C |
| 5377 | ASPM | 5378 | ADAM8 | 5379 | LINC01798 | 5380 | ADAMTS19 |
| 5381 | HES3 | 5382 | CHIA | 5383 | RAB24 | 5384 | NGB |
| 5385 | MIR30C2 | 5386 | DOCK9 | 5387 | SNORA56 | 5388 | LPAR3 |
| 5389 | RABIF | 5390 | BTBD3 | 5391 | LOC130058658 | 5392 | CLEC6A |
| 5393 | MIR579 | 5394 | LY9 | 5395 | GPS2 | 5396 | SEMA3E |
| 5397 | WNT7A | 5398 | C4orf54 | 5399 | RRAGB | 5400 | KRT74 |
| 5401 | KIR2DS5 | 5402 | LOC126806252 | 5403 | CNDP1 | 5404 | NOSTRIN |
| 5405 | RABL2A | 5406 | UBL4B | 5407 | HSALNG0103961 | 5408 | MTCO3P1 |
| 5409 | MBOAT2 | 5410 | NEURL3 | 5411 | ADAM28 | 5412 | RASGRF2 |
| 5413 | GAD1 | 5414 | LOC124900380 | 5415 | KIR3DS1 | 5416 | RP1 |
| 5417 | KRT23 | 5418 | TRHDE | 5419 | B3GALT1 | 5420 | lnc-ZNF296-6 |
| 5421 | CD177 | 5422 | KIAA1549 | 5423 | C6orf120 | 5424 | DDX11 |
| 5425 | ATP2C1 | 5426 | ITGAD | 5427 | GRIN1 | 5428 | ZC3HAV1L |
| 5429 | PTPMT1 | 5430 | IPCEF1 | 5431 | MIR3178 | 5432 | CBLL1 |
| 5433 | WNT10B | 5434 | EIF1 | 5435 | SNORD49A | 5436 | LGALSL |
| 5437 | DNAH17 | 5438 | GABRA5 | 5439 | MIR4516 | 5440 | lnc-TM6SF2-1 |
| 5441 | PLA2R1 | 5442 | LGALS7B | 5443 | HS3ST1 | 5444 | ABHD12B |
| 5445 | FNDC11 | 5446 | PRM3 | 5447 | ZNF138 | 5448 | MGAT4C |
| 5449 | TMEM163 | 5450 | BAHD1 | 5451 | YIPF1 | 5452 | RSPO3 |
| 5453 | RCHY1 | 5454 | C19orf38 | 5455 | DR1 | 5456 | SCG2 |
| 5457 | AGPAT4 | 5458 | NPTXR | 5459 | ST2 | 5460 | MIR129-2 |
| 5461 | MIR1237 | 5462 | EXOSC4 | 5463 | IGHA1 | 5464 | ENSG00000280087 |
| 5465 | SPNS2 | 5466 | FLNC-AS1 | 5467 | AK9 | 5468 | RIPPLY2 |
| 5469 | GDF6 | 5470 | SLC6A13 | 5471 | CGB5 | 5472 | OPRD1 |
| 5473 | TMPRSS7 | 5474 | ENSG00000256433 | 5475 | TRNT1 | 5476 | FCRL3 |
| 5477 | ANKRD26 | 5478 | PGAP2 | 5479 | HCG22 | 5480 | BABAM2 |
| 5481 | AGAP2 | 5482 | KCTD17 | 5483 | SNORD42B | 5484 | TMEM116 |
| 5485 | LINC00929 | 5486 | LINC01020 | 5487 | TTTY7 | 5488 | RIN3 |
| 5489 | CCR10 | 5490 | VCF2 | 5491 | ENSG00000285163 | 5492 | MYO5C |
| 5493 | ENSG00000231748 | 5494 | ADAM7 | 5495 | KCNRG | 5496 | DINOL |
| 5497 | IFNLR1 | 5498 | ABHD8 | 5499 | TENT4B | 5500 | FOSB |
| 5501 | WDR75 | 5502 | DOCK1 | 5503 | CCNA1 | 5504 | GGN |
| 5505 | SULT2B1 | 5506 | TEX36 | 5507 | IFNA13 | 5508 | GABRB2 |
| 5509 | PDF | 5510 | SUCNR1 | 5511 | CDH26 | 5512 | FAM85B |
| 5513 | ASIP | 5514 | MIR602 | 5515 | LIPJ | 5516 | GAR1 |
| 5517 | KRT6B | 5518 | EIF2AK3-AS1 | 5519 | GXYLT2 | 5520 | MACIR |
| 5521 | HARBI1 | 5522 | PCARE | 5523 | MIR487A | 5524 | DRD3 |
| 5525 | KIR2DS3 | 5526 | DYNC1I1 | 5527 | RNU1-35P | 5528 | MIR3144 |
| 5529 | MIR194-2 | 5530 | CELA3B | 5531 | SPRY4 | 5532 | AP5Z1 |
| 5533 | NLRC5 | 5534 | ABHD4 | 5535 | BMX | 5536 | RP9 |
| 5537 | REM1 | 5538 | DMXL1 | 5539 | PARP14 | 5540 | SNORA24 |
| 5541 | TRDMT1 | 5542 | BCL6B | 5543 | TRUB2 | 5544 | TSPOAP1-AS1 |
| 5545 | CSKMT | 5546 | PAPOLG | 5547 | BCAT1 | 5548 | RNR4 |
| 5549 | ARMCX5 | 5550 | PRMT6 | 5551 | WAPL | 5552 | RBPJL |
| 5553 | TPT1-AS1 | 5554 | DOK1 | 5555 | SCARNA10 | 5556 | MIR376A2 |
| 5557 | MRPS14 | 5558 | HERPUD2 | 5559 | WNT2B | 5560 | SHOX2 |
| 5561 | SNORD124 | 5562 | HLA-DRB3 | 5563 | ZNF697 | 5564 | NUF2 |
| 5565 | MIR520F | 5566 | SNORD134 | 5567 | SELENOM | 5568 | SLCO1C1 |
| 5569 | PHAX | 5570 | RPL24P5 | 5571 | SAMD12 | 5572 | ACER3 |
| 5573 | MIR484 | 5574 | CXCR2P1 | 5575 | STXBP5L | 5576 | PXN-AS1 |
| 5577 | CFAP58-DT | 5578 | RPS15AP19 | 5579 | RPL26P27 | 5580 | KDM4D |
| 5581 | RPL36A-HNRNPH2 | 5582 | PPIAL4G | 5583 | RAB29 | 5584 | PNMT |
| 5585 | WDR18 | 5586 | ALMS1-IT1 | 5587 | ZBTB9 | 5588 | TIMM29 |
| 5589 | MIR1291 | 5590 | ZNF397 | 5591 | GUCA1B | 5592 | MIR646HG |
| 5593 | SVIL-AS1 | 5594 | WDR27 | 5595 | GAPDH-DT | 5596 | RN7SL688P |
| 5597 | SCAMP4 | 5598 | BMP8B | 5599 | CDK18 | 5600 | HSALNG0068330 |
| 5601 | MRPS21 | 5602 | TILAM | 5603 | MYLK-AS1 | 5604 | POLL |
| 5605 | RASGRP4 | 5606 | OLIG3 | 5607 | TAF4B | 5608 | MIR1182 |
| 5609 | SLC5A3 | 5610 | APOBEC3B | 5611 | POGLUT3 | 5612 | FOXC2-AS1 |
| 5613 | ITGBL1 | 5614 | LINC00649 | 5615 | ENSG00000262020 | 5616 | HSALNG0087578 |
| 5617 | MIR138-2 | 5618 | STK32B | 5619 | GALK2 | 5620 | DLK2 |
| 5621 | CRB1 | 5622 | EIF2A | 5623 | SHQ1 | 5624 | SEC61G |
| 5625 | COL11A1 | 5626 | LOC130009836 | 5627 | LOC130009837 | 5628 | EFR3A |
| 5629 | NET1 | 5630 | MIR521-1 | 5631 | ENSG00000255046 | 5632 | CYLD-AS1 |
| 5633 | IMPG2 | 5634 | CDKN2D | 5635 | RARRES1 | 5636 | SNORD118 |
| 5637 | TMEM100 | 5638 | PALM | 5639 | LOC117307477 | 5640 | SLC9A5 |
| 5641 | RPS19BP1 | 5642 | CRISPLD2 | 5643 | CRHR1 | 5644 | PIGH |
| 5645 | USP45 | 5646 | VPS72 | 5647 | ABHD1 | 5648 | IL20RA |
| 5649 | TMEM175 | 5650 | MTHFSD | 5651 | lnc-ZNF101-1 | 5652 | GPR141 |
| 5653 | LOC105371081 | 5654 | SERPINB7 | 5655 | B3GALT9 | 5656 | SEC31B |
| 5657 | FUNDC1 | 5658 | CCL24 | 5659 | LIX1L | 5660 | SLC24A4 |
| 5661 | RNU6-908P | 5662 | ATP10B | 5663 | NOX5 | 5664 | SNORA5C |
| 5665 | SLC16A4 | 5666 | ZNF382 | 5667 | SNORA80E | 5668 | TULP2 |
| 5669 | PPIAL4E | 5670 | HSALNG0144148 | 5671 | lnc-TMEM268-4 | 5672 | CADM2 |
| 5673 | IL1RL2 | 5674 | MRPL16 | 5675 | PHETA1 | 5676 | lnc-EXOC3L4-1 |
| 5677 | PDCD6-AHRR | 5678 | INA | 5679 | ASB4 | 5680 | MIR218-2 |
| 5681 | SPRR1B | 5682 | CDT1 | 5683 | ENSG00000228509 | 5684 | PPIAP6 |
| 5685 | PPP1R16B | 5686 | CEMIP | 5687 | LINC00299 | 5688 | ID2-AS1 |
| 5689 | DGKG | 5690 | GPAT2 | 5691 | ABHD16B | 5692 | SCARNA1 |
| 5693 | H2BC26 | 5694 | GABRB3 | 5695 | UBE2S | 5696 | MUC22 |
| 5697 | MIR758 | 5698 | SCART1 | 5699 | LRRC1 | 5700 | SNORA62 |
| 5701 | SLC35D2 | 5702 | PRSS3 | 5703 | LINC00494 | 5704 | MTCL1 |
| 5705 | RNU6-959P | 5706 | LOC124906252 | 5707 | ABHD14A | 5708 | PDYN |
| 5709 | CMTM7 | 5710 | ZMYM5 | 5711 | IFN1@ | 5712 | CLIC3 |
| 5713 | KRT37 | 5714 | MIR765 | 5715 | MIR874 | 5716 | MRPL50 |
| 5717 | SAMD9 | 5718 | AWAT1 | 5719 | LOC105371762 | 5720 | LINC03135 |
| 5721 | POTEG | 5722 | DEUP1 | 5723 | POTEM | 5724 | TIGD4 |
| 5725 | POU2AF3 | 5726 | ATP6V1F | 5727 | LMBR1 | 5728 | LMBRD2 |
| 5729 | TEX48 | 5730 | THSD7A | 5731 | SHOX | 5732 | TMTC4 |
| 5733 | FAM81B | 5734 | RPS4Y1 | 5735 | PNISR-AS1 | 5736 | ADD3-AS1 |
| 5737 | GNPDA1 | 5738 | SFTA2 | 5739 | NGF-AS1 | 5740 | LINC03002 |
| 5741 | P2RY11 | 5742 | MUC3B | 5743 | FOXS1 | 5744 | GBP5 |
| 5745 | SNORA33 | 5746 | DUS4L | 5747 | ATP9B | 5748 | LRRC43 |
| 5749 | MIR608 | 5750 | DHX35 | 5751 | UBE2B | 5752 | HSALNG0068329 |
| 5753 | SCRG1 | 5754 | TSR2 | 5755 | ENSG00000306274 | 5756 | CYSLTR1 |
| 5757 | LOC130068886 | 5758 | SLC22A23 | 5759 | P2RY14 | 5760 | CEP57 |
| 5761 | AAGAB | 5762 | LOC101928277 | 5763 | SMC6 | 5764 | MIR3120 |
| 5765 | GGT3P | 5766 | LINC00462 | 5767 | MIR486-2 | 5768 | LOC101928994 |
| 5769 | KDM4A-AS1 | 5770 | MTNR1B | 5771 | GRIN2A | 5772 | MIR1269A |
| 5773 | SCIN | 5774 | MS4A2 | 5775 | MYOZ1 | 5776 | CREBL2 |
| 5777 | MIR1262 | 5778 | TMEM39A | 5779 | RNU6ATAC | 5780 | EFCAB11 |
| 5781 | NLRP5 | 5782 | DOCK3 | 5783 | SMIM23 | 5784 | LINC01307 |
| 5785 | MORC4 | 5786 | SMOC2 | 5787 | SIX3 | 5788 | DLGAP1-AS1 |
| 5789 | SNHG18 | 5790 | LBX1 | 5791 | MIR5705 | 5792 | ART3 |
| 5793 | MIR889 | 5794 | SNORA7A | 5795 | UBA3 | 5796 | MIR6809 |
| 5797 | SNHG32 | 5798 | SSTR4 | 5799 | ELOVL7 | 5800 | RAB11FIP2 |
| 5801 | MIR5703 | 5802 | ENSG00000285040 | 5803 | LOC124903741 | 5804 | SRRD |
| 5805 | MPPE1 | 5806 | APOBEC3H | 5807 | ANKAR | 5808 | NDST4 |
| 5809 | lnc-HLA-C-2 | 5810 | HSALNG0133406 | 5811 | WDR62 | 5812 | GPR137 |
| 5813 | MIR301B | 5814 | MIR647 | 5815 | SNORA5A | 5816 | ALX4 |
| 5817 | FAM124B | 5818 | SIM1 | 5819 | FSCN2 | 5820 | ASCL1 |
| 5821 | CRMA | 5822 | DUS4L-BCAP29 | 5823 | LINC01082 | 5824 | CAPSL-DT |
| 5825 | LINC02132 | 5826 | ENSG00000269667 | 5827 | ENSG00000275106 | 5828 | LOC105377347 |
| 5829 | lnc-TNPO3-9 | 5830 | COL4A6 | 5831 | MIR1227 | 5832 | ENSG00000298396 |
| 5833 | HSALNG0049249 | 5834 | ENSG00000303513 | 5835 | KIF18A | 5836 | SCARNA4 |
| 5837 | COLCA1 | 5838 | MIR9-1HG | 5839 | TRAPPC2 | 5840 | CNTN5 |
| 5841 | SNORA5B | 5842 | EFCAB14-AS1 | 5843 | FGF13 | 5844 | PDE6G |
| 5845 | DPCD | 5846 | PIGC | 5847 | TM7SF3 | 5848 | RILPL2 |
| 5849 | OR4C11 | 5850 | OR4P4 | 5851 | BACH2 | 5852 | GABRA2 |
| 5853 | CAVIN4 | 5854 | ENSG00000285647 | 5855 | lnc-DDX60L-3 | 5856 | ENSG00000298426 |
| 5857 | LOC107986198 | 5858 | NONHSAG026010.2 | 5859 | piR-56133-114 | 5860 | ENSG00000248969 |
| 5861 | HSALNG0087581 | 5862 | HSALNG0102088 | 5863 | piR-38580-086 | 5864 | FOXR1 |
| 5865 | SNORA6 | 5866 | MIR4715 | 5867 | GCOM1 | 5868 | SIM2 |
| 5869 | ENSG00000293518 | 5870 | PODNL1 | 5871 | EXOSC7 | 5872 | TTC31 |
| 5873 | HTR1B | 5874 | SHCBP1 | 5875 | RAB8B | 5876 | LINC00635 |
| 5877 | LINC01194 | 5878 | ZC2HC1C | 5879 | SNORA67 | 5880 | NELL1 |
| 5881 | NRROS | 5882 | MYOCD | 5883 | GAD2 | 5884 | NBPF10 |
| 5885 | HAGLROS | 5886 | JKAMP | 5887 | CSTL1 | 5888 | STARD4-AS1 |
| 5889 | MIR219A2 | 5890 | APOBEC3A | 5891 | SLC35D3 | 5892 | SCHIP1 |
| 5893 | MIR3200 | 5894 | LINC00987 | 5895 | LOC124900181 | 5896 | HSALNG0076986 |
| 5897 | BNC2 | 5898 | ADAMTS12 | 5899 | ENSG00000300654 | 5900 | HSALNG0073924 |
| 5901 | LOC126805749 | 5902 | IFFO1 | 5903 | RFX4 | 5904 | BMP15 |
| 5905 | LINC01857 | 5906 | CAMTA2 | 5907 | EPHA7 | 5908 | ENSG00000293569 |
| 5909 | EVA1C | 5910 | lnc-FAM84B-8 | 5911 | DNASE2B | 5912 | SLC26A8 |
| 5913 | ALMS1P1 | 5914 | LARP6 | 5915 | RLN3 | 5916 | ZNF787 |
| 5917 | ECT2L | 5918 | ZBTB12 | 5919 | MIR6891 | 5920 | NACAP8 |
| 5921 | lnc-CRYAA-1 | 5922 | HSALNG0049258 | 5923 | piR-47864 | 5924 | piR-52740 |
| 5925 | HSALNG0076987 | 5926 | SEMA6D | 5927 | MIR1181 | 5928 | RPL13P6 |
| 5929 | SNORD29 | 5930 | NOVA2 | 5931 | TUBE1 | 5932 | PIF1 |
| 5933 | HSALNG0143317 | 5934 | HSALNG0036091 | 5935 | HSALNG0087584 | 5936 | lnc-IFT80-3 |
| 5937 | ANAPC4 | 5938 | CHD9NB | 5939 | LINC00534 | 5940 | LDLR-AS1 |
| 5941 | MIR3591 | 5942 | AP1G2 | 5943 | BHLHA9 | 5944 | PAPOLB |
| 5945 | MIR376B | 5946 | CALCR | 5947 | CCDC93 | 5948 | FAR2 |
| 5949 | PUS3 | 5950 | STRN4 | 5951 | RLN2 | 5952 | ABHD13 |
| 5953 | DLEU7-AS1 | 5954 | CENPI | 5955 | PEX5L | 5956 | MPIG6B |
| 5957 | DEFB104A | 5958 | CFM1 | 5959 | LOC108491823 | 5960 | LOC111674464 |
| 5961 | LOC111674465 | 5962 | LOC111674466 | 5963 | LOC111674470 | 5964 | LOC111674471 |
| 5965 | LOC111674473 | 5966 | LOC111674474 | 5967 | LOC111674476 | 5968 | LOC111674478 |
| 5969 | LOC111674479 | 5970 | LOC113219471 | 5971 | LOC113523647 | 5972 | LOC113604967 |
| 5973 | LOC113633875 | 5974 | LOC113633876 | 5975 | LOC113664107 | 5976 | KCNIP4 |
| 5977 | HSALNG0038430 | 5978 | ENSG00000298211 | 5979 | NGID-105180392 | 5980 | GFY |
| 5981 | ZBTB11 | 5982 | ANKK1 | 5983 | SVEP1 | 5984 | HOXC6 |
| 5985 | RNU6-474P | 5986 | MIR548Z | 5987 | AP1S2 | 5988 | LIPK |
| 5989 | FZD1 | 5990 | LOC101927082 | 5991 | MYO7B | 5992 | SNORD56 |
| 5993 | LOC124903416 | 5994 | MSL2 | 5995 | LINC00824 | 5996 | RNU7-130P |
| 5997 | BMP8A | 5998 | PAPPA-AS1 | 5999 | LGI2 | 6000 | HLA-DQB3 |
| 6001 | lnc-IL12RB2-1 | 6002 | HSALNG0041149 | 6003 | lnc-HLA-DQB1-2 | 6004 | ENSG00000302994 |
| 6005 | piR-33614-202 | 6006 | piR-44610-006 | 6007 | TRIM58 | 6008 | LOC126860125 |
| 6009 | DLX5 | 6010 | TRA | 6011 | P2RX2 | 6012 | ZSCAN5A |
| 6013 | FRZB | 6014 | DEFA1 | 6015 | SNORA10B | 6016 | ZNF334 |
| 6017 | LAYN | 6018 | ADAMTS5 | 6019 | ENSG00000276861 | 6020 | HHAT |
| 6021 | EFCAB14 | 6022 | MIR1275 | 6023 | WDFY4 | 6024 | MIR4521 |
| 6025 | IL17D | 6026 | PPP1R9A | 6027 | DELEC1 | 6028 | TSTD3 |
| 6029 | VWA7 | 6030 | RGS4 | 6031 | SNORA66 | 6032 | MIR618 |
| 6033 | GDPD1 | 6034 | ENSG00000259242 | 6035 | LINC01648 | 6036 | ELOCP20 |
| 6037 | MEMO1P2 | 6038 | RNA5SP112 | 6039 | lnc-MBOAT7-2 | 6040 | HSALNG0127481 |
| 6041 | piR-41195-080 | 6042 | piR-59297-626 | 6043 | HSALNG0125988 | 6044 | USP30 |
| 6045 | MAP1LC3C | 6046 | MIR1260A | 6047 | lnc-CCDC167-2 | 6048 | RXFP1 |
| 6049 | HTR1A | 6050 | GOLGA8H | 6051 | ENSG00000296370 | 6052 | LOC101927741 |
| 6053 | LINC01428 | 6054 | LOC102723878 | 6055 | ENSG00000271553 | 6056 | ZSCAN4 |
| 6057 | RBM24 | 6058 | HDDC2 | 6059 | MIR1238 | 6060 | YPEL5 |
| 6061 | UNC5A | 6062 | HVBS7 | 6063 | HVBS8 | 6064 | OR4N2 |
| 6065 | DIRC1 | 6066 | SRP54-AS1 | 6067 | FBXW4 | 6068 | LINC00278 |
| 6069 | SEPTIN11 | 6070 | SNORA15 | 6071 | PKP4-AS1 | 6072 | ENSG00000225000 |
| 6073 | lnc-KCNQ2-2 | 6074 | TMEM203 | 6075 | LMOD3 | 6076 | MIR3162 |
| 6077 | ARIH2P1 | 6078 | AP5M1 | 6079 | DRC12 | 6080 | IQCJ-SCHIP1 |
| 6081 | WAKMAR2 | 6082 | LOC105371082 | 6083 | VPS35L | 6084 | LOC129389837 |
| 6085 | AP5S1 | 6086 | MYMK | 6087 | ENSG00000207024 | 6088 | IL20 |
| 6089 | TREHP1 | 6090 | LSM14B | 6091 | MIR943 | 6092 | HRH3 |
| 6093 | DDX53 | 6094 | lnc-CRK-3 | 6095 | lnc-TRIM27-18 | 6096 | PNLDC1 |
| 6097 | PRRT4 | 6098 | SOHLH2 | 6099 | TPTE2 | 6100 | BTF3P13 |
| 6101 | lnc-ITPR3-2 | 6102 | lnc-IL12A-2 | 6103 | lnc-HLA-DRA-3 | 6104 | piR-39858-805 |
| 6105 | ENSG00000299769 | 6106 | KCTD15 | 6107 | ENSG00000255422 | 6108 | ADCY1 |
| 6109 | SVIP | 6110 | LOC126860124 | 6111 | KRT8P34 | 6112 | lnc-ZPBP2-6 |
| 6113 | HSALNG0049436 | 6114 | HSALNG0061263 | 6115 | lnc-SCNN1A-2 | 6116 | piR-61240-151 |
| 6117 | CENATAC-DT | 6118 | FAR1 | 6119 | SNORD50A | 6120 | CSGALNACT1 |
| 6121 | MEG8 | 6122 | MIR603 | 6123 | ENSG00000199668 | 6124 | PCSK4 |
| 6125 | DOCK5 | 6126 | CMTM2 | 6127 | KCNK2 | 6128 | ACER1 |
| 6129 | SNORD45C | 6130 | HOATZ | 6131 | lnc-CD58-2 | 6132 | SPRR2A |
| 6133 | LOC101928373 | 6134 | HSALNG0030237 | 6135 | PAMR1 | 6136 | LINC02713 |
| 6137 | LINC01756 | 6138 | LINC01718 | 6139 | RPL13AP7 | 6140 | ATP5MGP4 |
| 6141 | RNA5SP166 | 6142 | RNA5SP489 | 6143 | RNU6-187P | 6144 | OTX2P1 |
| 6145 | EEF1A1P20 | 6146 | MTCO1P38 | 6147 | lnc-TM6SF2-2 | 6148 | HSALNG0131408 |
| 6149 | LOC105370163 | 6150 | LOC107986195 | 6151 | ENSG00000302709 | 6152 | HSALNG0068856 |
| 6153 | LOC105377483 | 6154 | piR-57133-376 | 6155 | SCARNA13 | 6156 | OR4K1 |
| 6157 | OR4K2 | 6158 | OR4S2 | 6159 | OR4C6 | 6160 | OR4K5 |
| 6161 | OR4Q3 | 6162 | OR11H1 | 6163 | OR11H12 | 6164 | LINC00226 |
| 6165 | ARHGAP19-SLIT1 | 6166 | lnc-MBNL2-3 | 6167 | FBXL2 | 6168 | RAB41 |
| 6169 | FRMPD2 | 6170 | ZBTB43 | 6171 | CCDC152 | 6172 | ENSG00000298543 |
| 6173 | SCAMP1 | 6174 | LOC124629375 | 6175 | MIR5188 | 6176 | ENSG00000245869 |
| 6177 | MIR3662 | 6178 | NAA11 | 6179 | TM2D3 | 6180 | MT-TR |
| 6181 | UTS2B | 6182 | MIR3183 | 6183 | MIR7155 | 6184 | MIR599 |
| 6185 | MIR875 | 6186 | LOC130055323 | 6187 | LOC130055324 | 6188 | AP1M2 |
| 6189 | KCNK4-CATSPERZ | 6190 | LINC02040 | 6191 | HSALNG0149142 | 6192 | piR-48759-385 |
| 6193 | KIF21B | 6194 | CELSR3 | 6195 | WNT10A | 6196 | GDF5 |
| 6197 | UXS1 | 6198 | GRIN2B | 6199 | MIR4721 | 6200 | RPF2 |
| 6201 | MIR6855 | 6202 | TMEM88 | 6203 | ENSG00000222529 | 6204 | SLC26A5 |
| 6205 | SPCS1 | 6206 | MIR4664 | 6207 | MIR3198-2 | 6208 | RBMY1B |
| 6209 | UBLCP1 | 6210 | SNORA36A | 6211 | TSPAN12 | 6212 | CATSPERZ |
| 6213 | NEMP2-DT | 6214 | piR-43105-342 | 6215 | SSTR5-AS1 | 6216 | MAMDC4 |
| 6217 | WLS | 6218 | MAPT-IT1 | 6219 | TBC1D30 | 6220 | SCARNA23 |
| 6221 | ATP1B4 | 6222 | ICAM5 | 6223 | LRRC3C | 6224 | IL12B-AS1 |
| 6225 | LOC105377683 | 6226 | RNU6-565P | 6227 | HSALNG0094037 | 6228 | EFR3B |
| 6229 | CAPN9 | 6230 | SNORA7B | 6231 | C16orf78 | 6232 | MIR1972-1 |
| 6233 | LOC110806262 | 6234 | MIR4635 | 6235 | MIR7974 | 6236 | MIR4465 |
| 6237 | SNORD52 | 6238 | MIR890 | 6239 | MFSD6 | 6240 | LINC03004 |
| 6241 | LOC124900868 | 6242 | lnc-SFRP4-2 | 6243 | HSALNG0088101 | 6244 | ARHGAP22 |
| 6245 | GOLGA8R | 6246 | MIR4520-2 | 6247 | BEX2 | 6248 | CENPCP1 |
| 6249 | SPATA31A3 | 6250 | CHI3L2 | 6251 | MIR552 | 6252 | TRH |
| 6253 | LINC01237 | 6254 | TET2-AS1 | 6255 | CFAP263 | 6256 | LINC01381 |
| 6257 | DOCK6-AS1 | 6258 | TBX15 | 6259 | lnc-ADCY10-1 | 6260 | lnc-UBC-3 |
| 6261 | lnc-HSD17B10-3 | 6262 | REXO1L1P | 6263 | UOX | 6264 | NPAP1 |
| 6265 | MIR3648-2 | 6266 | ENSG00000284825 | 6267 | COX7CP3 | 6268 | EPHX4 |
| 6269 | UBL3 | 6270 | SGIP1 | 6271 | TPI1P2 | 6272 | ENSG00000267159 |
| 6273 | ENSG00000299144 | 6274 | MIR4508 | 6275 | NUDT10 | 6276 | MIR548L |
| 6277 | MIR3195 | 6278 | MIR298 | 6279 | ARLN | 6280 | LPCAT4 |
| 6281 | GALR2 | 6282 | MIR623 | 6283 | CASD1 | 6284 | FCRL4 |
| 6285 | MIR518B | 6286 | IQCJ-SCHIP1-AS1 | 6287 | LINC02723 | 6288 | LRRC37A15P |
| 6289 | ENSG00000224163 | 6290 | lnc-FAM109A-1 | 6291 | GPRASP1 | 6292 | CDON |
| 6293 | HOPX | 6294 | GPR65 | 6295 | SNORA30 | 6296 | LINC02670 |
| 6297 | LINC01735 | 6298 | LINC02290 | 6299 | LINC02663 | 6300 | LINC01751 |
| 6301 | RPS4XP19 | 6302 | RNU6-603P | 6303 | RPL21P110 | 6304 | RPL18AP17 |
| 6305 | MTCH2P4 | 6306 | lnc-IFT80-5 | 6307 | HSALNG0030208-002 | 6308 | HSALNG0049401 |
| 6309 | piR-39858-768 | 6310 | piR-43105-496 | 6311 | MIR575 | 6312 | MIR658 |
| 6313 | ENPP7P13 | 6314 | RPL34P26 | 6315 | LOC124902020 | 6316 | DOCK10 |
| 6317 | LOC123002309 | 6318 | LOC123002310 | 6319 | AVPR1B | 6320 | MIR7704 |
| 6321 | BRICD5 | 6322 | MIR543 | 6323 | DAZ1 | 6324 | ZFY |
| 6325 | LOC107652445 | 6326 | HOXC13 | 6327 | MIR3198-1 | 6328 | OR51B2 |
| 6329 | EBLN1 | 6330 | ENSG00000249141 | 6331 | ENSG00000242162 | 6332 | ENSG00000306406 |
| 6333 | HSALNG0103272 | 6334 | LOC124903364 | 6335 | NRAP | 6336 | RNF151 |
| 6337 | MIR4281 | 6338 | MIR3157 | 6339 | RBMY1F | 6340 | RBMY1J |
| 6341 | RBMY1D | 6342 | RBMY1E | 6343 | MIR1255A | 6344 | SFTA1P |
| 6345 | ST8SIA4 | 6346 | LOC124900242 | 6347 | ENSG00000224794 | 6348 | ENSG00000255176 |
| 6349 | ENSG00000201566 | 6350 | ENSG00000272980 | 6351 | LOC124905119 | 6352 | ENSG00000299593 |
| 6353 | HSALNG0068510 | 6354 | LOC130004273 | 6355 | MIR492 | 6356 | MIR1272 |
| 6357 | BAGE | 6358 | MIR5787 | 6359 | GOLGA8T | 6360 | MIR1199 |
| 6361 | MIR604 | 6362 | GJB7 | 6363 | MIR2861 | 6364 | RPS10P14 |
| 6365 | ENSG00000293497 | 6366 | ENSG00000289191 | 6367 | MIR655 | 6368 | OR2AG1 |
| 6369 | CYP26C1 | 6370 | EXOC6B | 6371 | NUPR2 | 6372 | LOC110740340 |
| 6373 | MIR4668 | 6374 | OR8J1 | 6375 | lnc-ATP1A1-1 | 6376 | HSALNG0061260 |
| 6377 | LOC105369519 | 6378 | CLRN1-AS1 | 6379 | OXGR1 | 6380 | MIR4519 |
| 6381 | ASB11 | 6382 | TRPV3 | 6383 | CCDC169-SOHLH2 | 6384 | KRT18P39 |
| 6385 | lnc-CLEC16A-8 | 6386 | HSALNG0088098 | 6387 | HSALNG0088099 | 6388 | LOC124902788 |
| 6389 | lnc-FLI1-5 | 6390 | LOC124902789 | 6391 | MIR620 | 6392 | MIR302F |
| 6393 | ABITRAM | 6394 | GOLGA8Q | 6395 | KCTD8 | 6396 | MIR4513 |
| 6397 | MIR4286 | 6398 | IL21-AS1 | 6399 | KRT8P46 | 6400 | ENSG00000286913 |
| 6401 | ENSG00000250321 | 6402 | ENSG00000305209 | 6403 | HSALNG0024501 | 6404 | lnc-ICAM3-1 |
| 6405 | MIR548I2 | 6406 | MIR6727 | 6407 | TSHR-AS1 | 6408 | SNORD13 |
| 6409 | CPB1 | 6410 | LINC00853 | 6411 | MIR1825 | 6412 | LOC106627982 |
| 6413 | MIR595 | 6414 | LINC01793 | 6415 | IGAN1 | 6416 | IGAN2 |
| 6417 | LINC02273 | 6418 | VIM-AS1 | 6419 | ATXN2-AS | 6420 | RNU6-376P |
| 6421 | PTPN11P3 | 6422 | lnc-PRDM1-3 | 6423 | IQCJ-SCHIP1-AS1-001 | 6424 | lnc-CHP2-1 |
| 6425 | lnc-BPHL-10 | 6426 | lnc-DDX6-4 | 6427 | lnc-PDE4A-4-001 | 6428 | lnc-PDE4A-4-002 |
| 6429 | piR-61580-582 | 6430 | MIR521-2 | 6431 | OSGEPL1 | 6432 | ELF2P2 |
| 6433 | GSPT2 | 6434 | LINC00312 | 6435 | MIR4315-1 | 6436 | NKX2-4 |
| 6437 | SLC25A21 | 6438 | HHIP-AS1 | 6439 | GLRA3 | 6440 | LINC02098 |
| 6441 | MIR548AC | 6442 | IGBP1P1 | 6443 | ENSG00000269919 | 6444 | LINC01845 |
| 6445 | ENSG00000280216 | 6446 | lnc-DENND1B-6 | 6447 | HSALNG0087098 | 6448 | HSALNG0087579 |
| 6449 | HSALNG0135499 | 6450 | lnc-BTG4-3 | 6451 | lnc-PLEKHG6-5 | 6452 | piR-43304 |
| 6453 | piR-61101-324 | 6454 | ENSG00000299161 | 6455 | ENSG00000304360 | 6456 | ENSG00000306318 |
| 6457 | HSALNG0006338 | 6458 | LOC105378925 | 6459 | piR-56133-186 | 6460 | piR-57135-050 |
| 6461 | piR-59297-540 | 6462 | piR-60146-129 | 6463 | SCARNA2 | 6464 | ENSG00000202198 |
| 6465 | MIR548X | 6466 | RNU11 | 6467 | POPDC2 | 6468 | MIR6886 |
| 6469 | MIR374C | 6470 | MIR1234 | 6471 | MIR3135B | 6472 | MIR548X2 |
| 6473 | ZSCAN5B | 6474 | MIR651 | 6475 | ZNF2 | 6476 | C2orf81 |
| 6477 | ENSG00000276376 | 6478 | LINC01232 | 6479 | MIR3714 | 6480 | CFAP144P2 |
| 6481 | SNRPGP8 | 6482 | lnc-SCNN1A-1 | 6483 | lnc-GTF3C1-3 | 6484 | HSALNG0055144 |
| 6485 | HSALNG0109573 | 6486 | SNODBsnoDB1529 | 6487 | lnc-PRDX5-1 | 6488 | lnc-TNP2-1 |
| 6489 | piR-50852-124 | 6490 | piR-51137-090 | 6491 | ENSG00000302760 | 6492 | HSALNG0021124 |
| 6493 | HSALNG0028322 | 6494 | HSALNG0109575 | 6495 | HSALNG0116265 | 6496 | HSALNG0116280 |
| 6497 | HSALNG0143084 | 6498 | piR-57929 | 6499 | LOC105371800 | 6500 | lnc-PSMG4-3 |
| 6501 | LINC01842 | 6502 | MIR4463 | 6503 | MIR4491 | 6504 | ZNF676 |
| 6505 | SCARNA8 | 6506 | GPA33 | 6507 | PAGE1 | 6508 | HHT4 |
| 6509 | IL17C | 6510 | GABRA1 | 6511 | MIR887 | 6512 | HNP1 |
| 6513 | CDK5R2 | 6514 | MIR4677 | 6515 | PDZPH1P | 6516 | RNU4ATAC4P |
| 6517 | RNU6-1091P | 6518 | KARS1P3 | 6519 | RPL17P28 | 6520 | H3P5 |
| 6521 | ENSG00000288064 | 6522 | MIR10399 | 6523 | lnc-HEMGN-4 | 6524 | lnc-IGSF3-2 |
| 6525 | HSALNG0032205 | 6526 | LOC124902226 | 6527 | lnc-BTNL2-2 | 6528 | lnc-CCDC88B-1 |
| 6529 | HSALNG0094047 | 6530 | HSALNG0097109 | 6531 | LOC124900337 | 6532 | LOC124900645 |
| 6533 | NONHSAG041785.2 | 6534 | lnc-ALK-5 | 6535 | lnc-FAM109A-5 | 6536 | lnc-ICAM3-2 |
| 6537 | lnc-LBH-3 | 6538 | lnc-NEMP2-7 | 6539 | lnc-ZC3HAV1L-1 | 6540 | miTC10 |
| 6541 | piR-36588-116 | 6542 | piR-39099-141 | 6543 | HSALNG0082008 | 6544 | CLEC4D |
| 6545 | MIR4717 | 6546 | PAGE3 | 6547 | RABGAP1L-DT | 6548 | lnc-EPC1-4 |
| 6549 | GABRA6 | 6550 | MIR3123 | 6551 | RNU4-5P | 6552 | LRRC14B |
| 6553 | PWAR5 | 6554 | lnc-ZEB2-19 | 6555 | COPS5P1 | 6556 | TRK-TTT15-1 |
| 6557 | MIR448 | 6558 | OPN1MW2 | 6559 | LOC110283621 | 6560 | SCARNA9 |
| 6561 | MIR6747 | 6562 | MIR412 | 6563 | MIR4756 | 6564 | GAGE1 |
| 6565 | LOC111255642 | 6566 | MAGEB2 | 6567 | MIR4464 | 6568 | SNORA49 |
| 6569 | SSX5 | 6570 | LINC01711 | 6571 | SCGB3A2 | 6572 | MIR626 |
| 6573 | TRN-GTT4-1 | 6574 | LOC112679202 | 6575 | LOC129937077 | 6576 | LOC129937078 |
| 6577 | LOC129937076 | 6578 | MIR323B | 6579 | MIR664B | 6580 | CRISP3 |
| 6581 | LELP1 | 6582 | MIR5193 | 6583 | DMP1-AS1 | 6584 | MIR764 |
| 6585 | MIR7706 | 6586 | MIR4428 | 6587 | MIR6881 | 6588 | LOC111255645 |
| 6589 | LINC00297 | 6590 | RPL19P8 | 6591 | LOC109286563 | 6592 | LOC129934277 |
| 6593 | FMO1 | 6594 | FMO4 | 6595 | LCO | 6596 | PBC1 |
| 6597 | LEAP2 | 6598 | COX7A2 | 6599 | RINT1 | 6600 | FLD1 |
| 6601 | NAIC | 6602 | SLC25A6 | 6603 | Stap-coc pbp2 | 6604 | Bact 16S rRNA |
| 6605 | P4HA1 |  |  |  |  |  |  |

**Table S2.** Overlapping targets (n = 708) between SPD-related targets and liver fibrosis–associated targets.

| **No.** | **Target** | **No.** | **Target** | **No.** | **Target** | **No.** | **Target** |
| --- | --- | --- | --- | --- | --- | --- | --- |
| 1 | CA2 | 2 | CA7 | 3 | CA1 | 4 | CA6 |
| 5 | CA14 | 6 | CA9 | 7 | CA4 | 8 | CA5B |
| 9 | CA5A | 10 | CASP2 | 11 | CA3 | 12 | CA13 |
| 13 | CHRNB2 | 14 | CHRNA4 | 15 | CHRNB4 | 16 | CHRNA3 |
| 17 | CHRNA7 | 18 | ODC1 | 19 | ALPL | 20 | SLC5A7 |
| 21 | DNM1 | 22 | ADH1A | 23 | CHRM4 | 24 | FYN |
| 25 | ADRA2C | 26 | CHRM5 | 27 | EGFR | 28 | CHRM2 |
| 29 | CHRM1 | 30 | ACHE | 31 | SLC6A2 | 32 | CHRM3 |
| 33 | SIGMAR1 | 34 | CYP2D6 | 35 | HTR6 | 36 | ADRA2B |
| 37 | NOS1 | 38 | RCOR1 | 39 | PAOX | 40 | HRH2 |
| 41 | DRD2 | 42 | HRH1 | 43 | ADRA1B | 44 | DRD3 |
| 45 | OPRK1 | 46 | HTR5A | 47 | CA12 | 48 | MAOA |
| 49 | MAOB | 50 | HTR2C | 51 | METAP1 | 52 | ADH1C |
| 53 | PLA2G2A | 54 | PLA2G1B | 55 | SLC6A4 | 56 | PRMT6 |
| 57 | CARM1 | 58 | INMT | 59 | LCK | 60 | HRH3 |
| 61 | KDM1A | 62 | KCNH2 | 63 | DPP7 | 64 | SLC6A3 |
| 65 | ACTN2 | 66 | ACTN3 | 67 | ADIPOQ | 68 | ADRB1 |
| 69 | ADRB2 | 70 | AGRP | 71 | AKT1 | 72 | AKT2 |
| 73 | ANKRD2 | 74 | ANXA5 | 75 | AOC1 | 76 | APP |
| 77 | ARG1 | 78 | ASGR1 | 79 | ASIC1 | 80 | ATF6 |
| 81 | ATP2A1 | 82 | BAX | 83 | BCL2 | 84 | CASP3 |
| 85 | CASP7 | 86 | CASP8 | 87 | CASP9 | 88 | CASR |
| 89 | CCL2 | 90 | CCNA2 | 91 | CCNB1 | 92 | CDKN1A |
| 93 | CEBPA | 94 | CHRNA1 | 95 | CKM | 96 | COBL |
| 97 | COL19A1 | 98 | COL2A1 | 99 | CREB1 | 100 | CYCS |
| 101 | DDIT3 | 102 | DIABLO | 103 | DMD | 104 | DMP1 |
| 105 | DPP4 | 106 | EIF2AK3 | 107 | EIF3B | 108 | ERN1 |
| 109 | FABP1 | 110 | FAS | 111 | FASLG | 112 | FGF9/16/20 |
| 113 | FIS1 | 114 | FOS | 115 | FOXO1 | 116 | FOXO3 |
| 117 | FXYD2 | 118 | GAST | 119 | GCLM | 120 | GJA5 |
| 121 | GRIA2 | 122 | GRIN2B | 123 | GSK3B | 124 | GSTA1 |
| 125 | GSTM1 | 126 | HMOX1 | 127 | HSP-1 | 128 | HSPA5 |
| 129 | IAPP | 130 | IBSP | 131 | ICAM1 | 132 | IDH2 |
| 133 | IL1B | 134 | IL4 | 135 | INS | 136 | INS1 |
| 137 | INSR | 138 | KY | 139 | LEP | 140 | LMOD2 |
| 141 | MAP4 | 142 | MAPK1 | 143 | MAPK14 | 144 | MAPK3 |
| 145 | MAPK8 | 146 | MCU | 147 | MDM2 | 148 | MEF2C |
| 149 | MFF | 150 | MFN1 | 151 | MPO | 152 | MT1 |
| 153 | MYBPH | 154 | MYD88 | 155 | MYF5 | 156 | MYF6 |
| 157 | MYH1 | 158 | MYH3 | 159 | MYH4 | 160 | MYH8 |
| 161 | MYL1 | 162 | MYLPF | 163 | MYOD1 | 164 | MYOG |
| 165 | MYOM2 | 166 | NEB | 167 | NFATC1 | 168 | NFE2L2 |
| 169 | NFKB1 | 170 | NFKBIA | 171 | NOS2 | 172 | NOS3 |
| 173 | NPPA | 174 | NPY | 175 | NQO1 | 176 | PARP1 |
| 177 | PINK1 | 178 | POPDC1 | 179 | PPARG | 180 | PRKAA1 |
| 181 | PRKG1 | 182 | PTPN1 | 183 | RELA | 184 | RYR1 |
| 185 | SAO | 186 | SAT1 | 187 | SCN8A | 188 | SHBG |
| 189 | SM30A | 190 | SMOX | 191 | SMYD1 | 192 | SOD1 |
| 193 | SPP1 | 194 | SPP19L | 195 | STAT3 | 196 | TH |
| 197 | TIA1 | 198 | TLR4 | 199 | TLR9 | 200 | TNF |
| 201 | TNNC2 | 202 | TNNI1 | 203 | TNNI2 | 204 | TNNT1 |
| 205 | TNNT3 | 206 | TP53 | 207 | TPM2 | 208 | TRDN |
| 209 | TTN | 210 | UGT1A6 | 211 | UNC5C | 212 | VGLL2 |
| 213 | XBP1 | 214 | XIAP | 215 | XIRP1 |  |  |

**Table S3.** Key targets (n = 138) identified through GO and KEGG enrichment analysis.

| **No.** | **Target** | **No.** | **Target** | **No.** | **Target** | **No.** | **Target** |
| --- | --- | --- | --- | --- | --- | --- | --- |
| 1 | CA2 | 2 | CA7 | 3 | CA1 | 4 | CA4 |
| 5 | CA5A | 6 | CHRNA3 | 7 | ODC1 | 8 | ALPL |
| 9 | SLC5A7 | 10 | DNM1 | 11 | ADH1A | 12 | FYN |
| 13 | EGFR | 14 | ACHE | 15 | CHRM3 | 16 | CYP2D6 |
| 17 | HTR6 | 18 | NOS1 | 19 | HRH2 | 20 | DRD2 |
| 21 | HRH1 | 22 | ADRA1B | 23 | DRD3 | 24 | OPRK1 |
| 25 | MAOA | 26 | MAOB | 27 | ADH1C | 28 | PLA2G2A |
| 29 | PLA2G1B | 30 | SLC6A4 | 31 | PRMT6 | 32 | HRH3 |
| 33 | SLC6A3 | 34 | ACTN3 | 35 | ADIPOQ | 36 | ADRB2 |
| 37 | AKT1 | 38 | AKT2 | 39 | ANXA5 | 40 | AOC1 |
| 41 | APP | 42 | ARG1 | 43 | ASGR1 | 44 | ASIC1 |
| 45 | ATF6 | 46 | ATP2A1 | 47 | BAX | 48 | BCL2 |
| 49 | CASP3 | 50 | CASP8 | 51 | CASP9 | 52 | CASR |
| 53 | CCL2 | 54 | CCNA2 | 55 | CCNB1 | 56 | CDKN1A |
| 57 | CEBPA | 58 | CKM | 59 | COL2A1 | 60 | CREB1 |
| 61 | CYCS | 62 | DDIT3 | 63 | DIABLO | 64 | DMD |
| 65 | DPP4 | 66 | EIF2AK3 | 67 | ERN1 | 68 | FABP1 |
| 69 | FAS | 70 | FASLG | 71 | FIS1 | 72 | FOS |
| 73 | FOXO1 | 74 | FOXO3 | 75 | FXYD2 | 76 | GAST |
| 77 | GCLM | 78 | GJA5 | 79 | GRIN2B | 80 | GSK3B |
| 81 | GSTA1 | 82 | GSTM1 | 83 | HMOX1 | 84 | HSPA5 |
| 85 | IBSP | 86 | ICAM1 | 87 | IDH2 | 88 | IL1B |
| 89 | IL4 | 90 | INS | 91 | INSR | 92 | LEP |
| 93 | MAPK1 | 94 | MAPK14 | 95 | MAPK3 | 96 | MAPK8 |
| 97 | MCU | 98 | MDM2 | 99 | MEF2C | 100 | MFF |
| 101 | MPO | 102 | MYD88 | 103 | MYH1 | 104 | MYH3 |
| 105 | MYOD1 | 106 | NFATC1 | 107 | NFE2L2 | 108 | NFKB1 |
| 109 | NFKBIA | 110 | NOS2 | 111 | NOS3 | 112 | NPPA |
| 113 | NPY | 114 | NQO1 | 115 | PARP1 | 116 | PINK1 |
| 117 | POPDC1 | 118 | PPARG | 119 | PRKAA1 | 120 | PRKG1 |
| 121 | PTPN1 | 122 | RELA | 123 | RYR1 | 124 | SHBG |
| 125 | SOD1 | 126 | SPP1 | 127 | STAT3 | 128 | TH |
| 129 | TLR4 | 130 | TLR9 | 131 | TNF | 132 | TNNC2 |
| 133 | TNNT3 | 134 | TP53 | 135 | TPM2 | 136 | UGT1A6 |
| 137 | XBP1 | 138 | XIAP |  |  |  |  |

**Table S4.** Primers and oligos used in this study.

| **Primers for quantitative RT-PCR** | |
| --- | --- |
| COL1A1-Human | Forward: GACGGCTCAGAGTCACCCA Reverse: GGAGACCACGAGGACCAGA |
| COL1A1-Mouse | Forward: ACCCTGCCCGCACATG  Reverse: CCCTCGCTTCCGTACTCG |
| α-SMA-Human | Forward: ATCGTCCACCGCAAATGC Reverse: AAGGAACTGGAGGCGCTG |
| α-SMA-Mouse | Forward: AATGGCTCTGGGCCTGTAA Reverse: TCTCTTGCTCTGGGCTTCAT |
| TNF-α-Human | Forward: GCCCATGTTGTAGCAAACCC Reverse: TCTGGTAGGAGACGGCGATG |
| TNF-α-Mouse | Forward: CCTGCCCCAAGGACACC Reverse: AGAGCAATGACTCCAAAGTAGA  CC |
| BNG-Human | Forward: CAGTGGCTTTGAACCTGGAG Reverse: GGGAGGTCTTTGGGGATGC |
| BNG-Mouse | Forward: CCTCCAGCACCTCTATGCTC Reverse: ACTTTGAGGATACGGTTGTC |
| TIMP-1-Human | Forward: GCTTCTGGCATCCTGTTGTT  Reverse: TGGTTGACTTCTGGTGTCCC |
| TIMP-1-Mouse | Forward: CCCAGAAATCAACGAGACCA  Reverse: ACGCCAGGGAACCAAGAA |
| UBE2G2-Human | Forward: ATCTACCCTGATGGGAGAGTCT Reverse: CTCCACTTTCGTCATTGGGC |
| UBE2G2-Mouse | Forward: TGGCCGAGTATAAGCAATTAACC Reverse: GGCTCAAGGGGTAGTCAAGT |
| STAB1-Human | Forward: CAAGCAATGAGGCTGTGGAC Reverse: GCCGTGGTTGTAGATGTGGT |
| STAB1-Mouse | Forward: CATTGTGCAACGGCACTTGA Reverse: CCCACAGATGCTGCAAGTCT |
| STAB2-Human | Forward: ACTGGCTCCTTACCAAACCTGC Reverse: GAGCAAACACTGTGTAGGCATCG |
| STAB2-Mouse | Forward: GGCTGGGGCAGGAACTAC Reverse: CGCTTGGGCTCTCGGAC |
| CD209B-Human | Forward: GCAGTCTTCCAGAAGTAACCGC Reverse: GCTCTCCTCTGTTCCAATACTGC |
| CD209B-Mouse | Forward: GGCTAAAGGACCAACCTGGATG  Reverse: CTCACCGATGTTGTTAGGCTCC |
| IL-1-Human | Forward: ATTGAGCCTCATGCTCTGTTCTT Reverse: GAAGGCGAAGCGCTTGTC |
| IL-1-Mouse | Forward: AAGCCTCGTGCTGTCGGA Reverse: CCATCTTCTTCTTTGGGTATTGC |
| EHD3-Human | Forward: TCAGGAAACTCAACGCCTTTG Reverse: CTCCAGCAGGGTTAGGTAG |
| EHD3-Mouse | Forward: CGCCGTGCTTGAAAGTATCAG Reverse: ATAATTCGGTCCACCCGCTC |
| EDN1-Human | Forward: CAAGCAGGAAAAGAACTCAG Reverse: CTGGTTTGTCTTAGGTGTTC |
| EDN1-Mouse | Forward: AGAGTGTGTCTACTTCTGCCA Reverse: CTTCCAAGTCCATACGGAACAA |
| LAMB1-Human | Forward: AGGTTGGAGCTGCCTCAGTA Reverse: ACACTCCCTGGAAACAGTGG |
| LAMB1-Mouse | Forward: CAGTGCCAGTGCCGTCCTAATG Reverse: CCAGTGATCGCATCGCAGAAGG |
| PECAM1-Human | Forward:  AAGTGGAGTCCAGCCGCATATC Reverse: ATGGAGCAGGACAGGTTCAGTC |
| PECAM1-Mouse | Forward: TGGTTGTCATTGGAGTGGTC  Reverse: TTCTCGCTGTTGGAGTTCAG |
| β-actin-Human | Forward: AGGCCAACCGCGAGAAGATGACC Reverse: GAAGTCCAGGGCGACGTAGCAC |
| GAPDH-Human | Forward: CGACCACTTTGTCAAGCTCA Reverse: AGGGGTCTACATGGCAACTG |
| GAPDH-Mouse | Forward: CTCCCACTCTTCCACCTTCG Reverse: CCACCACCCTGTTGCTGTAG |
