# Supplementary material for: Spermidine suppresses liver fibrosis by remodeling the communication signal between liver sinusoidal endothelial cells and hepatic stellate cells
Source: Cell Death Discov. 2026 May 7;12:287. doi: 10.1038/s41420-026-03129-4 (PMC13320189; doi:10.1038/s41420-026-03129-4)
Supplement: Supplementary file 2 — Full and uncropped western blots [file 41420_2026_3129_MOESM2_ESM.pdf]

Figure2C

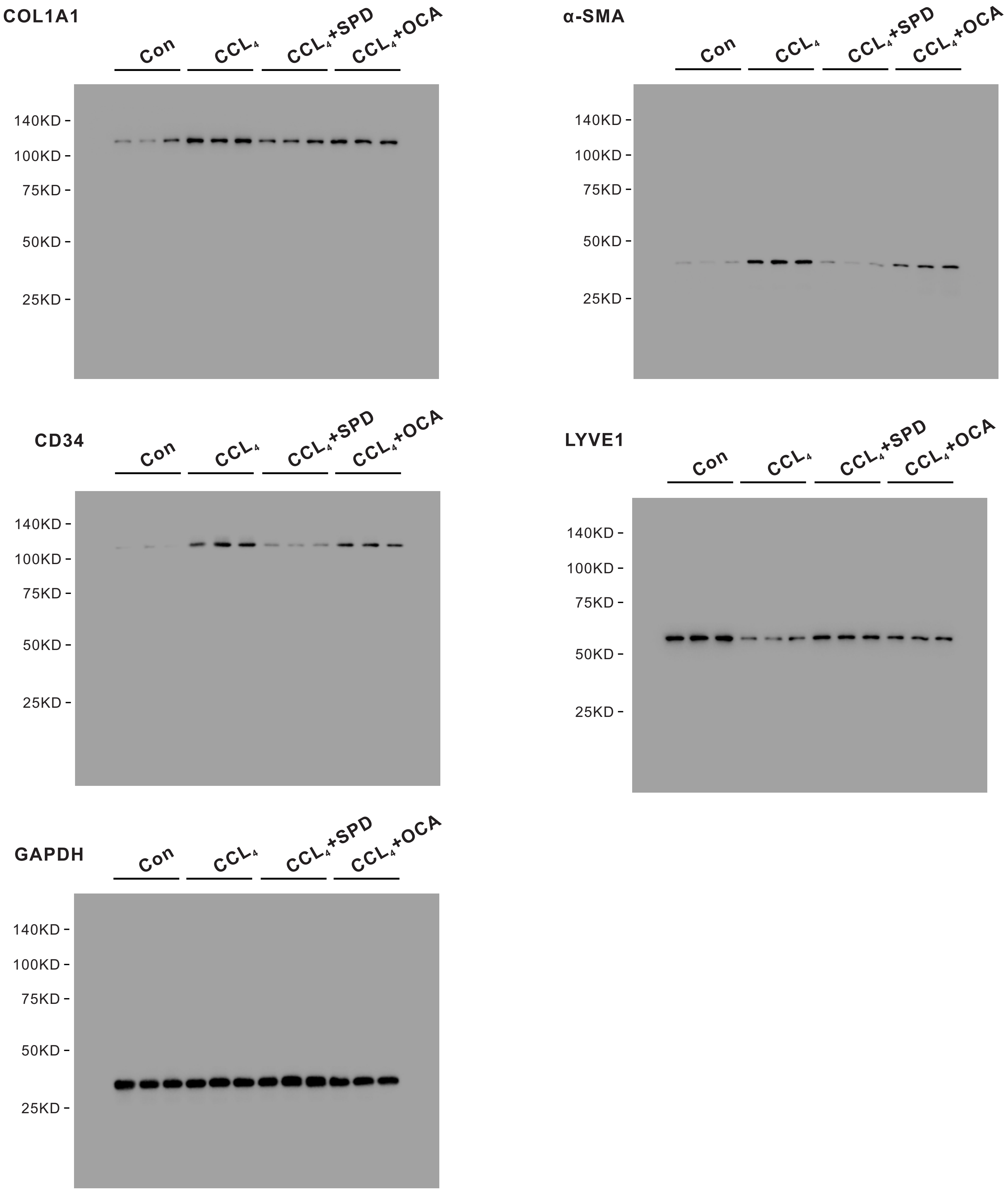
COL1A1
α-SMA
CD34
LYVE1
GAPDH
Con
CCL4
CCL4+SPD
CCL4+OCA
140KD
100KD
75KD
50KD
25KD

Figure2H

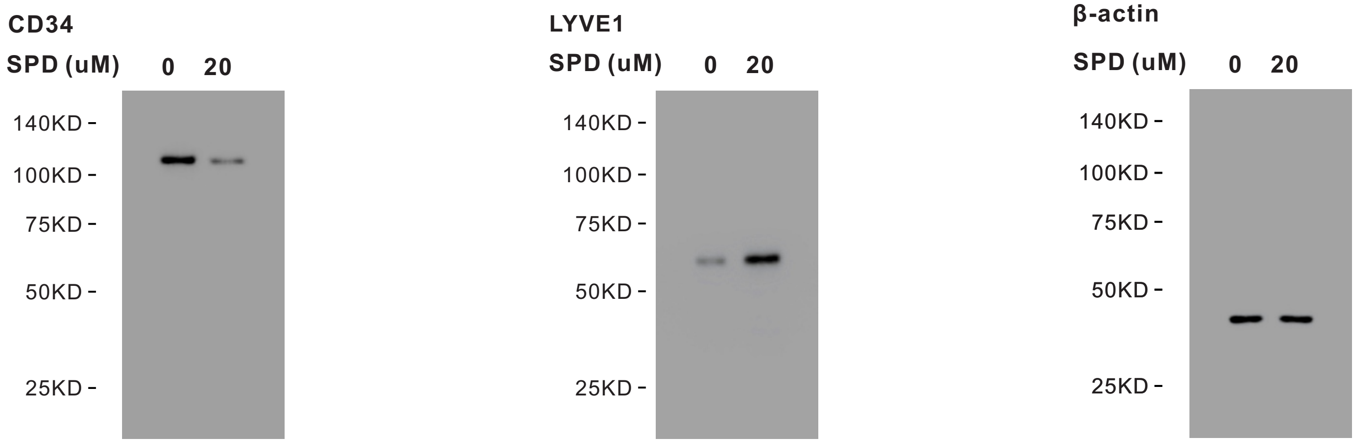

Figure2I

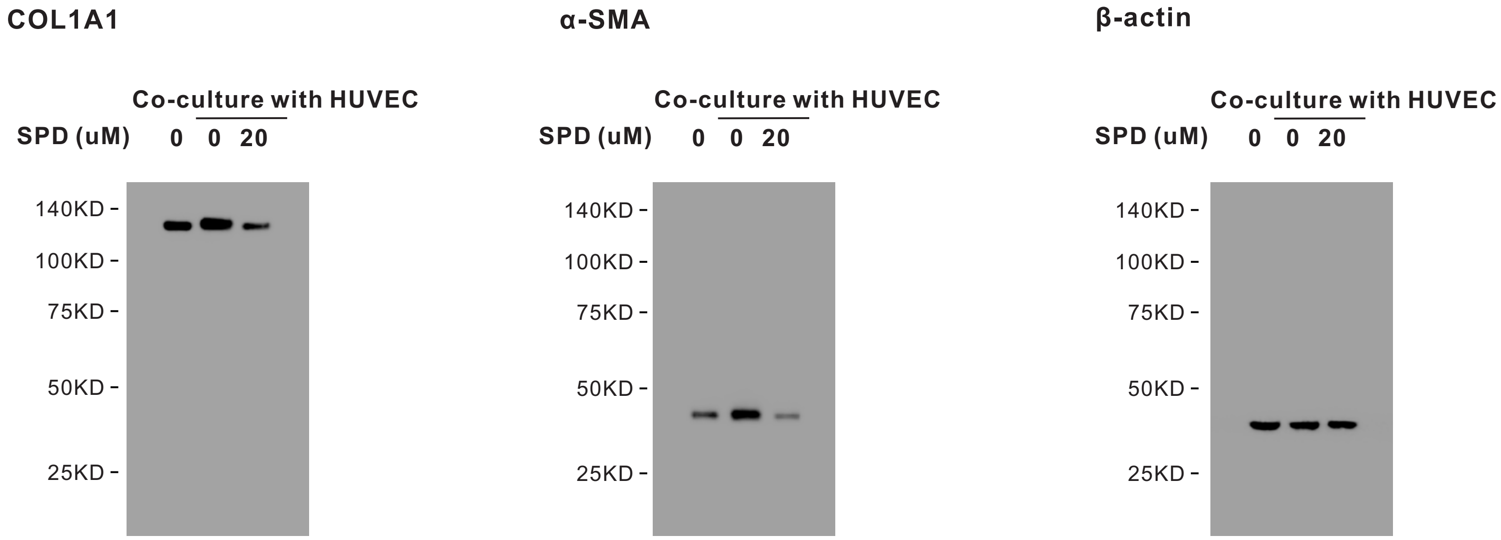

Figure3E

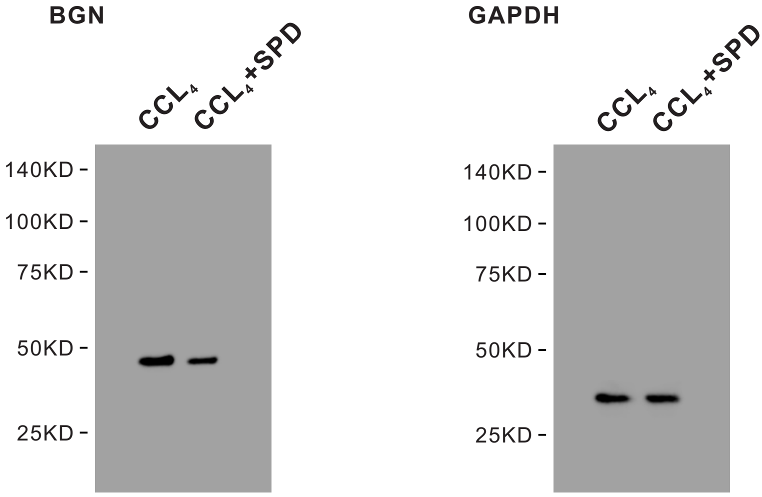

Figure3F

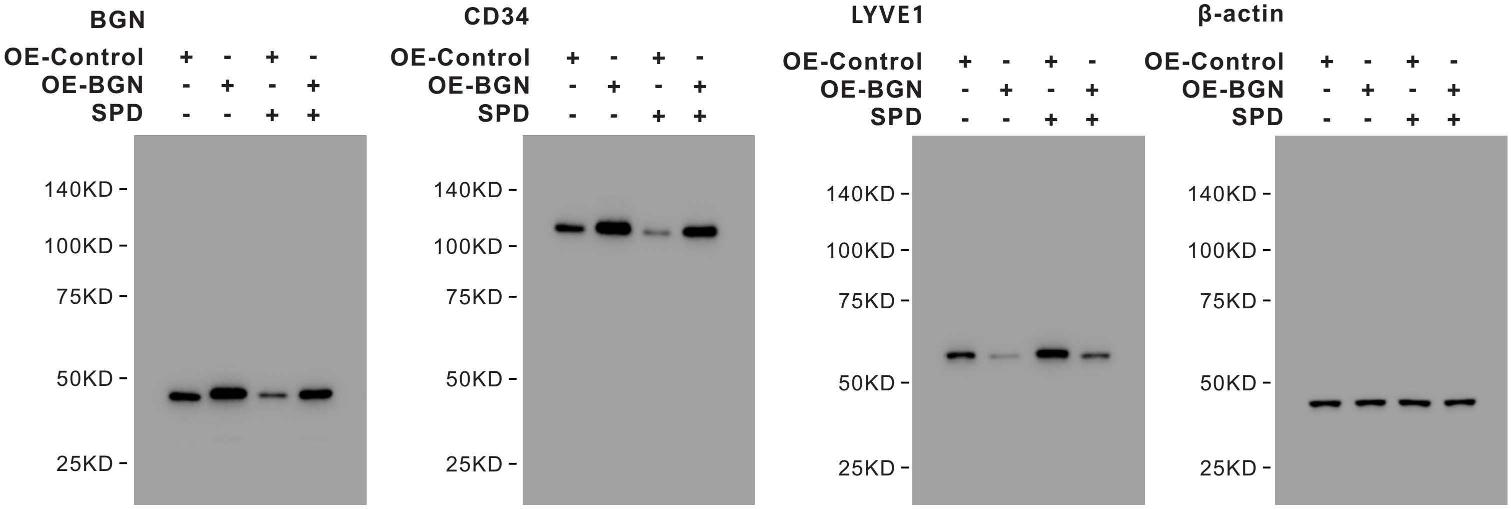

Figure3I

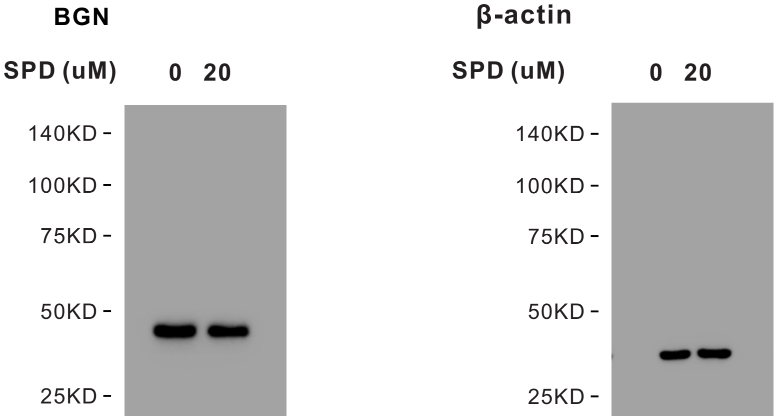

Figure3J

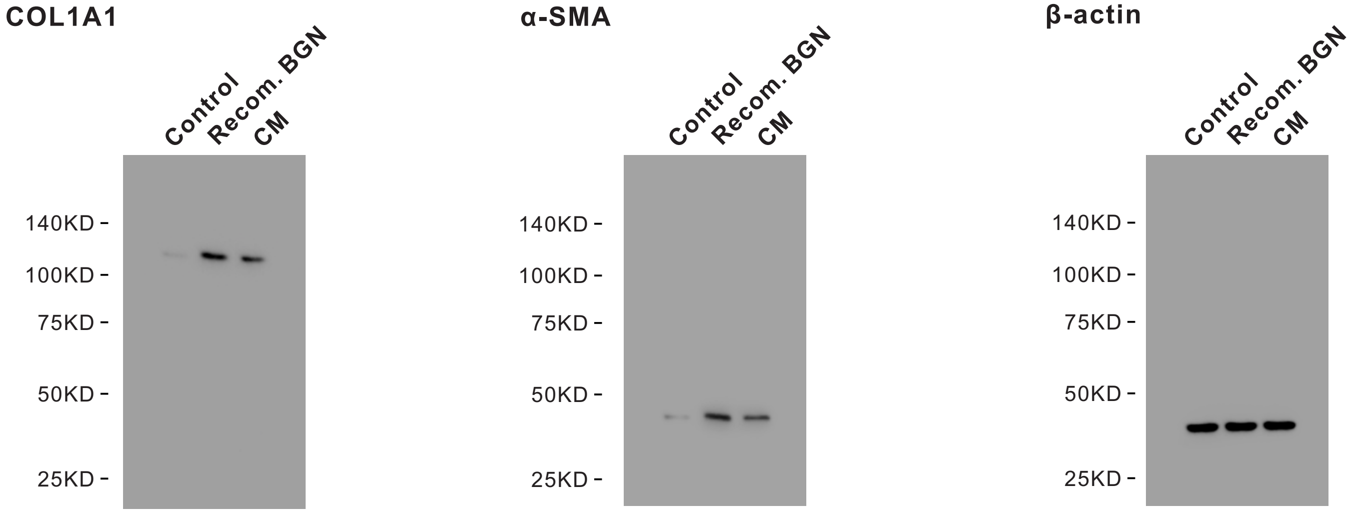

Figure3K

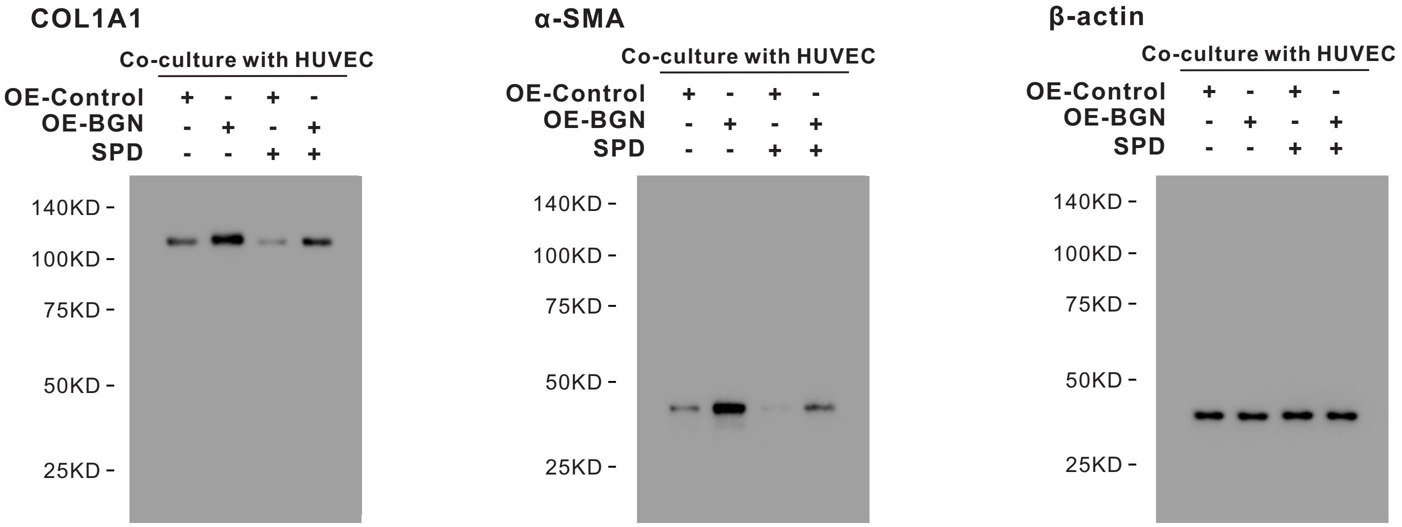

Figure4F left

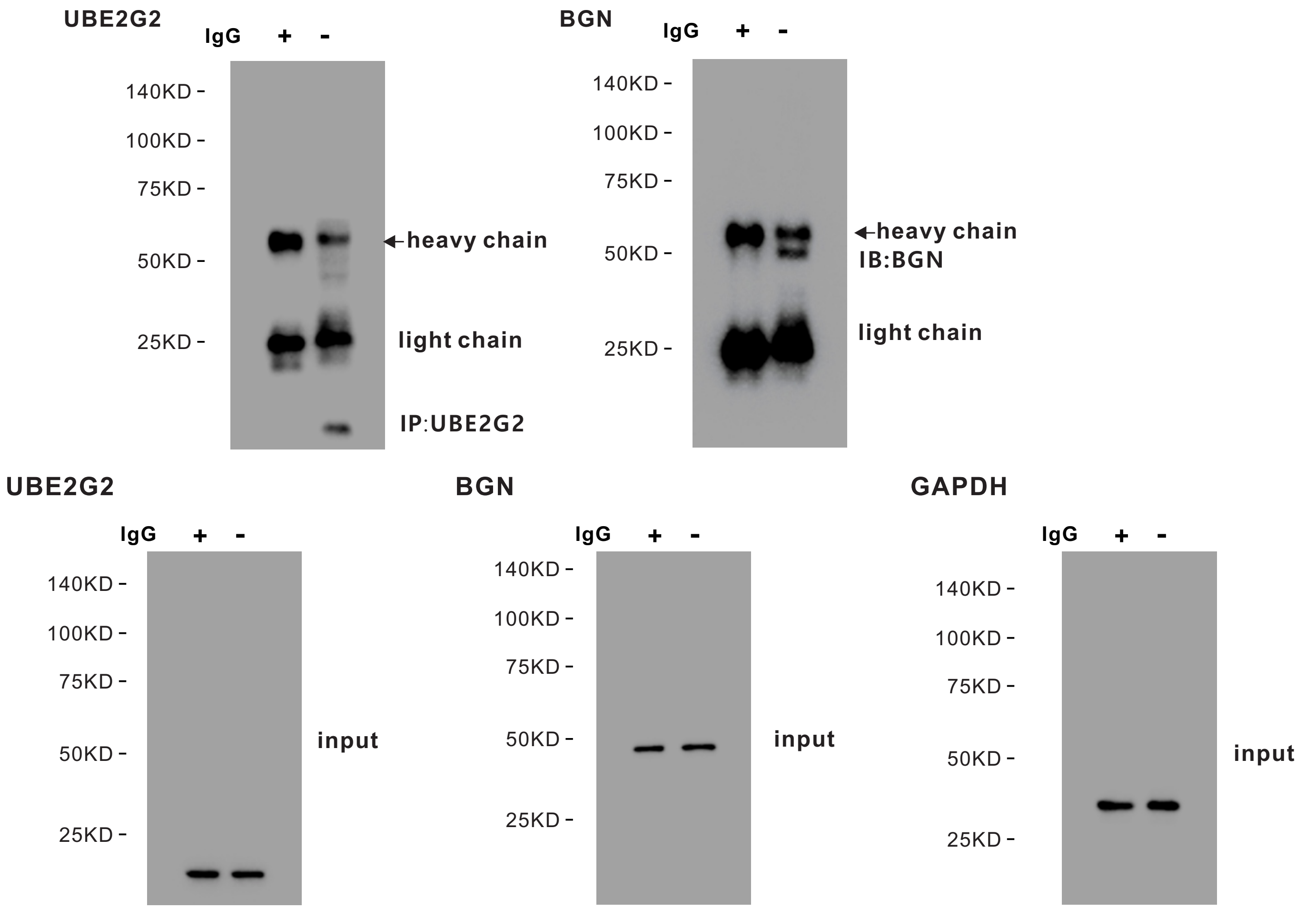

Figure4F right

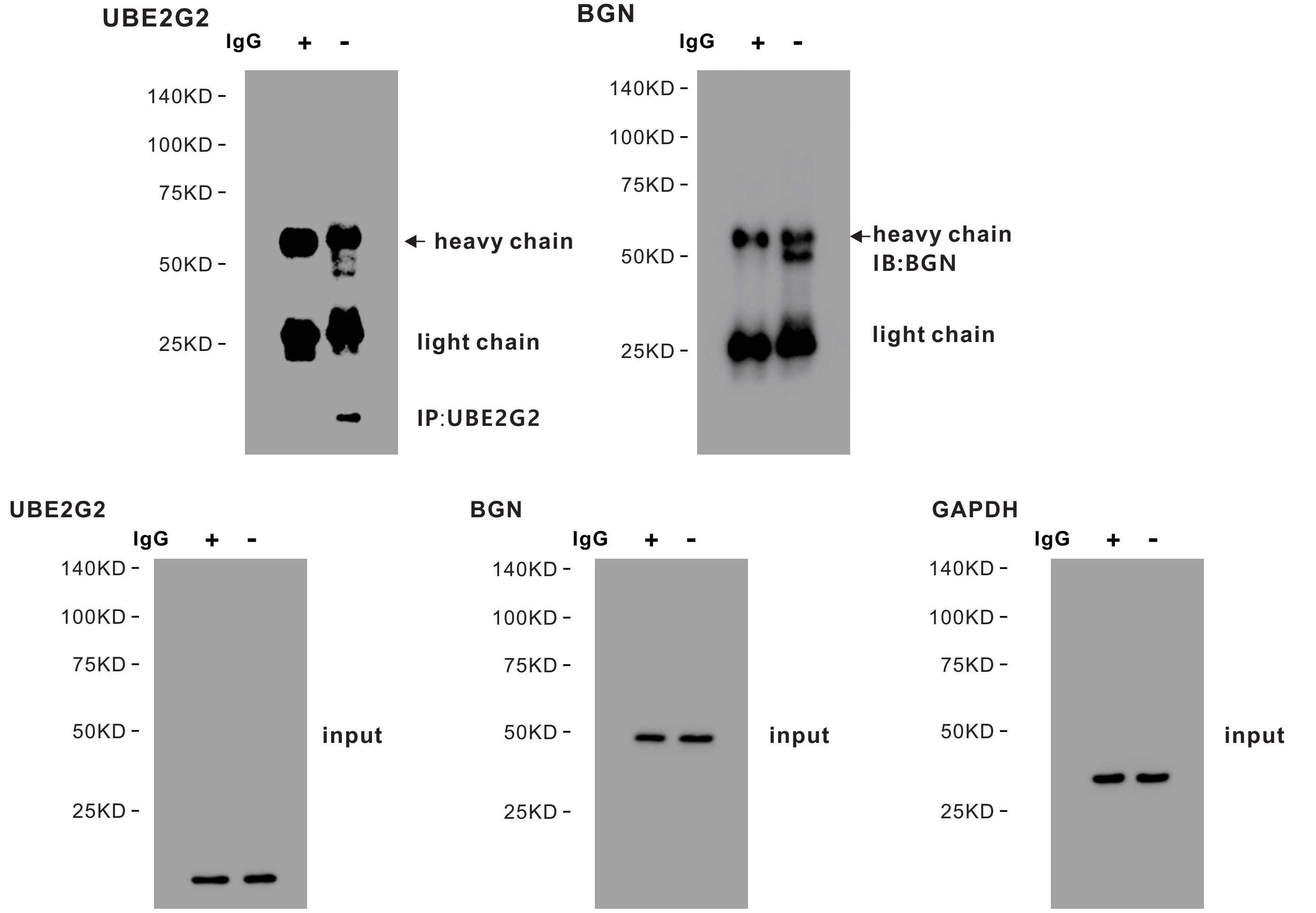

Figure4G

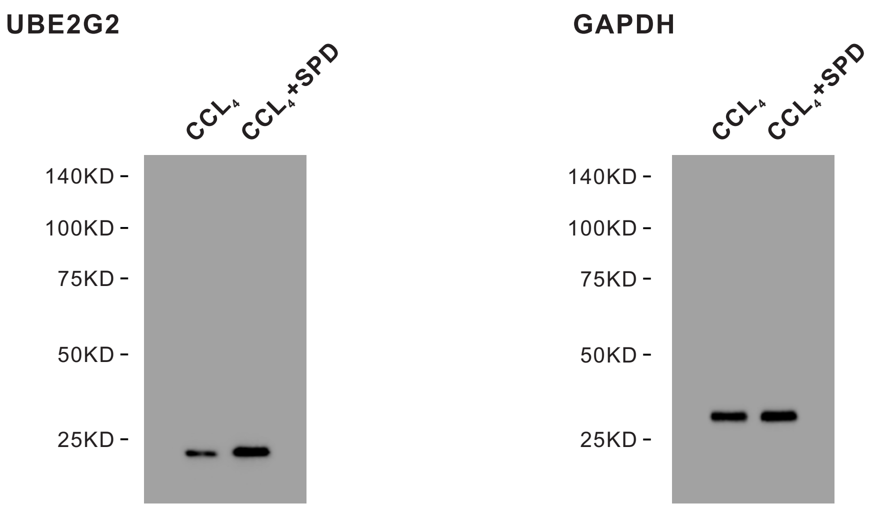

Figure4I

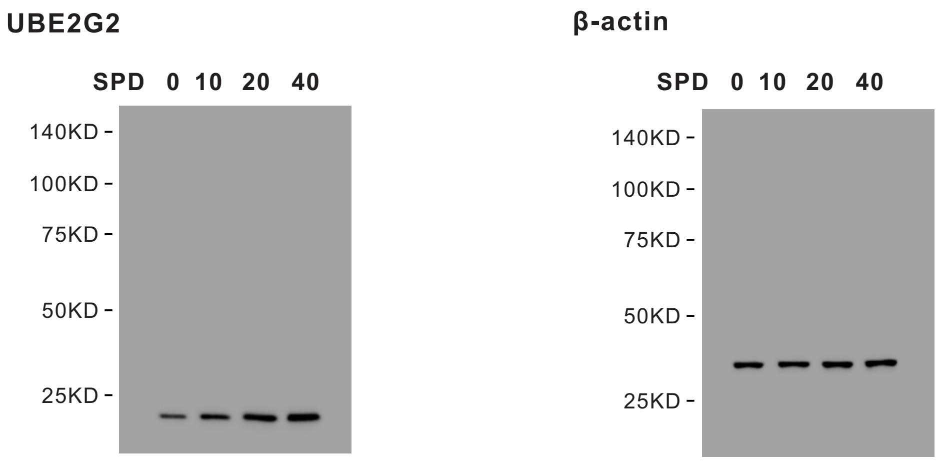

Figure4J liver tissuse

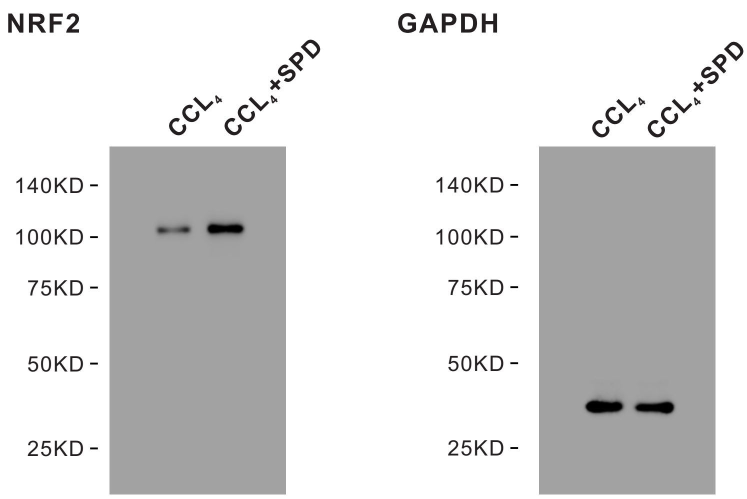

Figure4J HUVEC

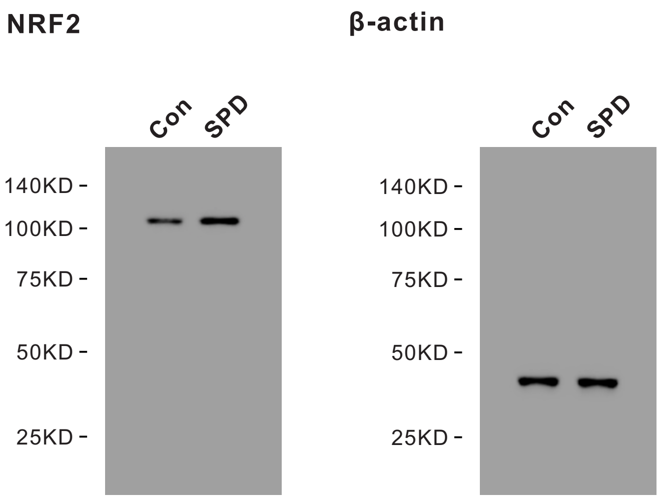

Figure4K

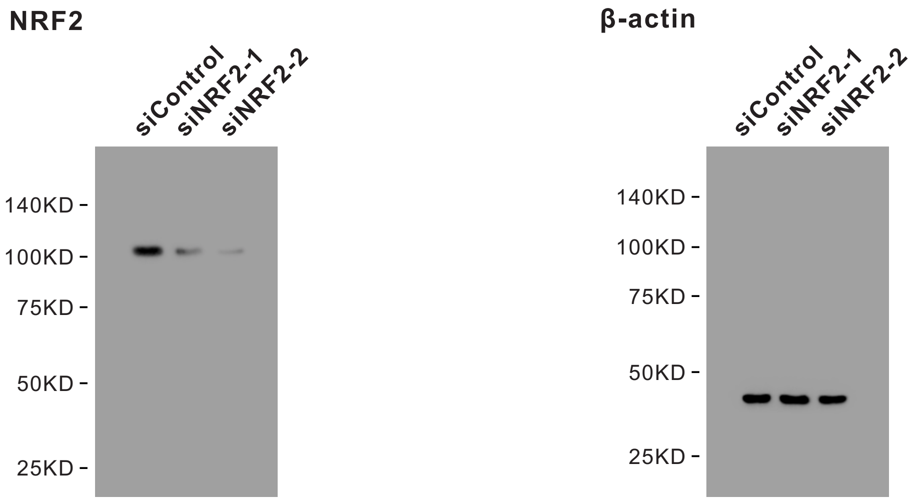

Figure4M

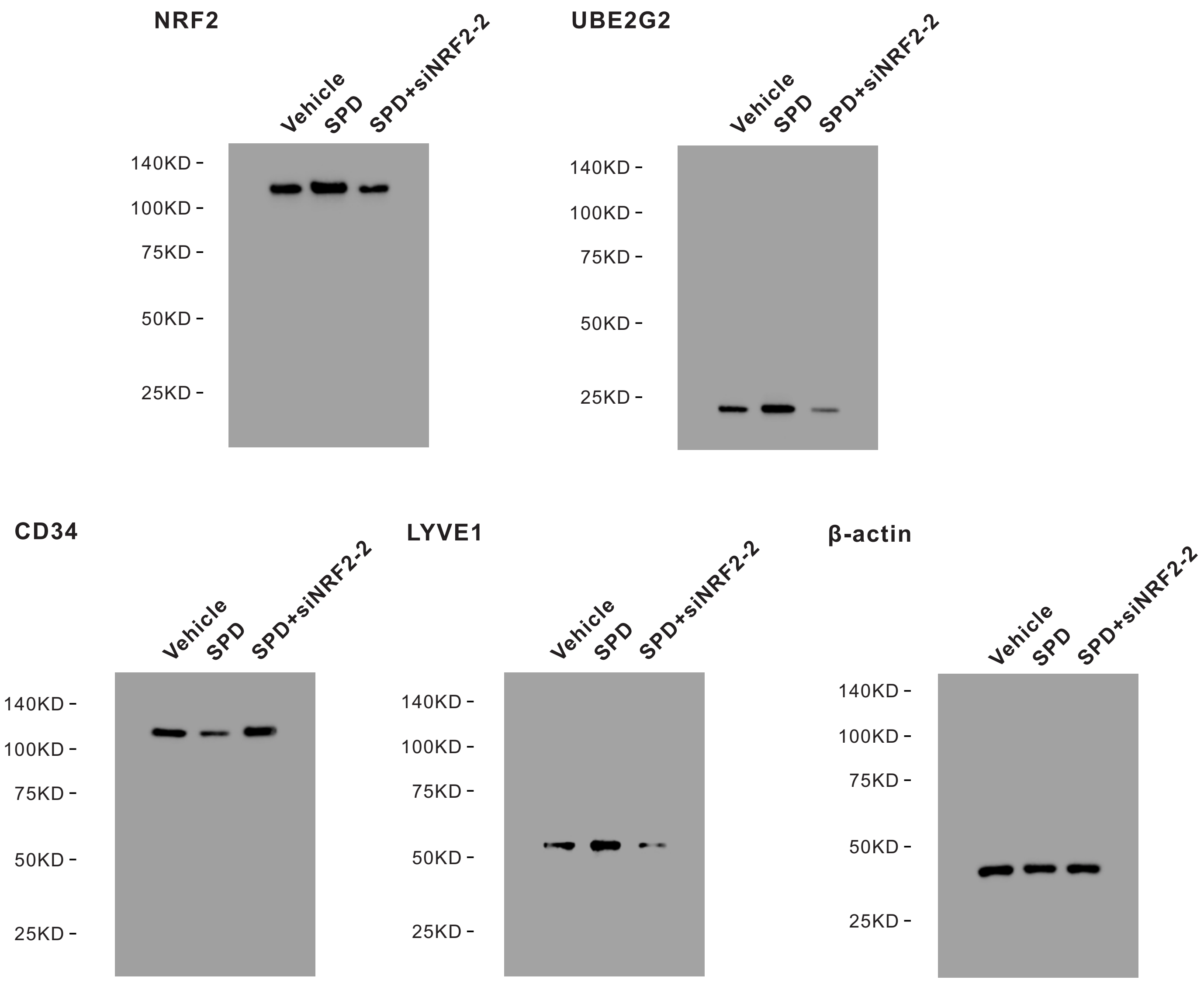

Figure5A Left

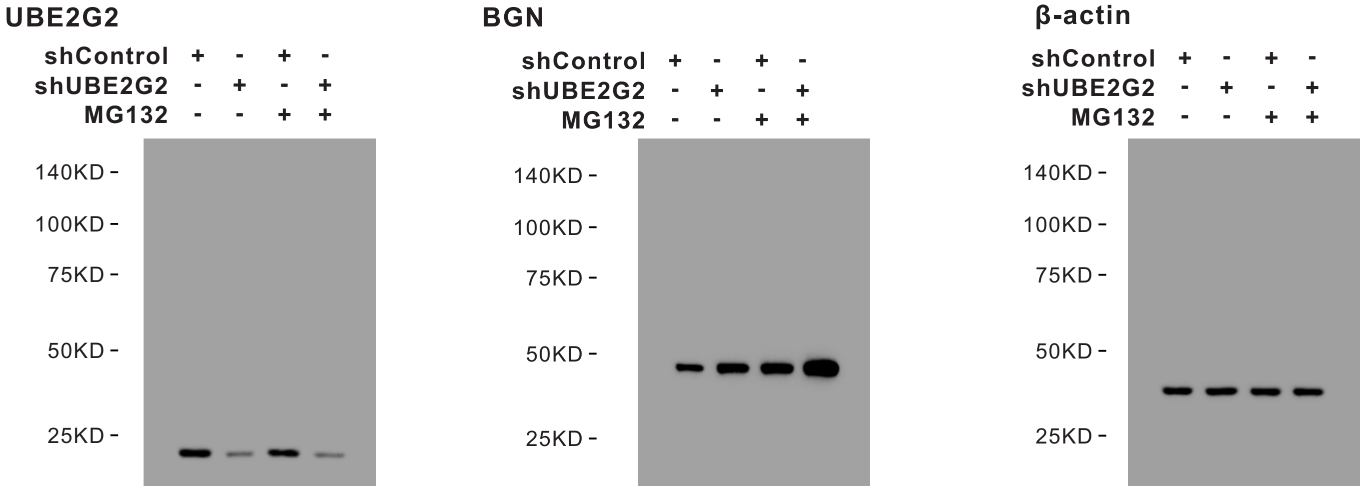

Figure5A Right

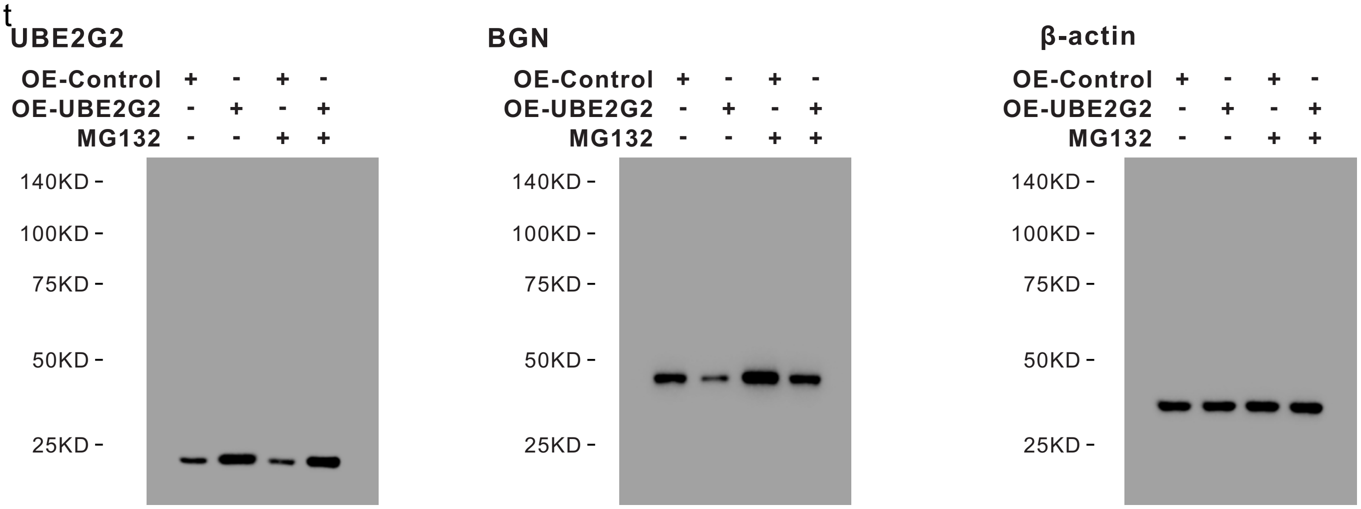

Figure5B Left

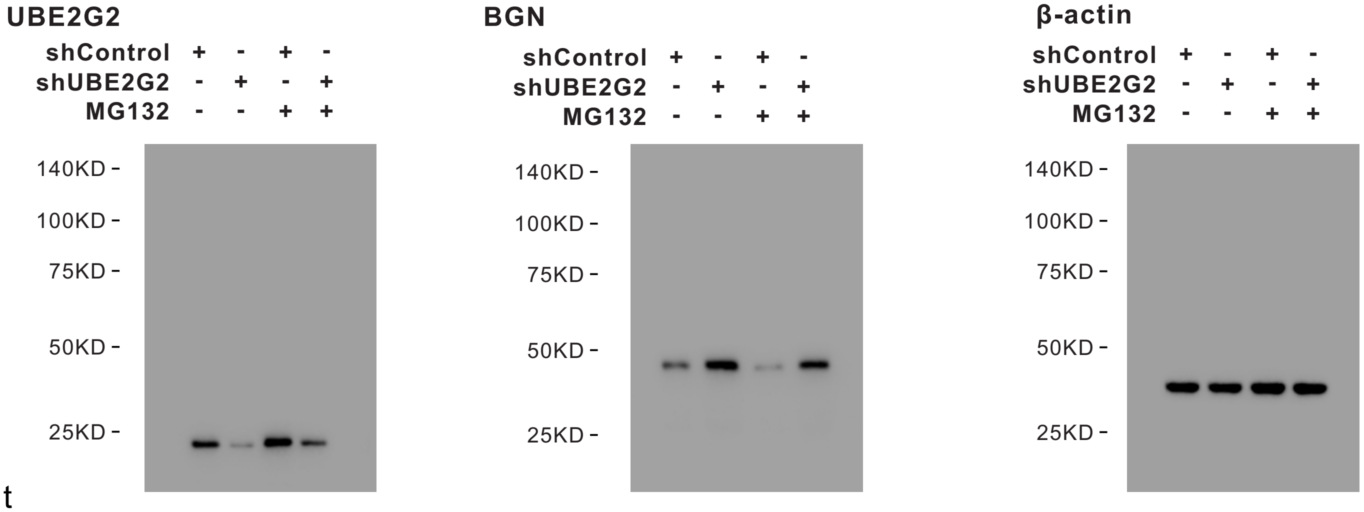

Figure5B Right

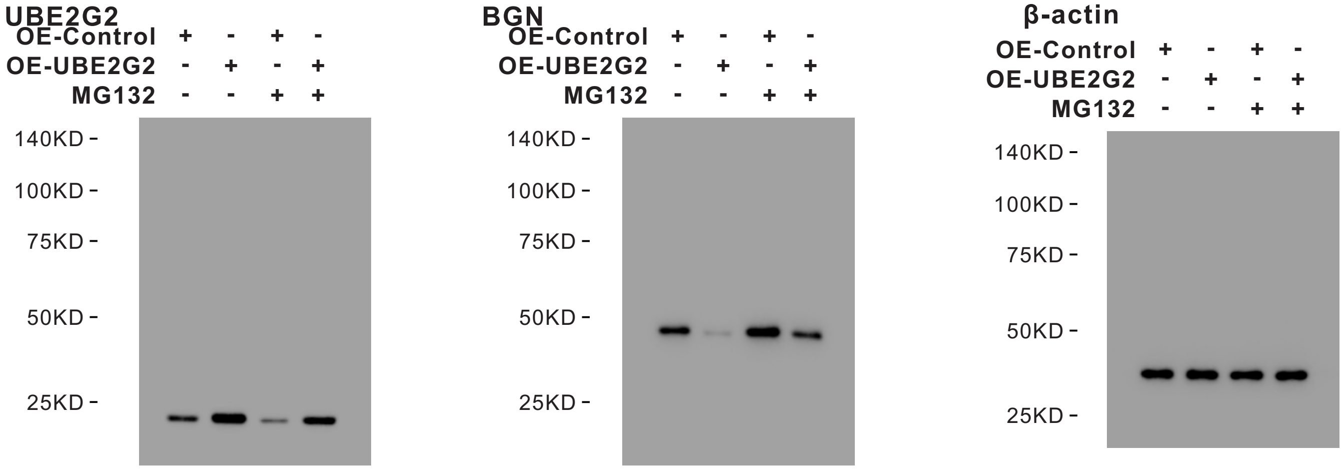

Figure5C

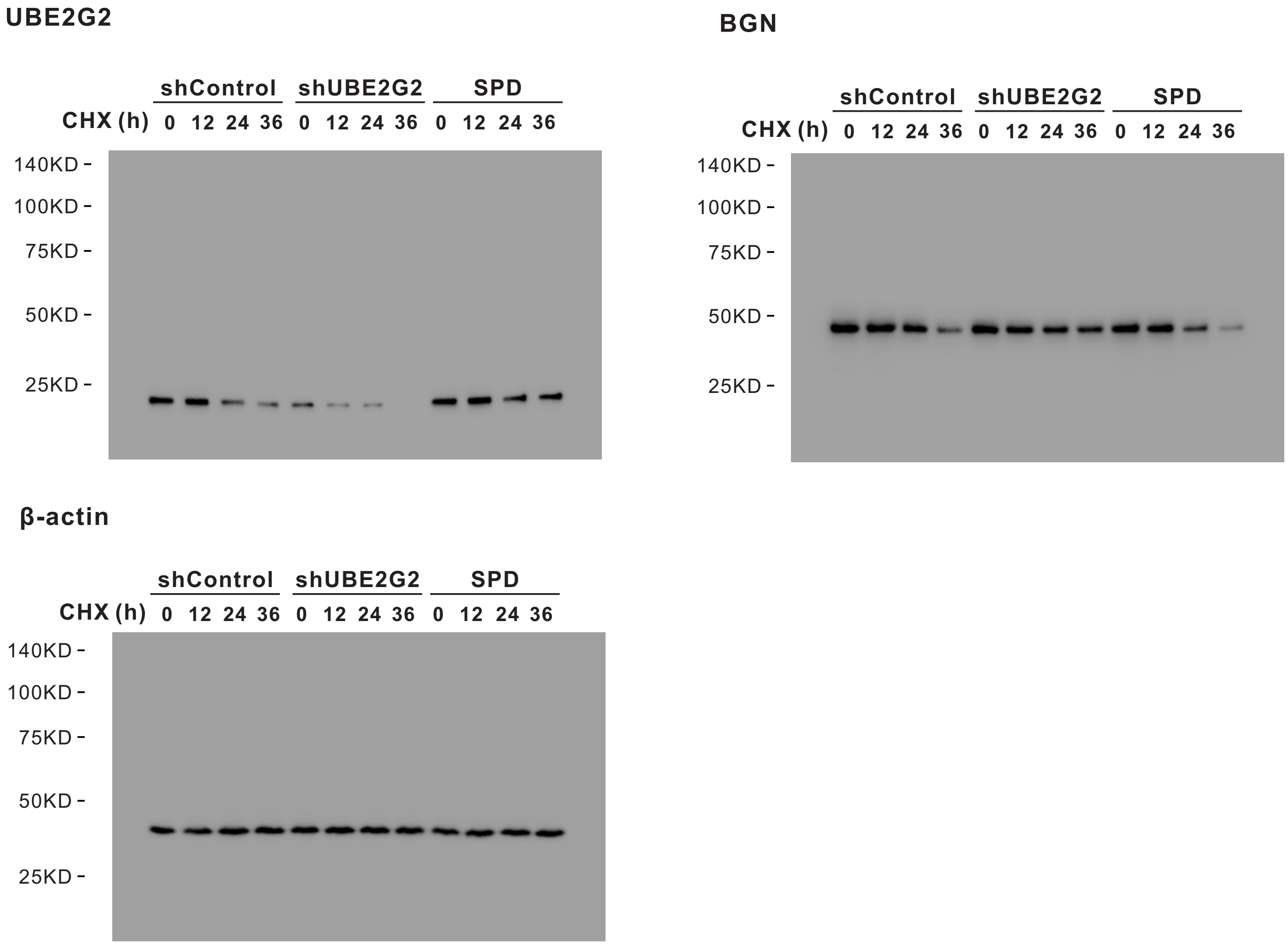

Figure5D

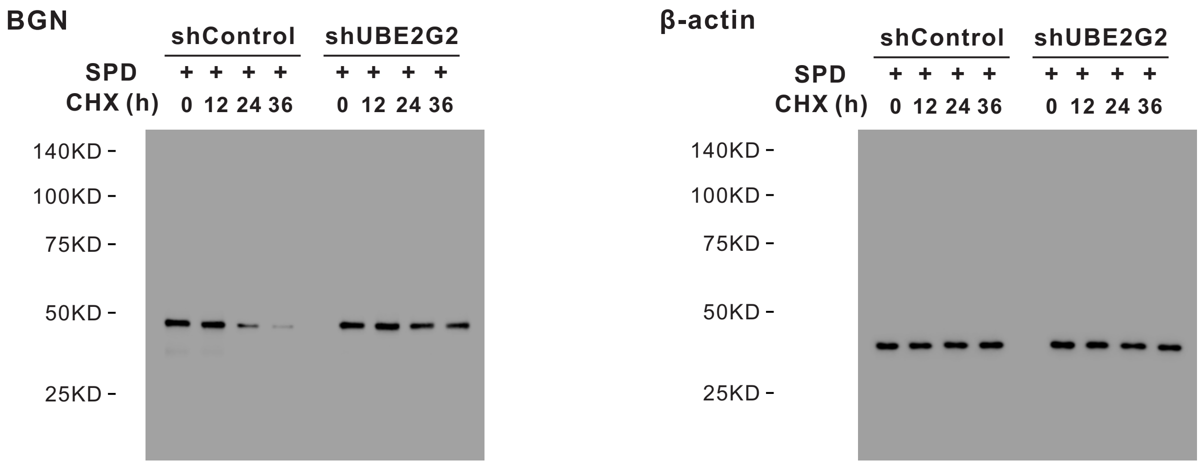

Figure5E

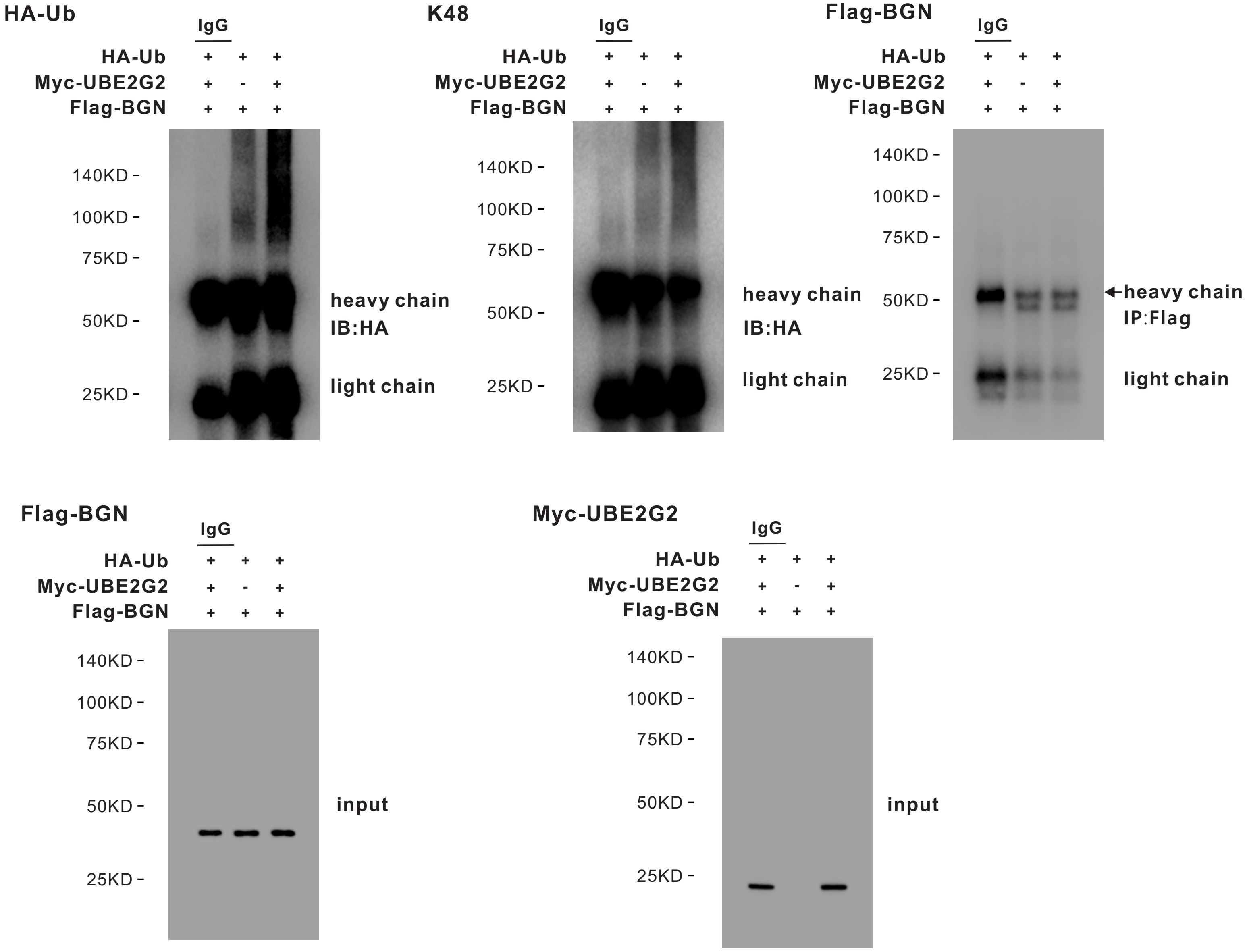

Figure5F

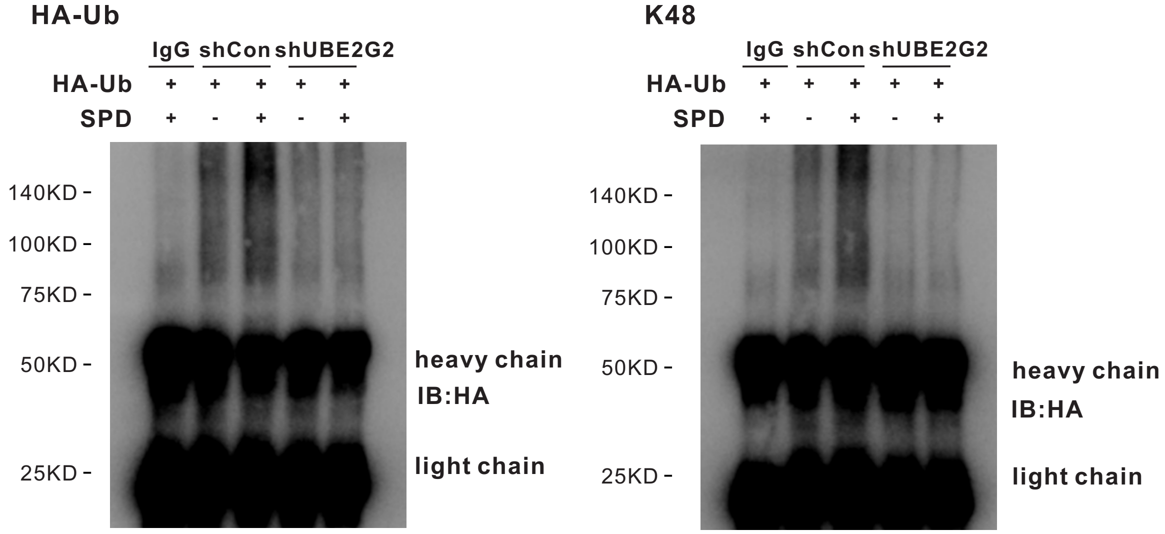

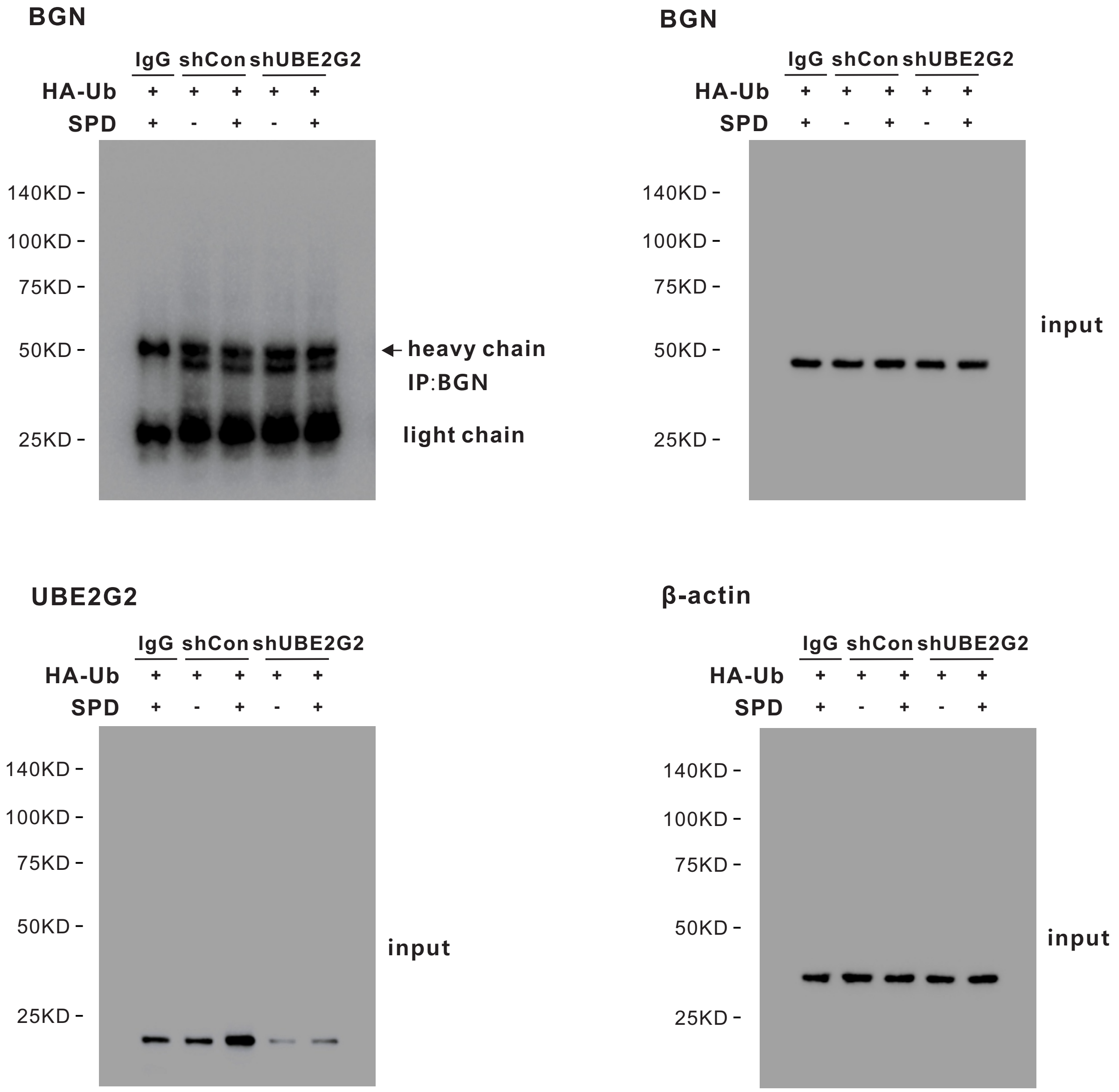

BGN
IgG shCon shUBE2G2
HA-Ub + + + + +
SPD + - + - +
140KD
100KD
75KD
50KD
25KD
heavy chain
IP:BGN
light chain
BGN
IgG shCon shUBE2G2
HA-Ub + + + + +
SPD + - + - +
140KD
100KD
75KD
50KD
25KD
input
UBE2G2
IgG shCon shUBE2G2
HA-Ub + + + + +
SPD + - + - +
140KD
100KD
75KD
50KD
25KD
input
β-actin
IgG shCon shUBE2G2
HA-Ub + + + + +
SPD + - + - +
140KD
100KD
75KD
50KD
25KD
input

Figure5G

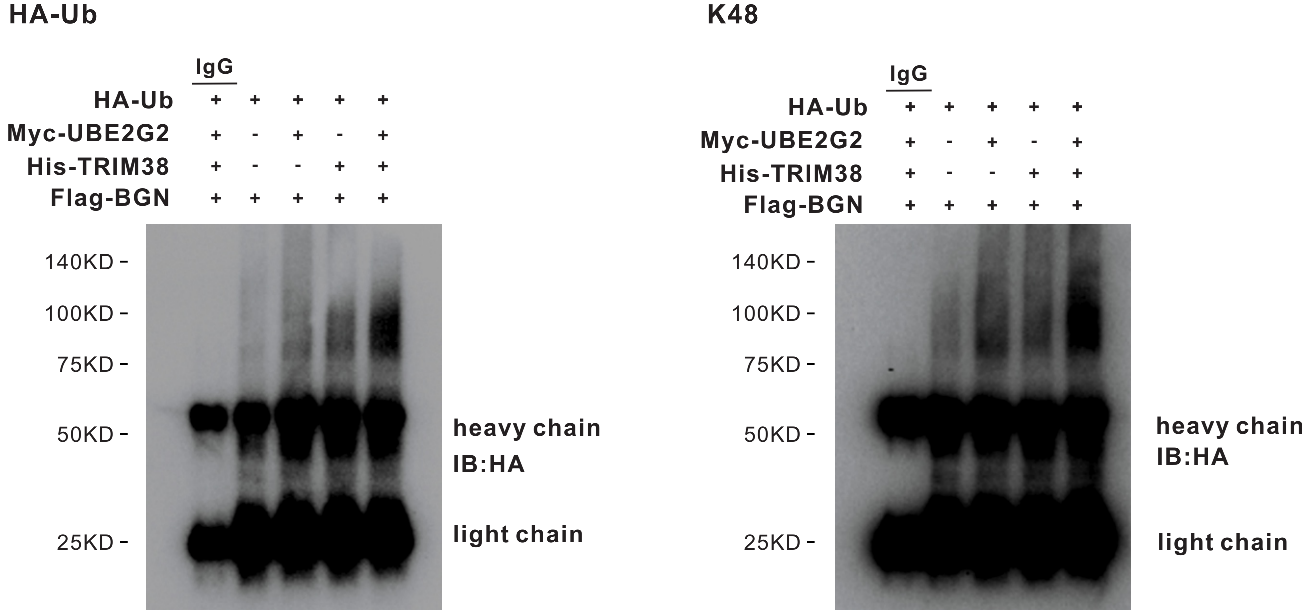

HA-Ub
IgG
HA-Ub + + + + +
Myc-UBE2G2 + - + - +
His-TRIM38 + - - + +
Flag-BGN + + + + +
140KD
100KD
75KD
50KD
25KD
heavy chain
IB:HA
light chain
K48
IgG
HA-Ub + + + + +
Myc-UBE2G2 + - + - +
His-TRIM38 + - - + +
Flag-BGN + + + + +
140KD
100KD
75KD
50KD
25KD
heavy chain
IB:HA
light chain

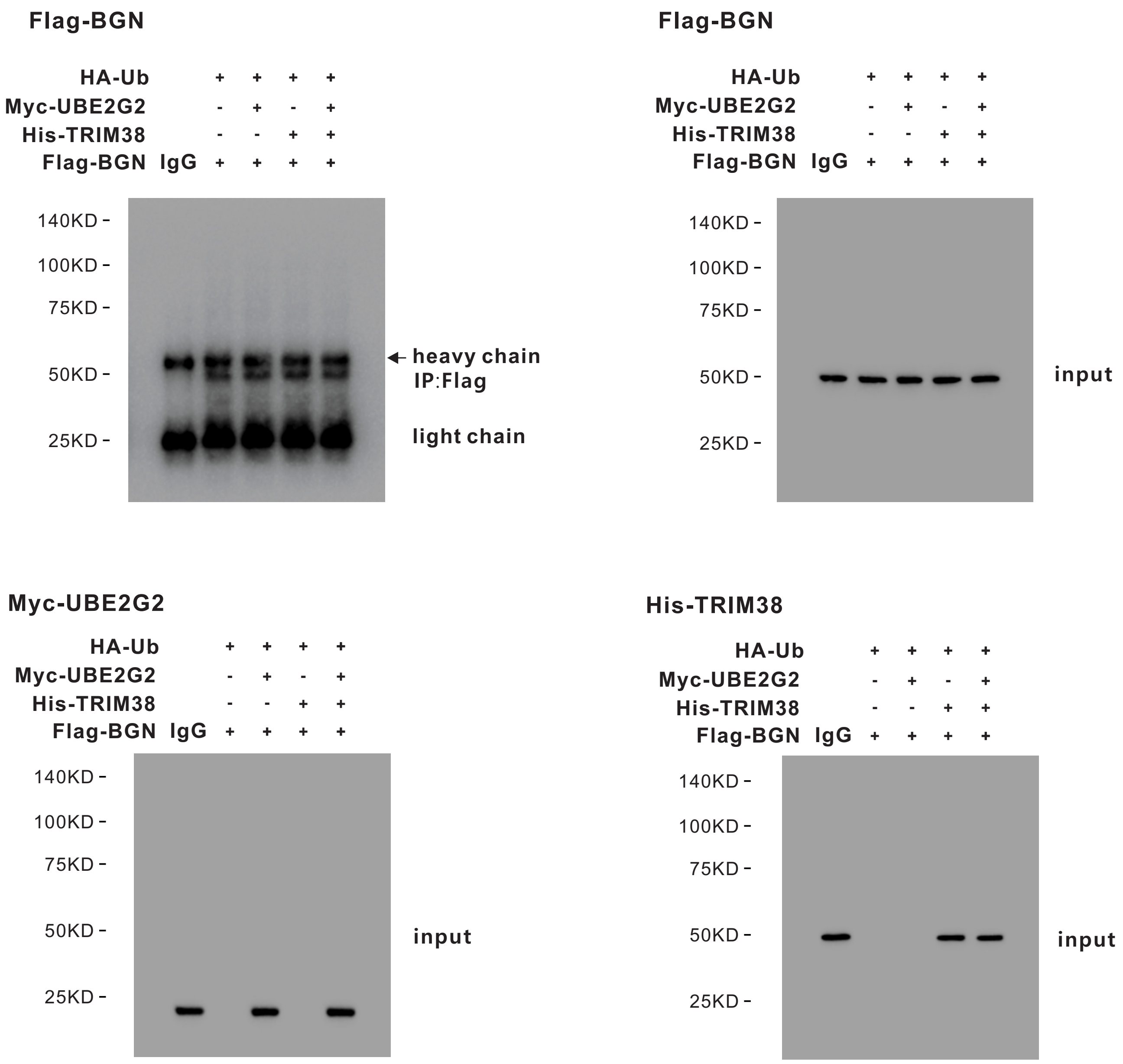
Flag-BGN
HA-Ub + + + +
Myc-UBE2G2 - + - +
His-TRIM38 - - + +
Flag-BGN IgG + + + +
140KD
100KD
75KD
50KD
25KD
heavy chain
IP:Flag
light chain
Flag-BGN
HA-Ub + + + +
Myc-UBE2G2 - + - +
His-TRIM38 - - + +
Flag-BGN IgG + + + +
140KD
100KD
75KD
50KD
25KD
input
Myc-UBE2G2
HA-Ub + + + +
Myc-UBE2G2 - + - +
His-TRIM38 - - + +
Flag-BGN IgG + + + +
140KD
100KD
75KD
50KD
25KD
input
His-TRIM38
HA-Ub + + + +
Myc-UBE2G2 - + - +
His-TRIM38 - - + +
Flag-BGN IgG + + + +
140KD
100KD
75KD
50KD
25KD
input

Figure6A

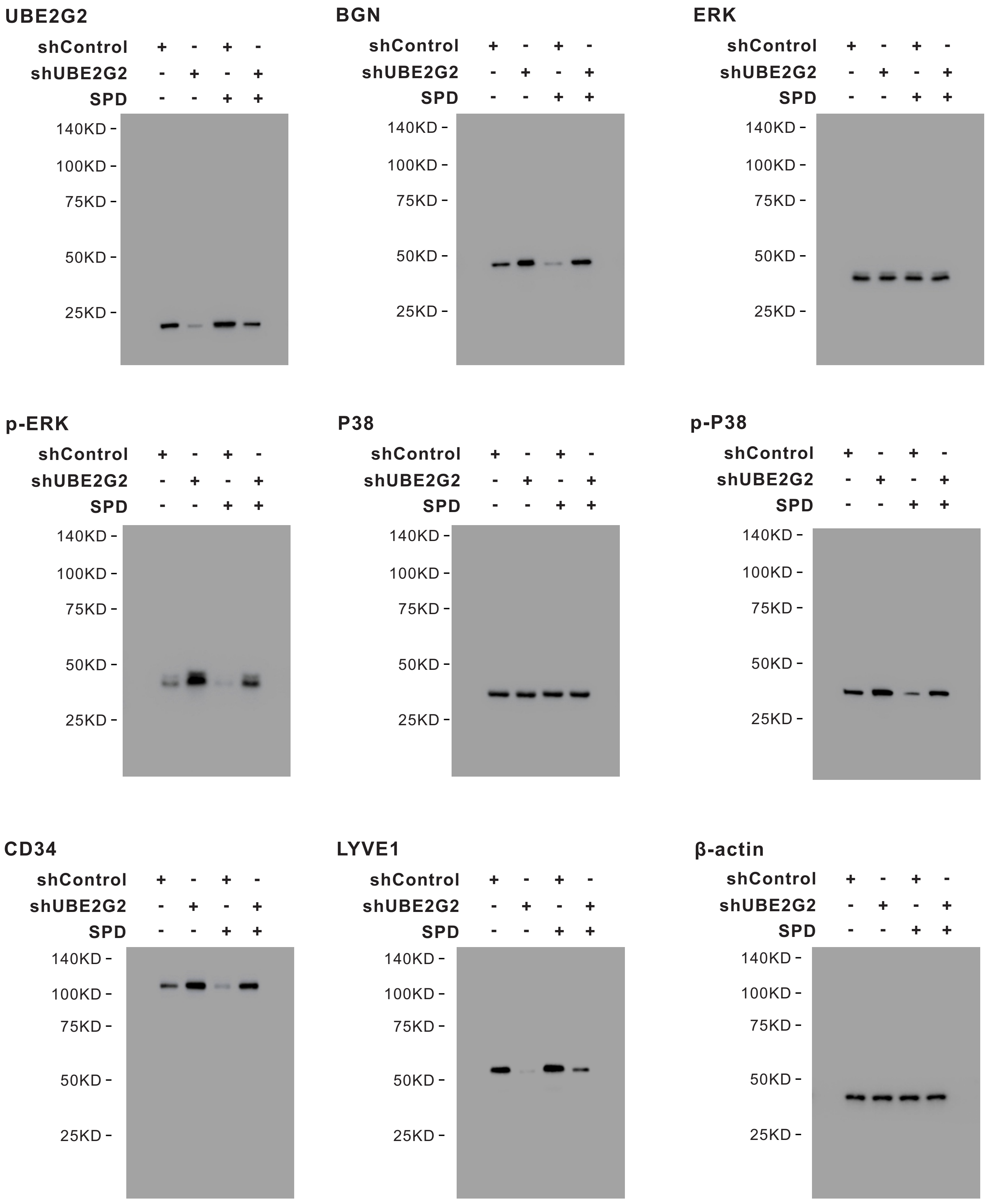
UBE2G2
shControl + - + -
shUBE2G2 - + - +
SPD - - + +
140KD
100KD
75KD
50KD
25KD
BGN
shControl + - + -
shUBE2G2 - + - +
SPD - - + +
140KD
100KD
75KD
50KD
25KD
ERK
shControl + - + -
shUBE2G2 - + - +
SPD - - + +
140KD
100KD
75KD
50KD
25KD
p-ERK
shControl + - + -
shUBE2G2 - + - +
SPD - - + +
140KD
100KD
75KD
50KD
25KD
P38
shControl + - + -
shUBE2G2 - + - +
SPD - - + +
140KD
100KD
75KD
50KD
25KD
p-P38
shControl + - + -
shUBE2G2 - + - +
SPD - - + +
140KD
100KD
75KD
50KD
25KD
CD34
shControl + - + -
shUBE2G2 - + - +
SPD - - + +
140KD
100KD
75KD
50KD
25KD
LYVE1
shControl + - + -
shUBE2G2 - + - +
SPD - - + +
140KD
100KD
75KD
50KD
25KD
β-actin
shControl + - + -
shUBE2G2 - + - +
SPD - - + +
140KD
100KD
75KD
50KD
25KD

Figure6E

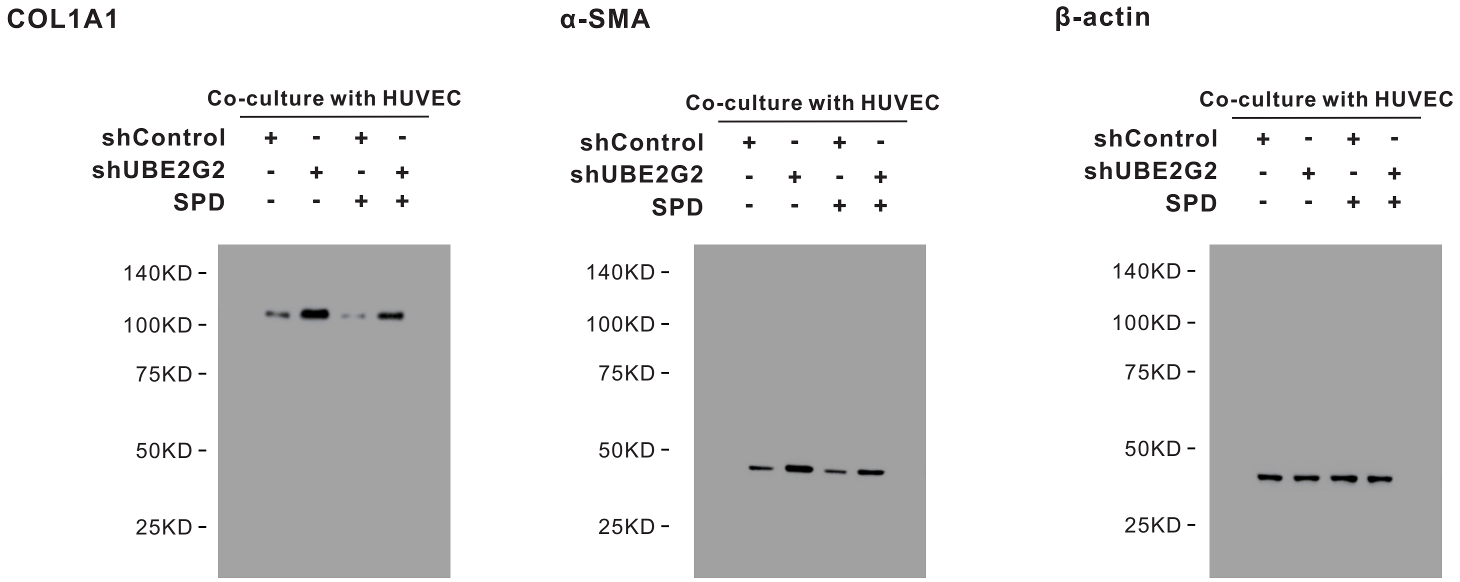
COL1A1
α-SMA
β-actin
Co-culture with HUVEC
shControl + - + -
shUBE2G2 - + - +
SPD - - + +
140KD
100KD
75KD
50KD
25KD

Figure7D

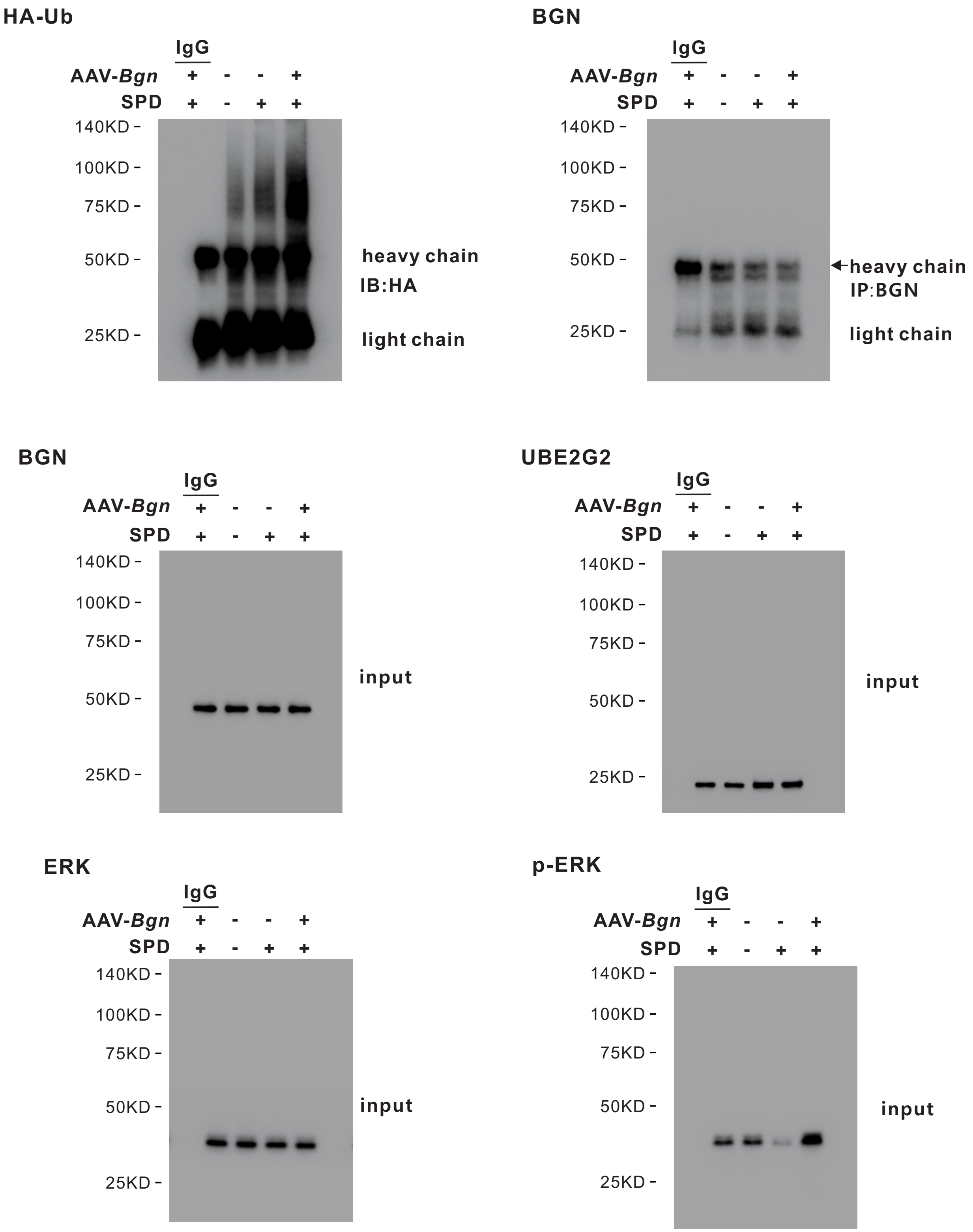
HA-Ub
IgG
AAV-*Bgn* + - - +
SPD + - + +
140KD
100KD
75KD
50KD
25KD
heavy chain
IB:HA
light chain
BGN
IgG
AAV-*Bgn* + - - +
SPD + - + +
140KD
100KD
75KD
50KD
25KD
heavy chain
IP:BGN
light chain
BGN
IgG
AAV-*Bgn* + - - +
SPD + - + +
140KD
100KD
75KD
50KD
25KD
input
UBE2G2
IgG
AAV-*Bgn* + - - +
SPD + - + +
140KD
100KD
75KD
50KD
25KD
input
ERK
IgG
AAV-*Bgn* + - - +
SPD + - + +
140KD
100KD
75KD
50KD
25KD
input
p-ERK
IgG
AAV-*Bgn* + - - +
SPD + - + +
140KD
100KD
75KD
50KD
25KD
input

P38

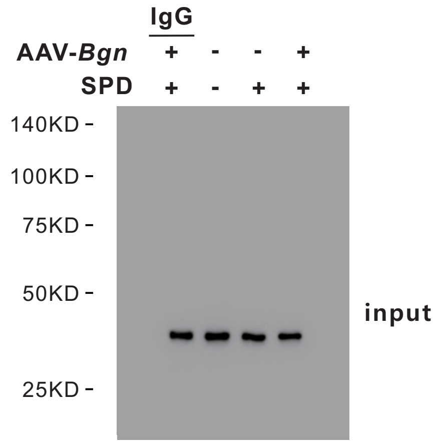

p-P38

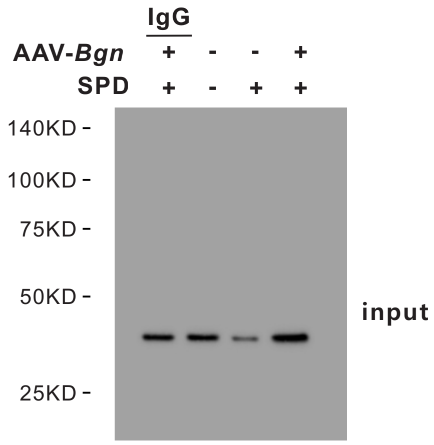

CD34

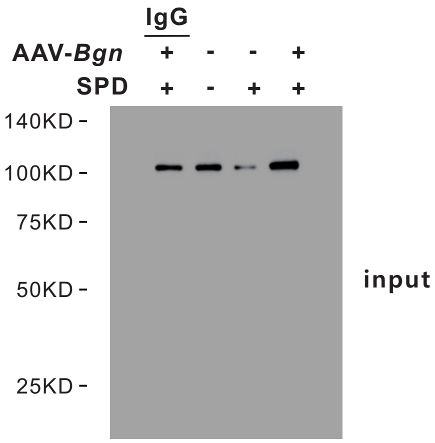

LYVE1

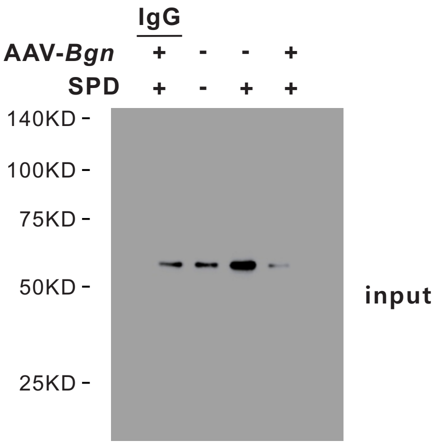

GAPDH

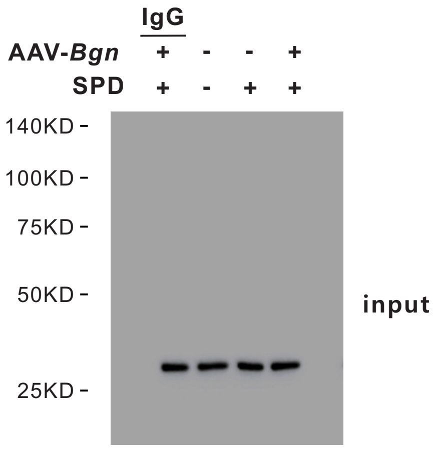

Figure7I

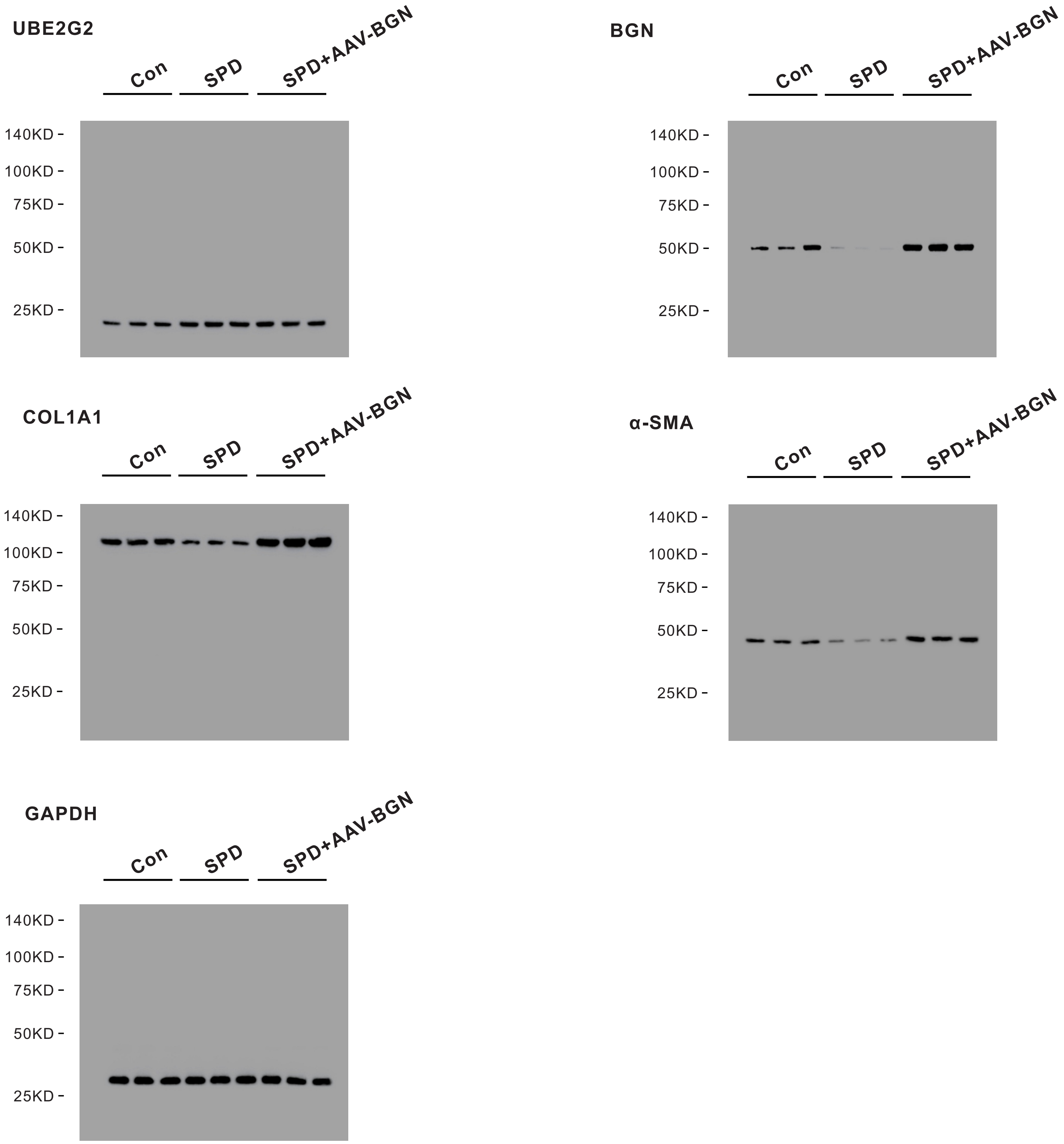

UBE2G2
Con
SPD
SPD+AAV-BGN
140KD
100KD
75KD
50KD
25KD
BGN
Con
SPD
SPD+AAV-BGN
140KD
100KD
75KD
50KD
25KD
COL1A1
Con
SPD
SPD+AAV-BGN
140KD
100KD
75KD
50KD
25KD
α-SMA
Con
SPD
SPD+AAV-BGN
140KD
100KD
75KD
50KD
25KD
GAPDH
Con
SPD
SPD+AAV-BGN
140KD
100KD
75KD
50KD
25KD

FigureS2E left

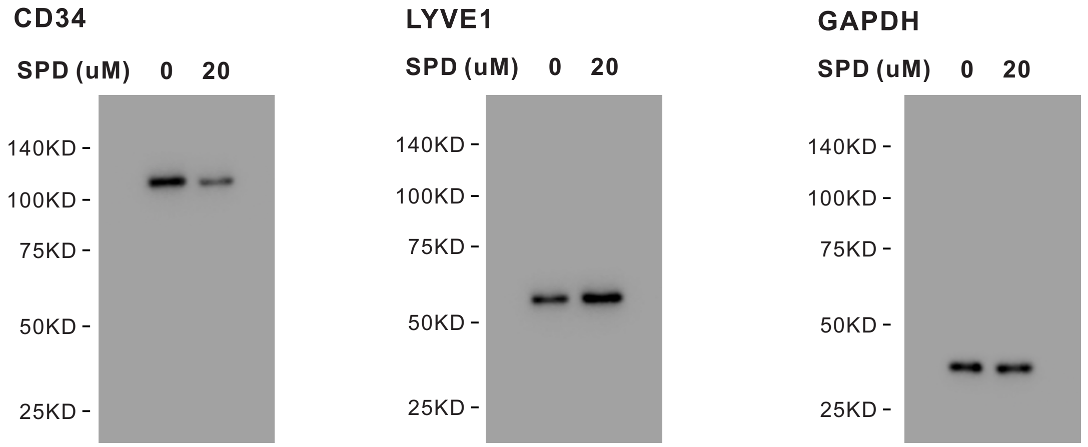

FigureS2E right

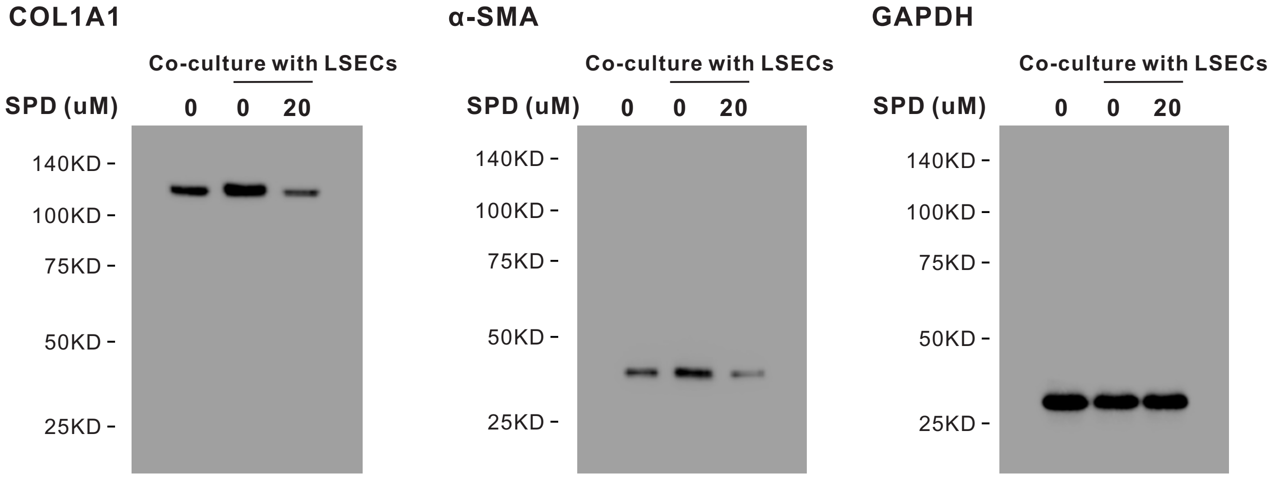

FigureS3F

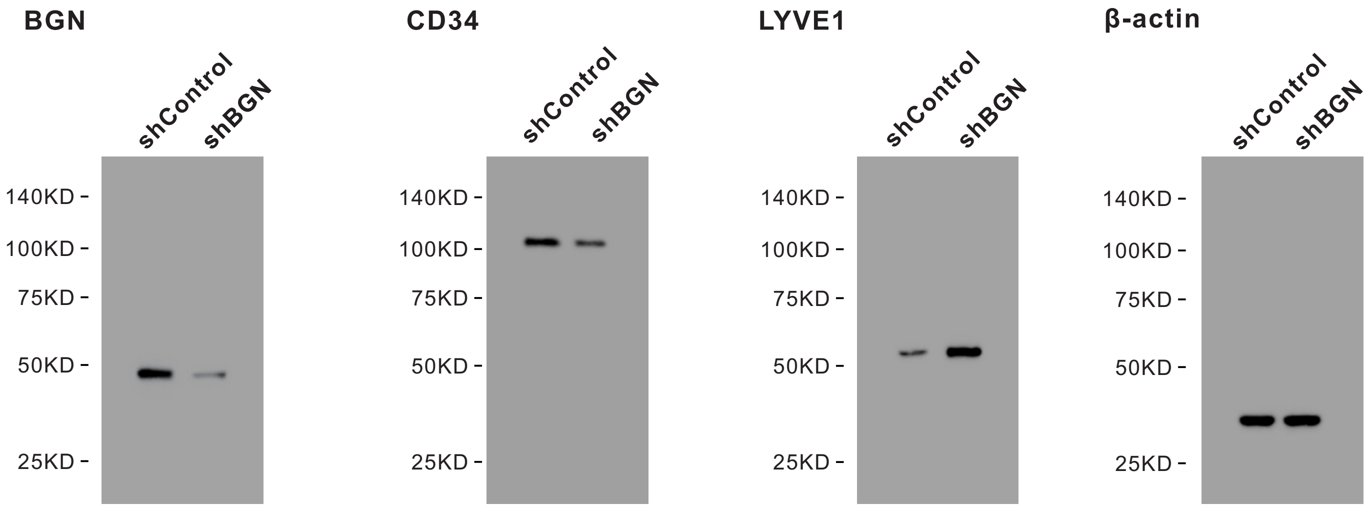

FigureS3I left

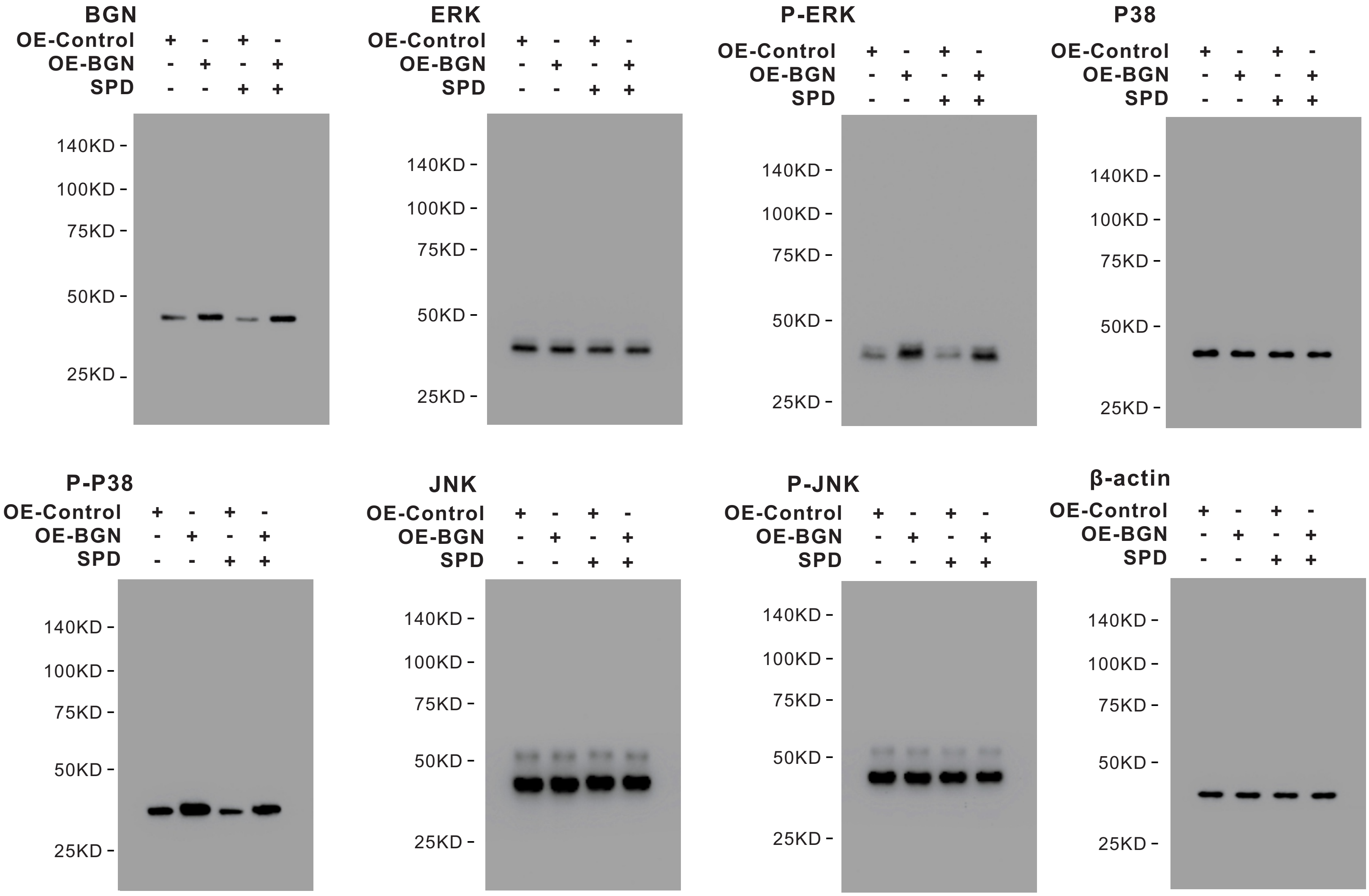

FigureS3I right

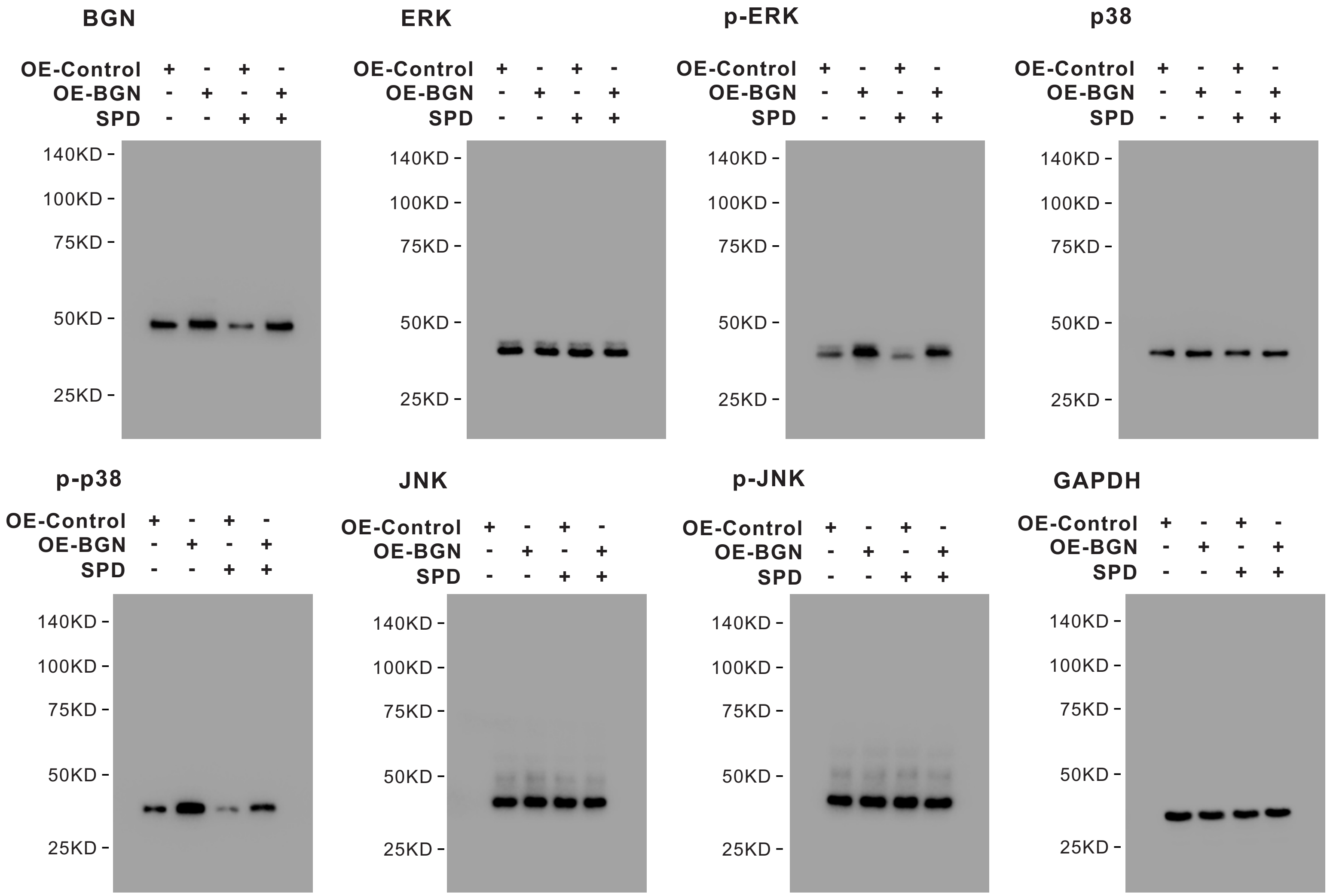

FigureS3J

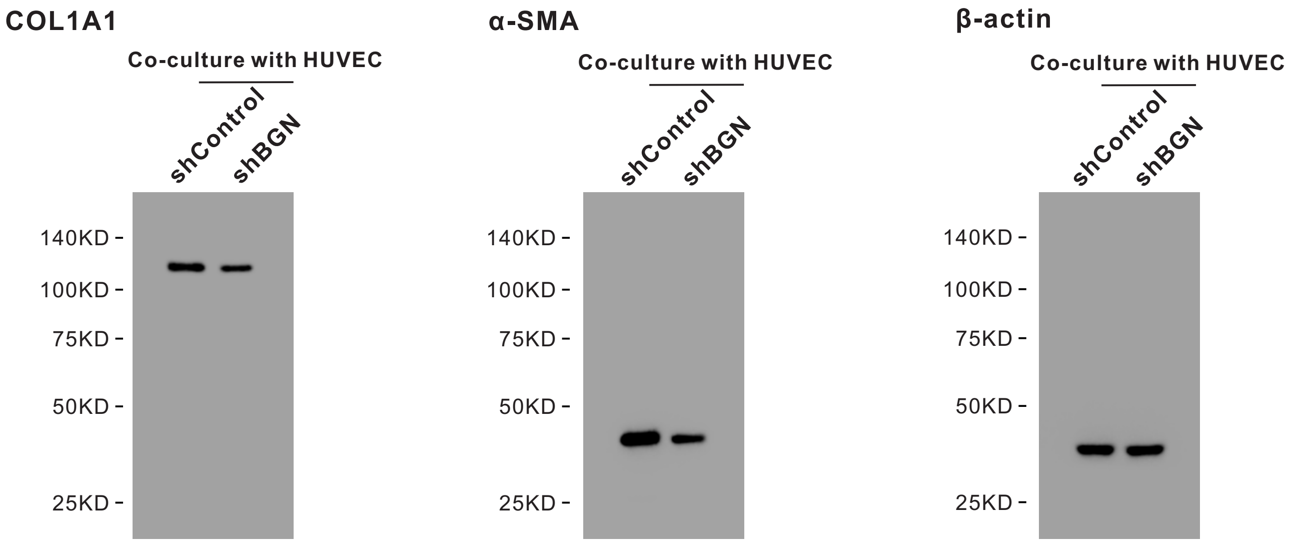

FigureS4B

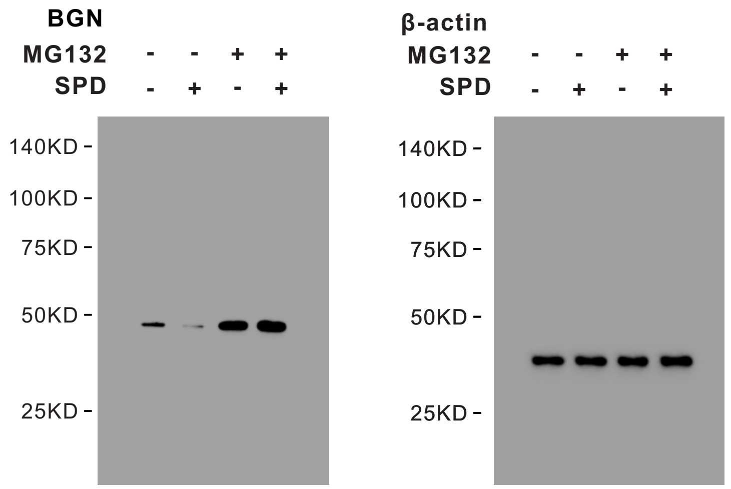

FigureS4C

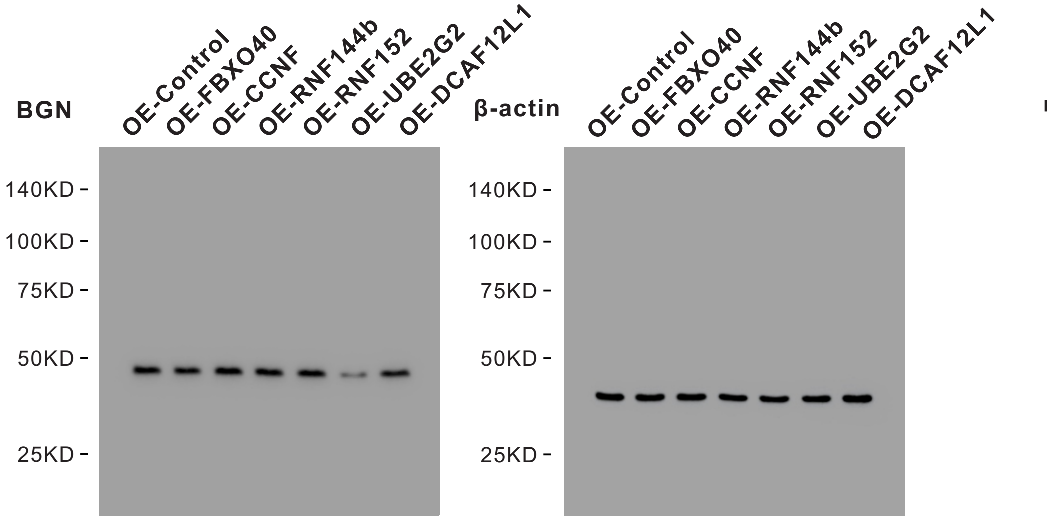

FigureS4G

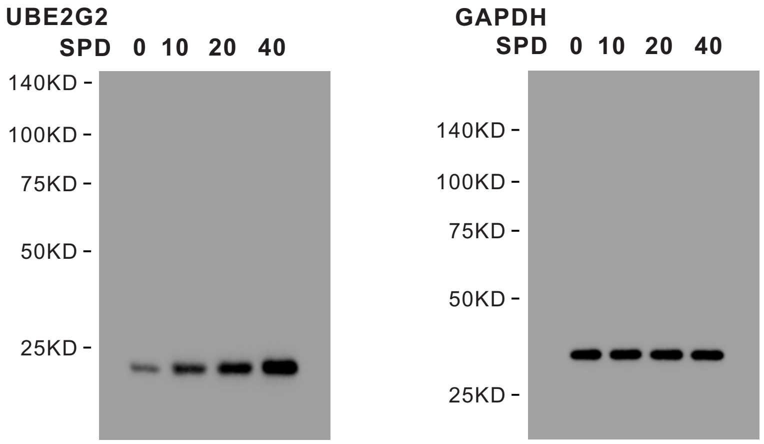

FigureS4H

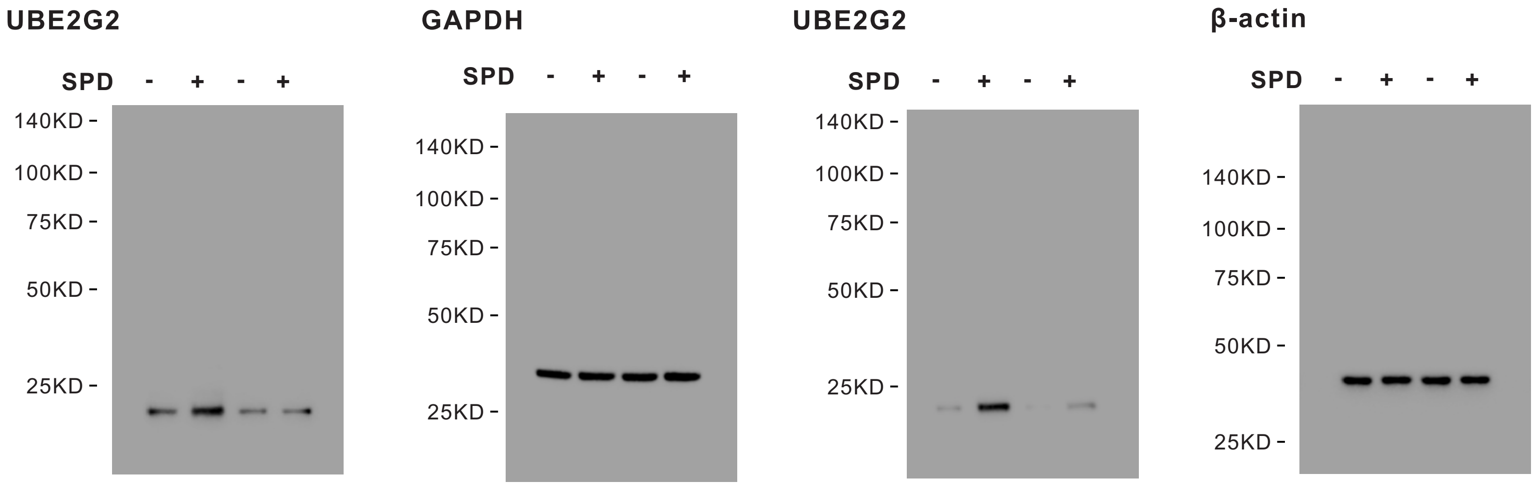

FigureS4I

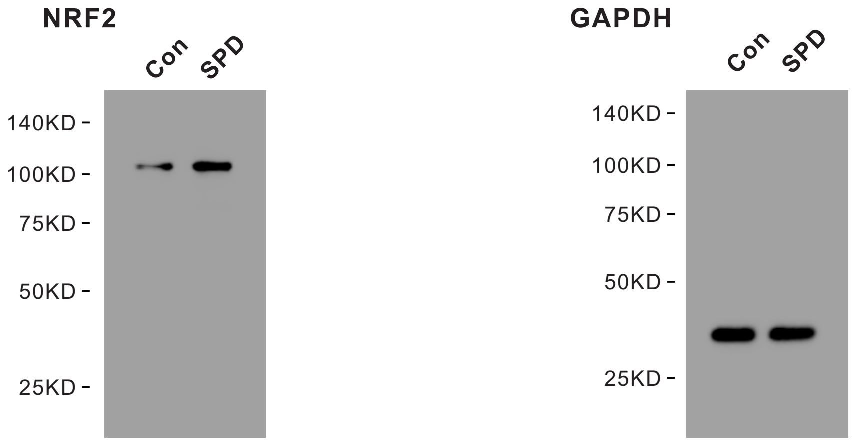

FigureS4J

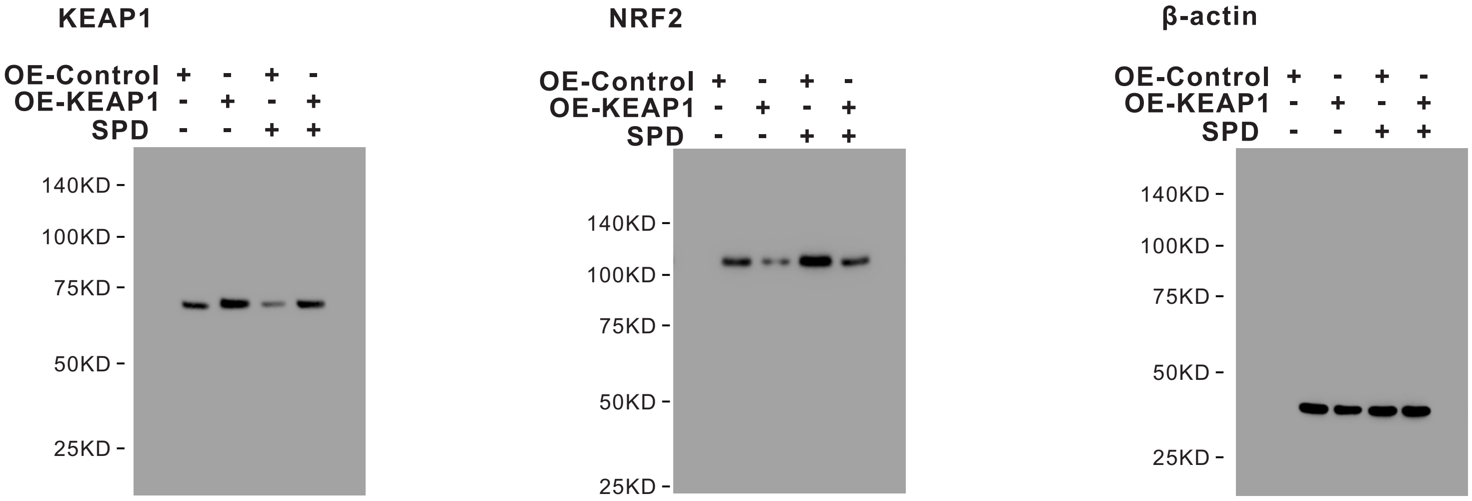

FigureS5A

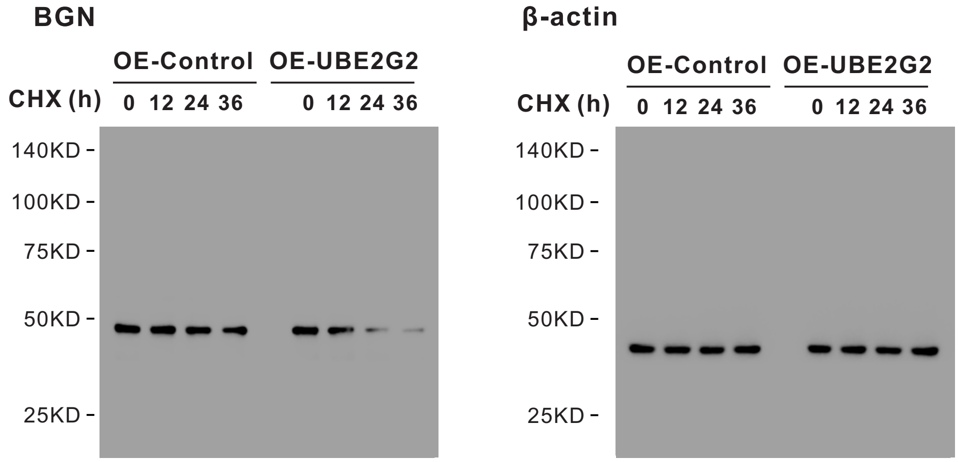

FigureS5B

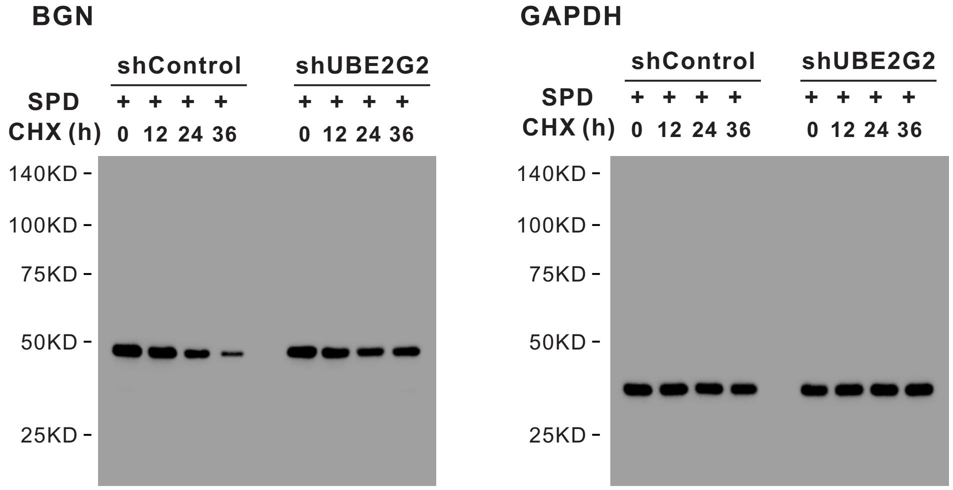

FigureS5C

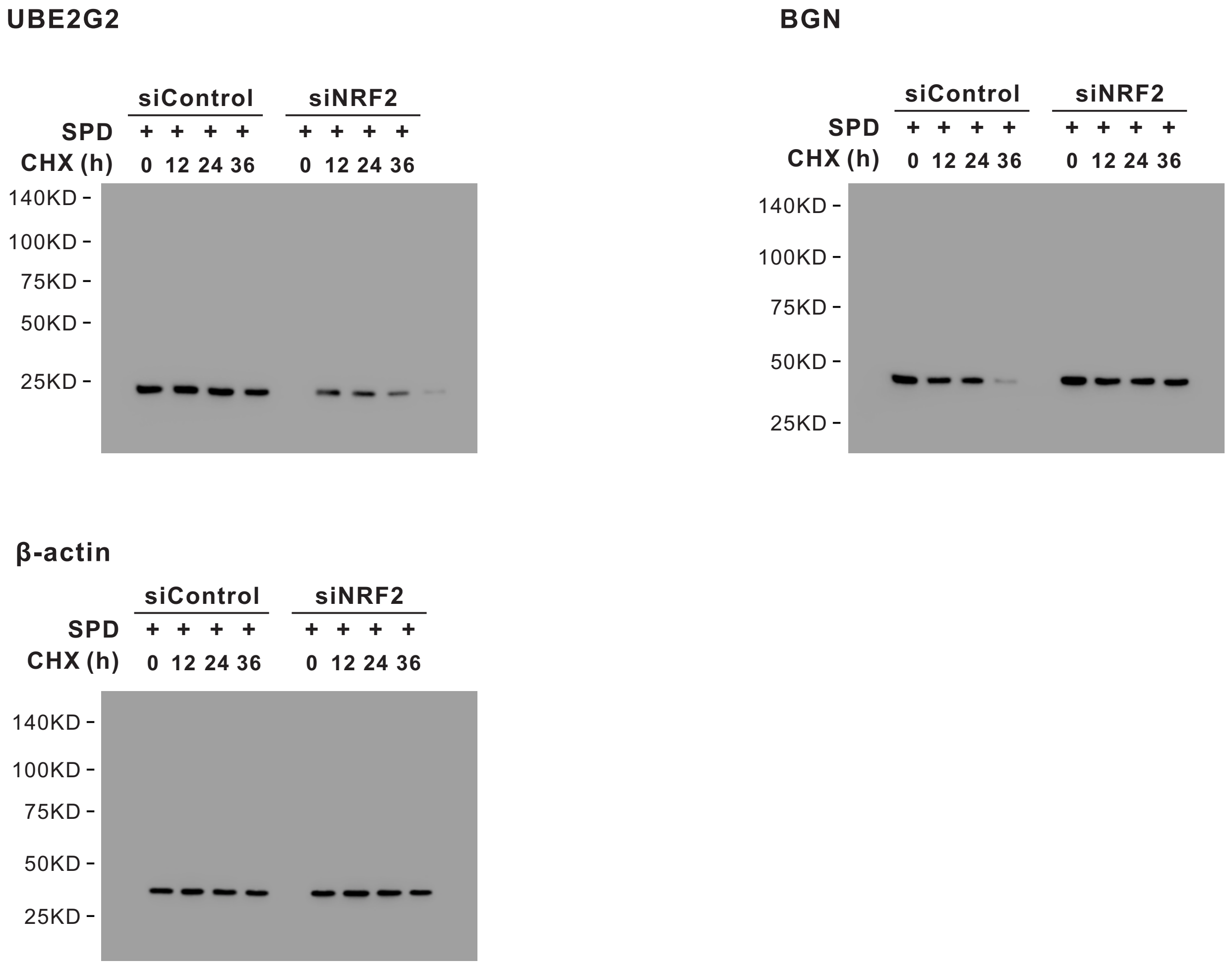

FigureS5D

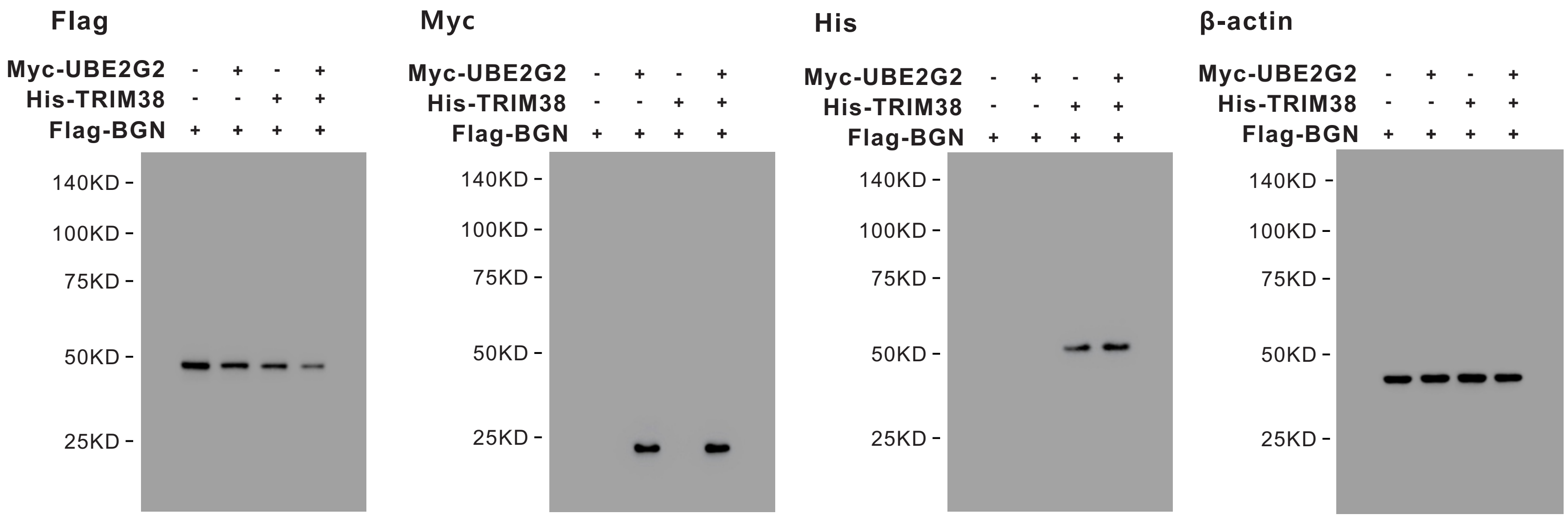

FigureS5E

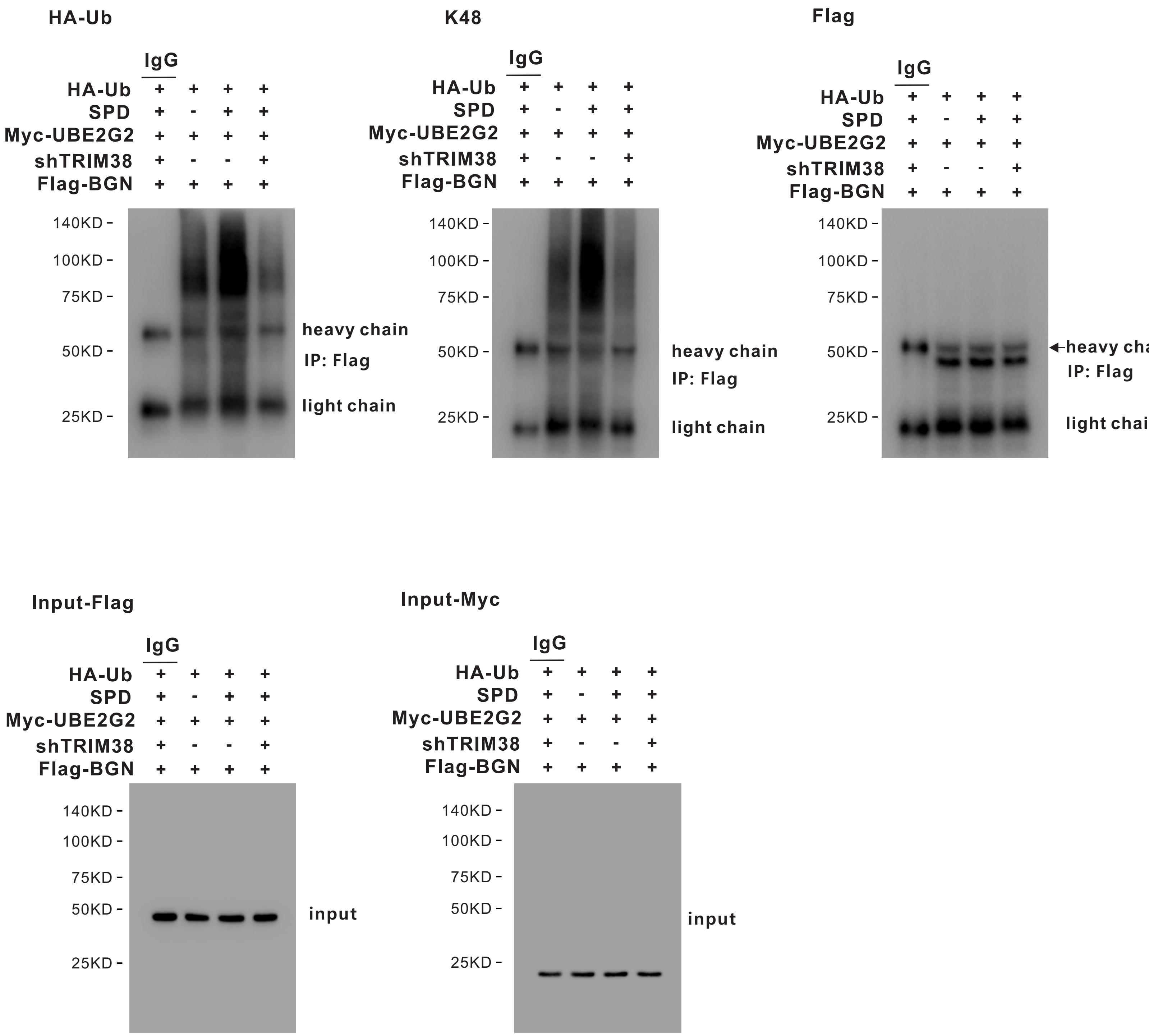
HA-Ub
IgG
HA-Ub + + + +
SPD + - + +
Myc-UBE2G2 + + + +
shTRIM38 + - - +
Flag-BGN + + + +
140KD
100KD
75KD
50KD
25KD
heavy chain
IP: Flag
light chain
K48
IgG
HA-Ub + + + +
SPD + - + +
Myc-UBE2G2 + + + +
shTRIM38 + - - +
Flag-BGN + + + +
140KD
100KD
75KD
50KD
25KD
heavy chain
IP: Flag
light chain
Flag
IgG
HA-Ub + + + +
SPD + - + +
Myc-UBE2G2 + + + +
shTRIM38 + - - +
Flag-BGN + + + +
140KD
100KD
75KD
50KD
25KD
heavy cha
IP: Flag
light chai
Input-Flag
IgG
HA-Ub + + + +
SPD + - + +
Myc-UBE2G2 + + + +
shTRIM38 + - - +
Flag-BGN + + + +
140KD
100KD
75KD
50KD
25KD
input
Input-Myc
IgG
HA-Ub + + + +
SPD + - + +
Myc-UBE2G2 + + + +
shTRIM38 + - - +
Flag-BGN + + + +
140KD
100KD
75KD
50KD
25KD
input

FigureS5F

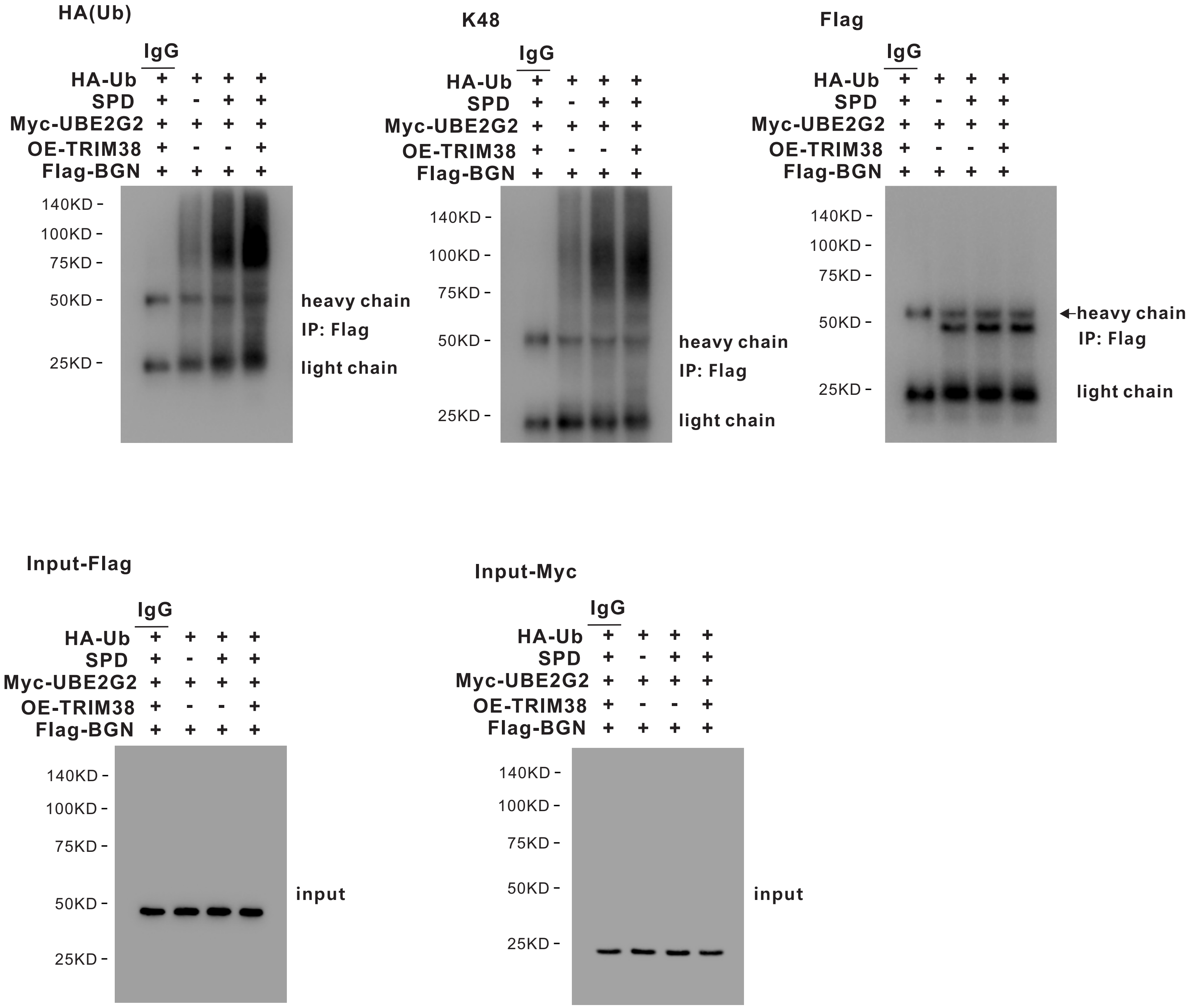
HA(Ub)
K48
Flag
IgG
HA-Ub + + + +
SPD + - + +
Myc-UBE2G2 + + + +
OE-TRIM38 + - - +
Flag-BGN + + + +
140KD
100KD
75KD
50KD
25KD
heavy chain
IP: Flag
light chain
Input-Flag
Input-Myc
input

FigureS6B

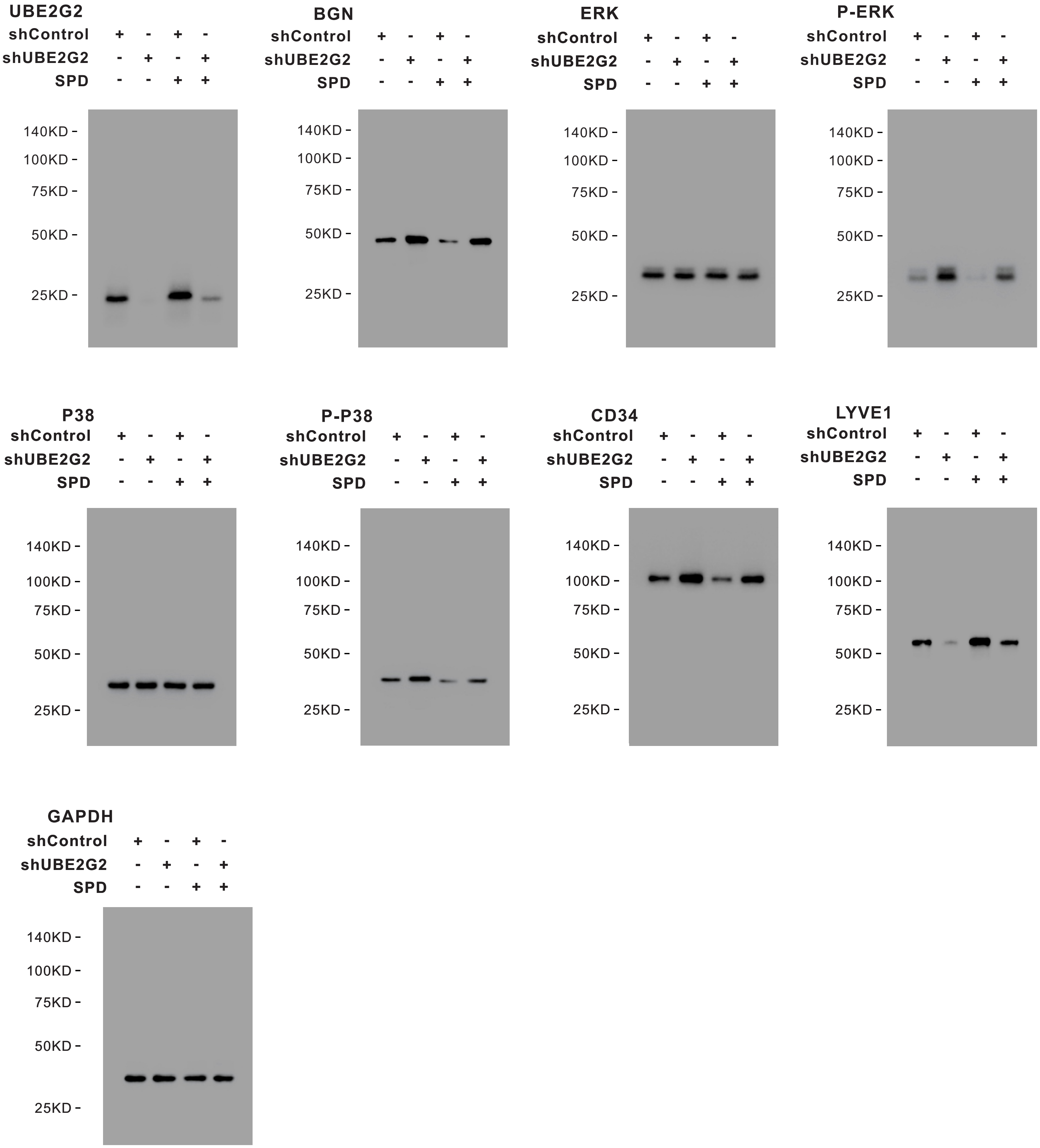
UBE2G2
shControl + - + -
shUBE2G2 - + - +
SPD - - + +
140KD
100KD
75KD
50KD
25KD
BGN
shControl + - + -
shUBE2G2 - + - +
SPD - - + +
140KD
100KD
75KD
50KD
25KD
ERK
shControl + - + -
shUBE2G2 - + - +
SPD - - + +
140KD
100KD
75KD
50KD
25KD
P-ERK
shControl + - + -
shUBE2G2 - + - +
SPD - - + +
140KD
100KD
75KD
50KD
25KD
P38
shControl + - + -
shUBE2G2 - + - +
SPD - - + +
140KD
100KD
75KD
50KD
25KD
P-P38
shControl + - + -
shUBE2G2 - + - +
SPD - - + +
140KD
100KD
75KD
50KD
25KD
CD34
shControl + - + -
shUBE2G2 - + - +
SPD - - + +
140KD
100KD
75KD
50KD
25KD
LYVE1
shControl + - + -
shUBE2G2 - + - +
SPD - - + +
140KD
100KD
75KD
50KD
25KD
GAPDH
shControl + - + -
shUBE2G2 - + - +
SPD - - + +
140KD
100KD
75KD
50KD
25KD

FigureS6D

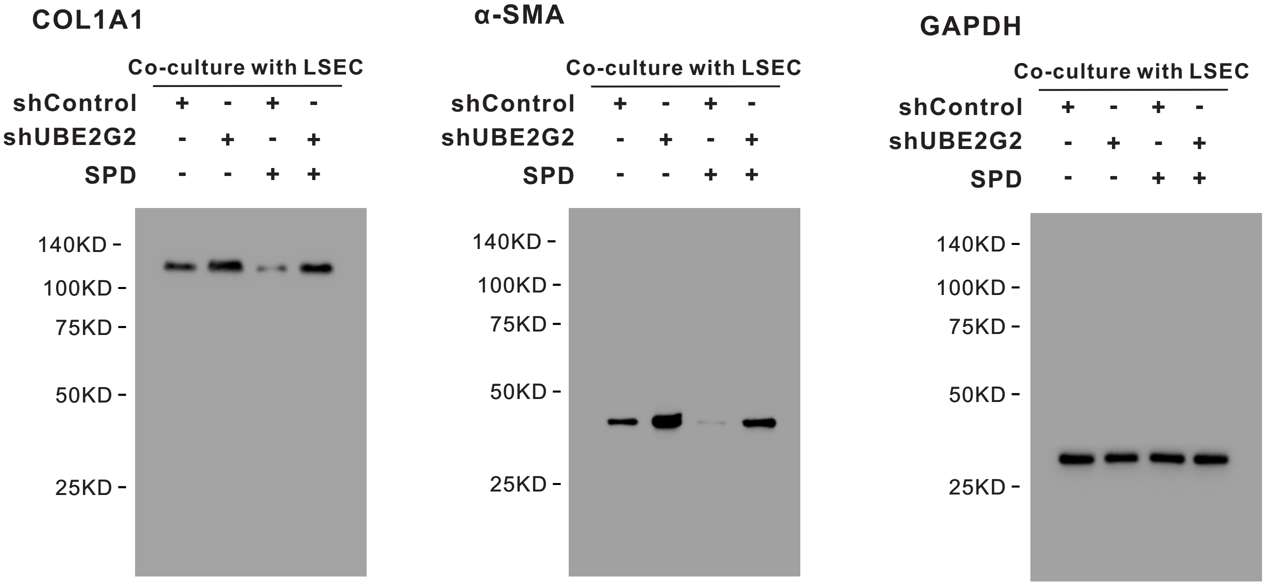
COL1A1
Co-culture with LSEC
shControl + - + -
shUBE2G2 - + - +
SPD - - + +
140KD
100KD
75KD
50KD
25KD
α-SMA
Co-culture with LSEC
shControl + - + -
shUBE2G2 - + - +
SPD - - + +
140KD
100KD
75KD
50KD
25KD
GAPDH
Co-culture with LSEC
shControl + - + -
shUBE2G2 - + - +
SPD - - + +
140KD
100KD
75KD
50KD
25KD

FigureS7A

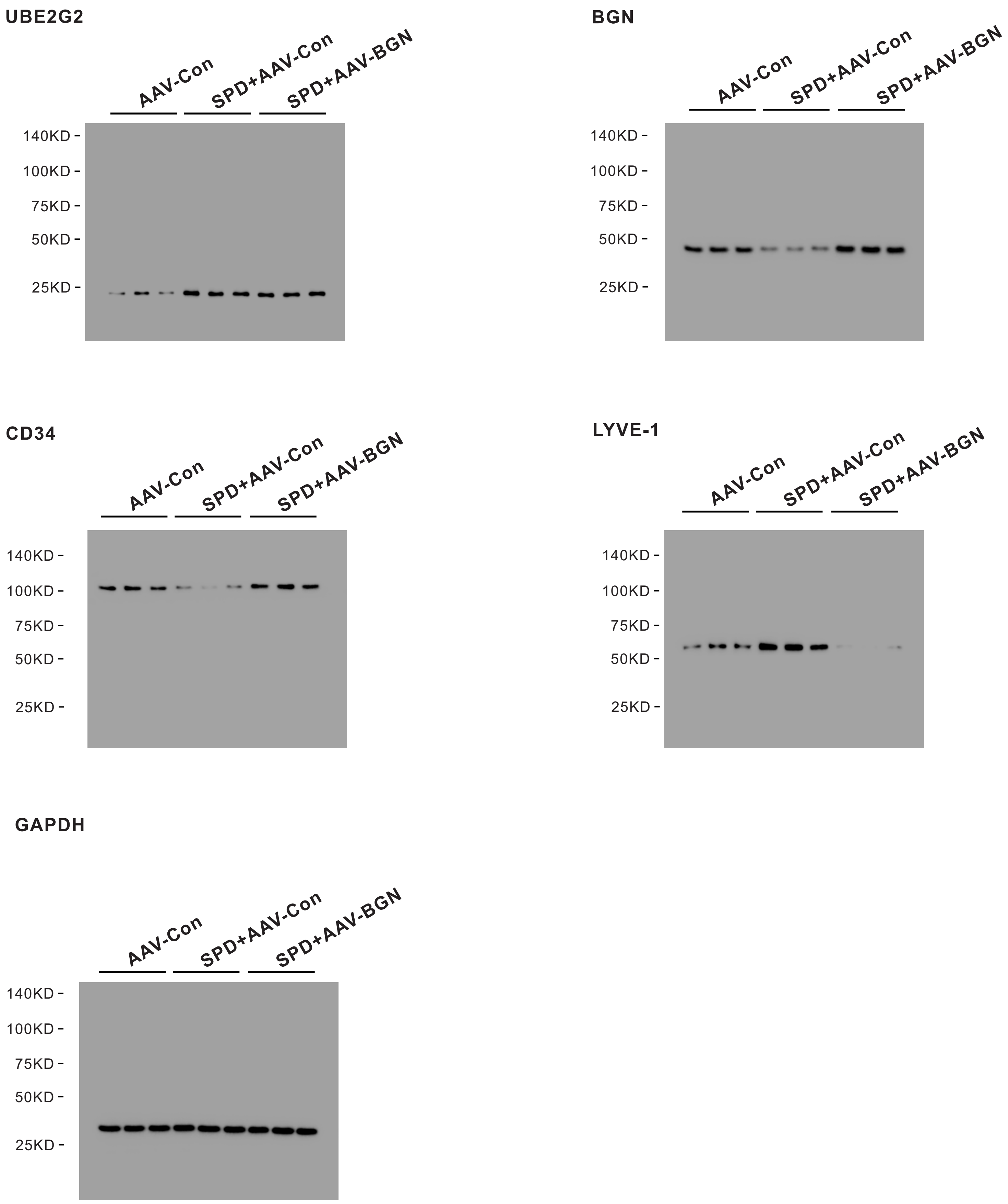
UBE2G2
AAV-Con
SPD+AAV-Con
SPD+AAV-BGN
140KD
100KD
75KD
50KD
25KD
BGN
AAV-Con
SPD+AAV-Con
SPD+AAV-BGN
140KD
100KD
75KD
50KD
25KD
CD34
AAV-Con
SPD+AAV-Con
SPD+AAV-BGN
140KD
100KD
75KD
50KD
25KD
LYVE-1
AAV-Con
SPD+AAV-Con
SPD+AAV-BGN
140KD
100KD
75KD
50KD
25KD
GAPDH
AAV-Con
SPD+AAV-Con
SPD+AAV-BGN
140KD
100KD
75KD
50KD
25KD
